# Single-Locus versus Multilocus Patterns of Local Adaptation to Climate in Eastern White Pine (*Pinus strobus*, Pinaceae)

**DOI:** 10.1371/journal.pone.0158691

**Published:** 2016-07-07

**Authors:** Om P. Rajora, Andrew J. Eckert, John W. R. Zinck

**Affiliations:** 1 Faculty of Forestry and Environmental Management, University of New Brunswick, Fredericton, New Brunswick, Canada; 2 Department of Biology, Virginia Commonwealth University, Richmond, Virginia, United States of America; Austrian Federal Research Centre for Forests BFW, AUSTRIA

## Abstract

Natural plant populations are often adapted to their local climate and environmental conditions, and populations of forest trees offer some of the best examples of this pattern. However, little empirical work has focused on the relative contribution of single-locus versus multilocus effects to the genetic architecture of local adaptation in plants/forest trees. Here, we employ eastern white pine (*Pinus strobus*) to test the hypothesis that it is the inter-genic effects that primarily drive climate-induced local adaptation. The genetic structure of 29 range-wide natural populations of eastern white pine was determined in relation to local climatic factors using both a reference set of SSR markers, and SNPs located in candidate genes putatively involved in adaptive response to climate. Comparisons were made between marker sets using standard single-locus outlier analysis, single-locus and multilocus environment association analyses and a novel implementation of Population Graphs. Magnitudes of population structure were similar between the two marker sets. Outlier loci consistent with diversifying selection were rare for both SNPs and SSRs. However, genetic distances based on the multilocus among population covariances (cGD) were significantly more correlated to climate, even after correcting for spatial effects, for SNPs as compared to SSRs. Coalescent simulations confirmed that the differences in mutation rates between SSRs and SNPs did not affect the topologies of the Population Graphs, and hence values of cGD and their correlations with associated climate variables. We conclude that the multilocus covariances among populations primarily reflect adaptation to local climate and environment in eastern white pine. This result highlights the complexity of the genetic architecture of adaptive traits, as well as the need to consider multilocus effects in studies of local adaptation.

## Introduction

Natural plant populations are often adapted to their local climate and environmental conditions [1,2]. Populations of forest trees offer some of the best examples of this pattern, with over a century of observation and experimentation documenting this phenomenon [3]. For example, common garden and provenance test studies have demonstrated repeatedly that forest trees exhibit strong geographic variation for fitness-related phenotypes, which is often correlated with environmental variables measured in the source populations [4]. Such patterns are expected for populations that are adapted to their local environments [5]. The loci comprising the genetic architecture underlying local adaptation for forest trees, however, are largely unknown [3]. This dearth of knowledge stems partly from the complexity of the underlying genetic architecture of local adaptation in forest trees relative to the types of statistical methods used to document local adaptation from genetic data. Specifically, statistical methods used to detect loci as contributing to the genetic architecture of local adaptation often focus on single-locus effects, whereas the underlying genetic architecture of local adaptation for forest trees likely involves the among-population component of intergenic linkage disequilibrium [6–11].

Some of the best examples of local adaptation for forest tree populations are found for quantitative traits as opposed to simple Mendelian traits, *e*.*g*. [12], reviewed in [10]. Quantitative traits with strong relationships to various fitness components, such as date of bud set or bud flush *cf*. [13], are expected to be polygenic in forest trees [14]. There are two ways for diversifying natural selection to produce locally adapted phenotypes based on polygenic traits—small allele frequency changes that result in the build-up of excessive multilocus covariances among populations and large allele frequency changes eventually resulting in fixation of the most beneficial allele at each of the loci in each of the populations [6–10]. Although the relative contribution of the two processes depends on the genetic architecture of the phenotypic trait and its relationship to fitness, the demographic history of the populations, the type and strength of selection, and timing for the onset of selection [8,15], the among-population component of multilocus covariance can become the main driving force of adaptive genetic differentiation when a phenotypic trait is controlled by several genes [8,10,16]. In these cases, genetic differentiation at each of the causative loci is closer to the overall level of neutral differentiation [10]. This results in a large discrepancy between differentiation of fitness-related phenotypic traits and the allelic differentiation at the loci controlling these traits.

The most commonly used method of identifying loci as contributing to local adaptation among natural plant populations, especially forest trees, is the detection of single-locus outliers, such as those based on *F*_ST_ [17–19] or those based on single-locus effects in relation to the overall variance-covariance structures among populations [20–22], which are often conducted using scans of candidate genes. Outliers detected by these methods typically have large, or at least the largest among the loci surveyed, allele frequency differences among sampled populations. Many causative loci, therefore, are unlikely to be detected using scans for *F*_ST_ outliers, since the conditions for large allele frequency differences to arise among populations of forest trees may be rare [23]. For example, Ma *et al*. [24], using SNPs from 25 candidate genes involved in the photoperiodic pathway and circadian clock in *Populus tremula*, noted that none of the four SNPs showing significant allele-frequency clines with latitude or the six SNPs associated with growth cessation were *F*_ST_ outliers. Additionally, epistatic selection was found to be strong at fine spatial scales in *Fagus sylvatica*, whereas *F*_ST_ outliers within candidate genes were rare [25].

Pioneering empirical work in deer mice (*Peromyscus maniculatus*) used patterns of linkage disequilibrium to document the important role of intergenic covariance to local adaptation [26]. It is the among-population component of these intergenic covariances that is the relevant quantity [6], and, as shown for deer mice, this component can be extensive and can be used to identify putatively causative genes and gene regions. Limited examples are also available for forest trees, although most studies searched for these patterns only among loci identified using single-locus tests, *e*.*g*. [24]. This approach, however, ignores loci not labeled as outliers, although under many selection regimes and demographic models it is the covariances among these unremarkable loci that affect differentiation among populations for fitness-related quantitative traits [27]. Alternatively, the use of genetic marker data as multilocus genotypic vectors implicitly captures these interlocus covariances, so that it may be more fruitful to begin with a multilocus analysis prior to investigation of single-locus effects [6,28,29].

Local adaptation among forest tree populations has been extensively documented despite recent divergences among populations and extensive gene flow [3,29–31]. Climate is likely the main driver of these patterns across many species [4,32,33]. A multitude of scans for *F*_ST_ outliers, however, have resulted in relatively limited lists of putatively causal genes [10]. Here we address this disconnect using a comparison between markers located within candidate genes putatively responsive to climate factors and a set of reference genetic markers genotyped for a range-wide sample comprised of 29 eastern white pine (*Pinus strobus* L.) populations.

**Fig 1:**
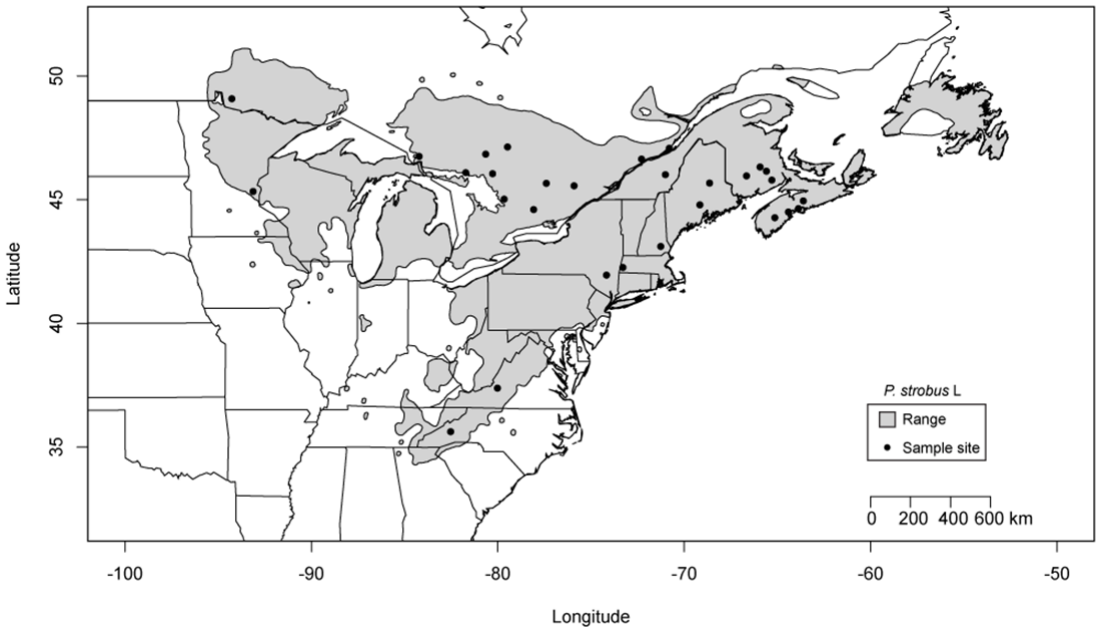
The distribution of eastern white pine in relation to sampled populations. The shaded area represents the natural range of eastern white pine.

Eastern white pine provides an ideal experimental organism for studying genetic architecture of local adaptation. It is a wide-ranging conifer species that inhabits diverse forested ecosystems across North America, with a range spanning the temperate forests of the southern Appalachian Mountains to the boreal forests north of the Great Lakes [34] (Fig 1). Its wide geographical range results in populations inhabiting highly diverse climate and environmental conditions (Fig 1). The range establishment of this species is recent, especially in terms of *N*_e_ generations [32], so that selective pressures imposed by this climate disparity have had relatively a small number of generations to shape allele frequencies. Despite this recent colonization, a multitude of studies have documented differences among populations for adaptive traits at multiple spatial scales [35–39].

Most of the previous studies on molecular genetic diversity and population genetic structure of eastern white covered small geographic areas in Ontario [40–42], Michigan [43] and Wisconsin [44], as well as for a limited number of populations from different parts of Canada [45,46]. These studies were conducted using allozymes and SSRs primarily to examine the genetic effects of harvesting or questions of central-marginal population dynamics. Most recently, range-wide genetic diversity has been examined in this species based on nuclear and chloroplast genetic markers [47,48]. Low magnitudes of genetic structure were observed for eastern white pine populations from Canada for both allozyme (*F*_ST_ = 0.061) [45] and microsatellite (*F*_ST_ = 0.084, 0.104) [46,47] markers. However, genetic architecture of local adaptation is poorly understood in this species.

Our goal was to assess the relative contributions of single-locus versus multilocus effects to climate adaptation for datasets of realistic sizes. First, we establish that allele frequencies at single nucleotide polymorphisms (SNPs) within a set of candidate genes are overly correlated to bioclimatic variables relative to putatively neutral microsatellites (SSRs). We then establish that this result is driven by differences between putatively selective and neutral marker types in the pattern of conditional covariances in allele frequencies across populations rather than enrichment of allele frequency outliers within the candidate gene SNPs.

## Materials and Methods

### Ethics statement—Field sampling

The studied eastern white pine populations are located on public lands in Canada and USA, which are not designated as protected areas. The field sampling was done in consultation with the representatives of the agencies that manage public forests. Therefore, no specific permission was required for field sampling from the studied locations. Our study did not involve an endangered species.

### Populations and sampling

A total of 29 eastern white pine populations from throughout its range were sampled (Table 1; Fig 1). The populations were sampled along the south to north transect spanning in latitude from 35.62° to 46.32° in the east and 49.05° in the west, and in the east-west direction ranging from longitude of 63.61°W to 94.27°W. This sampling scheme covers diverse climate conditions with south to north gradient in temperature and east to west gradient in precipitation (Fig 1), and variation in photoperiod regimes to which eastern white pine has to adapt over its natural range. All of the sampled populations were located in natural forests. Fifty mature trees were sampled randomly from each population. Since selection alters allele frequencies, accurate estimates of allele frequencies are essential to detect signals of selection. An adequate sample size is required for reliable estimation of allele frequencies. Therefore, we chose to have a large sample size per population. The sampled trees within a population were separated by 30 m to minimize the chance of sampling siblings. We collected needles from each of the sampled mature trees. Each needle sample was stored in a sealed plastic bag, with a 5 g silica desiccant pack, at -20°C pending DNA extraction. Total genomic DNA was extracted from needle tissues of individual sampled trees using a modified CTAB method [49].

**Table 1:** Populations of eastern white pine (*Pinus strobus*) sampled for this study and their geographic coordinates. Bioclimatic data are given by population in the associated data file (S10 Table) for this study.

Population	Population ID	Longitude	Latitude
New Brunswick—Canaan River	NBCI	-65.59	46.15
New Brunswick—Chipman Road	NBCR	-65.93	46.32
New Brunswick—Odell Park	NBOP	-66.66	45.96
New Brunswick—Paper Mill Hill	NBPH	-65.30	45.79
Massachusetts—Stockbridge	MASB	-73.29	42.26
Maine- Baxter State Park	MEBP	-68.64	45.67
Maine—Etna Brook	MEEB	-69.16	44.79
New Hampshire—Deerfield	NHDF	-71.26	43.11
New York—Pacama Catskills	NYCM	-74.17	41.95
Nova Scotia—Dory Mills Lake	NSDL	-64.41	44.50
Nova Scotia—Lake Rossignol	NSRL	-65.14	44.27
Nova Scotia—Saint Margret's Bay	NSMB	-63.87	44.64
Nova Scotia—Uniacke	NSUM	-63.61	44.95
Minnesota—Boot Lake	MNBL	-93.13	45.33
Ontario—Crow Lake	ONCL	-94.27	49.08
Ontario—French River	ONFR	-80.28	46.05
Ontario—Goulais River	ONGR	-84.22	46.75
Ontario—High Falls	ONHF	-78.08	44.60
Ontario—Muskoka	ONML	-79.66	45.02
Ontario—Whitefish Reserve	ONMF	-81.72	46.09
Ontario—Renfrew County	ONRC	-77.40	45.66
Ontario—Timiskaming	ONTO	-79.48	47.13
Ontario—Wolf Lake	ONWL	-80.65	46.84
Quebec—Cap Tourmente	PQCT	-70.80	47.08
Quebec—Lac Phillip	PQLP	-75.91	45.56
Quebec—Saint Renyold	PQSR	-71.01	46.01
Quebec—Saint Stanilis	PQSS	-72.29	46.64
Virginia—Bennett Springs	VASB	-80.02	37.38
North Carolina—Asheville	NCAV	-82.53	35.62

### Climate data

Climate data were retrieved from the WorldClim global climate layers (v. 1.4 release 3) [50]. We chose to focus on the 19 bioclimatic variables, which are functions of seasonal temperature and precipitation variables, due to their common use to model species distribution patterns [51]. The relevant climate grids were obtained from the WorldClim website (http://www.worldclim.org/current) at a resolution of 30 seconds (~1 km resolution) from which the 19 bioclimatic variables were extracted using the geographical coordinates of each population and the raster library of R [52]. Each of the bioclimatic variables was mean-centered and standardized prior to use in downstream analysis. For single-locus analyses, all 19 bioclimatic variables were reduced into a set of principal components (PCs). For multilocus analyses, the 19 centered and standardized bioclimatic variables were then trimmed based on their pairwise correlations, so that only bioclimatic variables with Pearson correlation coefficients (*r*) in the range -0.80 < *r* < 0.80 were retained. This resulted in retention of nine bioclimatic variables (bioclim): bioclim1 (annual mean temperature), bioclim2 (mean diurnal temperature range), bioclim4 (temperature seasonality), bioclim8 (mean temperature of the wettest quarter), bioclim10 (mean temperature of warmest quarter), bioclim13 (precipitation of the wettest month), bioclim14 (precipitation of the driest month), bioclim15 (precipitation seasonality), and bioclim18 (precipitation of the warmest quarter).

### Genetic data

Two types of genetic markers were used—SSRs and SNPs. All 50 sampled trees per population were genotyped for 12 nuclear SSRs (S1 Table) [41,53] as described in [47] and S1 Text, whereas 22 randomly selected trees from 50 sampled trees per population were genotyped for SNPs in climate-related candidate genes. The details on SSR markers and their reference sequences, primers, GenBank accession numbers and BAC annotations are provided in S1 Table.

We used data for 44 SNPs in 25 candidate genes putatively involved in the adaptive response to cold and drought stresses, photoperiodic response, phenology, growth, development, and maintenance of biological processes, cellular integrity and functions under stress conditions caused by climate factors (Table 2; S2 and S3 Tables) and [54,55]. These genes and SNPs were selected from screening 96 candidate genes. We selected expressed sequence tags (ESTs) for 96 candidate genes putatively involved in responses to cold (temperature), photoperiod, and drought (precipitation) as well as genes affecting phenology, growth and development, and maintaining cellular integrity and functions under stress conditions from the existing publications, conifer EST databases, and GenBank [54–56]. Selection of EST contigs from the TreeGenes database [56] was based on their functional annotations as determined using BLAST tools. The EST sequences of the candidate genes were compared against the NCBI *Arabidopsis* protein database using BLASTx. Putative homologs of the candidate genes have been previously demonstrated to be functionally involved in the adaptive responses to climate factors in the model plant, *Arabidopsis* (see S2 Table for 25 candidate genes used in the final dataset). And SNPs in some of them have been found to be associated with cold hardiness, climate-related adaptive genetic differentiation or co-located with cold hardiness QTL in conifers [54,55,57].

**Table 2:** A summary of SNPs, candidate genes and their biological functions from functional analysis of homologues in model plant *Arabidopsis* or other plants. The details on these candidate genes, including EST loci, GenBank and TreeGenes database ID, and references for the identification of biological functions are provided in S2 Table.

SNP ID	Candidate gene	Climate-responsive biological gene function
RPSS03_05	Chaperonin-60 alpha subunit	Plastid division and organization; protein folding; senescence; growth and development
RPSS04_02, RPSS04_03	Ankyrin repeat containing protein	Molecular chaperon; plant cellular metabolism; growth and development; regulation of defense response
RPSS05_01, RPSS05_04, RPSS05_05	Malate dehydrogenase—peroxisomal	Oxidoreduction; carbohydrate metabolic process; chlorophyll biosynthetic process; response to light stimulus; regulation of plant-type hypersensitive response; growth; signal transduction
RPSS06_03	Peroxidase	Response to oxidative stress, and abiotic and biotic stresses
RPSS08_01, RPSS08_03	Caffeoyl-CoA 3-O-methyltransferase	Lignin and flavonoid biosynthesis; abiotic and biotic stress responses and defense
RPSS12_01, RPSS12_03	NADH dehydrogenase subunit 7	Oxidoreductase activities; cellular respiration
RPSS14_03, RPSS14_06	Multidrug resistance associated protein 1	ABC transmembrane transport; cell membrane integrity; abiotic stress response; oxido-reductase activities
RPSS16_01, RPSS16_03	Potassium-dependent sodium-calcium exchanger-like protein	Cation and transmembrane transport; cell membrane integrity; plant nutrition; growth and development; signal transduction
RPSS19_02, RPSS19_03, RPSS19_04, RPSS19_06	Photosystem II cp47 chlorophyll apoprotein	Photosynthesis; chlorophyll binding; growth and phenology
RPSS28_04, RPSS28_06	Elongation factor 2 like protein	Freezing tolerance and cold acclimation; heat tolerance; molecular chaperone
RPSS30_01, RPSS30_02	Metallothionein-like protein (MT-like)	Response to osmotic and other abiotic stresses; oxidative damage control; cellular homeostasis; leaf senescence
RPSS31_01, RPSS31_02	Oxygen evolving complex 33 kda photosystem II protein	Photosynthesis, cold and other abiotic and biotic stress response; cellular cation homeostasis; morphogenesis
RPSS32_03	Calcium-dependent protein kinase	Regulation of stomatal movement, transport, osmotic stress, salt stress, and anion channel activity
RPSS33_01	MYB transcription factor	Regulation of development, metabolism and response to abiotic and biotic stresses
RPSS36_05	Dehydrin	Drought, cold and freezing stress tolerance
RPSS47_04	Permease	Plastid development; plant growth; mineral nutrition; transport of biochemical, such as auxins, ions and metals; protection from oxidative stress; abiotic stress tolerance
RPSS61_02, RPSS61_03, RPSS61_05, RPSS61_06	Glutathione S-transferase	Response to environment; detoxification; protection from oxidative damage
RPSS62_01, RPSS62_02	Cinnamate 4-hydroxylase	Lignin and flavonoid biosynthesis; abiotic and biotic stress responses and defense
RPSS66_04	Heat shock protein	Abiotic stress response and protection of plants; molecular chaperons
RPSS71_02	ADP/ATP translocator or adenine nucleotide translocator (ANT)	Maintenance of mitochondria function and integrity; photosynthesis and respiration; nucleotide transport; growth and development; response to stress; regulation of programmed cell death and plant-type hypersensitive response
RPSS77_04	MYB transcription factor	Regulation of development, metabolism and response to abiotic and biotic stresses
RPSS86_01, RPSS86_02, RPSS86_04, RPSS86_06	Chlorophyll a/b-binding protein type 1 (CABBP1)	Photosynthesis; response to light and its intensity; light harvesting, regulation of stomatal conductance; drought stress response
RPSS87_05	Metallothionein-like protein (MT-like)	Response to osmotic and other abiotic stresses; oxidative damage control; cellular homeostasis; water transport
RPSS96_02	Thiazolebiosynthetic enzyme (TBE)	Response to cold, DNA damage stimulus and light; starch biosynthetic process

Primers for 72 candidate genes yielded PCR amplicons in eastern white pine. We identified SNPs in these 72 unique ESTs in a discovery panel of 32 eastern white pine samples representing populations from the entire range of the species using an EcoTilling approach [58] based on the SNiPer Eco-Mix Kit (Frontier Genomics, Auke Bay, Alaska, USA). Polymorphic fragments were sequenced by Beckman Coulter Genomics (Danvers, Massachusetts, USA). The resulting sequences were quality trimmed using a PHRED threshold score of 20 and aligned. Overall, 184 SNPs in 55 genes were identified. Of these, 155 SNPs in 45 genes, which had a minimum flanking region of 60 bp and met the criteria of multiplex genotyping assay, were used for genotyping. SNP genotypes were determined using the Illumina GoldenGate platform (Illumina, San Diego, CA, USA) as carried out by the Laboratoire Yohan Bossé at the Institut Universitaire de Cardiologie et de Pneumologie de Québec (Québec, PQ, Canada). The SNP arrays were imaged using the Illumina BeadArray technology and analyzed using BeadStudio (v. 3.1.3.0). SNP loci were filtered for those that had call rates greater than 80% and a minor allele frequency greater than 1%. One hundred forty-one SNPs in 45 genes met the first criterion. And 44 SNPs in 25 genes (Table 2; S2 and S3 Tables) met both of these criteria and, thus, were used in data analysis.

### Population genetic analysis

Genetic diversity of the sampled populations was quantified for each dataset (*i*.*e*. SSRs and SNPs) using standard statistics (effective number of alleles: *A*_E_, observed heterozygosity: *H*_O_, expected heterozygosity: *H*_*E*_), and Wright’s fixation index (*F*_IS_) was calculated from these values. These parameters were estimated for each locus in each population. Averages across loci within populations were used to represent the overall multilocus patterns. Correlations involving multilocus averages within populations were assessed using Pearson correlations, the significance of which were assessed via permutation of population identifiers (*n* = 9,999) at α = 0.05.

Genetic structure among the sampled populations was estimated to test the hypothesis that it differs between reference (*i*.*e*. SSRs) and putatively functional candidate gene (*i*.*e*. SNPs) marker sets. We focused specifically on both aspects of genetic structure: its magnitude [59,60] and its shape [61]. Patterns of population structure were thus analyzed using two approaches: (1) summary statistics to assess its magnitude (*e*.*g*. *F*_ST_) and (2) Population Graphs to assess its shape.

We used the *hierfstat* library in R to estimate multilocus values of *F*_ST_ for each dataset [62]. Bootstrapping over loci (*n* = 10,000) was used to construct 95% confidence intervals for each estimate. We tested a null model of no differences in the magnitude of genetic structure between marker sets using the 95% bootstrap confidence intervals for the multilocus estimates for each marker type. For comparison, we also estimated *G*_ST_’ using the *gstudio* library in R. This approach accounts for differences in heterozygosities between marker types [63].

Outliers with respect to the magnitude of *F*_ST_ were identified using BayeScan ver. 2.1. Separate analyses were conducted for each marker type. This approach partitions the observed values of *F*_ST_ into effects of populations shared across all loci and effects of loci shared across all populations using logistic regression [18]. When the effect of a locus shared across all populations deviates significantly from 0 (*i*.*e*. FDR *q*-value < 0.10), the locus is labeled as an outlier. BayeScan implements the logistic regression model in a Bayesian framework, with model parameters estimated via Markov Chain Monte Carlo (MCMC). Point estimates for parameters are given as the mean of their posterior distributions. We used the default MCMC settings for BayeScan (*i*.*e*. 20 pilot runs each of length of 5,000 steps, a burn-in of 50,000 steps followed by an additional 50,000 steps thinned every 10 steps). Multiple MCMC analyses (*n* = 3) were conducted for four different prior weights on the null model (10:1, 100:1, 1000:1, and 10000:1) to check for convergence. The proportion of outliers for each marker type at each prior weight was compared using a *Z*-test and α = 0.05.

The Population Graph approach begins by partitioning multilocus genetic variation into components of within- and among-population genetic variance using standard methods [64,65]. The among-population genetic variance component is comprised of the set of pairwise multilocus covariances, derived from distances [61], in allele frequencies among all populations. The Population Graph approach uses quantities derived from these covariances (*i*.*e*. pairwise partial correlations; see [61]) to define the minimal set that is needed to explain the among-population component of genetic variation. Specifically, each pairwise partial correlation coefficient is tested statistically as being significantly different from zero. From the set of pairwise partial correlations that were significant, the topology of a Population Graph is defined and the conditional genetic distances (cGDs) are computed as the shortest path between pairs of populations on this topology [65–67]. Values for cGD are estimated conditional on the Population Graph topology (*i*.*e*. as Type III sums of squares are for linear models), so that they highlight edges of the graph that cannot be explained using the remaining edges. Simulation and empirical work have shown that the topology of a Population Graph is sensitive to standard demographic processes, such as gene flow and range expansions [65,66]. This is largely because these processes also create unique patterns in the multilocus covariances among populations.

A separate Population Graph was estimated for each dataset. Differences in the topology of genetic structure between datasets was tested using distance congruence as employed in the *test_congruence* function in the *popgraph* library of R, which represents a Mantel test between a matrix of cGD values derived from each Population Graph. If a significant correlation (i.e., *r* ≠ 0) is detected then the two Population Graphs share more similarities than by chance.

### Environmental association analysis

#### Single-locus environmental association analysis

For comparison, we also conducted a simple, single-locus environmental association analysis. We used square root arcsine transformed allele frequencies within the 29 populations to correlate with the 19 bioclimatic variables. Specifically, for each SNP, each of the top three principal components (PCs) from the principal components analysis (PCA) of the 19 bioclimatic variables were correlated with the residuals of the transformed population allele frequencies. The top three PCs were chosen because they had eigenvalues greater than 1.0 and collectively explained 88.9% of the total variance. Correlation was assessed using Spearman rank correlations, the significance of which was assessed using large-sample approximations as employed in the cor.test function of R. Latitude and longitude and their nonlinear transformations were used to control for spatial autocorrelation and demographic history, as done similarly for the multilocus analyses, with the residuals of the population allele frequencies produced via regressing these quantities on the square root arcsine transformed allele frequencies and taking the residuals. Multiple tests were accounted for using the false discovery rate of [68], with a q-value of 0.05 taken as the significance threshold. All analyses were conducted using R.

#### Multilocus environmental association analyses

We used Redundancy Analysis (RDA) to explore the linear relationship between allele frequencies and climate for each dataset, as well as to partition the overall variance in allele frequencies across loci to effects explained by climate, geography, and their interaction [69]. In this analysis, a matrix of arcsine-square root transformed allele frequencies within populations was the response variable, while the climate and geographical variables for each population were the predictors. For SSRs, the matrix corresponding to the arcsine-square root transformed frequency in each population of each allele at each locus was used as the response. For geographical variables, centered values of latitude and longitude along with their non-linear transformations were used to account for spatial effects [69]. The optimal set of geographical predictors was found for each molecular marker dataset using the *ordistep* function with the default settings in the *vegan* library of R, which is a stepwise model-building algorithm suitable for constrained ordination. Both unconditional and conditional RDA models were fit to each type of molecular data and variance partitioning of the effects of climate, geography, and the interaction of climate and geography was conducted using these results (see [69]). Statistical significance of the model and each axis was tested using a permutation-based (*n* = 9,999 permutations) analysis of variance (ANOVA) following [69]. All analysis was conducted using the *vegan* package in R.

We also tested the relationship of cGD to climate and geography using multiple regression on distance matrices (MRM) [70] as carried out in the *ecodist* library of R. Pairwise Euclidean distances were used to represent climate variation and were calculated using the nine centered and standardized bioclimatic variables. Geography was represented as either distance (km) as calculated using the *rdist* function in the *fields* library in R or as Euclidean distances of the geographical variables used in the RDA. The same models as those used for the RDA were fit using MRM, with the conditional models based on the residuals in the response matrices calculated using a custom R script. Separate analyses were performed for each dataset.

### Simulations to assess the effect of mutation rate differences on climate correlations

We used a coalescent simulation approach as employed in the program *ms* [71] to assess the effect of mutation rate differences between marker types on structure of the Population Graphs and hence their correlations with an associated climate variable. We focused on a 10 population system in which neighbouring populations oriented along a one-dimensional landscape shared migrants at a rate of 2*N*_*e*_*m* = 2.5. All other connections among populations were set to 2*N*_*e*_*m* = 0.0. From each population, 10 diploid individuals were sampled. The global effective population size (*N*_e_) was set to 10,000 for each marker type, with SNP data simulated assuming a mutation rate of 1.0 x 10^−9^ per site/generation and SSR data simulated assuming a mutation rate of 1.0 x 10^−3^ or 1.0 x 10^−5^ per site/generation using the infinite allele model. Simulation of SSR data followed the algorithm of [72]. To test the effect of mutation rate differences on the structure of Population Graphs, we simulated 1,000 multilocus datasets of each marker type with the number of loci in each dataset matching those of each observed dataset (SNPs: 44; SSRs: 12). For each simulated dataset for each marker type, we estimated a Population Graph using the approach described for the empirical data. We tested for congruence between Population Graphs of each marker type for each replicated simulation using the *test_congruence* function in the *popgraph* library of R. The set of Mantel correlation coefficients describing congruence in each case was used as the null distribution against which the observed value was compared. If mutation rate explained the difference in the observed data then we would expect the observed Mantel correlation coefficient to be explained as a likely outcome of simulated null distribution. We discarded simulated Population Graphs containing disjunct groups of populations, which produce matrices of cGD values, upon which the congruence test is based, containing undefined values. This was sensible because the observed Population Graphs did not contain disjunct groups of populations.

## Results

### Genetic diversity

Magnitudes of genetic diversity, and the inbreeding coefficient (*F*_IS_), were different between the marker types (Table 3; S4 and S5 Tables). SSRs had higher heterozygosities (*H*_O_ and *H*_E_), higher *A*_E_ values, and a larger deficit of observed heterozygotes relative to SNPs (i.e. *F*_IS_ > 0). Differences between marker types were significant for averages taken across loci and populations (Table 3; S4 and S5 Tables).

**Table 3:** Summary of overall genetic diversity and inbreeding coefficient for each marker set. Values in parentheses represent the 95% bootstrap confidence intervals for the average across populations (*n* = 10,000 replicates across populations). *A*_E_ = effective number of alleles per locus; *H*_O_ = observed heterozygosity; *H*_E_ = expected heterozygosity; *F*_IS_ = within-population inbreeding coefficient; *F*_ST_ = inter-population genetic differentiation; *G*_ST_’ = inter-population genetic differentiation independent of heterozygosity differences.

Parameter	SSR	SNP
	(loci = 12)	(loci = 44)
*A*_E_	4.93 (4.67 – 5.19)	1.33 (1.31 – 1.36)
*H*_O_	0.67 (0.65 – 0.70)	0.26 (0.24 – 0.28)
*H*_E_	0.74 (0.72 – 0.75)	0.20 (0.18 – 0.21)
*F*_IS_	0.09 (0.05 – 0.12)	-0.17 (-0.20 – -0.13)
*F*_ST_	0.113 (0.079 – 0.139)	0.136 (0.100 – 0.148)
*G*_*ST*_*’*	0.391 (0.079 – 0.139)	0.033 (0.002 – 0.067)

Measures of genetic diversity were correlated across populations between marker types (*H*_E_: *r* = 0.485, *P* = 0.0028; *A*_E_: *r* = 0.546, *P* = 0.0001). Genetic diversity illustrated geographical trends for each marker type, with *H*_E_ and *A*_E_ inversely correlated with latitude (SSRs: *H*_E_, *r* = -0.485, *P* = 0.0044; *A*_E_, *r* = -0.548, *P* = 0.0034; SNPs: *H*_E_: *r* = -0.414, *P* = 0.0066; *A*_E_: *r* = -0.496, *P* = 0.0019) and positively correlated with longitude (SSRs: *H*_E_, *r* = 0.341, *P* = 0.0383; *A*_E_, *r* = 0.469, *P* = 0.0070; SNPs: *H*_E_: *r* = 0.155, *P* = 0.2148; *A*_E_: *r* = 0.117, *P* = 0.2766) (S5 Fig). As such, *H*_E_ and *A*_E_ decreased from south to north and increased from east to west, although the correlations with longitude for SNPs were not statistically significant. Correlations of *F*_IS_ were also observed with latitude (SSRs: *r* = -0.427, *P* = 0.0058; SNPs: *r* = 0.437, *P* = 0.0046) and longitude (SSRs: *r* = -0.404, *P* = 0.0162; SNPs: *r* = -0.013, *P* = 0.4604), although the correlation of *F*_IS_ with longitude was not significantly different from 0 for SNPs. Given that *F*_IS_ was positive on average within populations for SSRs, this translated into *F*_IS_ decreasing towards 0 from south to north for SSRs. Given that *F*_IS_ was negative on average within populations for SNPs, *F*_IS_ increased towards 0 from south to north for SNPs.

### Genetic structure

Allele frequencies across the range of eastern white pine were structured for both marker types (Table 4; S6 and S7 Tables). Multilocus values for *F*_ST_ were significantly greater than 0 for both SSRs (*F*_ST_ = 0.113) and SNPs (*F*_ST_ = 0.136). The 95% bootstrap confidence intervals for each estimate, however, revealed that the magnitude of this statistic did not differ between the marker types (SSRs: 0.079–0.139; SNPs: 0.100–0.148). When differences in heterozygosities were accounted for between marker types, however, SSRs (*G*_ST_’ = 0.391, 95% CI: 0.326–0.458) were significantly more structured than SNPs (*G*_ST_’ = 0.033, 95% CI: 0.002–0.067).

**Table 4:** Summary of the BayeScan results for *F*_ST_ outliers. Values in parentheses are 95% credible intervals. Results are listed for a range of prior weights on the null model.

Locus		*F*_ST_	Locus effect (α)	*q*-value	ESS (α)
**SNP**					
RPSS14_03		0.287			
	10:1	0.266	1.030 (0.504 – 1.582)	0.0005	4184.33
	100:1	0.267	1.053 (0.509 – 1.621)	0.0042	4560.31
	1000:1	0.247	0.892 (0.001 – 1.544)	0.0813	4302.19
	10000:1	0.160	0.225 (0.000 – 1.345)	0.4918	3014.35
RPSS61_05		0.043			
	10:1	0.038	-1.465 (-2.381 – -0.664)	< 0.0001	4011.22
	100:1	0.038	-1.445 (-2.339 – -0.654)	0.0002	4042.03
	1000:1	0.039	-1.445 (-2.325 – -0.595)	0.0074	4546.29
	10000:1	0.055	-1.213 (-2.335 – 0.000)	0.1928	1094.88
**SSR**					
RPS12		0.026			
	10:1	0.024	-2.163 (-2.401 – -1.948)	< 0.0001	406.67
	100:1	0.023	-2.148 (-2.364 – -1.939)	< 0.0001	600.09
	1000:1	0.024	-2.058 (-2.267 – -1.844)	< 0.0001	486.80
	10000:1	0.024	-2.039 (-2.237 – -1.837)	< 0.0001	839.50
RPS20		0.083			
	10:1	0.076	-0.934 (-1.143 – -0.740)	< 0.0001	422.65
	100:1	0.075	-0.918 (-1.107 – -0.729)	< 0.0001	445.42
	1000:1	0.075	-0.835 (-1.014 – -0.661)	< 0.0001	416.77
	10000:1	0.076	-0.813 (-0.989 – -0.650)	< 0.0001	453.21
RPS25		0.082			
	10:1	0.071	-1.003 (-1.235 – -0.786)	< 0.0001	420.07
	100:1	0.072	-0.980 (-1.201 – -0.758)	< 0.0001	576.26
	1000:1	0.072	-0.883 (-1.095 – -0.666)	< 0.0001	627.75
	10000:1	0.072	-0.862 (-1.052 – -0.666)	< 0.0001	367.68
RPS39		0.063			
	10:1	0.067	-1.063 (-1.315 – -0.811)	< 0.0001	436.23
	100:1	0.067	-1.057 (-1.311 – -0.802)	< 0.0001	533.67
	1000:1	0.067	-0.975 (-1.239 – -0.719)	< 0.0001	585.90
	10000:1	0.066	-0.949 (-1.185 – -0.707)	< 0.0001	487.01
RPS50		0.039			
	10:1	0.043	-1.540 (-1.773 – -1.326)	< 0.0001	388.13
	100:1	0.044	-1.514 (-1.729 – -1.298)	< 0.0001	357.42
	1000:1	0.043	-1.443 (-1.665 – -1.233)	< 0.0001	389.85
	10000:1	0.044	-1.403 (-1.614 – -1.189)	< 0.0001	278.99

The BayeScan analysis resulted in two and five of the loci being identified as outliers for SNPs, and SSRs, respectively (Table 4). In the case for SNPs, the only outlier SNP (RPSS14_03), which was consistent with diversifying selection, had an observed value of *F*_ST_ that was approximately two-fold larger than the average, whereas the outlier SNP (RPSS61_05) which was consistent with stabilizing selection had an observed *F*_ST_ that was approximately four-fold smaller than the average. These SNPs, however, only exhibited significant estimates for locus effects when the prior odds of the neutral model were set to 1000:1 or less (Table 4). All five SSR loci identified as outliers had estimates of *F*_ST_ that were approximately two- to five-fold less than the average, which was consistent with stabilizing selection. These five loci were outliers for all values of the prior odds placed on the neutral model. More information about convergence and specific results from the BayeScan analysis are given in S1 Text and S1–S4 Figs. The proportion of outliers at prior odds of 1000:1 or less differed between marker types when considering all outliers (1000:1 odds: *Z* = -2.9697, *P* = 0.0031), but not when considering only outliers consistent with diversifying selection (1000:1 odds: *Z* = 1.2727, *P* = 0.2031).

**Fig 2:**
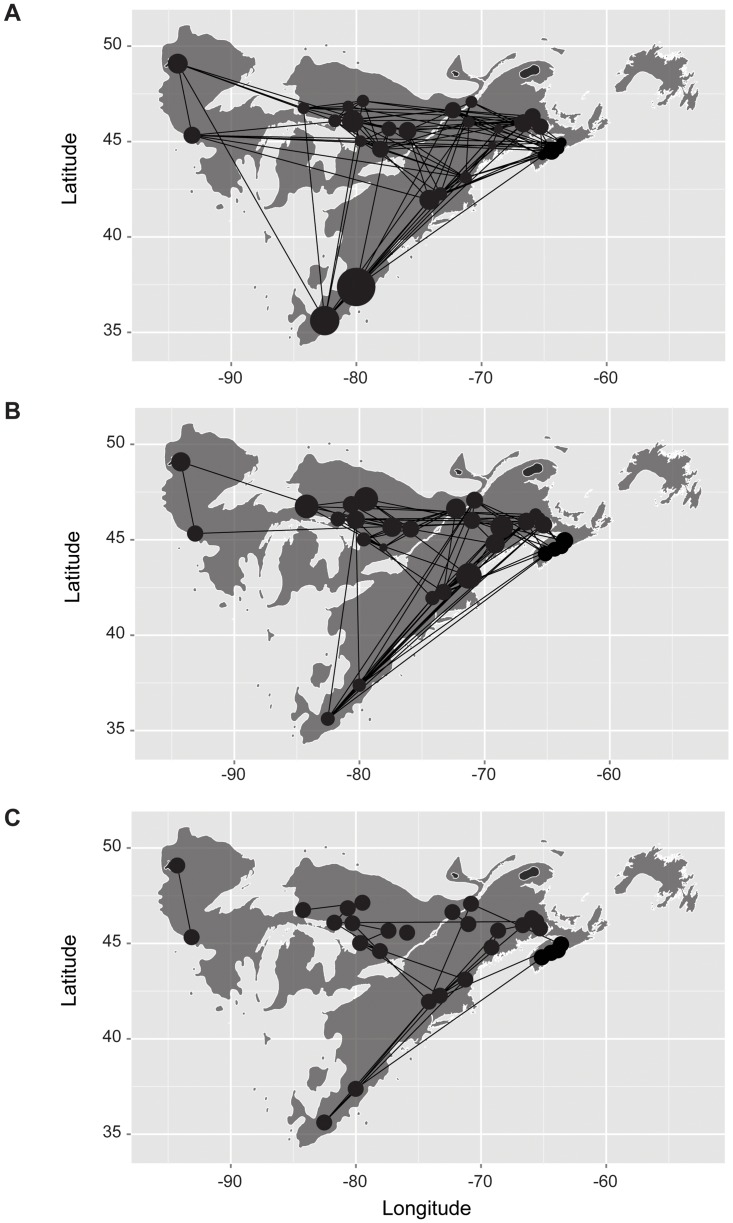
The geographical basis of Population Graphs differs between marker types. Except for panel (C), nodes are scaled proportional to the within-population genetic variance (σ^2^_W_). (A) The Population Graph for the 12 SSR loci. (B) The Population Graph for the 44 SNP loci. (C) The consensus Population Graph for SSRs and SNPs.

Inspection of the Population Graph for each marker type revealed strong geographical trends (Fig 2). Significant covariances among populations were common along the south-to-north axis of the Atlantic seaboard and the east-to-west axis across the top of the Great Lakes for both marker types. Differences between Population Graphs based on each marker type were largely apparent for the cluster of northern populations, which appeared to be more connected for the SSRs than for the SNPs, and for the degree of connectivity between the Atlantic seaboard and the populations north of the Great Lakes, which were more connected for the SSRs. Despite these differences, however, there was a significant correlation between the cGDs from two marker types (*r* = 0.132, *P* = 0.0070), although summary statistics of Population Graph topologies for each marker type revealed many differences (S8 Table). Inspection of the congruence Population Graph revealed that this correlation was driven largely by each of the aforementioned major geographical axes (Fig 2C). The shape of the congruence Population Graph mirrors that for a hypothesized phylogeographical model for eastern white pine involving numerous south-to-north corridors of expansion from a southern refugium after the Last Glacial Maximum [47].

### Environmental (climate) association analysis

#### Single-locus associations

Correlations between transformed allele frequencies within populations and climate were apparent. A total of 21 SNP-climate PC combinations survived multiple test corrections when geography was ignored. When the effect of geography was removed, this dropped to a total of 2 SNP-climate PC combinations. These correlations were between SNP RPSS28_05 and climate PC1 (*ρ* = 0.66256; *p* = 0.00013; *q* = 0.01733) and SNP RPSS62_01 and climate PC3 (*ρ* = 0.62167; *p* = 0.00042; *q* = 0.02789). Climate PC1 explained 53.2% of the variance in the original climate data, whereas climate PC3 explained 9.5% of this variance. The putative homolog of the contig containing RPSS28_05 is an elongation factor like protein, whereas that for the contig containing RPSS62_01 is cinnamate 4-hydroxylase. Inclusion of nonlinear transformations and cross products of geographical variables, however, removed these 2 significant correlations [69]. These patterns remained unchanged if the reduced set of bioclimatic variables (see Materials and Methods) were used.

#### Multilocus associations

**Fig 3:**
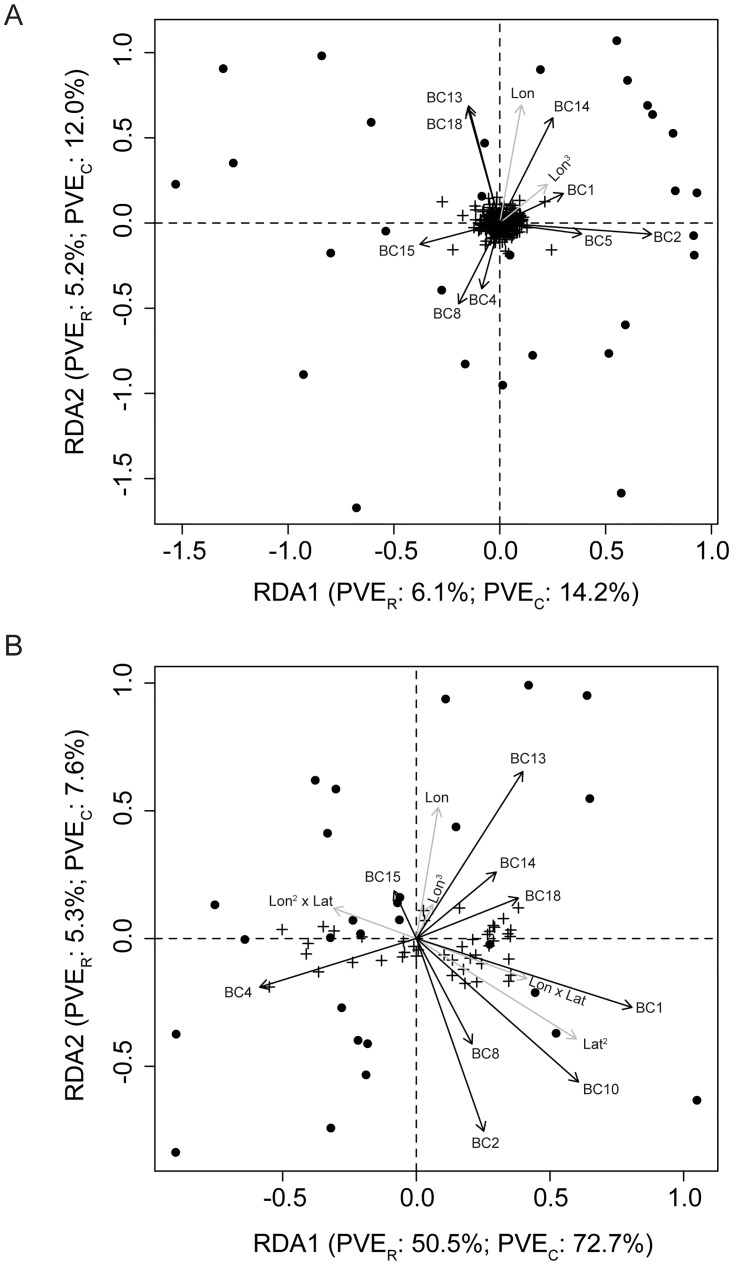
Triplots of RDA solutions illustrate differential effects of climate and geography across marker sets. The specific RDA model is one that includes both climate and geography (i.e. the third model in Table 5). Points represent populations, crosses represent SNPs or SSR alleles, and arrows represent geographical (gray) or climate (black) variables. (A) RDA model with climate and geography for SSRs. Both of the illustrated axes were statistically significant (*P* < 0.05), as were axes 3 and 4. (B) RDA model with climate and geography for SNPs. Both of the illustrated axes were statistically significant (*P* < 0.05). Abbreviations: BC, bioclimatic variable; Lat, latitude; Lon, longitude; PVE_C_, percent constrained variance explained; PVE_R_, percent raw variance explained.

Climate and geography were differentially important in structuring genetic diversity across marker types (Table 5; Fig 3). Geography was more important in structuring genetic diversity for SNPs as opposed to SSRs. This was evident in two ways. First, only two of the nine possible geographical variables were selected for SSRs, while five were selected for SNPs during the model selection of geographical variables using RDA. Second, the RDA model using these variables was not statistically significant for SSRs (Table 5). Climate was also differentially important across marker types, with more of the variance in allele frequencies for SNPs accounted for by climate (SNPs: *R*^2^_adj_ = 0.442; SSRs: *R*^2^_adj_ = 0.040). After accounting for the influence on climate by geographical variables, this effect disappeared, with the pRDA model describing the effects of climate conditional on geography not being statistically significant for SNPs (*F*_9,14_ = 1.386, *P* = 0.153; Table 5). The same pRDA model, however, was statistically significant for SSRs, although the proportion of variance accounted for by this model was small (*R*^2^_adj_ = 0.047; Table 5).

**Table 5:** RDA and pRDA results by molecular marker type reveal differential effects of climate and geography across marker types. Bolded values are those with *P*-values < 0.05.

		SSR			SNP	
Effect	*R*^2^_adj_	*F* (df_1_,df_2_)	*P*	*R*^2^_adj_	*F* (df_1_,df_2_)	*P*
Geography^a^	0.02242	1.0713 (9,19)	0.073	**0.28883**	**2.2635 (9,19)**	**0.012**
Climate	**0.04020**	**1.1303 (9,19)**	**0.003**	**0.44247**	**3.4690 (9,19)**	**0.001**
Geography +Climate	**0.06635**	**1.1809 (11,17)**	**0.001**	**0.38958**	**2.2765 (14,14)**	**0.008**
Geography|Climate	0.02615	1.2661 (2,17)	0.051	-0.05288	0.6708 (5,15)	0.815
Climate|Geography	**0.04697**	**1.1453 (9,17)**	**0.006**	0.09217	1.3859 (9,14)	0.153

^a^Geographical variables were those selected from the original set of nine variables defined following [69] using the *ordistep* function in the *vegan* library of R. For SSRs, these were: longitude and longitude^3^. For SNPs, these were: longitude, latitude^2^, longitude x latitude, longitude^2^ x latitude, and longitude^3^.

**Fig 4:**
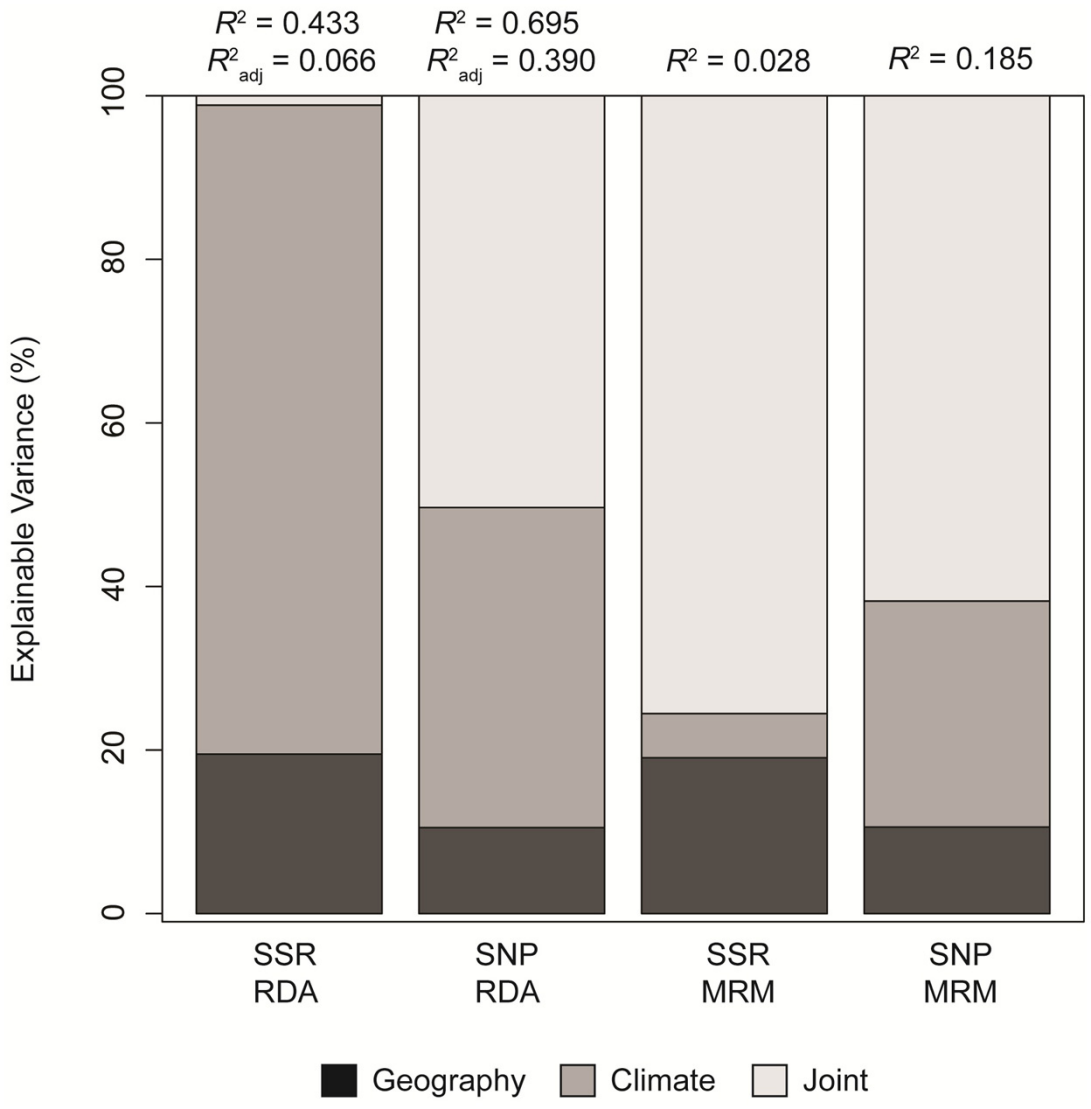
Variance partitioning by type of analysis for each marker set reveals differential effects of climate, geography, and the confounding of climate and geography. For the RDA analyses, partitioning was carried out using the inertia (i.e. variance), so the illustrated percentages are for *R*^2^ and not *R*^2^_adj_. Abbreviations: MRM, multiple regression on distance matrices; RDA, redundancy analysis.

These patterns were also evident in how variance was partitioned among effects due to climate, geography, and the joint action of climate and geography for each marker type (Fig 4). The total amount of variance explained by geography and climate was much larger for SNPs than for SSRs (i.e., *R*^2^_adj_ for SNPs was two to 14-fold higher depending on the model, see Table 5). The partitioning of this explainable variance, moreover, differed strongly between marker types, with climate independent of geography explaining 79.36% of the variance for SSRs, while only 39.14% for SNPs. Considering the confounded effects of climate and geography, however, revealed that 50.34% of the effects observed for SNPs was due to this confounding, while it was only 1.15% for SSRs. This is consistent with the statistically non-significant effect of climate conditional on geography for SNPs (Table 5).

We further explored these patterns using MRM where the response matrix was constructed from values of cGD and the predictor matrices were constructed from Euclidean distances based on climate variables and those geographical variables used in the RDA. In this case, none of the MRM models were statistically significant for SSRs, while four of the five models were statistically significant for SNPs (Table 6). The relative magnitude difference between explainable variance (i.e., *R*^2^) for SSRs versus that for SNPs, however, was similar to that observed for RDA. For example, the *R*^2^ derived from a MRM model of climate predicting cGD for SNPs was approximately 14-fold larger than the same model for SSRs, whereas the same comparison for RDA models resulted in an approximately 11-fold increase in explanatory power.

**Table 6:** MRM analyses using cGD as the response matrix reveal that the SNP data are overly correlated to climate and climate conditional on geography. Differences in *R*^2^ values between marker types are significant using a permutation approach.

			SSR					SNP		
Effect	*R*^2^	*F*	*P*	*β*_*geography*_	*β*_*climate*_	*R*^2^	*F*	*P*	*β*_*geography*_	*β*_*climate*_
Geography^a^	0.018	7.432	0.139	0.0004	---	**0.130**	**60.439**	**0.001**	**0.0004**	---
Climate	0.013	5.131	0.149	---	0.4562	**0.172**	**83.746**	**0.001**	---	**0.7523**
Geography+Climate^a^	0.028	5.765	0.069	0.0024	-0.1540	**0.185**	**45.836**	**0.001**	**0.0010**	**0.4955**
Geography|Climate^a^	0.005	2.174	0.454	0.0002	---	0.020	8.059	0.055	0.0002	---
Climate|Geography^a^	0.002	0.593	0.659	---	0.1545	**0.051**	**21.818**	**0.001**	---	**0.3833**

^a^Geographical distances were based on Euclidean distances derived from those geographical variables used in the RDA (see Table 5).

Partitioning the variance among effects due to climate, geography, and the confounded effects of climate and geography revealed large differences relative to the analysis based on RDA (Fig 4). In this analysis, the confounded effects of climate and geography accounted for 75.54% of the explainable variance for SSRs, while climate independent of geography accounted for only 5.40%. Patterns of explainable variance for SNPs were similar to those based on RDA, with the largest fraction of explainable variance attributable to the confounded effects of climate and geography (61.79%). As with the RDA analyses, however, climate accounted for a significantly larger fraction of the total variance in cGD for SNPs relative to SSRs (Table 6).

**Fig 5:**
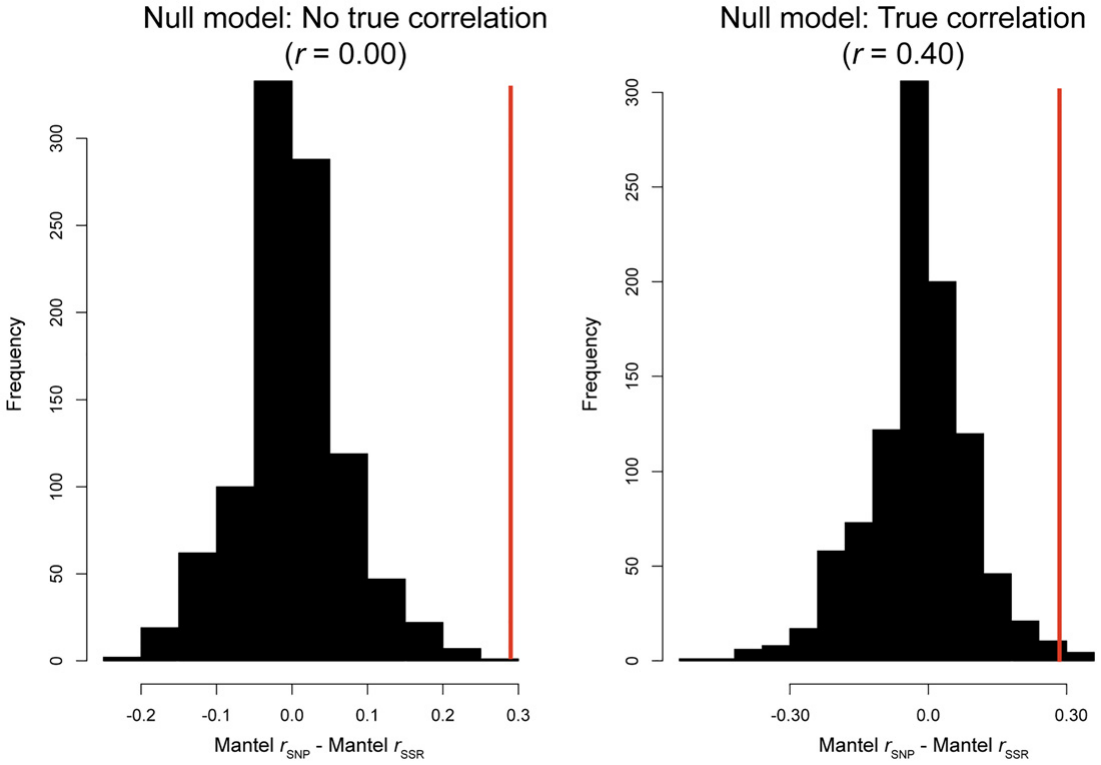
Null distributions for the difference between Mantel correlations for SNPs and SSRs under a simple, one-dimensional stepping-stone model of population structure illustrate the non-effect of mutation rate differences in producing environmental correlations. Vertical red lines mark the observed difference from the main text. (A) A null model of no true environmental correlations. (B). A null model of a true environmental correlation (r = 0.40). Only the differences between marker sets are illustrated, because estimates from each set separately were largely unbiased (bias < 0.15), with the positive biases explainable by use of 19 bioclimate variables in the simulations that were independent, whereas in reality these are highly correlated.

Coalescent simulations confirmed that the observed differences in topologies for the Population Graphs, and hence values of cGD, between marker types was not explainable by differences in mutation rate. The observed correlation between Population Graphs based on each dataset (*r* = 0.132) was not a likely outcome in simulations along a one-dimensional stepping stone model for a difference in mutation rates between marker types of 1.0 x 10^4^ (*P* < 0.001) or 1 x 10^6^ (*P* = 0.001). This value was too small compared to those simulated under a neutral, one-dimensional stepping stone model. Under a null model of no correlation between population structure and climate, both marker sets yielded slightly positive correlations on average (Mantel r: 0.024 to 0.028). The distribution of differences between marker types, however, was centered on zero (Fig 5A). The same form of result was observed when there was a true correlation between population structure and climate. Both marker sets slightly overestimated the true correlation (Mantel r: 0.456 to 0.461), but the expected difference between marker types was again centered on zero (Fig 5B). These two results confirm that mutation rate, at least in the way we modeled it, does not affect climate correlations. In both cases, moreover, the observed difference between marker types exceeded that expected under the null model (Fig 5).

## Discussion

Populations of forest trees offer some of the best examples of local adaptation for plants to their environment [3]. Here we have employed eastern white pine to test the hypothesis that it is the multilocus covariances among populations that differentiate putatively adaptive loci from those reflecting primarily neutral processes [73]. Specifically, we have shown using multivariate methods that a set of 44 SNPs located in candidate genes involved in adaptive responses to climate have cGD values that are overly correlated to climate relative to a reference set of SSRs. Similarities between marker sets, moreover, followed those expected due to models of the Pleistocene phylogeography for eastern white pine [47]. The differences in mutation rates between SSRs and SNPs likely did not affect the topologies of the Population Graphs, and hence values of cGD and their correlations with associated climate variables.

### Genetic diversity and demography

Genetic diversity for SSRs was substantially higher than that for SNPs. This is consistent with the much higher mutation rates for SSRs [74] than those for biallelic SNPs located within putatively functional genes [75]. Observed levels of genetic diversity for eastern white pine were larger than those reported in much earlier studies, especially for the SSRs [41,43,44,46], and are most likely due to the large sample sizes and extensive geographical breadth of our sampling scheme. Regardless of the difference in magnitude, however, patterns of genetic diversity across populations were positively correlated between two marker types. The process driving this correlation is most likely the common demographic history underlying all loci in the genome of eastern white pine [76].

Genetic diversity (*A*_E_ and *H*_E_) decreased from south to north and increased from east to west for both marker types. This geographical pattern is consistent with the postglacial migration of eastern white pine from a southern refugium [32,47]. The consensus Population Graph confirmed that this re-colonization likely involved numerous geographical paths and/or multiple re-colonization events [47]. Local genetic bottlenecks in the founding populations were also likely associated with these re-colonization events, which are evidenced by the south-to-north and east-to-west trends for patterns of genetic diversity. These trends were also apparent for *F*_IS_, which was due to the differing strength of geographical influence on *H*_O_ versus *H*_E_ (see S1 Text File; S5 Fig). As such, populations across the range of eastern white pine are likely not at any form of equilibrium (*e*.*g*. migration-drift-selection equilibrium), although many of the commonly employed methods to identify components of the genetic architecture underlying local adaptation assume some form of equilibrium.

It is well established that the mating system, such as inbreeding, normally affects all loci and alleles in the same way, whereas selection does not affect all loci and alleles. Thus, significant heterogeneity in inbreeding coefficients (*F*_IS_) between loci may reflect the evidence of selection [77], and the extent of differences in *F*_IS_ may reflect the differences in the intensity of selection. In our study, we consistently observed significantly higher *F*_IS_ values for SSR than for SNP markers for the studied populations with most populations showing deficiency of heterozygotes for SSR markers and excess of heterozygotes for SNP markers (S4 and S5 Tables). The SSR markers are considered to be putatively neutral whereas the SNP markers were from candidate genes putatively involved in adaptive response to climatic conditions. As such, the SSR-based *F*_IS_ values may reflect the signatures of mating system and the SNP-SSR *F*_IS_ differences as signatures of natural selection. The differences between the SNP and SSR based *F*_IS_ values showed a geographical trend decreasing from south to north and from east to west. The *F*_IS_ differences showed a significantly negative correlation with latitude (r = -0.547, *p* = 0.0002), and non-significant negative correlation with longitude (r = -0.235, *p* = 0.292). These results may reflect the geographic pattern of differences in the intensity of selection along the climate gradient. However, these inferences need to be confirmed through experimentation.

### Genetic architecture of local adaptation to climate

Eastern white pine established its current range recently, at least in terms of 4*N*_e_ generations [32]. As such, local selection pressures in the northern portion of its range are relatively novel and recent. In this case, it is expected that the first responses to diversifying selection should be the build-up of excessive multilocus covariances among populations and among loci [6–10]. This was confirmed here, as signals of associations with climate were apparent in multilocus (*i*.*e*. multivariate) analyses, but not necessarily in single-locus analyses. Analysis of *F*_ST_ outliers identified only a single marginal case (*i*.*e*. RPSS14_03 was only an outlier for prior odds of 1000:1 or less for the neutral model) consistent with diversifying selection for SNPs. Since climate and genetic variation are structured spatially, *F*_ST_ outliers are a reasonable approximation to single-locus effects, so that these effects are indeed rare within our data. This SNP was located in a putative ABC-transporter (putative homolog of AT3G21250; BLASTx: identity: 72%, coverage: 95%, e-value: 4e-62), which is a member of a gene family that has been implicated in a variety of stress responses in model plants [78]. The point, however, is that *F*_ST_ outliers were not the major signal in our data despite the genes from which SNPs were derived being candidates for functional responses to climate-related factors. Even if they were, there are numerous ways with which to be an outlier, not all of which are consistent with climate-mediated selection pressures [79]. It was thus the correlations between the multilocus covariances among populations, as assessed through the use of multilocus analyses (RDA and MRM with cGD), and climate that were the outliers. As shown for *Drosophila*, this is also consistent with interactions among quantitative trait loci without strong, single-locus effects [27,80], although these were not examined explicitly here. It is likely, therefore, that this type of response will be that to future climate change, as the pace of climate change will be fast relative to the generation time of most forest trees [10,33,81].

Climate and geography were strongly confounded for the RDA and MRM analyses, although the magnitude of this confounding varied by marker type. For the SNP data, the strongest signals of correlation between genetic variation and climate came from the analysis using cGD. Values for cGD are estimated conditional on the Population Graph topology, so that they highlight edges of the graph that cannot be explained using the remaining edges. This is why the set of cGD values can be considered as the shape of genetic variation underlying magnitude-based statistics such as *F*_ST_. As such, these edges often are most sensitive to demographic processes underlying the observed data [61]. In the case of expansions from refugia, these processes are structured spatially, so that it is unsurprising that the correlation structure differed between RDA and MRM based on cGD. In essence, analysis using cGD values highlighted the spatial components of this demographic history, which themselves are confounded with climate gradients most likely affecting fitness differences among trees as they colonized novel environments during expansions out of Pleistocene refugia.

Our results are consistent with theoretical predictions that the loci contributing to local adaptation are remarkable because of correlated and subtle allele frequency differences among populations (i.e. have elevated levels of among population covariance in allele frequencies) and not large, single-locus effects [6–11], yet are at odds with recent empirical work searching for single-locus outliers within and among populations of forest trees (*e*.*g*. [79,82,83], but see [24, 84–86] for examples of multilocus approaches to the study of adaptation in trees). This implies that important and ecologically relevant genetic patterns may be missed due to the focus on single loci. The relative magnitude of how much is missed, however, depends upon a number of biological (*e*.*g*. trait architecture, patterns of linkage disequilibrium) and statistical (*e*.*g*. saturation of the genome, sample sizes) attributes of the study system. Some of this disconnect may be remedied by using genomic prediction methods [87], *cf*. [84,88] to rank genotypes as more or less suited for particular environments and the focus on quantitative genetic prediction from common garden data, *e*.*g*. [89]. Foundational questions about the genetic architecture of locally adapted phenotypes, however, will need to consider both multilocus and single-locus effects [29], although the identity of the specific loci contributing to local adaptation may remain difficult to establish [90].

### Caveats

There are several caveats to our explanations and choices of analysis. First, the observation that climate and geography were more confounded for SNP relative to SSR data could be artifactual. An alternate explanation is that mutation rate differences between these two types of markers confounded the spatial signal in the SSR data (see [91]). Use of a control set of SNPs would have alleviated this concern, but since these data were not available we used simulations to assess the impact of mutation rate variation on patterns of cGD. Coalescent simulations could not account for differences as large as those for the observed Population Graph topologies. For example, assuming a one-dimensional stepping stone model, the average congruence between markers with mutation rate differences on the order of 1.0 x 10^6^ was *r* = 0.864 (95% CI: 0.324–0.991). The observed value for the SNP and SSR data was *r* = 0.132. Although additional models of population structure and demographic processes need to be examined, it is clear that mutation rate difference is not likely the driver of the observed patterns (see also [92] for a similar consideration of null alleles and cGD values). The reason is that the mutation rate in the simulations is random and not spatially autocorrelated. If this assumption of the simulations, however, is violated in eastern white pine then our conclusion that the mutation rate difference between marker types had no effect is incorrect. Future simulations should thus investigate how demography (e.g. bottlenecks in spatially proximal populations) could introduce spatial autocorrelation in the effective mutation rate and confound inferences using cGD.

Second, we assumed that the multilocus analysis we conducted implicitly modeled the among-population component of intergenic covariances [6]. We analyzed several SNPs located at the same gene locus. If the observed multilocus covariances were driven largely by physical linkage among non-causative loci, then we would expect that the levels of intragenic linkage disequilibrium would be larger than the levels of intergenic linkage disequilibrium. The opposite pattern, however, was observed for both *r*^2^ and *D*’ (S6 Fig), so that the largest values of each statistic were observed only among SNPs located in different genes and not among SNPs located within the same gene.

Third, we assumed that our candidate genes do in fact underlie locally adapted traits in eastern white pine. Our focal genes have protein products with functional responses to climate factors for model plants (see Table 2; S2 Table). Protein products from these candidate genes are potentially involved in responses to cold (temperature), drought (precipitation), and seasonal photoperiod (daylight length), as well as underlie growth, development and phenological traits in model plant species (see S2 Table). The south to north gradient in temperature and photoperiod, as well as the east to west gradient in precipitation (Fig 1), are the dominant climate factors to which eastern white pine has had to adapt over its natural range.

### Conclusions and future directions

Forest tree species are clearly adapted to their local environments, a conclusion supported by a wealth of experimental data [3,4,29,88]. We have presented data that illustrate differences in the multilocus covariances among populations between climate-responsive candidate genes and a reference set of SSRs in eastern white pine. Extrapolation of this result to other tree species, considering that their life history is similar to eastern white pine, implies that future work should consider multilocus effects [27], an idea that greatly predates the genomic revolution for non-model species [6,28] and references therein. Future work is also needed to rigorously establish the utility of Population Graphs and other multivariate methods to the study of local adaptation [93], as other data-intensive methods are available for genome-wide inferences, *e*.*g*. [90,94]. The key for use of many of these methods to study signals of selection, including cGD, is the ability to create focal sets of genes with which to perform hypothesis tests. For example, we used candidate genes based on homology with known plant proteins in model species. Other approaches could include loci linked to phenotypes through phenotype-genotype associations or those correlated with environmental variables. Further work, moreover, could focus on the ability to search the space of possible focal sets of loci sampled from a large experimental set (e.g. transcriptome or genome-wide SNPs) without the need of additional information to classify loci as being interesting. Studies within natural populations, especially when combined with the wealth of genetic associations discovered in experimental plantings, highlight forest trees as an unparalleled system with which to study the genetic architecture of locally adapted traits in plants.

## Supporting Information

S1 Fig Trace plots for the log-likelihood of the MCMC runs from BayeScan for the three replicates for a given prior odds (1000:1) of the null model for both SNPs (top panels) and SSRs (bottom panels). Colors denote independent runs (black, red, blue). The green line in each plot gives the average across the chain. Note that the scale on the x-axis is the generation divided by 10, which was the thinning interval.
(PDF)

S2 Fig Effective sample size estimates for the log-likelihood (ln*L*) for all 24 independent runs of the MCMC sampler for each data set. **(A) SNPs. (B) SSRs**. Note that the uncorrected sample size from the posterior distribution was *n* = 5,000. The difference between the ESS pictured 5,000 is the correction due to autocorrelation along the Markov chain.
(PDF)

S3 Fig Posterior distribution of the log-likelihood from a model with prior odds of the null model of 1000:1 for SNPs (A) and SSRs (B). (PDF)

S4 Fig Summaries of pairwise Kolmogorov-Smirnov tests for posterior distributions of the log-likelihood for SNPs (A) and SSRs (B). The lower triangular is pictured in all plots, with the three runs for a prior odds of the null model of 10:1 at the bottom and the three runs for a prior odds of the null model of 10000:1 at the top. The diagonal has been omitted in all plots. Note that the test statistic is illustrated in the left panel and the corresponding *P*-value in the right panel. Only *P*-values for tests involving comparisons between runs with different prior odds for the null model were significant (α = 0.05).
(PDF)

S5 Fig The relationship between geography (latitude and longitude) and two estimates of heterozygosity for SNPs (A, B) and SSRs (C, D). Red color denotes estimates for observed heterozygosity, while black color denotes estimates for expected heterozygosity.
(PDF)

S6 Fig Distributions of linkage disequilibrium statistics for intergenic versus intragenic comparisons. **(A)** *r*^2^. **(B)** |*D*’|.
(PDF)

S1 Table Microsatellite markers for eastern white pine. Shown is the reference sequence for each marker. Primers are listed in Table 1 of Echt *et al*. (1996). The column entitled BAC gives the GenBank accession for the loblolly pine Bacterial Artificial Chromosome (BAC) to which the reference sequence for the SSR was assigned using results from BLASTn.
(DOCX)

S2 Table Expressed sequence tag (EST) loci (RPSS), annotations and the number of SNPs assayed in eastern white pine. EST sequences were obtained from GenBank, the TreeGenes database (Wegrzyn *et al*., 2008), and published articles (TreeSNP, Pavy *et al*., 2008). Numbers after SNP names denote different SNPs derived from the same amplicon. Biological functions of the candidate genes (ESTs) listed are based on the functions reported from functional analysis of homologues in model plant *Arabidopsis* or other plants. Also SNP in the homolog of RPSS96 was reported to be co-located with the QTL for cold hardiness in Douglas-fir (Wheeler *et al*., 2005).
(DOCX)

S3 Table Flanking sequences of targeted SNPs in eastern white pine. The two alleles at each focal SNP are located between parentheses.
(DOCX)

S4 Table Genetic diversity statistics, and fixation index for eastern white populations for the SSRs. (DOCX)

S5 Table Genetic diversity statistics, and fixation index for eastern white pine populations for the SNPs. (DOCX)

S6 Table Hierarchical *F*-statistics by locus for the SSRs ordered from largest to smallest. **Negative values are effectively zero**. The fraction of *F*_*ST*_ due to climate group (CG) was estimated using the variance components obtained from hierfstat (i.e. σ_CG_^2^/(σ_CG_^2^ + σ_POP_^2^)).
(DOCX)

S7 Table Hierarchical *F*-statistics by locus for the SNPs ordered from largest to smallest. **Negative values are effectively zero**. The fraction of *F*_*ST*_ due to climate group (CG) was estimated using the variance components (σ^2^) obtained from hierfstat (i.e. σ_CG_^2^/(σ_CG_^2^ + σ_POP_^2^)).
(DOCX)

S8 Table Attributes for the Population Graphs resulting from each marker set. (DOCX)

S9 Table Individual trees microsatellite genotype data. (PDF)

S10 Table Bioclimatic factors data for the locations of the sampled eastern white pine populations. (PDF)

S1 Text Supplemental text and references. (DOCX)
