# Supplementary material for: Single-Locus versus Multilocus Patterns of Local Adaptation to Climate in Eastern White Pine (Pinus strobus, Pinaceae)
Source: PLoS One. 2016 Jul 7;11(7):e0158691. doi: 10.1371/journal.pone.0158691 (PMC4936701; doi:10.1371/journal.pone.0158691)
Supplement: S1 Fig — Colors denote independent runs (black, red, blue). The green line in each plot gives the average across the chain. Note that the scale on the x-axis is the generation divided by 10, which was the thinning interval. (PDF) [file pone.0158691.s001.pdf]

Supplemental Figures

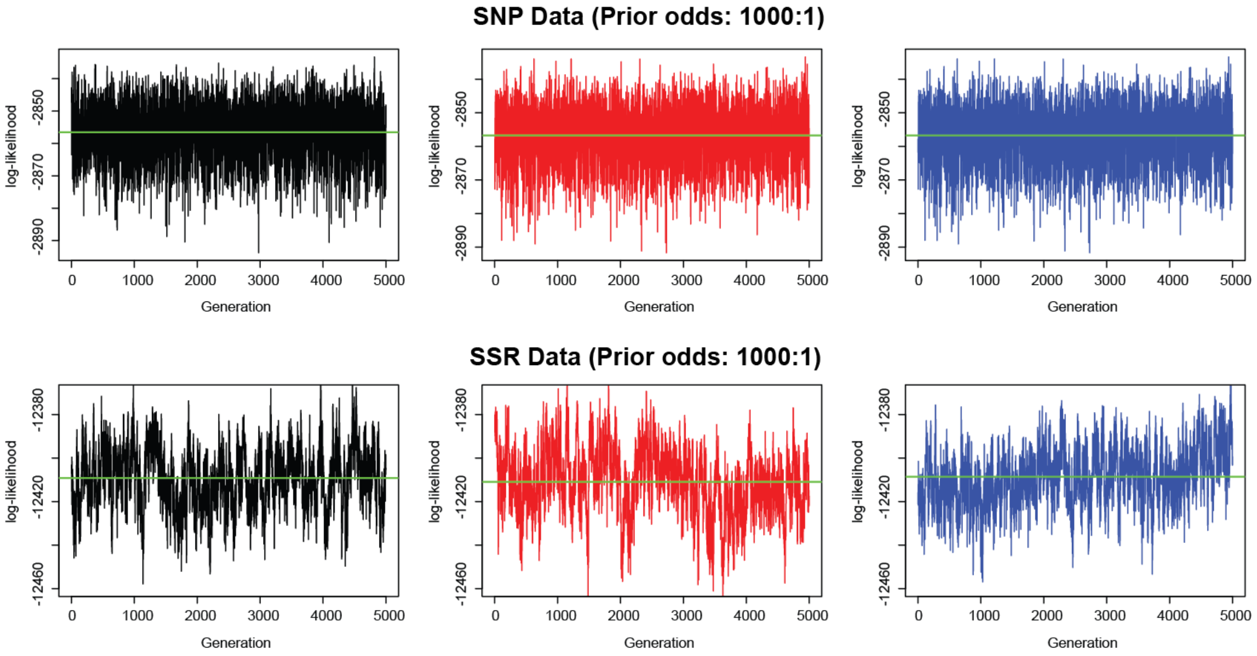

Figure S1. Trace plots for the log-likelihood of the MCMC runs from BayeScan for the three replicates for a given prior odds (1000:1) of the null model for both SNPs (top panels) and SSRs (bottom panels). Colors denote independent runs (black, red, blue). The green line in each plot gives the average across the chain. Note that the scale on the x-axis is the generation divided by 10, which was the thinning interval.
