# Supplementary material for: Single-Locus versus Multilocus Patterns of Local Adaptation to Climate in Eastern White Pine (Pinus strobus, Pinaceae)
Source: PLoS One. 2016 Jul 7;11(7):e0158691. doi: 10.1371/journal.pone.0158691 (PMC4936701; doi:10.1371/journal.pone.0158691)
Supplement: S2 Fig — (A) SNPs. (B) SSRs. Note that the uncorrected sample size from the posterior distribution was n = 5,000. The difference between the ESS pictured 5,000 is the correction due to autocorrelation along the Markov chain. (PDF) [file pone.0158691.s002.pdf]

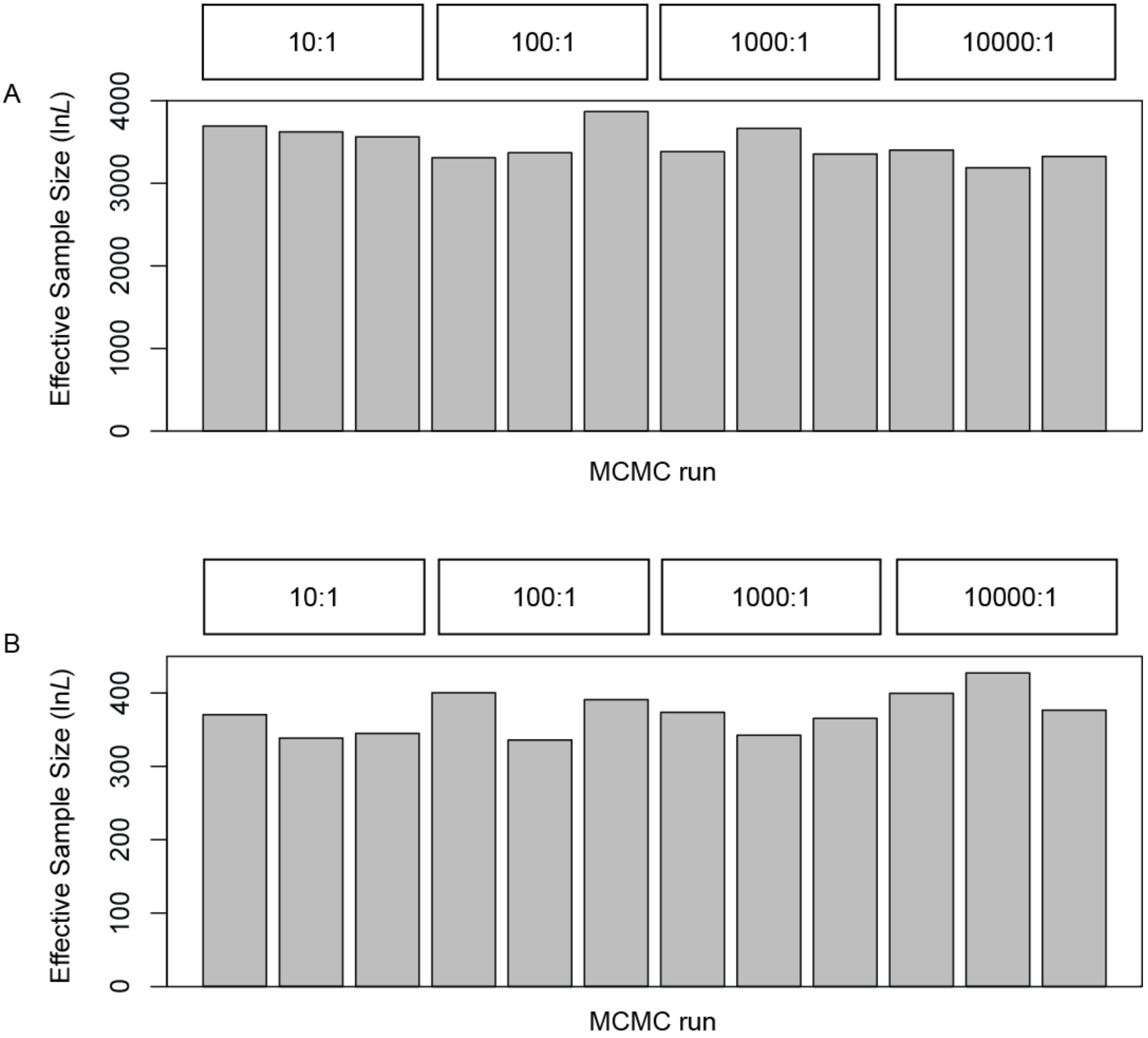

Figure S2. Effective sample size estimates for the log-likelihood (ln*L*) for all 24 independent runs of the MCMC sampler for each data set. (A). SNPs. (B). SSRs. Note that the uncorrected sample size from the posterior distribution was *n* = 5,000. The difference between the ESS pictured 5,000 is the correction due to autocorrelation along the Markov chain.
