# Supplementary material for: Single-Locus versus Multilocus Patterns of Local Adaptation to Climate in Eastern White Pine (Pinus strobus, Pinaceae)
Source: PLoS One. 2016 Jul 7;11(7):e0158691. doi: 10.1371/journal.pone.0158691 (PMC4936701; doi:10.1371/journal.pone.0158691)
Supplement: S3 Fig — (PDF) [file pone.0158691.s003.pdf]

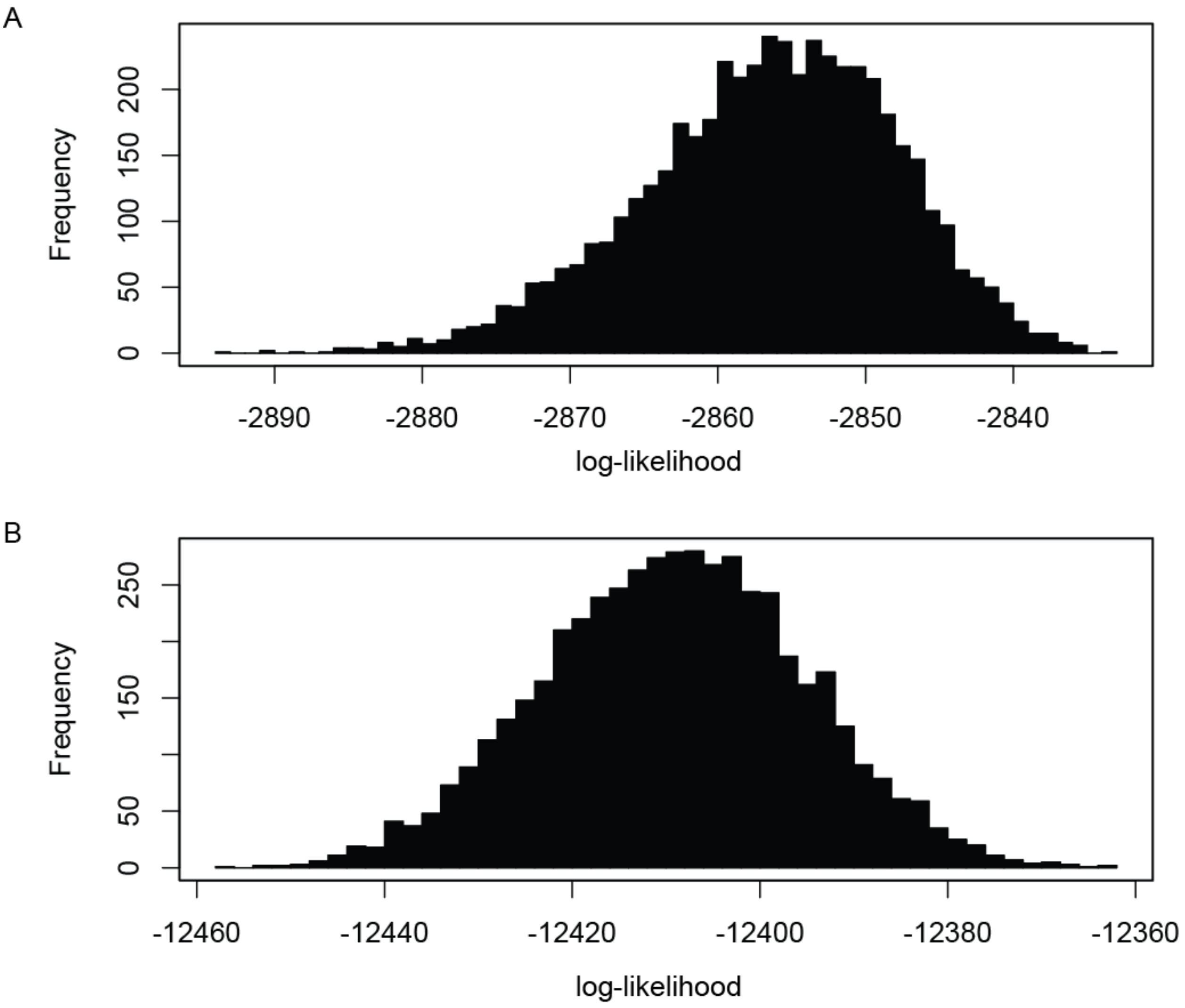

Figure S3. Posterior distribution of the log-likelihood from a model with prior odds of the null model of 1000:1 for SNPs (A) and SSRs (B).
