# Supplementary material for: Single-Locus versus Multilocus Patterns of Local Adaptation to Climate in Eastern White Pine (Pinus strobus, Pinaceae)
Source: PLoS One. 2016 Jul 7;11(7):e0158691. doi: 10.1371/journal.pone.0158691 (PMC4936701; doi:10.1371/journal.pone.0158691)
Supplement: S4 Fig — The lower triangular is pictured in all plots, with the three runs for a prior odds of the null model of 10:1 at the bottom and the three runs for a prior odds of the null model of 10000:1 at the top. The diagonal has been omitted in all plots. Note that the test statistic is illustrated in the left panel and the corresponding P-value in the right panel. Only P-values for tests involving comparisons between runs with different prior odds for the null model were significant (α = 0.05). (PDF) [file pone.0158691.s004.pdf]

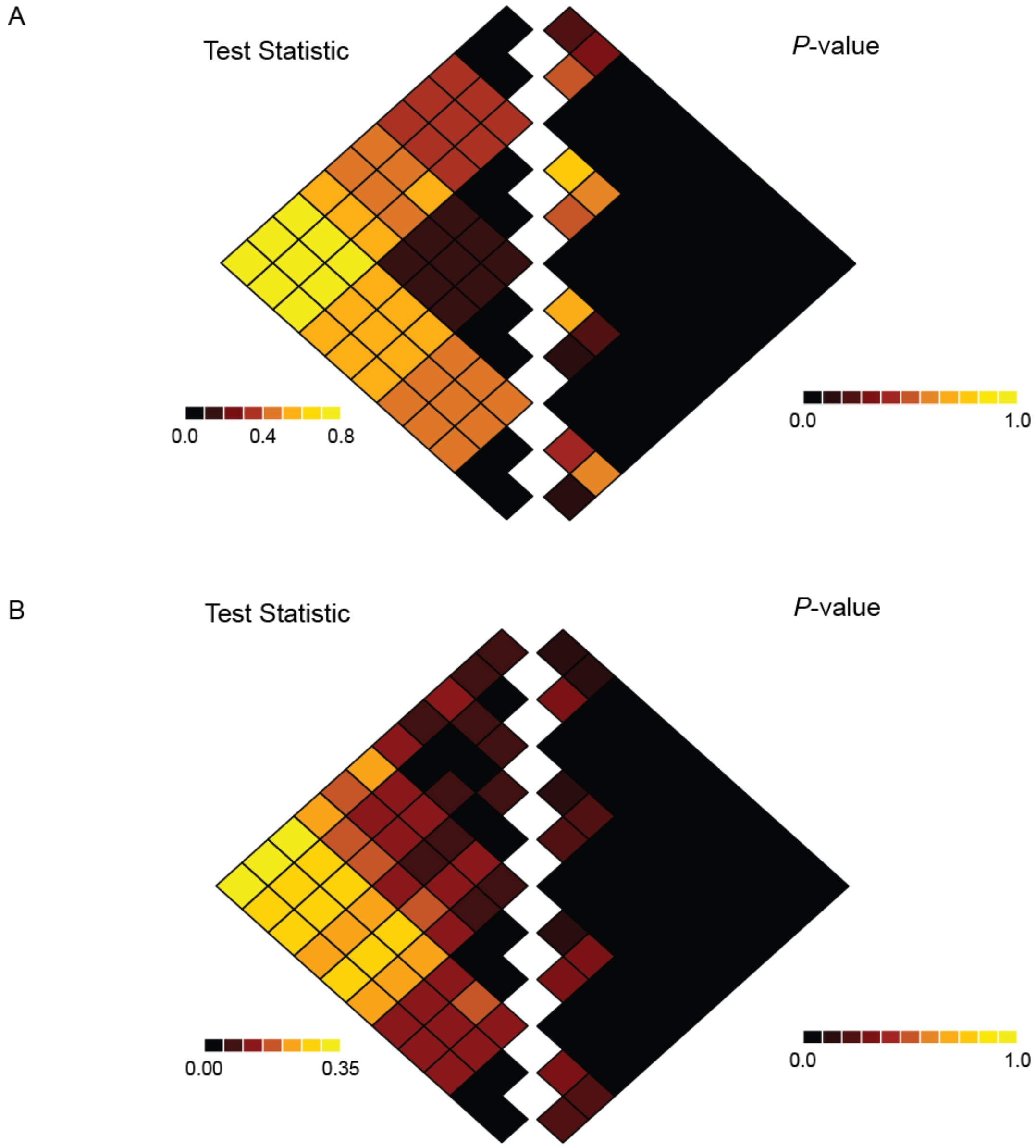

Figure S4. Summaries of pairwise Kolmogorov-Smirnov tests for posterior distributions of the log-likelihood for SNPs (A) and SSRs (B). The lower triangular is pictured in all plots, with the three runs for a prior odds of the null model of 10:1 at the bottom and the three runs for a prior odds of the null model of 10000:1 at the top. The diagonal has been omitted in all plots. Note that the test statistic is illustrated in the left panel and the corresponding *P*-value in the right panel. Only *P*-values for tests involving comparisons between runs with different prior odds for the null model were significant ($\alpha = 0.05$).
