# Supplementary material for: Single-Locus versus Multilocus Patterns of Local Adaptation to Climate in Eastern White Pine (Pinus strobus, Pinaceae)
Source: PLoS One. 2016 Jul 7;11(7):e0158691. doi: 10.1371/journal.pone.0158691 (PMC4936701; doi:10.1371/journal.pone.0158691)
Supplement: S5 Fig — Red color denotes estimates for observed heterozygosity, while black color denotes estimates for expected heterozygosity. (PDF) [file pone.0158691.s005.pdf]

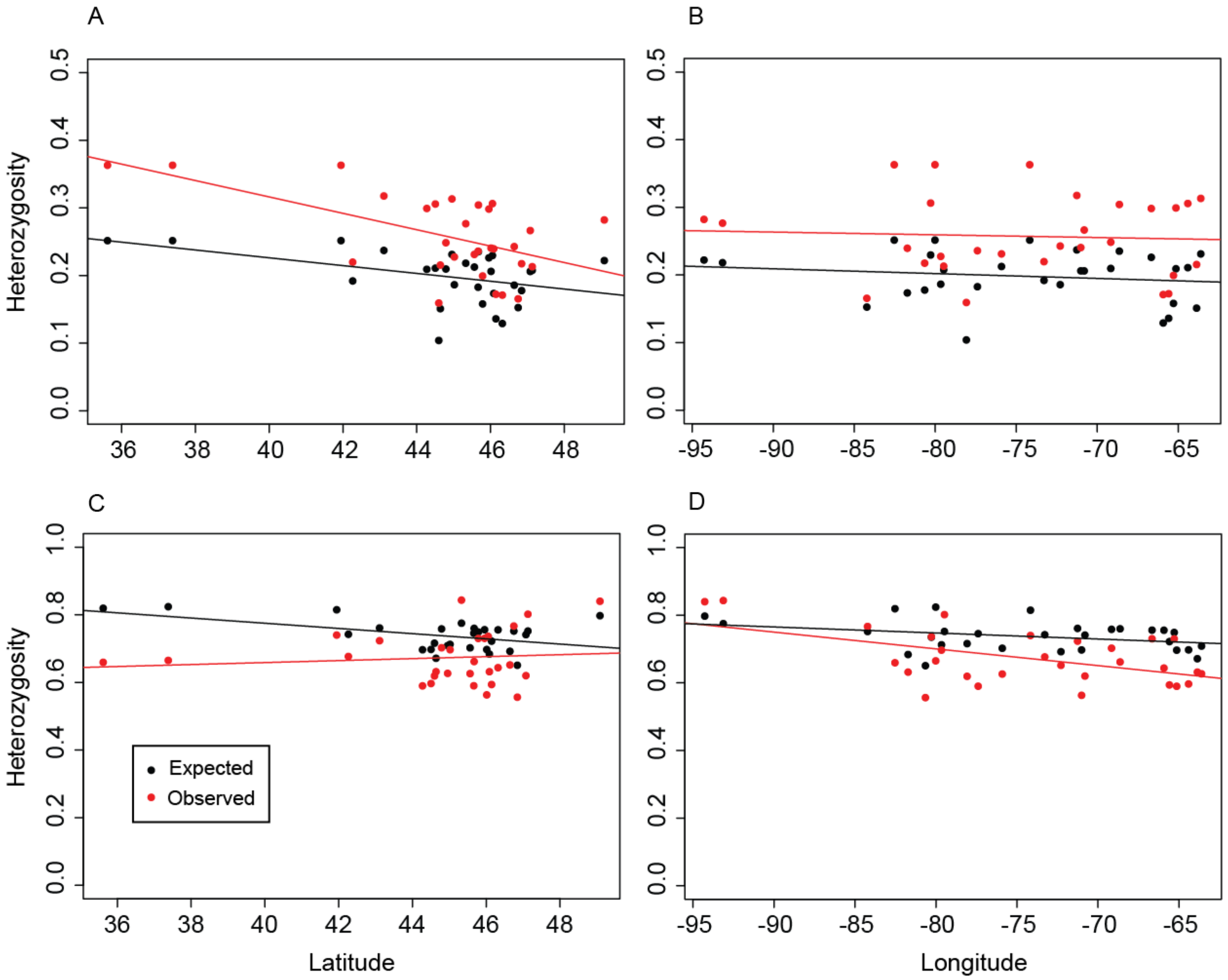

Figure S5. The relationship between geography (latitude and longitude) and two estimates of heterozygosity for SNPs (A, B) and SSRs (C, D). Red color denotes estimates for observed heterozygosity, while black color denotes estimates for expected heterozygosity.
