# Supplementary material for: Single-Locus versus Multilocus Patterns of Local Adaptation to Climate in Eastern White Pine (Pinus strobus, Pinaceae)
Source: PLoS One. 2016 Jul 7;11(7):e0158691. doi: 10.1371/journal.pone.0158691 (PMC4936701; doi:10.1371/journal.pone.0158691)
Supplement: S6 Fig — (A) r2. (B) |D’|. (PDF) [file pone.0158691.s006.pdf]

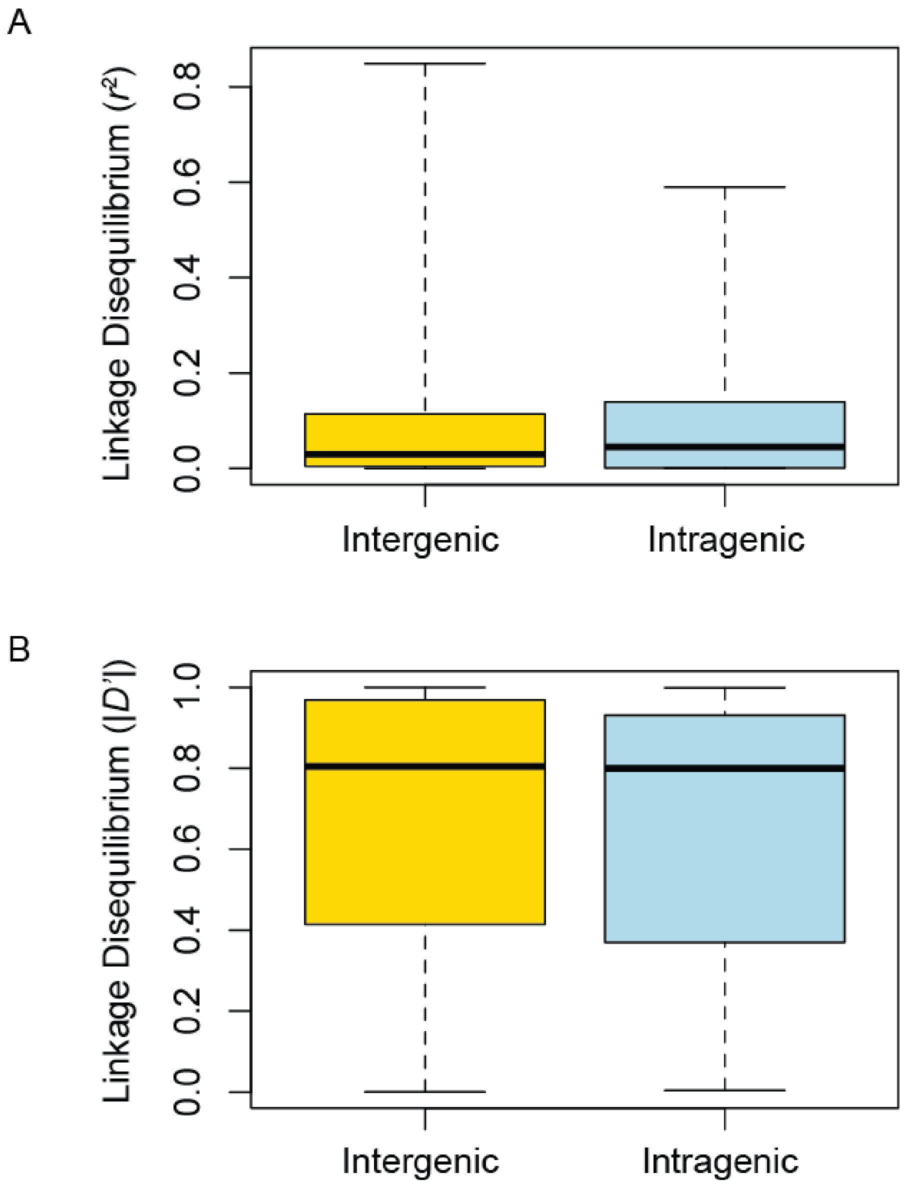

Figure S6. Distributions of linkage disequilibrium statistics for intergenic versus intragenic comparisons. (A). r^2**. (B) |*D'*|.**
