# Supplementary material for: Single-Locus versus Multilocus Patterns of Local Adaptation to Climate in Eastern White Pine (Pinus strobus, Pinaceae)
Source: PLoS One. 2016 Jul 7;11(7):e0158691. doi: 10.1371/journal.pone.0158691 (PMC4936701; doi:10.1371/journal.pone.0158691)
Supplement: S1 Table — Shown is the reference sequence for each marker. Primers are listed in Table 1 of Echt et al. (1996). The column entitled BAC gives the GenBank accession for the loblolly pine Bacterial Artificial Chromosome (BAC) to which the reference sequence for the SSR was assigned using results from BLASTn. (DOCX) [file pone.0158691.s007.docx]

**SUPPORTING INFORMATION**

**Supplemental Tables**

**Table S1. Microsatellite markers for eastern white pine.** Shown is the reference sequence for each marker. Primers are listed in Table 1 of Echt *et al.* (1996)^1^. The column entitled BAC gives the GenBank accession for the loblolly pine Bacterial Artificial Chromosome (BAC) to which the reference sequence for the SSR was assigned using results from BLASTn.

| **SSR ID** | **GenBank** | **Sequence** | **BAC** |
| --- | --- | --- | --- |
| RPS1b | U60239 | GATCAAGTATCTCTTCAAAAGTCTAGTGTGCACATGGGCCCACTATTCAAGATGTCATTTTGTAAAACAAAGTTTATATATATGTGTGTCTGTGTCTGTGTCTGTGTGTGTGTGTGTGTGTGTAGATTTAAAAAGTATATTTTTAGTAATATTTAAAAAACAAATATTTTTGAAAAATAAATATTATAAATTTCATTGGTCAACATTAACTTATACCTTATAACCTCATGTTTCTGCTAACATCATATGAGTCATCTCCACTCATAGAACCAAACATTTTGTTTCCACGAACTGGTTAACGAGTGAGATGGATC | NA |
| RPS2 | U60240 | GATCATGTGTCGATGGTTGTTTATAGTAATTTATGTACATGTGGACCCATTATCCATAAGTGAATCTTGGAAATTGGGTTTCACACAATGACTCTCATATCATTTTTACCCTGGCGAGCTTTTGGATTTTGGTAAATGTGCGAGGGGTGGTAAAAGAGTGGTATTAGGGCTCCCAATTCTAGCACTTGGACTATATGGGTGAAATGTGTTTTTCATACATGTATGTTAGTTGTAAATGTGCTTGATATGTATTCATGGTGTTGGTCATTGTTCCAGTTGTGTCATCATGATAGGTTAATGATGCTGACGTTAGTATGATTTGTATGTTTATTTAATTATGTGTGTGTGTGTGTGTGTGTGTGTGTGTGTATGTTATGTTAATCTAGACTTTAATTGGTGCATACGTGATAGCCTCCACTTCACTTCACTAAGGTACGTGTGATTTGGTTTATATTTAGTTGTCTGACATTGAATGTTTACAAGAAGTAGAGGATC | AC241337 |
| RPS12 | U60242 | ATTTAATATCAATGTGGAGATGGTGATTTAAATATGACCATAAAATAATGTGTAACAAAAGCTACACACATACACACACACACACACACACACACACACACACACATATATATATGGGCGCTCTCTCACACAATGTGTGGGGGACAACAAAGAAAAGAACGGGTGGCGGGAGGTTTCTGGTTAGGTCAGAAGTTGCAGGTCATGAATCTGCAACCCTGTGGTGCTCCAAGGTCTGCCGACTAGCCCCTTGTTAGTCGGGCTTGGAGTGCCTGTTCCCCTGGCGGTTCTACATTGACCCTGAGTGTGCTCGTCATTCCCAGAGTTCTGTGTGGTCGGGAATTTCCACGCTATGTGTGGG | NA |
| RPS20 | U60244 | ATTATCTCACTTCCCCACAGGTTAACACACACACACACACACACACACACACACACATATATATATATATTAAAACTTCCCTACGGTCGATTTACACTTGCATATCACTCATTTTCTGTGTAATACTGAATCCCGCCTATCTTGTTAAATACATTGTAAATTATGTTTAAACTATGTTTAAATATATTTATAAAAAATCCCATAGCATATAATTTCTGGG | NA |
| RPS25 | U60245 | ATTTGCGATAACACATATGGCAGAACACACACACACACACACACACACACACACACACAGATATATATATATATATATGATTTGTGCGATTGTGCGATCGTTCGATAGTGCGACGATCACGCATCGCAACTTTCAATGGAACCTTGGCATGGGCGGAGGGTGATTCTGTAAT | NA |
| RPS34b | U60246 | CCTATGACAACTAACCCATGGGACGACTTACATAGTCGGATAATCCATGCCGGCCCTTGNATGAATTTTTAAAACACTGATTTTTTCTAATCAGTGTGCGCTACATAACCTAGCGCACCAGTGTTCTCTTATCACAGCGCACCAAGCACATTTTGTTATAAACACACACACACACACACACACACACACATATTATTTTATTTATTTTTAATATGTGCACCATATGTAAAATAGGGCAGCGGTGCGCTATTTCATTATAGTGCATTACGCATGT | NA |
| RPS39 | U60247 | CCGGATTCTAGCCAGCTCCAACCAGAATCCTGGCGAAATTGTGGGATGCTTAAATTCTCTCCTGCGGTCAAAATCGTGAAGATTTAAAAAGTGTGTGTTCATTTAGTTTGAGACAACACACACTCACACACACACACACACACACACACAGAGCTATATATATATTATTGGGTCAGCGAGCCATGACCCCCACCGGACCCCACGCCCATGT | NA |
| RPS50 | U60248 | CCACATCTGCTATTTTTTTATAGTGTGTATTTCCAGAAACCTTTTCTAGGTTGTCCCAGAAATCTGTTTTAGAGCATAGATCTCTAGTATTAGAACTTGTGGAGCATTGTTTCAACACACACACACACACACACACACACACACACACAGATATATATATCCATATTTCAGTTGTTGCTACTCTTATAGACAATGCAGTTATAGCATTCTGACATTTCATGTGTTAGCAACATTTCATATTTAGGTGTTTTATTTCATGGGAGG | NA |
| RPS60 | U60249 | ACCAGGGTGATGATCGGGGGTAGGTGTCGACGATAATGGCGGTGAGAACAAGGCTGAATGACAAGAACGCGCAGGATGGTGGAGAAAATACACTGGAAGCTTGTGATAATCAGCGGGAATGCATGCTTCCCACGTAAAATAGTTTGCCTGGCGTGGCAGAATATCACAATGAAAATGAATGAGACACACACACACACACACACACACACACACACACACACATATATATATATATACGGCAGGGGAAATGACACAGACACACGGGAATATGACGCATGGATGTACGAAGGACAGGTGGCGAACTGACGTGGCTATGGAGTCAGCGGGTGGAGGAGAACATGACTTGGGCCGTCGGATCTTGAAACCAAATCAACGGCTCAAAAACACTGAGGCAACGCAATGGTGGG | NA |
| RPS118 | U60252 | AACTCTGTGCCACTCTTGTTCTGCTTGGCGCACTGGTCGAAATTGAGGCTTAAGTATTGGTGGATTGGAGGACTTCACAACGAAACCCATTGTGGTGTGTTTGTGAAATCGTTTAGATTGCTAAACAGAGGTTGGGGGATCAAACCCACCCCCCCTTGTCTTGAAGAAATGGTCGTTACACACACACACACACACACACACACACACACACACACACACACACATTAAGGTTTATGGTCGGAGGTGGGCAGGTCCACCCCCGGTGGTCCACTAGTTTTTGTTACCACCATTTAATATGGTAAGGTGGTTGGGGGGGCGCGGGGTCATGG | NA |
| RPS119 | U60253 | ACTTGTGAGAAGATACTTCCTCCAAGTTACTATTGTCATACATAAAATTAATGTAAGTGTTCTTCTTGGGTTTGATAATCATAACTTTTTTTTGTCAATGTTAACAGTTATACCCATGGTAGAGCACACACACACACACACACACATATATATATGAGATTTTGTTATTAAACTTTTATGTTTAAAAGTGTTTTTTAGAAGACAAGGTTAAT | AC241315 |
| RPS127 | U60255 | ACTTGTAAGAAGATACTTCCTCCAAGTTACTATTGTCATACATAAAATTAATGTAAGTNTTCTTCTTGGGTTTGATAATCATAACTTTTTTTTGTCAATGTTAACAGTTATACCCATGGTAGAGCACACACACACACACACACACATATATATATGAGATTTTGTTATTAAACTTTTATGTTTAAAAGTGTTTTTTAGAAGACAAGGTTAAT | AC241315 |

^1^Echt CS, May-Marquardt P, Hseih M, Zahorchak R. 1996. Characterization of microsatellite markers in eastern white pine. Genome 39: 1102–1108.
