# Supplementary material for: Single-Locus versus Multilocus Patterns of Local Adaptation to Climate in Eastern White Pine (Pinus strobus, Pinaceae)
Source: PLoS One. 2016 Jul 7;11(7):e0158691. doi: 10.1371/journal.pone.0158691 (PMC4936701; doi:10.1371/journal.pone.0158691)
Supplement: S2 Table — EST sequences were obtained from GenBank, the TreeGenes database (Wegrzyn et al., 2008), and published articles (TreeSNP, Pavy et al., 2008). Numbers after SNP names denote different SNPs derived from the same amplicon. Biological functions of the candidate genes (ESTs) listed are based on the functions reported from functional analysis of homologues in model plant Arabidopsis or other plants. Also SNP in the homolog of RPSS96 was reported to be co-located with the QTL for cold hardiness in Douglas-fir (Wheeler et al., 2005). (DOCX) [file pone.0158691.s008.docx]

**Table S2.** **Expressed sequence tag (EST) loci (RPSS), annotations and the number of SNPs assayed in eastern white pine.** EST sequences were obtained from GenBank, the TreeGenes database (Wegrzyn *et al.*, 2008) ^1^, and published articles (TreeSNP, Pavy *et al.*, 2008)^1^. Numbers after SNP names denote different SNPs derived from the same amplicon. Biological functions of the candidate genes (ESTs) listed are based on the functions reported from functional analysis of homologues in model plant *Arabidopsis* or other plants. Also SNP in the homolog of RPSS96 was reported to be co-located with the QTL for cold hardiness in Douglas-fir (Wheeler *et al*., 2005) ^1^.

| **EST ID** | **SNP ID** | **Database ID** | **Annotations** | **Climate-responsive biological gene function** | **References^1^ for biological functions** |
| --- | --- | --- | --- | --- | --- |
| RPSS03 | RPSS03_05 | TreeGenes CL1489Contig1; Genbank XP_002534347 | Chaperonin-60 alpha subunit | Plastid division and organization; protein folding; senescence; growth and development | Suzuki *et al.*, 2009; Peng *et al*., 2011 |
| RPSS04 | RPSS04_02 RPSS04_03 | TreeGenes 0_6116  Genbank NP_001064246 | Ankyrin repeat containing protein | Molecular chaperon; plant cellular metabolism; growth and development; regulation of defense response | Shen *et al.*, 2010 |
| RPSS05 | RPSS05_01  RPSS05_04 RPSS05_05 | TreeGenes CL1148Contig1  Genbank XP_003631692 | Malate dehydrogenase - peroxisomal | Oxidoreduction, carbohydrate metabolic process, chlorophyll biosynthetic process, response to light stimulus, regulation of plant-type hypersensitive response; growth; signal transduction | Pracharoenwattana *et al*., 2007; Cousins *et al.* 2008; |
| RPSS06 | RPSS06_03 | TreeGenes 0_3458  Genbank XP_002530724 | Peroxidase | Response to oxidative stress, and abiotic and biotic stresses | Campa, 1990; Cosio &Dunand, 2008 |
| RPSS08 | RPSS08_01  RPSS08_03 | TreeGenes CL544Contig1 GenBank XP_002282867 | Caffeoyl-CoA 3-O-methyltransferase | Lignin and flavonoid biosynthesis; abiotic and biotic stress responses and defense | Zhong *et al.,* 2000; Vanholme *et al,* 2012. |
| RPSS12 | RPSS12_01 RPSS12_03 | TreeGenes 0_8552  GenBank YP_001661405 | NADH dehydrogenase subunit 7 | Oxidoreductase activities; cellular respiration | Unseld *et al*., 1997 |
| RPSS14 | RPSS14_03 RPSS14_06 | TreeGenes 0_16607  GenBank XP_002527423 | Multidrug resistance associated protein 1 | ABC transmembrane transport; cell membrane integrity; abiotic stress response; oxido-reductase activities | Martinoia *et al.,* 2002; Gaillard *et al.,* 2008; Verrier *et al.,* 2008 |
| RPSS16 | RPSS16_01 RPSS16_03 | TreeGenes 2_4183  GenBank XP_002512354 | Potassium-dependent sodium-calcium exchanger-like protein | Cation and transmembrane transport; cell membrane integrity; plant nutrition; growth and development; signal transduction | Mäser *et al.,* 2001 |
| RPSS19 | RPSS19_02 RPSS19_03 RPSS19_04 RPSS19_06 | TreeGenes UMN_3561  GenBank  YP_002905295 | Photosystem II cp47 chlorophyll apoprotein | Photosynthesis; chlorophyll binding; growth and phenology | Wydrzynski & Satoh, 2005. |
| RPSS28 | RPSS28_04 RPSS28_06 | Genbank XP_002947666 | Elongation factor 2 like protein | Freezing tolerance and cold acclimation; heat tolerance; molecular chaperone | Thomson, 1999; Guo *et al.,* 2002; Fu *et al.*, 2012 |
| RPSS30 | RPSS30_01 RPSS30_02 | GenBank AA701809 | Metallothionein-like protein (MT-like) | Response to osmotic and other abiotic stresses; oxidative damage control; cellular homeostasis; leaf senescence | Robinson *et al.,* 1993; Leszczyszyn *et al*., 2013 |
| RPSS31 | RPSS31_01 RPSS31_02 | TreeGenes CL304Contig1  GenBank XP_003557544 | Oxygen evolving complex 33 kda photosystem II protein | Photosynthesis, cold and other abiotic and biotic stress response; cellular cation homeostasis; morphogenesis | Allahverdiyeva *et al*., 2013; Liu *et al*., 2009 |
| RPSS32 | RPSS32_03 | TreeGenes CL2332Contig1  GenBank XP_003525360 | Calcium-dependent protein kinase | Regulation of stomatal movement, transport, osmotic stress, salt stress, and anion channel activity | Mori *et al.,* 2006 |
| RPSS33 | RPSS33_01 | GenBank DQ399071 | MYB transcription factor | Regulation of development, metabolism and response to abiotic and biotic stresses | Dubos *et al.,* 2010; Ambawat *et al*., 2013 |
| RPSS36 | RPSS36_05 | TreeSNP 16494 | Dehydrin | Drought, cold and freezing stress tolerance | Puhakainen *et al.*, 2004; Hanin *et al*., 2011 |
| RPSS47 | RPSS47_04 | TreeSNP 15945 | Permease | Plastid development; plant growth; mineral nutrition; transport of biochemical, such as auxins, ions and metals; protection from oxidative stress; abiotic stress tolerance | Duy *et al.*, 2011; Delhaize *et al*., 2007; Kohl *et al.,* 2012 |
| RPSS61 | RPSS61_02 RPSS61_03 RPSS61_05 RPSS61_06 | TreeSNP 11399 | Glutathione S-transferase | Response to environment; detoxification; protection from oxidative damage | Marrs, 1996; Edwards *et al.,* 2000 |
| RPSS62 | RPSS62_01  RPSS62_02 | TreeSNP 16305 | Cinnamate 4-hydroxylase | Lignin and flavonoid biosynthesis; abiotic and biotic stress responses and defense | Bell-Lelong *et al.,* 1997; [Vanholme *et al.*, 2012](http://www.ncbi.nlm.nih.gov/pubmed?term=Vanholme%20R%5BAuthor%5D&cauthor=true&cauthor_uid=23012438) |
| RPSS66 | RPSS66_04 | TreeSNP 16643 | Heat shock protein | Abiotic stress response and protection of plants; molecular chaperons | Wang *et al.,* 2004 |
| RPSS71 | RPSS71_02 | GenBank AJ132535 | ADP/ATP translocator or adenine nucleotide translocator (ANT) | Maintenance of mitochondria function and integrity; photosynthesis and respiration; nucleotide transport; growth and development; response to stress; regulation of programmed cell death and plant-type hypersensitive response | Palmieri *et al.,* 2008; Kharenko *et al.,* 2011 |
| RPSS77 | RPSS77_04 | TreeSNP 13020 | MYB transcription factor | Regulation of development, metabolism and response to abiotic and biotic stresses | Dubos *et al.,* 2010; Ambawat *et al*., 2013 |
| RPSS86 | RPSS86_01 RPSS86_02 RPSS86_04  RPSS86_06 | TreeGenes CL598Contig2  GenBank XP_002519950 | Chlorophyll a/b-binding protein type 1 (CABBP1) | Photosynthesis; response to light and its intensity; light harvesting, regulation of stomatal conductance; drought stress response | de Bianchi *et al.,* 2011 |
| RPSS87 | RPSS87_05 | GenBank H75184 | Metallothionein-like protein (MT-like) | Response to osmotic and other abiotic stresses; oxidative damage control; cellular homeostasis; water transport | Robinson *et al.,* 1993; Leszczyszyn *et al*., 2013 |
| RPSS96 | RPSS96_02 | GenBank AL749819 | Thiazolebiosynthetic enzyme (TBE) | Response to cold, DNA damage stimulus and light; starch biosynthetic process | Goulas *et al.,* 2006; Wheeler *et al.,* 2005 |

**^1^References**

Ambawat S, Sharma P, Yadav NR, Radav RC. 2013. MYB transcription factor genes as regulators for plant responses: an overview. [*Physiology and Molecular Biology of Plants*](http://link.springer.com/journal/12298) 19: 307-321.

[Allahverdiyeva Y](http://www.ncbi.nlm.nih.gov/pubmed?term=Allahverdiyeva%20Y%5BAuthor%5D&cauthor=true&cauthor_uid=23647309), [Suorsa M](http://www.ncbi.nlm.nih.gov/pubmed?term=Suorsa%20M%5BAuthor%5D&cauthor=true&cauthor_uid=23647309), [Rossi F](http://www.ncbi.nlm.nih.gov/pubmed?term=Rossi%20F%5BAuthor%5D&cauthor=true&cauthor_uid=23647309), [Pavesi A](http://www.ncbi.nlm.nih.gov/pubmed?term=Pavesi%20A%5BAuthor%5D&cauthor=true&cauthor_uid=23647309), [Kater MM](http://www.ncbi.nlm.nih.gov/pubmed?term=Kater%20MM%5BAuthor%5D&cauthor=true&cauthor_uid=23647309), [Antonacci A](http://www.ncbi.nlm.nih.gov/pubmed?term=Antonacci%20A%5BAuthor%5D&cauthor=true&cauthor_uid=23647309), [Tadini L](http://www.ncbi.nlm.nih.gov/pubmed?term=Tadini%20L%5BAuthor%5D&cauthor=true&cauthor_uid=23647309), [Pribil M](http://www.ncbi.nlm.nih.gov/pubmed?term=Pribil%20M%5BAuthor%5D&cauthor=true&cauthor_uid=23647309), [Schneider A](http://www.ncbi.nlm.nih.gov/pubmed?term=Schneider%20A%5BAuthor%5D&cauthor=true&cauthor_uid=23647309), [Wanner G](http://www.ncbi.nlm.nih.gov/pubmed?term=Wanner%20G%5BAuthor%5D&cauthor=true&cauthor_uid=23647309), [Leister D](http://www.ncbi.nlm.nih.gov/pubmed?term=Leister%20D%5BAuthor%5D&cauthor=true&cauthor_uid=23647309), [Aro EM](http://www.ncbi.nlm.nih.gov/pubmed?term=Aro%20EM%5BAuthor%5D&cauthor=true&cauthor_uid=23647309), [Barbato R](http://www.ncbi.nlm.nih.gov/pubmed?term=Barbato%20R%5BAuthor%5D&cauthor=true&cauthor_uid=23647309), [Pesaresi P](http://www.ncbi.nlm.nih.gov/pubmed?term=Pesaresi%20P%5BAuthor%5D&cauthor=true&cauthor_uid=23647309). 2013. *Arabidopsis* plants lacking PsbQ and PsbR subunits of the oxygen-evolving complex show altered PSII super-complex organization and short-term adaptive mechanisms. [*Plant Journal*](http://www.ncbi.nlm.nih.gov/pubmed/23647309?dopt=Abstract) 75:671-684.

Bell-Lelong DA, Cusumano JC, Meyer K, Chapple C. 1997. Cinnamate-4-hydroxilase expression in *Arabidopsis* (regulation in response to development and the environment). *Plant Physiology* 113:3729-3738.

Campa A. 1990. Biological roles of plant peroxidases: known and potential functions. In Everse J, Everse, KE, Grisham MB (eds), Peroxidases in Chemistry and Biology, CRC Press. Pp 25-50.

Cosio C, Dunand C. 2008. Specific functions of individual class III peroxidase genes. *Journal of Experimental Botany* 60: 391-408.

[Cousins AB](http://www.ncbi.nlm.nih.gov/pubmed?term=Cousins%20AB%5BAuthor%5D&cauthor=true&cauthor_uid=18685043), [Pracharoenwattana I](http://www.ncbi.nlm.nih.gov/pubmed?term=Pracharoenwattana%20I%5BAuthor%5D&cauthor=true&cauthor_uid=18685043), [Zhou W](http://www.ncbi.nlm.nih.gov/pubmed?term=Zhou%20W%5BAuthor%5D&cauthor=true&cauthor_uid=18685043), [Smith SM](http://www.ncbi.nlm.nih.gov/pubmed?term=Smith%20SM%5BAuthor%5D&cauthor=true&cauthor_uid=18685043), [Badger MR](http://www.ncbi.nlm.nih.gov/pubmed?term=Badger%20MR%5BAuthor%5D&cauthor=true&cauthor_uid=18685043). 2008. Peroxisomal malate dehydrogenase is not essential for photorespiration in *Arabidopsis* but its absence causes an increase in the stoichiometry of photorespiratory CO_2_ release. [*Plant Physiology*](http://www.ncbi.nlm.nih.gov/pubmed/18685043) 148:786-795.

[See comment in PubMed Commons below](http://www.ncbi.nlm.nih.gov/pubmed/21803939?dopt=Abstract#comments)[de Bianchi S](http://www.ncbi.nlm.nih.gov/pubmed?term=de%20Bianchi%20S%5BAuthor%5D&cauthor=true&cauthor_uid=21803939), [Betterle N](http://www.ncbi.nlm.nih.gov/pubmed?term=Betterle%20N%5BAuthor%5D&cauthor=true&cauthor_uid=21803939), [Kouril R](http://www.ncbi.nlm.nih.gov/pubmed?term=Kouril%20R%5BAuthor%5D&cauthor=true&cauthor_uid=21803939), [Cazzaniga S](http://www.ncbi.nlm.nih.gov/pubmed?term=Cazzaniga%20S%5BAuthor%5D&cauthor=true&cauthor_uid=21803939), [Boekema E](http://www.ncbi.nlm.nih.gov/pubmed?term=Boekema%20E%5BAuthor%5D&cauthor=true&cauthor_uid=21803939), [Bassi R](http://www.ncbi.nlm.nih.gov/pubmed?term=Bassi%20R%5BAuthor%5D&cauthor=true&cauthor_uid=21803939), [Dall'Osto L](http://www.ncbi.nlm.nih.gov/pubmed?term=Dall'Osto%20L%5BAuthor%5D&cauthor=true&cauthor_uid=21803939). 2011. *Arabidopsis* mutants deleted in the light-harvesting protein Lhcb4 have a disrupted photosystem II macrostructure and are defective in photoprotection. [*Plant Cell*](http://www.ncbi.nlm.nih.gov/pubmed/21803939?dopt=Abstract) 23:2659-2679.

Delhaize E, Gruber BD, Ryan PR. 2007. The role of organic anion permeases in aluminium resistance and mineral nutrition. *FEBS Letters* 581: 2255-2262.

Dubos C, Stracke R, Grotewold E, Weisshaar B, Martin C, Lepiniec L. 2010. Myb transcription factors in *Arabidopsis*. *Trends in Plant Science* 15: 573-581.

Duy D, Stube R, Wanner G, Philippar K. 2011. The chloroplast permease PIC1 regulates plant growth and development by directing homeostasis and transport of iron. *Plant Physiology* 155: 1709-1722.

Edwards R, Dixon DP, Walbot V. 2000. Plant glutathione S-tranferases: enzymes with multiple functions in sickness and death. *Trends in Plant Science* 5:193-198.

Fu J, Momcilovic I, Vara Prasad PV. 2012. Roles of protein synthesis elongation factor EF-Tu in heat tolerance in plants. *Journal of Botany* 2012, doi:10.1155/2012/835836.

[Gaillard S](http://www.ncbi.nlm.nih.gov/pubmed?term=Gaillard%20S%5BAuthor%5D&cauthor=true&cauthor_uid=18307782), [Jacquet H](http://www.ncbi.nlm.nih.gov/pubmed?term=Jacquet%20H%5BAuthor%5D&cauthor=true&cauthor_uid=18307782), [Vavasseur A](http://www.ncbi.nlm.nih.gov/pubmed?term=Vavasseur%20A%5BAuthor%5D&cauthor=true&cauthor_uid=18307782), [Leonhardt N](http://www.ncbi.nlm.nih.gov/pubmed?term=Leonhardt%20N%5BAuthor%5D&cauthor=true&cauthor_uid=18307782), [Forestier C](http://www.ncbi.nlm.nih.gov/pubmed?term=Forestier%20C%5BAuthor%5D&cauthor=true&cauthor_uid=18307782). 2008. AtMRP6/AtABCC6, an ATP-binding cassette transporter gene expressed during early steps of seedling development and up-regulated by cadmium in *Arabidopsis thaliana*. [*BMC Plant Biology*](http://www.ncbi.nlm.nih.gov/pubmed/18307782?dopt=Abstract) 8:22.

[Goulas E](http://www.ncbi.nlm.nih.gov/pubmed?term=Goulas%20E%5BAuthor%5D&cauthor=true&cauthor_uid=16923014), [Schubert M](http://www.ncbi.nlm.nih.gov/pubmed?term=Schubert%20M%5BAuthor%5D&cauthor=true&cauthor_uid=16923014), [Kieselbach T](http://www.ncbi.nlm.nih.gov/pubmed?term=Kieselbach%20T%5BAuthor%5D&cauthor=true&cauthor_uid=16923014), [Kleczkowski LA](http://www.ncbi.nlm.nih.gov/pubmed?term=Kleczkowski%20LA%5BAuthor%5D&cauthor=true&cauthor_uid=16923014), [Gardeström P](http://www.ncbi.nlm.nih.gov/pubmed?term=Gardestr%C3%B6m%20P%5BAuthor%5D&cauthor=true&cauthor_uid=16923014), [Schröder W](http://www.ncbi.nlm.nih.gov/pubmed?term=Schr%C3%B6der%20W%5BAuthor%5D&cauthor=true&cauthor_uid=16923014), [Hurry V](http://www.ncbi.nlm.nih.gov/pubmed?term=Hurry%20V%5BAuthor%5D&cauthor=true&cauthor_uid=16923014). 2006. The chloroplast lumen and stromal proteomes of *Arabidopsis thaliana* show differential sensitivity to short- and long-term exposure to low temperature. [*Plant Journal*](http://www.ncbi.nlm.nih.gov/pubmed/16923014?dopt=Abstract) 47:720-734.

Guo Y, Xiong L, Ishitani M, Zhu J-K. 2002. An *Arabidopsis* mutation in translationelongation factor 2 caused superinduction of CBF/DREB1 transcription factor genes but blocks the induction of their downstream targets under low temperatures. *Proceedings of the National Academy of Sciences, USA* 99: 7786-7791.

[Hanin M](http://www.ncbi.nlm.nih.gov/pubmed?term=Hanin%20M%5BAuthor%5D&cauthor=true&cauthor_uid=21897131), [Brini F](http://www.ncbi.nlm.nih.gov/pubmed?term=Brini%20F%5BAuthor%5D&cauthor=true&cauthor_uid=21897131), [Ebel C](http://www.ncbi.nlm.nih.gov/pubmed?term=Ebel%20C%5BAuthor%5D&cauthor=true&cauthor_uid=21897131), [Toda Y](http://www.ncbi.nlm.nih.gov/pubmed?term=Toda%20Y%5BAuthor%5D&cauthor=true&cauthor_uid=21897131), [Takeda S](http://www.ncbi.nlm.nih.gov/pubmed?term=Takeda%20S%5BAuthor%5D&cauthor=true&cauthor_uid=21897131), [Masmoudi K](http://www.ncbi.nlm.nih.gov/pubmed?term=Masmoudi%20K%5BAuthor%5D&cauthor=true&cauthor_uid=21897131). 2011. Plant dehydrins and stress tolerance: versatile proteins for complex mechanisms. [*Plant Signal Behaviour*](http://www.ncbi.nlm.nih.gov/pubmed/21897131/) 6:1503-1509.

[Kharenko OA](http://www.ncbi.nlm.nih.gov/pubmed?term=Kharenko%20OA%5BAuthor%5D&cauthor=true&cauthor_uid=21473740), [Boyd J](http://www.ncbi.nlm.nih.gov/pubmed?term=Boyd%20J%5BAuthor%5D&cauthor=true&cauthor_uid=21473740), [Nelson KM](http://www.ncbi.nlm.nih.gov/pubmed?term=Nelson%20KM%5BAuthor%5D&cauthor=true&cauthor_uid=21473740), [Abrams SR](http://www.ncbi.nlm.nih.gov/pubmed?term=Abrams%20SR%5BAuthor%5D&cauthor=true&cauthor_uid=21473740), [Loewen MC](http://www.ncbi.nlm.nih.gov/pubmed?term=Loewen%20MC%5BAuthor%5D&cauthor=true&cauthor_uid=21473740). 2011. Identification and characterization of interactions between abscisic acid and mitochondrial adenine nucleotide translocators. [*Biochemistry Journal*](http://www.ncbi.nlm.nih.gov/pubmed/21473740?dopt=Abstract) 437: 117-123.

Kohl S, Hollmann J, Blattner FR, Radchuk V, Andersch F,Steuernagel B, Schmutzer T, Uwe Scholz U, Krupinska K, Weber H, Weschke W. 2012. A putative role for amino acid permeases in sink-source communication of barley tissues uncovered by RNA-seq. *BMC Plant Biology* 12:154.

[Leszczyszyn O](http://www.ncbi.nlm.nih.gov/pubmed?term=Leszczyszyn%20OI%5BAuthor%5D&cauthor=true&cauthor_uid=23694960), [Imam HT](http://www.ncbi.nlm.nih.gov/pubmed?term=Imam%20HT%5BAuthor%5D&cauthor=true&cauthor_uid=23694960), [Blindauer CA](http://www.ncbi.nlm.nih.gov/pubmed?term=Blindauer%20CA%5BAuthor%5D&cauthor=true&cauthor_uid=23694960). 2013. Diversity and distribution of plant metallothioneins: a review of structure, properties and functions. [*Metallomics*](http://www.ncbi.nlm.nih.gov/pubmed/23694960) 5:1146-1169.

[Liu H](http://www.ncbi.nlm.nih.gov/pubmed?term=Liu%20H%5BAuthor%5D&cauthor=true&cauthor_uid=19289096), [Frankel LK](http://www.ncbi.nlm.nih.gov/pubmed?term=Frankel%20LK%5BAuthor%5D&cauthor=true&cauthor_uid=19289096), [Bricker TM](http://www.ncbi.nlm.nih.gov/pubmed?term=Bricker%20TM%5BAuthor%5D&cauthor=true&cauthor_uid=19289096). 2009. Functional complementation of the *Arabidopsis thaliana* psbo1 mutant phenotype with an N-terminally His6-tagged PsbO-1 protein in photosystem II. *Biochimica et Biophysica Acta* 1787:1029-1038.

Marrs KA. 1996. The functions and regulation of glutathione S-transferases in plants. *Annual Review of Plant Physiology and Plant Molecular Biology* 47:127-158.

Martinoia E, Klein M, Geisler M, Bovet L, Forestier C, Kolukisaoglu U, Muller-Rober B, Schulz B. 2002. Multifunctionality of plant ABC transporters – More than just detoxifiers. *Planta* 214: 345–355.

[See comment in PubMed Commons below](http://www.ncbi.nlm.nih.gov/pubmed/11500563?dopt=Abstract#comments)[Mäser P](http://www.ncbi.nlm.nih.gov/pubmed?term=M%C3%A4ser%20P%5BAuthor%5D&cauthor=true&cauthor_uid=11500563), [Thomine S](http://www.ncbi.nlm.nih.gov/pubmed?term=Thomine%20S%5BAuthor%5D&cauthor=true&cauthor_uid=11500563), [Schroeder JI](http://www.ncbi.nlm.nih.gov/pubmed?term=Schroeder%20JI%5BAuthor%5D&cauthor=true&cauthor_uid=11500563), [Ward JM](http://www.ncbi.nlm.nih.gov/pubmed?term=Ward%20JM%5BAuthor%5D&cauthor=true&cauthor_uid=11500563), [Hirschi K](http://www.ncbi.nlm.nih.gov/pubmed?term=Hirschi%20K%5BAuthor%5D&cauthor=true&cauthor_uid=11500563), [Sze H](http://www.ncbi.nlm.nih.gov/pubmed?term=Sze%20H%5BAuthor%5D&cauthor=true&cauthor_uid=11500563), [Talke IN](http://www.ncbi.nlm.nih.gov/pubmed?term=Talke%20IN%5BAuthor%5D&cauthor=true&cauthor_uid=11500563), [Amtmann A](http://www.ncbi.nlm.nih.gov/pubmed?term=Amtmann%20A%5BAuthor%5D&cauthor=true&cauthor_uid=11500563), [Maathuis FJ](http://www.ncbi.nlm.nih.gov/pubmed?term=Maathuis%20FJ%5BAuthor%5D&cauthor=true&cauthor_uid=11500563), [Sanders D](http://www.ncbi.nlm.nih.gov/pubmed?term=Sanders%20D%5BAuthor%5D&cauthor=true&cauthor_uid=11500563), [Harper JF](http://www.ncbi.nlm.nih.gov/pubmed?term=Harper%20JF%5BAuthor%5D&cauthor=true&cauthor_uid=11500563), [Tchieu J](http://www.ncbi.nlm.nih.gov/pubmed?term=Tchieu%20J%5BAuthor%5D&cauthor=true&cauthor_uid=11500563), [Gribskov M](http://www.ncbi.nlm.nih.gov/pubmed?term=Gribskov%20M%5BAuthor%5D&cauthor=true&cauthor_uid=11500563), [Persans MW](http://www.ncbi.nlm.nih.gov/pubmed?term=Persans%20MW%5BAuthor%5D&cauthor=true&cauthor_uid=11500563), [Salt DE](http://www.ncbi.nlm.nih.gov/pubmed?term=Salt%20DE%5BAuthor%5D&cauthor=true&cauthor_uid=11500563), [Kim SA](http://www.ncbi.nlm.nih.gov/pubmed?term=Kim%20SA%5BAuthor%5D&cauthor=true&cauthor_uid=11500563), [Guerinot ML](http://www.ncbi.nlm.nih.gov/pubmed?term=Guerinot%20ML%5BAuthor%5D&cauthor=true&cauthor_uid=11500563). 2001. Phylogenetic relationships within cation transporter families of *Arabidopsis*. [*Plant Physiology*](http://www.ncbi.nlm.nih.gov/pubmed/11500563?dopt=Abstract) 126:1646-1667.

[Mori IC](http://www.ncbi.nlm.nih.gov/pubmed?term=Mori%20IC%5BAuthor%5D&cauthor=true&cauthor_uid=17032064), [Murata Y](http://www.ncbi.nlm.nih.gov/pubmed?term=Murata%20Y%5BAuthor%5D&cauthor=true&cauthor_uid=17032064), [Yang Y](http://www.ncbi.nlm.nih.gov/pubmed?term=Yang%20Y%5BAuthor%5D&cauthor=true&cauthor_uid=17032064), [Munemasa S](http://www.ncbi.nlm.nih.gov/pubmed?term=Munemasa%20S%5BAuthor%5D&cauthor=true&cauthor_uid=17032064), [Wang YF](http://www.ncbi.nlm.nih.gov/pubmed?term=Wang%20YF%5BAuthor%5D&cauthor=true&cauthor_uid=17032064), [Andreoli S](http://www.ncbi.nlm.nih.gov/pubmed?term=Andreoli%20S%5BAuthor%5D&cauthor=true&cauthor_uid=17032064), [Tiriac H](http://www.ncbi.nlm.nih.gov/pubmed?term=Tiriac%20H%5BAuthor%5D&cauthor=true&cauthor_uid=17032064), [Alonso JM](http://www.ncbi.nlm.nih.gov/pubmed?term=Alonso%20JM%5BAuthor%5D&cauthor=true&cauthor_uid=17032064), [Harper JF](http://www.ncbi.nlm.nih.gov/pubmed?term=Harper%20JF%5BAuthor%5D&cauthor=true&cauthor_uid=17032064), [Ecker JR](http://www.ncbi.nlm.nih.gov/pubmed?term=Ecker%20JR%5BAuthor%5D&cauthor=true&cauthor_uid=17032064), [Kwak JM](http://www.ncbi.nlm.nih.gov/pubmed?term=Kwak%20JM%5BAuthor%5D&cauthor=true&cauthor_uid=17032064), [Schroeder JI](http://www.ncbi.nlm.nih.gov/pubmed?term=Schroeder%20JI%5BAuthor%5D&cauthor=true&cauthor_uid=17032064). 2006. [See comment in PubMed Commons below](http://www.ncbi.nlm.nih.gov/pubmed/17032064?dopt=Abstract#comments)CDPKs CPK6 and CPK3 function in ABA regulation of guard cell S-type anion- and Ca(2+)-permeable channels and stomatal closure. [*PLoS Biology*](http://www.ncbi.nlm.nih.gov/pubmed/17032064?dopt=Abstract) 4:e327.

[Palmieri L](http://www.ncbi.nlm.nih.gov/pubmed?term=Palmieri%20L%5BAuthor%5D&cauthor=true&cauthor_uid=18923018), [Santoro A](http://www.ncbi.nlm.nih.gov/pubmed?term=Santoro%20A%5BAuthor%5D&cauthor=true&cauthor_uid=18923018), [Carrari F](http://www.ncbi.nlm.nih.gov/pubmed?term=Carrari%20F%5BAuthor%5D&cauthor=true&cauthor_uid=18923018), [Blanco E](http://www.ncbi.nlm.nih.gov/pubmed?term=Blanco%20E%5BAuthor%5D&cauthor=true&cauthor_uid=18923018), [Nunes-Nesi A](http://www.ncbi.nlm.nih.gov/pubmed?term=Nunes-Nesi%20A%5BAuthor%5D&cauthor=true&cauthor_uid=18923018), [Arrigoni R](http://www.ncbi.nlm.nih.gov/pubmed?term=Arrigoni%20R%5BAuthor%5D&cauthor=true&cauthor_uid=18923018), [Genchi F](http://www.ncbi.nlm.nih.gov/pubmed?term=Genchi%20F%5BAuthor%5D&cauthor=true&cauthor_uid=18923018), [Fernie AR](http://www.ncbi.nlm.nih.gov/pubmed?term=Fernie%20AR%5BAuthor%5D&cauthor=true&cauthor_uid=18923018), [Palmieri F](http://www.ncbi.nlm.nih.gov/pubmed?term=Palmieri%20F%5BAuthor%5D&cauthor=true&cauthor_uid=18923018). 2008. Identification and characterization of ADNT1, a novel mitochondrial adenine nucleotide transporter from Arabidopsis. [*Plant Physiology*](http://www.ncbi.nlm.nih.gov/pubmed/18923018?dopt=Abstract) 148:1797-1808.

Pavy N, Pelgas B, Beauseigle S, Blais S, Gagnon F, Gosselin I, Lamothe M, Isabel N, Bousquet J. 2008. Enhancing genetic mapping of complex genomes through the design of highly-multiplexed SNP arrays: application to the large and unsequenced genomes of white spruce and black spruce. *BMC Genomics* 9: 21.

Peng L, Fukao Y, Myouga F, Motohashi R, Shinozaki K, Shikanai T. 2011. A chaperonin subunit with unique structures is essential for folding of a specific substrate. *PLOS Biology* 9: e1001040.

[See comment in PubMed Commons below](http://www.ncbi.nlm.nih.gov/pubmed/17376163#comments)[Pracharoenwattana](http://www.ncbi.nlm.nih.gov/pubmed?term=Pracharoenwattana%20I%5BAuthor%5D&cauthor=true&cauthor_uid=17376163) I, [Cornah JE](http://www.ncbi.nlm.nih.gov/pubmed?term=Cornah%20JE%5BAuthor%5D&cauthor=true&cauthor_uid=17376163), [Smith SM](http://www.ncbi.nlm.nih.gov/pubmed?term=Smith%20SM%5BAuthor%5D&cauthor=true&cauthor_uid=17376163). 2007. *Arabidopsis* peroxisomal malate dehydrogenase functions in beta-oxidation but not in the glyoxylate cycle. [*Plant Journal*](http://www.ncbi.nlm.nih.gov/pubmed/17376163) *50*:381-90.

[See comment in PubMed Commons below](http://www.ncbi.nlm.nih.gov/pubmed/15356392#comments)[Puhakainen T](http://www.ncbi.nlm.nih.gov/pubmed?term=Puhakainen%20T%5BAuthor%5D&cauthor=true&cauthor_uid=15356392), [Hess MW](http://www.ncbi.nlm.nih.gov/pubmed?term=Hess%20MW%5BAuthor%5D&cauthor=true&cauthor_uid=15356392), [Mäkelä P](http://www.ncbi.nlm.nih.gov/pubmed?term=M%C3%A4kel%C3%A4%20P%5BAuthor%5D&cauthor=true&cauthor_uid=15356392), [Svensson J](http://www.ncbi.nlm.nih.gov/pubmed?term=Svensson%20J%5BAuthor%5D&cauthor=true&cauthor_uid=15356392), [Heino P](http://www.ncbi.nlm.nih.gov/pubmed?term=Heino%20P%5BAuthor%5D&cauthor=true&cauthor_uid=15356392), [Palva ET](http://www.ncbi.nlm.nih.gov/pubmed?term=Palva%20ET%5BAuthor%5D&cauthor=true&cauthor_uid=15356392). 2004. Overexpression of multiple dehydrin genes enhances tolerance to freezing stress in Arabidopsis. *Plant Molecular Biology* 54: 743-753.

Robinson NJ, Tommey AM, Kuske C, Jackson PJ. 1993. Plant metallothioneins. *Biochemistry Journal* 295:1-10.

Shen G, Kuppu S, Venkataramani S, Wang J, Yan J, Qiu X, Zhang H. 2010. Ankyrin repeat-containing protein 2A is an essential molecular chaperone for peroximal membrane-bound ascorbate peroxidase3 in *Arabidopsis*. *The Plant cell* 22: 811-831.

Suzuki K. Nakanishi H, Bower J, Yoder DW, Osteryoung KW, Miyagishima S. 2009. Plastid chaperonin proteins Cpn60 alpha and Cpn60 beta are required for plastid division in *Arabidopsis thaliana*. *BMC Plant Biology* 9: 38.

Thompson MF. 1999. Plant cold acclimation: freezing tolerance genes and regulatory mechanisms. *Annual Review of Plant Physiology and Plant Molecular Biology* 50:571-599.

[See comment in PubMed Commons below](http://www.ncbi.nlm.nih.gov/pubmed/8988169/#comments)[Unseld M](http://www.ncbi.nlm.nih.gov/pubmed?term=Unseld%20M%5BAuthor%5D&cauthor=true&cauthor_uid=8988169), [Marienfeld JR](http://www.ncbi.nlm.nih.gov/pubmed?term=Marienfeld%20JR%5BAuthor%5D&cauthor=true&cauthor_uid=8988169), [Brandt P](http://www.ncbi.nlm.nih.gov/pubmed?term=Brandt%20P%5BAuthor%5D&cauthor=true&cauthor_uid=8988169), [Brennicke A](http://www.ncbi.nlm.nih.gov/pubmed?term=Brennicke%20A%5BAuthor%5D&cauthor=true&cauthor_uid=8988169). 1997. The mitochondrial genome of *Arabidopsis thaliana* contains 57 genes in 366,924 nucleotides. [*Nature Genetics*](http://www.ncbi.nlm.nih.gov/pubmed/8988169/) 15:57-61.

[See comment in PubMed Commons below](http://www.ncbi.nlm.nih.gov/pubmed/18299247?dopt=Abstract#comments)[Verrier PJ](http://www.ncbi.nlm.nih.gov/pubmed?term=Verrier%20PJ%5BAuthor%5D&cauthor=true&cauthor_uid=18299247), [Bird D](http://www.ncbi.nlm.nih.gov/pubmed?term=Bird%20D%5BAuthor%5D&cauthor=true&cauthor_uid=18299247), [Burla B](http://www.ncbi.nlm.nih.gov/pubmed?term=Burla%20B%5BAuthor%5D&cauthor=true&cauthor_uid=18299247), [Dassa E](http://www.ncbi.nlm.nih.gov/pubmed?term=Dassa%20E%5BAuthor%5D&cauthor=true&cauthor_uid=18299247), [Forestier C](http://www.ncbi.nlm.nih.gov/pubmed?term=Forestier%20C%5BAuthor%5D&cauthor=true&cauthor_uid=18299247), [Geisler M](http://www.ncbi.nlm.nih.gov/pubmed?term=Geisler%20M%5BAuthor%5D&cauthor=true&cauthor_uid=18299247), [Klein M](http://www.ncbi.nlm.nih.gov/pubmed?term=Klein%20M%5BAuthor%5D&cauthor=true&cauthor_uid=18299247), [Kolukisaoglu U](http://www.ncbi.nlm.nih.gov/pubmed?term=Kolukisaoglu%20U%5BAuthor%5D&cauthor=true&cauthor_uid=18299247), [Lee Y](http://www.ncbi.nlm.nih.gov/pubmed?term=Lee%20Y%5BAuthor%5D&cauthor=true&cauthor_uid=18299247), [Martinoia E](http://www.ncbi.nlm.nih.gov/pubmed?term=Martinoia%20E%5BAuthor%5D&cauthor=true&cauthor_uid=18299247), [Murphy A](http://www.ncbi.nlm.nih.gov/pubmed?term=Murphy%20A%5BAuthor%5D&cauthor=true&cauthor_uid=18299247), [Rea PA](http://www.ncbi.nlm.nih.gov/pubmed?term=Rea%20PA%5BAuthor%5D&cauthor=true&cauthor_uid=18299247), [Samuels L](http://www.ncbi.nlm.nih.gov/pubmed?term=Samuels%20L%5BAuthor%5D&cauthor=true&cauthor_uid=18299247), [Schulz B](http://www.ncbi.nlm.nih.gov/pubmed?term=Schulz%20B%5BAuthor%5D&cauthor=true&cauthor_uid=18299247), [Spalding EJ](http://www.ncbi.nlm.nih.gov/pubmed?term=Spalding%20EJ%5BAuthor%5D&cauthor=true&cauthor_uid=18299247), [Yazaki K](http://www.ncbi.nlm.nih.gov/pubmed?term=Yazaki%20K%5BAuthor%5D&cauthor=true&cauthor_uid=18299247), [Theodoulou FL](http://www.ncbi.nlm.nih.gov/pubmed?term=Theodoulou%20FL%5BAuthor%5D&cauthor=true&cauthor_uid=18299247).2008. Plant ABC proteins--a unified nomenclature and updated inventory. [*Trends in Plant Science*](http://www.ncbi.nlm.nih.gov/pubmed/18299247?dopt=Abstract) 13:151-159.

[See comment in PubMed Commons below](http://www.ncbi.nlm.nih.gov/pubmed/23012438?dopt=Abstract#comments)[Vanholme R](http://www.ncbi.nlm.nih.gov/pubmed?term=Vanholme%20R%5BAuthor%5D&cauthor=true&cauthor_uid=23012438), [Storme V](http://www.ncbi.nlm.nih.gov/pubmed?term=Storme%20V%5BAuthor%5D&cauthor=true&cauthor_uid=23012438), [Vanholme B](http://www.ncbi.nlm.nih.gov/pubmed?term=Vanholme%20B%5BAuthor%5D&cauthor=true&cauthor_uid=23012438), [Sundin L](http://www.ncbi.nlm.nih.gov/pubmed?term=Sundin%20L%5BAuthor%5D&cauthor=true&cauthor_uid=23012438), [Christensen JH](http://www.ncbi.nlm.nih.gov/pubmed?term=Christensen%20JH%5BAuthor%5D&cauthor=true&cauthor_uid=23012438), [Goeminne G](http://www.ncbi.nlm.nih.gov/pubmed?term=Goeminne%20G%5BAuthor%5D&cauthor=true&cauthor_uid=23012438), [Halpin C](http://www.ncbi.nlm.nih.gov/pubmed?term=Halpin%20C%5BAuthor%5D&cauthor=true&cauthor_uid=23012438), [Rohde A](http://www.ncbi.nlm.nih.gov/pubmed?term=Rohde%20A%5BAuthor%5D&cauthor=true&cauthor_uid=23012438), [Morreel K](http://www.ncbi.nlm.nih.gov/pubmed?term=Morreel%20K%5BAuthor%5D&cauthor=true&cauthor_uid=23012438), [Boerjan W](http://www.ncbi.nlm.nih.gov/pubmed?term=Boerjan%20W%5BAuthor%5D&cauthor=true&cauthor_uid=23012438). 2012. A systems biology view of responses to lignin biosynthesis perturbations in *Arabidopsis*. [*Plant Cell*](http://www.ncbi.nlm.nih.gov/pubmed/23012438?dopt=Abstract) 24:3506-3529.

Wang W, Vinocur B, Shoseyov O, Altman A. 2004. Role of plant heat-shock proteins and molecular chaperones in the abiotic stress response. *Trends in Plant Science* 9: 1360-1385.

Wegrzyn JL, Lee JM, Tearse BR, Neale DB. 2008. TreeGenes: A forest tree database. *International Journal of Plant Genomics* 2008: 412875.

Wheeler NC,Jermstad KD, Krutovsky K, Aitken SN, Howe GT, Krakowski J, Neale DB. 2005. Mapping of quantitative trait loci controlling adaptive traits in costal Douglas fir. IV. Cold-hardiness QTL verification and candidate gene mapping. *Molecular Breeding* 15: 145-156.

Wydrzynski TJ, Satoh K (eds). 2005. Advances in Photosynthesis and respiration, Vol 22 Photosystem II. Springer.

[See comment in PubMed Commons below](http://www.ncbi.nlm.nih.gov/pubmed/11027707#comments)[Zhong R](http://www.ncbi.nlm.nih.gov/pubmed?term=Zhong%20R%5BAuthor%5D&cauthor=true&cauthor_uid=11027707), [Morrison WH III](http://www.ncbi.nlm.nih.gov/pubmed?term=Morrison%20WH%203rd%5BAuthor%5D&cauthor=true&cauthor_uid=11027707), [Himmelsbach DS](http://www.ncbi.nlm.nih.gov/pubmed?term=Himmelsbach%20DS%5BAuthor%5D&cauthor=true&cauthor_uid=11027707), [Poole FL II](http://www.ncbi.nlm.nih.gov/pubmed?term=Poole%20FL%202nd%5BAuthor%5D&cauthor=true&cauthor_uid=11027707), [Ye ZH](http://www.ncbi.nlm.nih.gov/pubmed?term=Ye%20ZH%5BAuthor%5D&cauthor=true&cauthor_uid=11027707). 2000. Essential role of caffeoyl coenzyme A O-methyltransferase in lignin biosynthesis in woody poplar plants. [*Plant Physiology*](http://www.ncbi.nlm.nih.gov/pubmed/11027707) 124:563-578.
