# Supplementary material for: Single-Locus versus Multilocus Patterns of Local Adaptation to Climate in Eastern White Pine (Pinus strobus, Pinaceae)
Source: PLoS One. 2016 Jul 7;11(7):e0158691. doi: 10.1371/journal.pone.0158691 (PMC4936701; doi:10.1371/journal.pone.0158691)
Supplement: S3 Table — The two alleles at each focal SNP are located between parentheses. (DOCX) [file pone.0158691.s009.docx]

**Table S3.** **Flanking sequences of targeted SNPs in eastern white pine**. The two alleles at each focal SNP are located between parentheses.

| **SNP** | **Flanking sequence** |
| --- | --- |
| RPSS03_05 | TCAGGGGAGTACCACATGGGAAACCCGGACGCTGGAGTACTGAAATGATGTCAGCTACCC**[T/G]**GTGAAATGAATGGTCAGTATGTTGTCTCCATTCCTTCCTCGAGTCTTTTAGAACGTCGTG |
| RPSS04_02 | GGTGTTTGCATGGGTTTGCCTTGGTATCGCGTTCATACCGTCGTATTGAATGATCCTGGC**[C/G]**GATTAATTTCTGTACATATAATGCATACAGCTCTAGTTGCTGGTTGGGCCGGTTCAATGA |
| RPSS04_03 | CGTGCGTTTTACAACGTCGTGGAGTACATAGGGACTCGGGATAGACTCCATGAATGCAAT**[A/G]**ATGACTCAACTTGGATGCTCTAAACAAGGGTGACTCGATGGTGCTGCCACACATCAAAGG |
| RPSS05_01 | GATTCCGGCAGGGATGATCACGAGATCCATTCCCACCAACG**[T/C]**TGCTTCCAGCTGCCTCTTTGCCCACAAATCCACGCACACTAGAAAACACA |
| RPSS05_04 | CCACAAATCCACGCACACTAAAAAACACAAAAATCGTTCCGATTAAGACCTAAAATGAA**[A/G]**CAAATAAAAAAGCCCCATTTTTCAAAATTGTACATACAACGGTGGTGGTGTCCATATGGC |
| RPSS05_05 | ATGAAACAAATAAGAAAGCCCCATTTTTCAAAATTGTACATACAACGGTGGTGGTGTCC**[A/T]**TATGGCTGATATCTGCAGTGACGCCGAGGGTATTGACAACATCATAGAGGTGAAGGAATG |
| RPSS06_03 | ATCGCCAATCACCTTCTCTCGTCGTTTTACAACGTCGTGACAAAACTTGCGAGTTCGATA**[A/T]**CGTCATAGGCAATCACCTTCTCTCGTCTTTTTAGAACGACATGATTTTCCAACACAAATA |
| RPSS08_01 | ACCGTCGTATTGAATGATCCTGGCCGATTAATTTCTGTACATATAATGCATACAGCTCTA**[T/G]**TTGCTGGTTGGGCCGGTTCAATGACTCTGTATGAATTAACAGTTTTTGATCCATCCGACC |
| RPSS08_03 | GATTAATTTCTGTACATATAATGCAGACAGCTCTAGTTGCTGGTTGGGCCGGTTCAATGAA**[A/T]**CTGTATGAATTAACAGTTTTTGATCCATCCGACCCTGTTCTTGATCCAATGTGGAGACAA |
| RPSS12_01 | GCTAATTTCATAAATTGAACCTTATCTCAAATTGATCCGTATTCCCATCTTGCACCAC**[C/A]**AGAGATATCCCTTTATCTCCTCCCTATTGAAATAACATTCCCTTCAGATAAGTTAAGTAT |
| RPSS12_03 | CTTTATCTCCTCCCTATTGAAATAACATTCCCTTCAGATAAGTTAAGTATGGAGAAATTA**[A/T]**GAGAAAAAAAAAAACAAAAATTCCATTGTATTTACCCTTTACAAAAGAAAAAGGGGAAGA |
| RPSS14_03 | CATTTCTCCCAGTTTGAATGGTTAGTGTGTCTATGGCCATACCTTCAAGGTTTCTAACTG**[C/T]**TATTCTACCTTCACTTGGCCAAGAGACAGGAAGTCTGCTGCTTTCTATAATTGCCGGAGG |
| RPSS14_06 | AATAAAGTCGGAGAGATTGCATTGCCATTGCACCAAAAATACTTGACAAGATGTAAGTGA**[A/G]**AGTGCATAAGAAAGAGCAAGACCCGCAAATGTCGTTTTACAAACGTCGTGAGATCTCATC |
| RPSS16_01 | GGGTTGCTTAGCGGCACTTGGTGTCATATTAAAGTTGTCACCAGCTTTATTGGGTCTGAC**[A/G]**GTATTGGCTTGGGGAAATTCCATTGGGGATCTAGTTGCAGATGTTGCAGTTGCAAAAGCT |
| RPSS16_03 | TGGTTTTCTGTTTTTGAGCTTACTTGGCTCATTATTTGTGATAACATGGTCAAGATTTCA**[C/G]**GTTCCTCGATTCTGGGGATTTTTCCTTATTGGTCGTTTTACAACGTCGTG |
| RPSS19_02 | AGTTACATAGTGTCTACTTTTTCCGATAAAGGGGTGTTTGCATGGGTTTGCCTTGGTATC**[T/G]**CGTTCATACCGTCGTATTGAATGATCCTGGCCGATTAATTTCTGTACATATAATGCATAC |
| RPSS19_03 | CATATAATGCATACAGCTCTAGTTGCTGGTTGGGCCGGTTCAATGACTCTGTATGAATTA**[A/G]**CAGTTTTTGATCCATCCGACCCTGTTCTTGATCCAATGTGGAGACAAGGTATGTTCGTTA |
| RPSS19_04 | TGACTCTGTATGAATTAGCAGTTTTTGATCCATCCGACCCTGTTCTTGATCCAATGTGGA**[T/G]**ACAAGGTATGTTCGTTATACCCTTTATGACTCGTTTGGGAATAAAGGGTCGTTTTACAAC |
| RPSS19_06 | ACATATAATGCATACAGCTCTAGTTGCTGGTTGGGCCGGTTCAATGACTCTGTATGAAT**[T/G]**AGCAGTTTTTGATCCATCCGACCCTGTTCTTGATCCAATGTGGAGACAAGGTATGTTCGT |
| RPSS28_04 | GATTGCTTGTCTTTGTGTTTGATACCCATCAAGAATAGTCAGCAGTGTGGTGGTTGTGAA**[T/C]**TGTGTGTGAAGATAGATTTATTAATGATGCAATTGATGCTGTTACTGTTGTTCCGTTTCA |
| RPSS28_06 | TAAGGTTAGACAAACAGAGTTGGAATGATTGCTTGTCTTTGTGTTTGATACCCATCAAGA**[A/G]**TAGTCAGCAGTGTGGTGGTTGTGAACTGTGTGTGAAGATAGATTTATTAATGATGCAATT |
| RPSS30_01 | CTTTACCGTGAAGGCGGAGGGCACGAACAAGAACCTTCCACCGGACTTCCAGAAGACGAA**[C/G]**CTCATGACACGGCTGACCTACACGCTGGACGAGATCCGCCCGCTGGAAGTCTCTTCGGAT |
| RPSS30_02 | GCTGGAAGTCTCTTCGGATGGCAAGCTCATGTTTGAAGAGAAGGATGACATCGACTACGC**[C/G]**GCGGTCACTGTTCATCTGCCCAAAGGTGAGCGCCTGCCCTTCCTGTTCACCGTCAGTCGT |
| RPSS31_01 | AGGCGGAGGGCACGAACAAGAACCTTCCACCGGACTTCCAGAAGACGAAGCTCATGACAC**[T/G]**GCTGACCTACACACTGGACGAGATCGGCCCGCTGGAAGTCTCTTCGGATGGCAAGCTCAT |
| RPSS31_02 | AACAAGAACCTTCCACCGGACTTCCAGAAGACGAAGCTCATGACACGGCTGACCTACACA**[T/C]**TGGACGAGATCGGCCCGCTGGAAGTCTCTTCGGATGGCAAGCTCATGTTTGAGGAGAAGG |
| RPSS32_03 | GTTGGTCCTGATGCTAGACCATCCATGGGTGAGAGAAGATGGTGAGGCACCAGACAAACC**[A/T]**CTTGACAGTGCTGTATTAGTTAGGATGAAGCAATTCAGGGCAATGAACAAGCTTAAAAAA |
| RPSS33_01 | TGCACGATACGCCAAACATTCTGTTCGCAATTTCAAAAGTCGTTTTACAACGTCGTGACA**[A/G]**TGGAAGAGATCTTATGGGTGAAAGAAAAACTGGCGGAAGCCAAACAGCAACTACGTAACG |
| RPSS36_05 | TATCTGCCTGCAAGCGTGGCCTGGCGGCAGAAAGAGCAGTTCTCCGATGGCGTCGGTTAC**[A/T]**GTTGGATCGGAGGAGATTTGGTCGTTTTACAACGTCGTGATAATATGAACACACTAAGAT |
| RPSS47_04 | CTGATATGACAGATGAACCCTGTACATGAGCTTACACTGAGTTGATTGAACGGAACCTTA**[T/C]**AATAAATGAGGTATTGGTAATGTGACTTTCAAATTATCTTTGGCAGAGATGAATTTTGCA |
| RPSS61_02 | GAGTTGACAGTCTCATCATATCAATGTCAGAGACCGGAATTTTGCTGAATTGTTGTTTCA**[A/T]**AACTTCATTTGTGTCAGACTGAACATTCAATCTTTCTTTGTACTCTTGCATTTCCTTTTT |
| RPSS61_03 | TGTCAGAGACCGGAATTTTGCTGAATTGTTGTTTCAAAACTTCATTTGTGTCAGACTGAA**[A/C]**ATTCAATCTTTCTTTGTACTCTTGCATTTCCTTTTTGTACCTTTCCTTGTCCTTCAATCC |
| RPSS61_05 | AATCCAAAATCTTGATAAACCTGCAATCATAAGGCAAAATCCTCAGCTATGTCCTGGAGC**[T/C]**CGCTAATGCATGAAACTATGTCCTATGTGCCCGCTACATGCAGGTTGTCATAGTACAAAA |
| RPSS61_06 | TACATGCAGGTTGTCATAGTACAAAAGAATTTTGTCTAAGCTTCATTTGAAAGAATCCTA**[T/G]**AGACACCATATGCACATCAGACCAATATCTTACCCCTCTTTCCTCTTCACTGAGTTTATT |
| RPSS62_01 | CTCTGAGGAACGGCCTGAGAACAGGAATGAAGTCACCATAGTTATACTCGAAGCTCTGAG**[A/C]**CAGGCGGCTCCTCTCGCCATTGAGTGCCTTGAGGCGGAGGAAGAGCGGGTCGTCCTCGCT |
| RPSS62_02 | TTGAGTGCCTTGAGGCGGAGGAAGAGCGGGTCGTCCTCGCTCTCGAACCTCCTGTCAAAC**[A/T]**TCATCCTGTACATGATATTATACATCACAAGCTGCAGGCGCCTCCTGATCACAATCCCCG |
| RPSS66_04 | GTACTCTTCCGTACGGACGATCCTTGCTCTCTCACCGCAGATGGGGTGGCACATTCATCA**[A/G]**ATGGATGTGAAGACTGCGTTCCTCATTGGAGTTATTGAGGAAGAGGTGTACATAGAGCAG |
| RPSS71_02 | TTTGGCTCTGGTGGAGGCTAATATGGATTTGATGATGGTGATAAATATTTAAGACTGTTT**[C/G]**AACACTAATTTTTAGGATTTTTGAGCCCTGACTCTGGCTCATTTTTTTGAATAATGAGTT |
| RPSS77_04 | GAAAGAATCAAATGAATTTGCAGTATCAGGCTTCTGCAAAAGAACAGTCTTTATAGCCTT**[A/G]**CAAAATCTCTTCCACTGTCCTCCCAGTACCAATATAATTGGCAATTACTTCCCACCTTTG |
| RPSS86_01 | CTTATTAGAGCTTGAGAGAGCGTGCAGAAGAAAAGAATTTGAGAAGATTCAAGCACACCA**[A/G]**CTTGAAACACTGGAGGTAATTATAGCAAGAGCCTTCCAACAGAAAGAGCTGGGGATCCAA |
| RPSS86_02 | ACTGGAGGTAATTATAGCAAGAGCCTTCCAACAGAAAGAGCTGGGGATCCAACCAGGCAG**[A/C]**AAATGGGATGGCAACACAATCCAGAAAGACTCCAAGAAAAGAGGTCGGAAAACAGATCTG |
| RPSS86_04 | CCAGAAAGACTCCAAGAAAAGAGGTCGGAAAACAGATCTGGAGAGAACAATCACAGTGGG**[A/C]**AAAATCCTGGTTGATTCTGGAAGATATGCTAAATTGACAAAGTACTATAAGCCCCTCCCA |
| RPSS 86_06 | TCCTGGTTGATTCTGGAAGATATGCTAAATTGACAAAGTACTATAAGCCCCTCCCAAACA**[A/G]**TGAATCATGAAGCTCACATCATGGAATACTAGAGGACTCAACTGTCCCGGGAAACACAGG |
| RPSS87_05 | GTACCACCCACTCCCTGACTGACAATTACGTTAGCATCTGCGGATCATGCTAAAATCGCG**[A/G]**CCTGACAATTATTCACTCAAGGCACGATCATGGCAAAAACAGCAGCAGCACTGCATATCC |
| RPSS96_02 | ATGTTATCACGGGAGACACACGGCGGGTGCTAACGTCCGTCGTGAAGAGGGAAACAACCC**[A/G]**GACCGCCAGCTAAGGTCCCAAAGTCATGGTTAAGTGGGAAACGATGTGGGAAGGCCCAGAC |
