# Supplementary material for: Single-Locus versus Multilocus Patterns of Local Adaptation to Climate in Eastern White Pine (Pinus strobus, Pinaceae)
Source: PLoS One. 2016 Jul 7;11(7):e0158691. doi: 10.1371/journal.pone.0158691 (PMC4936701; doi:10.1371/journal.pone.0158691)
Supplement: S4 Table — (DOCX) [file pone.0158691.s010.docx]

**Table S4.** **Genetic diversity statistics, and fixation index for eastern white populations for the SSRs.**

| **Population ID** | ***A_E_*** | ***H_E_*** | ***H_O_*** | ***F_IS_*** |
| --- | --- | --- | --- | --- |
| NBCI | 4.425 | 0.722 | 0.594 | 0.165 |
| NBCR | 5.045 | 0.755 | 0.644 | 0.134 |
| NBOP | 5.464 | 0.756 | 0.730 | 0.025 |
| NBPH | 4.544 | 0.749 | 0.730 | 0.012 |
| MASB | 5.177 | 0.742 | 0.677 | 0.101 |
| MEBP | 5.381 | 0.760 | 0.662 | 0.124 |
| MEEB | 4.981 | 0.758 | 0.702 | 0.071 |
| NHDF | 4.958 | 0.761 | 0.723 | 0.050 |
| NYCM | 6.259 | 0.815 | 0.740 | 0.098 |
| NSDL | 4.094 | 0.698 | 0.597 | 0.151 |
| NSRL | 4.288 | 0.697 | 0.590 | 0.163 |
| NSMB | 3.948 | 0.671 | 0.632 | 0.059 |
| NSUM | 3.967 | 0.709 | 0.627 | 0.087 |
| MNBL | 5.188 | 0.775 | 0.843 | -0.095 |
| ONCL | 5.877 | 0.797 | 0.840 | -0.056 |
| ONFR | 5.411 | 0.735 | 0.736 | 0.014 |
| ONGR | 4.763 | 0.752 | 0.767 | -0.023 |
| ONHF | 4.685 | 0.716 | 0.619 | 0.158 |
| ONML | 5.206 | 0.712 | 0.697 | 0.062 |
| ONMF | 4.527 | 0.684 | 0.632 | 0.075 |
| ONRC | 4.231 | 0.745 | 0.590 | 0.212 |
| ONTO | 5.137 | 0.752 | 0.802 | -0.070 |
| ONWL | 4.678 | 0.651 | 0.556 | 0.142 |
| PQCT | 4.356 | 0.741 | 0.620 | 0.147 |
| PQLP | 4.049 | 0.702 | 0.626 | 0.104 |
| PQSR | 4.173 | 0.697 | 0.563 | 0.209 |
| PQSS | 4.884 | 0.692 | 0.652 | 0.068 |
| VASB | 6.574 | 0.824 | 0.665 | 0.195 |
| NCAV | 6.605 | 0.819 | 0.659 | 0.196 |
