# Supplementary material for: Single-Locus versus Multilocus Patterns of Local Adaptation to Climate in Eastern White Pine (Pinus strobus, Pinaceae)
Source: PLoS One. 2016 Jul 7;11(7):e0158691. doi: 10.1371/journal.pone.0158691 (PMC4936701; doi:10.1371/journal.pone.0158691)
Supplement: S5 Table — (DOCX) [file pone.0158691.s011.docx]

**Table S5. Genetic diversity statistics, and fixation index for eastern white pine populations for the SNPs.**

| **Population ID** | ***A_E_*** | ***H_E_*** | ***H_O_*** | ***F_IS_*** |
| --- | --- | --- | --- | --- |
| NBCI | 1.233 | 0.136 | 0.172 | -0.117 |
| NBCR | 1.222 | 0.129 | 0.171 | -0.135 |
| NBOP | 1.393 | 0.226 | 0.298 | -0.249 |
| NBPH | 1.258 | 0.158 | 0.199 | -0.117 |
| MASB | 1.316 | 0.192 | 0.220 | -0.049 |
| MEBP | 1.395 | 0.235 | 0.304 | -0.153 |
| MEEB | 1.336 | 0.210 | 0.248 | -0.065 |
| NHDF | 1.397 | 0.237 | 0.318 | -0.139 |
| NYCM | 1.448 | 0.252 | 0.363 | -0.303 |
| NSDL | 1.365 | 0.211 | 0.306 | -0.274 |
| NSRL | 1.362 | 0.209 | 0.299 | -0.245 |
| NSMB | 1.257 | 0.151 | 0.215 | -0.293 |
| NSUM | 1.394 | 0.231 | 0.313 | -0.149 |
| MNBL | 1.359 | 0.218 | 0.277 | -0.185 |
| ONCL | 1.366 | 0.222 | 0.282 | -0.191 |
| ONFR | 1.392 | 0.229 | 0.306 | -0.185 |
| ONGR | 1.248 | 0.153 | 0.165 | -0.038 |
| ONHF | 1.175 | 0.104 | 0.159 | -0.238 |
| ONML | 1.301 | 0.186 | 0.227 | -0.124 |
| ONMF | 1.294 | 0.173 | 0.239 | -0.271 |
| ONRC | 1.303 | 0.183 | 0.236 | -0.187 |
| ONTO | 1.335 | 0.208 | 0.213 | 0.047 |
| ONWL | 1.291 | 0.178 | 0.217 | -0.133 |
| PQCT | 1.346 | 0.206 | 0.266 | -0.186 |
| PQLP | 1.342 | 0.212 | 0.231 | -0.043 |
| PQSR | 1.332 | 0.206 | 0.240 | 0.002 |
| PQSS | 1.308 | 0.186 | 0.243 | -0.213 |
| VASB | 1.448 | 0.252 | 0.363 | -0.303 |
| NCAV | 1.448 | 0.252 | 0.363 | -0.303 |
