# Supplementary material for: Single-Locus versus Multilocus Patterns of Local Adaptation to Climate in Eastern White Pine (Pinus strobus, Pinaceae)
Source: PLoS One. 2016 Jul 7;11(7):e0158691. doi: 10.1371/journal.pone.0158691 (PMC4936701; doi:10.1371/journal.pone.0158691)
Supplement: S6 Table — Negative values are effectively zero. The fraction of FST due to climate group (CG) was estimated using the variance components obtained from hierfstat (i.e. σCG2/(σCG2 + σPOP2)). (DOCX) [file pone.0158691.s012.docx]

**Table S6. Hierarchical *F*-statistics by locus for the SSRs ordered from largest to smallest.** Negative values are effectively zero. The fraction of *F_ST_* due to climate group (CG) was estimated using the variance components obtained from hierfstat (i.e. σ_CG_^2^/(σ_CG_^2^ + σ_POP_^2^)).

| **SSR Locus** | ***F_ST_*** | ***G*_ST_*’*** | ***F_CG,T_*** | **Fraction due to CG** |
| --- | --- | --- | --- | --- |
| RPS119 | 0.202 | 0.467 | 0.028 | 0.141 |
| RPS34b | 0.185 | 0.625 | -0.009 | -0.051 |
| RPS1b | 0.157 | 0.500 | 0.003 | 0.019 |
| RPS60 | 0.150 | 0.566 | 0.019 | 0.122 |
| RPS127 | 0.140 | 0.334 | 0.020 | 0.143 |
| RPS2 | 0.127 | 0.459 | 0.053 | 0.398 |
| RPS118 | 0.122 | 0.362 | 0.067 | 0.535 |
| RPS20 | 0.083 | 0.573 | 0.002 | 0.024 |
| RPS25 | 0.082 | 0.434 | 0.011 | 0.126 |
| RPS39 | 0.063 | 0.257 | 0.006 | 0.099 |
| RPS50 | 0.039 | 0.292 | -0.001 | -0.034 |
| RPS12 | 0.026 | 0.255 | 0.001 | 0.057 |
