# Supplementary material for: Single-Locus versus Multilocus Patterns of Local Adaptation to Climate in Eastern White Pine (Pinus strobus, Pinaceae)
Source: PLoS One. 2016 Jul 7;11(7):e0158691. doi: 10.1371/journal.pone.0158691 (PMC4936701; doi:10.1371/journal.pone.0158691)
Supplement: S7 Table — Negative values are effectively zero. The fraction of FST due to climate group (CG) was estimated using the variance components (σ2) obtained from hierfstat (i.e. σCG2/(σCG2 + σPOP2)). (DOCX) [file pone.0158691.s013.docx]

**Table S7.** **Hierarchical *F*-statistics by locus for the SNPs ordered from largest to smallest.** Negative values are effectively zero. The fraction of *F_ST_* due to climate group (CG) was estimated using the variance components (σ^2^) obtained from hierfstat (i.e. σ_CG_^2^/(σ_CG_^2^ + σ_POP_^2^)).

| **SNP Locus** | ***F_ST_*** | ***G*_ST_*’*** | ***F_CG,T_*** | **Fraction due to CG** |
| --- | --- | --- | --- | --- |
| RPSS14_03 | 0.287 | 0.409 | 0.193 | 0.627 |
| RPSS86_01 | 0.282 | 0.407 | 0.044 | 0.160 |
| RPSS61_03 | 0.229 | 0.242 | 0.152 | 0.624 |
| RPSS61_02 | 0.189 | 0.231 | -0.002 | -0.008 |
| RPSS61_06 | 0.186 | 0.211 | 0.138 | 0.686 |
| RPSS77_04 | 0.174 | 0.230 | 0.063 | 0.356 |
| RPSS14_06 | 0.171 | 0.207 | 0.058 | 0.327 |
| RPSS36_05 | 0.159 | -0.098 | 0.014 | 0.086 |
| RPSS16_01 | 0.155 | 0.157 | 0.051 | 0.327 |
| RPSS86_04 | 0.153 | 0.187 | 0.044 | 0.285 |
| RPSS05_01 | 0.150 | 0.162 | 0.067 | 0.422 |
| RPSS08_01 | 0.134 | 0.176 | 0.028 | 0.216 |
| RPSS05_05 | 0.129 | 0.226 | 0.028 | 0.222 |
| RPSS04_03 | 0.123 | 0.074 | 0.094 | 0.703 |
| RPSS05_04 | 0.122 | 0.109 | 0.101 | 0.757 |
| RPSS66_04 | 0.118 | 0.139 | 0.046 | 0.382 |
| RPSS62_01 | 0.115 | 0.172 | 0.099 | 0.793 |
| RPSS96_02 | 0.112 | 0.148 | 0.042 | 0.365 |
| RPSS33_01 | 0.109 | 0.149 | 0.031 | 0.286 |
| RPSS31_02 | 0.106 | 0.114 | 0.055 | 0.494 |
| RPSS30_01 | 0.104 | 0.141 | 0.040 | 0.375 |
| RPSS04_02 | 0.098 | 0.126 | 0.028 | 0.285 |
| RPSS71_02 | 0.095 | 0.136 | 0.047 | 0.478 |
| RPSS16_03 | 0.092 | 0.081 | 0.125 | 1.150 |
| RPSS87_05 | 0.090 | 0.110 | 0.033 | 0.348 |
| RPSS47_04 | 0.089 | 0.105 | -0.020 | -0.222 |
| RPSS86_06 | 0.089 | 0.112 | 0.022 | 0.244 |
| RPSS30_02 | 0.074 | 0.093 | -0.016 | -0.216 |
| RPSS03_05 | 0.067 | 0.084 | 0.051 | 0.719 |
| RPSS86_02 | 0.065 | 0.086 | 0.020 | 0.292 |
| RPSS62_02 | 0.059 | 0.063 | 0.010 | 0.167 |
| RPSS28_04 | 0.056 | 0.046 | 0.028 | 0.495 |
| RPSS32_03 | 0.044 | 0.051 | 0.010 | 0.216 |
| RPSS28_06 | 0.044 | 0.037 | 0.044 | 0.877 |
| RPSS61_05 | 0.043 | 0.045 | 0.028 | 0.591 |
| RPSS06_03 | 0.040 | 0.034 | 0.039 | 0.865 |
| RPSS31_01 | 0.028 | 0.047 | 0.036 | 1.096 |
| RPSS28_01 | 0.021 | -0.199 | 0.026 | 1.095 |
| RPSS28_03 | 0.020 | 0.017 | 0.030 | 1.239 |
| RPSS28_05 | 0.017 | 0.015 | 0.002 | 0.123 |
| RPSS33_03 | 0.017 | 0.022 | 0.012 | 0.657 |
| RPSS08_03 | 0.012 | 0.009 | 0.015 | 1.080 |
| RPSS07_01 | 0.011 | 0.009 | 0.006 | 0.502 |
| RPSS47_02 | -0.004 | 0.002 | -0.003 | 0.517 |
