# Supplementary material for: Single-Locus versus Multilocus Patterns of Local Adaptation to Climate in Eastern White Pine (Pinus strobus, Pinaceae)
Source: PLoS One. 2016 Jul 7;11(7):e0158691. doi: 10.1371/journal.pone.0158691 (PMC4936701; doi:10.1371/journal.pone.0158691)
Supplement: S8 Table — (DOCX) [file pone.0158691.s014.docx]

**Table S8. Attributes for the Population Graphs resulting from each marker set**

| **Graph Attribute** | **SNPs** | **SSRs** | **Consensus** |
| --- | --- | --- | --- |
| Diameter | 15.99 | 36.61 | 9.00 |
| Components | 1 | 1 | 8 |
| Order | 29 | 29 | 20 |
| Average degree (sd) | 5.86 (1.84) | 6.96 (2.01) | 1.86 (1.43) |
| Average closeness (sd) | 0.0047 (0.0005) | 0.0018 (0.0002) | 0.0025 (0.0009) |
