# Supplementary material for: Single-Locus versus Multilocus Patterns of Local Adaptation to Climate in Eastern White Pine (Pinus strobus, Pinaceae)
Source: PLoS One. 2016 Jul 7;11(7):e0158691. doi: 10.1371/journal.pone.0158691 (PMC4936701; doi:10.1371/journal.pone.0158691)
Supplement: S9 Table — (PDF) [file pone.0158691.s015.pdf]

Single-locus versus Multilocus Patterns of Local Adaptation to Climate in Eastern White Pine (Pinus strobus, Pinaceae)

Om P. Rajora, Andrew J. Eckert, and John W. R. Zinck

Microsatellite Genotype Data

Sample	Population	Longitude	Latitude	RSP1	RSP1.1	RSP2	RSP2.1	RSP12	RSP12.1	RSP20	RSP20	RSP25	RSP25.1	RSP34b	RSP34b.1	RSP39	RSP39.1	RSP50	RSP50.1	RSP60	RSP60.1	RSP127	RSP127.1	RSP118	RSP118.1	RSP119	RSP119.1
NBPH01	NBPMH	-65.29570278	45.78598611	230	232	183	183	204	204	158	164	128	132	176	184	193	195	190	194	255	259	212	214	139	147	163	165
NBPH02	NBPMH	-65.29570278	45.78598611	230	234	183	195	204	208	152	168	130	130	176	184	193	195	190	190	255	259	212	216	139	145	163	167
NBPH03	NBPMH	-65.29570278	45.78598611	230	232	183	195	186	188	146	158	130	130	166	186	185	193	190	190	257	271	212	214	139	145	163	165
NBPH04	NBPMH	-65.29570278	45.78598611	228	234	183	197	212	214	160	176	130	132	156	168	191	191	188	192	257	271	214	216	139	145	165	167
NBPH05	NBPMH	-65.29570278	45.78598611	230	230	185	197	208	208	158	176	128	132	164	168	191	193	192	196	255	275	170	216	139	145	167	167
NBPH06	NBPMH	-65.29570278	45.78598611	230	230	183	183	204	214	160	174	132	132	168	168	191	191	192	196	255	273	214	216	139	145	165	167
NBPH07	NBPMH	-65.29570278	45.78598611	230	230	183	185	208	210	150	150	130	130	166	170	179	191	190	192	255	271	198	214	139	145	149	165
NBPH08	NBPMH	-65.29570278	45.78598611	230	232	183	185	208	210	152	152	132	132	146	164	189	191	190	192	241	253	214	214	139	147	165	165
NBPH09	NBPMH	-65.29570278	45.78598611	230	230	183	183	174	174	158	174	132	160	166	166	189	201	188	192	255	269	170	214	139	145	165	165
NBPH10	NBPMH	-65.29570278	45.78598611	230	234	187	187	192	210	152	162	130	134	156	166	189	191	190	190	255	271	212	214	137	145	163	165
NBPH11	NBPMH	-65.29570278	45.78598611	230	238	185	185	210	216	154	162	134	136	144	162	181	181	164	188	255	271	214	216	139	145	165	167
NBPH12	NBPMH	-65.29570278	45.78598611	230	234	187	197	210	210	154	158	132	134	166	166	189	199	190	190	255	271	214	216	139	145	165	167
NBPH13	NBPMH	-65.29570278	45.78598611	228	228	179	185	200	210	148	158	132	132	166	166	189	189	190	190	255	271	214	214	139	145	165	165
NBPH14	NBPMH	-65.29570278	45.78598611	230	234	171	187	208	208	152	158	128	132	166	166	189	191	164	164	255	271	214	214	137	145	165	165
NBPH15	NBPMH	-65.29570278	45.78598611	234	240	183	185	188	210	164	164	128	132	166	182	175	189	190	190	239	263	212	216	139	145	163	167
NBPH16	NBPMH	-65.29570278	45.78598611	232	238	185	199	172	210	148	156	134	134	166	166	189	191	188	206	255	271	214	214	139	145	165	165
NBPH17	NBPMH	-65.29570278	45.78598611	232	234	185	185	204	216	146	158	130	130	166	166	191	191	194	194	255	273	170	214	139	147	165	165
NBPH18	NBPMH	-65.29570278	45.78598611	232	232	183	183	204																			

216	166	174	126	132	144	162	175	189	196	198
255	271	212	216	139	145	163	167			
NBPH19	NBPMH	-65.29570278		45.78598611		232	234	183	183	190
224	154	174	130	132	156	166	175	193	188	200
255	271	212	216	137	145	163	167			
NBPH20	NBPMH	-65.29570278		45.78598611		232	232	183	197	208
210	158	172	130	130	166	168	189	191	164	190
255	271	192	214	139	145	165	165			
NBPH21	NBPMH	-65.29570278		45.78598611		232	234	183	183	206
206	158	172	128	132	156	166	191	191	164	194
255	271	192	216	139	145	167	167			
NBPH22	NBPMH	-65.29570278		45.78598611		230	234	183	183	204
210	164	164	130	130	144	166	177	191	164	188
255	271	212	216	139	145	163	167			
NBPH23	NBPMH	-65.29570278		45.78598611		228	234	185	197	188
188	160	174	130	130	156	166	187	189	186	194
255	271	214	214	139	145	165	165			
NBPH24	NBPMH	-65.29570278		45.78598611		230	234	183	197	210
210	158	174	130	130	166	168	189	191	190	198
255	271	214	214	139	145	165	165			
NBPH25	NBPMH	-65.29570278		45.78598611		232	238	181	181	208
210	160	176	124	128	166	184	191	193	190	192
255	271	214	218	139	147	165	169			
NBPH26	NBPMH	-65.29570278		45.78598611		230	234	181	195	206
208	160	176	130	134	184	184	191	193	164	190
255	271	216	216	139	145	167	167			
NBPH27	NBPMH	-65.29570278		45.78598611		226	234	183	193	188
204	160	174	130	134	184	184	189	191	164	192
257	271	216	216	139	145	167	167			
NBPH28	NBPMH	-65.29570278		45.78598611		216	232	183	195	188
208	160	174	128	132	174	184	191	191	190	200
257	271	214	214	139	145	165	165			
NBPH29	NBPMH	-65.29570278		45.78598611		232	232	183	195	210
210	152	152	128	130	174	184	191	193	192	198
257	271	192	214	139	145	165	165			
NBPH30	NBPMH	-65.29570278		45.78598611		232	238	193	195	188
208	158	158	130	130	174	184	191	193	190	198
255	259	214	216	139	145	165	167			
NBPH31	NBPMH	-65.29570278		45.78598611		230	234	183	191	210
210	158	176	130	132	174	184	191	193	188	194
257	269	214	216	139	145	165	167			
NBPH32	NBPMH	-65.29570278		45.78598611		230	232	169	191	182
210	160	176	130	132	174	184	191	193	190	196
257	269	192	216	139	145	167	167			
NBPH33	NBPMH	-65.29570278		45.78598611		230	232	183	191	206
210	158	160	130	132	168	168	189	189	188	190
257	269	170	214	139	145	165	165			
NBPH34	NBPMH	-65.29570278		45.78598611		238	238	183	185	210
224	164	164	130	130	168	168	189	191	188	190
255	273	212	214	139	145	163	165			
NBPH35	NBPMH	-65.29570278		45.78598611		230	232	183	195	190
220	152	152	132	132	168	168	189	189	186	190
255	271	214	214	139	145	165	165			
NBPH36	NBPMH	-65.29570278		45.78598611		230	232	183	183	210
228	158	168	130	132	168	168	193	193	186	190
255	271	214	214	137	145	165	165			
NBPH37	NBPMH	-65.29570278		45.78598611		230	230	183	185	206
208	152	152	128	130	168	170	193	193	186	194
255	271	214	216	139	145	165	167			
NBPH38	NBPMH	-65.29570278		45.78598611		230	234	183	183	208
208	160	176	126	132	168	168	191	191	186	190
255	273	216	216	139	145	167	167			
NBPH39	NBPMH	-65.29570278		45.78598611		230	232	183	191	190

208	164	174	128	130	166	166	193	201	188	196
255	273	212	216	139	145	163	167			
NBPH40	NBPMH	-65.29570278		45.78598611		230	232	185	195	202
228	148	158	126	126	166	168	191	193	190	190
255	273	214	214	139	145	165	165			
NBPH41	NBPMH	-65.29570278		45.78598611		218	234	183	183	182
190	160	176	126	126	166	166	191	193	194	204
257	271	214	218	139	147	165	169			
NBPH42	NBPMH	-65.29570278		45.78598611		232	232	193	197	208
210	160	164	128	130	168	168	191	193	164	194
257	273	192	214	139	145	165	165			
NBPH43	NBPMH	-65.29570278		45.78598611		232	232	183	185	206
210	160	164	128	130	168	168	193	193	164	164
257	273	198	214	139	145	149	165			
NBPH44	NBPMH	-65.29570278		45.78598611		230	234	183	191	190
210	148	158	128	130	166	168	191	203	184	192
251	263	214	216	137	145	165	167			
NBPH45	NBPMH	-65.29570278		45.78598611		232	234	183	183	208
208	160	174	128	130	168	168	191	191	184	190
257	271	192	216	139	145	167	167			
NBPH46	NBPMH	-65.29570278		45.78598611		230	234	183	183	210
210	158	160	130	132	170	170	191	191	164	188
257	271	214	214	139	145	165	165			
NBPH47	NBPMH	-65.29570278		45.78598611		232	232	185	191	190
210	162	166	132	134	168	168	193	193	186	190
257	271	214	214	139	145	165	165			
NBPH48	NBPMH	-65.29570278		45.78598611		230	234	183	185	206
206	168	168	126	130	168	168	191	193	160	190
257	271	214	216	139	145	165	167			
NBPH49	NBPMH	-65.29570278		45.78598611		230	234	185	195	190
210	158	176	132	132	166	168	191	193	196	198
255	273	212	216	139	145	163	167			
NBPH50	NBPMH	-65.29570278		45.78598611		218	232	183	185	210
210	140	156	130	134	168	168	201	201	196	206
255	273	212	216	139	145	163	167			
NBCI01	NBCI	-65.59094167		46.14589722		204	228	183	183	210
210	156	170	130	130	164	168	191	193	190	190
255	255	212	212	139	147	167	167			
NBCI02	NBCI	-65.59094167		46.14589722		230	230	183	183	190
204	154	172	122	124	166	170	191	193	188	190
255	259	212	214	139	145	167	169			
NBCI03	NBCI	-65.59094167		46.14589722		228	230	177	191	190
210	148	156	128	130	166	168	179	193	188	198
257	257	212	214	139	145	167	169			
NBCI04	NBCI	-65.59094167		46.14589722		230	230	179	181	190
210	158	172	138	138	164	168	193	203	188	190
257	257	212	212	139	145	167	167			
NBCI05	NBCI	-65.59094167		46.14589722		228	232	181	181	206
206	156	172	146	146	164	166	177	193	194	196
257	257	212	212	139	145	167	167			
NBCI06	NBCI	-65.59094167		46.14589722		230	230	183	185	208
208	156	172	NA	NA	164	166	193	193	190	200
255	255	212	212	137	145	167	167			
NBCI07	NBCI	-65.59094167		46.14589722		232	246	181	183	214
214	168	168	128	130	156	166	179	191	188	188
257	261	212	212	139	145	167	167			
NBCI08	NBCI	-65.59094167		46.14589722		230	230	167	181	208
216	156	156	126	134	146	160	185	185	200	200
257	261	212	214	139	145	167	169			
NBCI09	NBCI	-65.59094167		46.14589722		228	230	181	191	190
206	156	156	130	134	164	166	177	191	186	190
255	273	212	214	139	147	167	169			
NBCI10	NBCI	-65.59094167		46.14589722		232	244	173	181	202

208	168	170	132	132	164	166	177	191	188	188
257	259	212	212	141	145	167	167			
NBCI11	NBCI	-65.59094167		46.14589722		230	246	169	185	206
210	152	160	126	128	164	168	185	195	196	196
259	259	212	214	139	145	167	169			
NBCI12	NBCI	-65.59094167		46.14589722		230	246	183	183	192
210	156	156	128	132	164	168	191	193	198	198
257	261	212	212	139	145	167	167			
NBCI13	NBCI	-65.59094167		46.14589722		230	234	169	183	204
204	156	160	132	132	156	166	191	193	200	200
259	261	214	214	139	145	169	169			
NBCI14	NBCI	-65.59094167		46.14589722		232	246	183	183	186
208	168	172	130	130	164	164	189	193	192	192
259	259	212	212	139	147	167	167			
NBCI15	NBCI	-65.59094167		46.14589722		230	230	169	183	206
208	158	172	128	130	164	168	183	193	188	194
259	259	212	212	139	147	167	167			
NBCI16	NBCI	-65.59094167		46.14589722		228	228	183	183	206
206	156	172	130	130	156	166	183	193	230	252
255	259	212	214	139	147	167	169			
NBCI17	NBCI	-65.59094167		46.14589722		228	228	183	183	214
214	156	158	130	132	156	166	191	191	230	252
255	261	212	214	139	147	167	169			
NBCI18	NBCI	-65.59094167		46.14589722		228	228	183	183	208
210	158	170	126	130	164	168	191	201	190	194
259	273	212	212	139	145	167	167			
NBCI19	NBCI	-65.59094167		46.14589722		228	228	183	183	190
218	156	156	134	134	164	166	179	193	180	188
255	257	216	216	139	145	171	171			
NBCI20	NBCI	-65.59094167		46.14589722		228	228	181	191	206
206	156	156	128	132	156	166	191	191	188	190
255	255	212	214	139	145	167	169			
NBCI21	NBCI	-65.59094167		46.14589722		228	228	169	183	208
208	156	156	128	128	144	166	193	193	190	190
255	255	212	214	137	145	167	169			
NBCI22	NBCI	-65.59094167		46.14589722		194	194	183	183	206
208	156	156	132	132	144	156	191	193	190	190
259	259	210	214	139	145	165	169			
NBCI23	NBCI	-65.59094167		46.14589722		228	232	183	183	206
206	158	174	128	128	164	166	193	193	196	204
259	265	212	212	139	145	167	167			
NBCI24	NBCI	-65.59094167		46.14589722		228	228	181	181	190
190	160	170	130	132	156	164	193	193	188	188
259	259	212	212	139	145	167	167			
NBCI25	NBCI	-65.59094167		46.14589722		228	228	181	185	180
190	158	172	130	130	156	164	193	201	200	200
255	255	212	212	139	145	167	167			
NBCI26	NBCI	-65.59094167		46.14589722		228	228	183	185	172
202	156	162	130	130	166	170	191	201	200	200
257	259	216	216	139	145	171	171			
NBCI27	NBCI	-65.59094167		46.14589722		230	244	177	183	190
210	152	160	128	128	168	170	181	193	190	194
257	259	212	212	139	145	167	167			
NBCI28	NBCI	-65.59094167		46.14589722		230	232	183	191	186
210	160	172	128	130	168	168	191	193	188	190
255	255	212	212	139	145	167	167			
NBCI29	NBCI	-65.59094167		46.14589722		230	230	183	183	172
172	158	160	128	130	166	168	191	193	190	190
255	255	216	216	137	145	171	171			
NBCI30	NBCI	-65.59094167		46.14589722		230	230	183	185	192
192	172	172	130	130	166	168	189	193	182	188
255	255	212	212	139	145	167	167			
NBCI31	NBCI	-65.59094167		46.14589722		230	230	183	183	208

210	158	170	130	130	166	168	189	191	192	192
255	255	210	212	139	145	165	167			
NBCI32	NBCI	-65.59094167		46.14589722		228	228	183	209	192
208	154	158	132	132	166	168	189	191	188	188
255	255	212	214	139	145	167	169			
NBCI33	NBCI	-65.59094167		46.14589722		228	228	183	183	186
208	156	156	132	132	166	166	201	201	190	192
255	255	212	214	139	147	167	169			
NBCI34	NBCI	-65.59094167		46.14589722		224	232	185	185	184
204	158	158	130	132	166	166	189	189	194	200
253	257	212	214	139	145	167	169			
NBCI35	NBCI	-65.59094167		46.14589722		226	236	185	191	212
216	150	158	122	128	166	168	185	189	194	200
255	259	NA	NA	139	145	NA	NA			
NBCI36	NBCI	-65.59094167		46.14589722		226	236	183	183	192
204	172	174	130	132	166	168	191	201	188	204
257	261	212	214	135	145	167	169			
NBCI37	NBCI	-65.59094167		46.14589722		226	232	183	183	192
204	156	156	132	132	156	166	191	191	194	206
255	259	212	212	139	145	167	167			
NBCI38	NBCI	-65.59094167		46.14589722		228	228	183	191	180
192	172	184	132	136	166	166	189	189	178	188
255	255	204	204	139	145	159	159			
NBCI39	NBCI	-65.59094167		46.14589722		228	232	183	185	208
212	158	172	130	130	166	166	189	191	190	190
255	255	212	212	139	145	167	167			
NBCI40	NBCI	-65.59094167		46.14589722		232	232	185	185	208
210	156	168	128	136	166	166	189	189	190	200
255	255	212	212	139	145	167	167			
NBCI41	NBCI	-65.59094167		46.14589722		232	232	183	183	210
216	170	172	130	132	156	166	189	189	194	200
255	255	212	214	139	145	167	169			
NBCI42	NBCI	-65.59094167		46.14589722		232	232	183	185	206
206	150	164	130	130	164	166	187	189	188	200
261	261	212	216	139	145	167	171			
NBCI43	NBCI	-65.59094167		46.14589722		230	230	169	185	196
208	150	158	130	132	164	166	177	191	192	202
261	261	212	212	139	145	167	167			
NBCI44	NBCI	-65.59094167		46.14589722		238	238	181	183	192
214	158	158	132	132	156	166	187	189	196	198
261	261	212	212	139	145	167	167			
NBCI45	NBCI	-65.59094167		46.14589722		232	232	183	185	192
212	158	160	128	128	156	166	191	197	194	200
257	261	214	214	139	145	169	169			
NBCI46	NBCI	-65.59094167		46.14589722		230	232	183	183	192
212	158	160	128	130	156	184	189	191	200	200
259	277	212	216	139	145	167	171			
NBCI47	NBCI	-65.59094167		46.14589722		232	232	183	183	208
212	158	166	130	132	156	166	191	191	188	200
257	257	212	216	135	145	167	171			
NBCI48	NBCI	-65.59094167		46.14589722		234	234	183	183	188
212	158	158	130	130	166	166	189	191	194	198
257	257	212	212	139	145	167	167			
NBCI49	NBCI	-65.59094167		46.14589722		230	230	181	181	212
212	158	158	132	132	166	166	189	191	190	190
257	257	212	212	139	145	167	167			
NBCI50	NBCI	-65.59094167		46.14589722		224	230	183	185	210
210	156	156	134	138	156	166	175	189	188	190
255	259	212	212	139	145	167	167			
NBCR01	NBCR	-65.929375		46.32062222		230	234	185	187	212
214	144	160	124	128	168	168	191	193	192	196
251	251	214	214	139	147	165	165			
NBCR02	NBCR	-65.929375		46.32062222		230	230	185	187	198

212	148	162	126	128	168	168	193	193	190	190
251	251	216	216	139	145	167	167			
NBCR03	NBCR	-65.929375		46.32062222		230	232	175	187	206
210	148	162	124	130	168	170	189	191	188	202
251	255	170	214	139	145	165	165			
NBCR04	NBCR	-65.929375		46.32062222		232	232	185	187	210
212	150	162	124	130	146	170	193	201	160	200
251	251	198	214	139	145	149	165			
NBCR05	NBCR	-65.929375		46.32062222		232	232	175	189	208
210	148	160	122	126	162	166	191	191	188	198
251	251	198	214	139	147	149	165			
NBCR06	NBCR	-65.929375		46.32062222		232	232	175	187	210
210	158	168	126	130	142	166	191	191	192	194
251	251	212	216	137	147	163	167			
NBCR07	NBCR	-65.929375		46.32062222		232	234	175	185	210
214	158	160	126	130	144	166	191	191	188	190
251	251	198	212	139	145	149	163			
NBCR08	NBCR	-65.929375		46.32062222		234	234	175	187	210
214	144	158	122	122	144	168	191	191	194	196
251	253	198	198	139	145	149	165			
NBCR09	NBCR	-65.929375		46.32062222		246	246	189	189	210
212	156	158	132	136	166	166	191	191	204	206
251	251	214	214	139	147	165	165			
NBCR10	NBCR	-65.929375		46.32062222		214	214	175	187	202
208	160	174	130	130	166	168	189	191	198	206
251	251	214	214	141	145	165	165			
NBCR11	NBCR	-65.929375		46.32062222		226	236	173	189	194
208	140	158	118	122	166	168	191	191	202	202
253	257	212	212	139	145	163	163			
NBCR12	NBCR	-65.929375		46.32062222		236	236	175	189	206
210	158	158	122	126	166	166	191	193	200	206
257	257	216	218	139	145	167	169			
NBCR13	NBCR	-65.929375		46.32062222		238	238	187	189	204
208	158	176	122	126	162	166	191	191	190	194
253	253	212	214	139	145	163	165			
NBCR14	NBCR	-65.929375		46.32062222		238	238	187	191	206
212	158	174	118	120	164	166	191	193	188	196
251	253	214	214	139	147	165	165			
NBCR15	NBCR	-65.929375		46.32062222		238	238	175	187	192
200	168	168	106	120	164	170	189	195	160	200
253	257	210	216	139	147	161	167			
NBCR16	NBCR	-65.929375		46.32062222		238	238	175	189	200
206	172	176	120	124	162	166	189	195	194	194
251	255	198	214	139	147	149	165			
NBCR17	NBCR	-65.929375		46.32062222		222	222	189	191	192
208	160	174	122	124	166	168	189	195	190	194
251	251	198	214	139	147	149	165			
NBCR18	NBCR	-65.929375		46.32062222		222	224	189	191	192
208	158	174	126	126	166	170	191	193	192	192
251	251	214	214	139	145	165	165			
NBCR19	NBCR	-65.929375		46.32062222		224	224	187	189	172
190	160	174	126	126	162	166	191	193	200	200
251	251	198	214	139	145	149	165			
NBCR20	NBCR	-65.929375		46.32062222		222	224	187	189	192
210	158	182	122	122	166	168	191	193	198	198
251	251	214	216	139	145	165	167			
NBCR21	NBCR	-65.929375		46.32062222		222	222	175	187	192
210	174	174	130	132	154	168	191	193	198	198
251	251	214	214	137	145	165	165			
NBCR22	NBCR	-65.929375		46.32062222		222	222	175	187	206
206	174	174	130	132	158	172	191	193	204	206
253	253	214	214	139	145	165	165			
NBCR23	NBCR	-65.929375		46.32062222		214	214	175	187	196

208	160	174	130	132	164	168	191	193	188	198
253	253	212	216	139	145	163	167			
NBCR24	NBCR	-65.929375	46.32062222	222	222	187	189	208		
218	158	174	130	132	164	168	189	193	188	188
251	251	214	216	139	145	165	167			
NBCR25	NBCR	-65.929375	46.32062222	246	246	187	189	208		
208	166	166	130	132	168	172	195	195	170	188
251	251	214	214	139	145	165	165			
NBCR26	NBCR	-65.929375	46.32062222	222	222	187	189	206		
216	164	166	130	132	166	168	191	193	190	190
253	255	214	214	139	145	165	165			
NBCR27	NBCR	-65.929375	46.32062222	222	224	175	187	182		
216	166	166	130	132	166	166	189	193	190	190
255	255	214	214	139	145	165	165			
NBCR28	NBCR	-65.929375	46.32062222	230	246	175	189	216		
218	146	162	120	124	168	168	191	191	194	194
253	253	214	214	139	145	165	165			
NBCR29	NBCR	-65.929375	46.32062222	222	222	189	191	218		
220	160	160	122	124	168	170	191	191	190	192
253	253	216	216	135	145	167	167			
NBCR30	NBCR	-65.929375	46.32062222	246	246	187	189	194		
210	174	176	130	132	168	168	191	193	160	200
253	253	214	216	139	145	165	167			
NBCR31	NBCR	-65.929375	46.32062222	222	222	175	185	188		
192	158	166	120	124	168	170	189	191	176	190
253	253	198	214	139	145	149	165			
NBCR32	NBCR	-65.929375	46.32062222	244	244	187	187	208		
210	144	158	122	124	156	156	189	191	190	196
253	253	214	214	139	145	165	165			
NBCR33	NBCR	-65.929375	46.32062222	220	220	189	193	206		
210	170	172	130	132	166	166	191	191	192	192
255	257	214	214	139	147	165	165			
NBCR34	NBCR	-65.929375	46.32062222	246	246	175	187	190		
208	172	180	120	124	166	166	189	191	188	188
253	253	198	214	139	145	149	165			
NBCR35	NBCR	-65.929375	46.32062222	214	214	175	189	176		
176	146	174	122	124	166	166	187	191	188	188
253	253	NA	NA	139	145	NA	NA			
NBCR36	NBCR	-65.929375	46.32062222	218	230	175	187	198		
208	158	158	130	132	156	166	199	199	188	188
253	253	214	214	135	145	165	165			
NBCR37	NBCR	-65.929375	46.32062222	224	230	175	187	208		
210	158	158	120	124	168	168	197	197	190	190
253	255	212	214	139	145	163	165			
NBCR38	NBCR	-65.929375	46.32062222	214	214	175	189	180		
188	158	158	122	124	156	166	189	191	192	192
253	253	214	214	139	145	165	165			
NBCR39	NBCR	-65.929375	46.32062222	246	246	187	189	210		
210	158	180	130	132	166	168	189	191	192	198
253	253	198	214	135	145	149	165			
NBCR40	NBCR	-65.929375	46.32062222	246	246	189	189	208		
218	158	160	120	124	168	168	195	197	192	192
253	253	214	218	139	145	165	169			
NBCR41	NBCR	-65.929375	46.32062222	246	246	187	191	202		
204	158	160	122	124	166	168	175	195	160	200
253	255	198	214	139	145	149	165			
NBCR42	NBCR	-65.929375	46.32062222	246	246	189	191	182		
206	156	158	128	136	164	168	191	199	202	204
253	255	216	216	139	145	167	167			
NBCR43	NBCR	-65.929375	46.32062222	246	246	189	189	188		
210	158	158	122	122	164	168	191	193	202	202
253	255	212	214	139	145	163	165			
NBCR44	NBCR	-65.929375	46.32062222	222	222	187	187	210		

212	158	158	122	122	166	166	193	193	202	202
251	251	214	214	139	145	165	165			
NBCR45	NBCR	-65.929375		46.32062222		222	222	177	177	210
220	154	158	130	130	164	168	195	195	202	202
253	253	198	214	139	145	149	165			
NBCR46	NBCR	-65.929375		46.32062222		214	214	177	177	208
208	154	158	128	132	156	166	193	193	202	202
253	255	198	214	139	145	149	165			
NBCR47	NBCR	-65.929375		46.32062222		214	214	175	189	208
210	158	160	126	128	156	166	195	195	190	190
251	251	214	216	135	147	165	167			
NBCR48	NBCR	-65.929375		46.32062222		238	238	187	187	208
210	158	160	124	128	166	168	191	191	202	204
253	255	198	214	139	145	149	165			
NBCR49	NBCR	-65.929375		46.32062222		244	244	187	187	210
210	158	174	122	122	164	166	191	193	198	200
251	251	214	216	139	145	165	167			
NBCR50	NBCR	-65.929375		46.32062222		232	236	187	187	208
220	158	160	122	122	166	168	191	191	160	200
253	253	214	218	139	145	165	169			
NBOP01	NBOP	-66.66477222		45.95601667		230	234	185	187	208
212	172	174	158	176	164	166	193	195	196	196
251	251	214	214	139	147	165	165			
NBOP02	NBOP	-66.66477222		45.95601667		230	230	185	187	210
216	158	168	132	136	164	166	191	193	190	190
251	251	216	216	137	145	167	167			
NBOP03	NBOP	-66.66477222		45.95601667		230	232	185	187	208
210	158	174	130	132	164	166	191	193	188	202
251	255	170	214	139	145	165	165			
NBOP04	NBOP	-66.66477222		45.95601667		232	232	185	187	208
210	156	160	134	138	166	168	193	193	196	200
251	251	198	214	139	145	149	165			
NBOP05	NBOP	-66.66477222		45.95601667		232	232	175	189	208
212	158	174	152	158	166	168	191	195	188	198
253	253	198	214	139	145	149	165			
NBOP06	NBOP	-66.66477222		45.95601667		232	232	175	185	208
210	156	158	130	134	166	168	191	191	192	194
251	251	212	216	137	145	163	167			
NBOP07	NBOP	-66.66477222		45.95601667		232	234	175	185	202
206	158	160	118	130	164	168	191	211	188	190
251	253	198	212	139	145	149	163			
NBOP08	NBOP	-66.66477222		45.95601667		232	234	175	185	208
210	160	178	128	132	166	168	191	193	194	196
251	253	198	198	139	145	149	151			
NBOP09	NBOP	-66.66477222		45.95601667		232	246	185	189	208
214	162	174	120	130	166	168	191	193	204	206
251	251	214	214	139	147	165	165			
NBOP10	NBOP	-66.66477222		45.95601667		214	214	175	187	186
210	158	172	134	136	164	166	191	193	198	206
251	251	214	214	141	145	165	165			
NBOP11	NBOP	-66.66477222		45.95601667		232	236	173	189	210
214	158	174	124	132	164	166	191	193	202	202
253	257	212	212	139	145	163	163			
NBOP12	NBOP	-66.66477222		45.95601667		236	236	175	189	208
212	176	178	110	130	166	168	189	191	200	206
257	257	216	218	139	145	167	169			
NBOP13	NBOP	-66.66477222		45.95601667		238	238	187	189	206
212	158	160	128	130	166	170	175	191	190	202
253	253	212	214	135	145	163	165			
NBOP14	NBOP	-66.66477222		45.95601667		238	238	187	191	204
208	160	178	132	134	164	166	189	191	188	196
251	253	214	214	139	147	165	165			
NBOP15	NBOP	-66.66477222		45.95601667		238	238	185	187	208

212	158	162	132	134	166	168	191	193	160	200
253	257	210	216	139	147	161	167			
NBOP16	NBOP	-66.66477222		45.95601667		238	238	175	189	208
212	162	172	118	134	164	168	193	193	194	194
253	255	198	214	139	147	149	165			
NBOP17	NBOP	-66.66477222		45.95601667		222	232	189	191	190
206	162	176	148	152	166	168	191	193	190	194
253	255	198	214	139	147	149	165			
NBOP18	NBOP	-66.66477222		45.95601667		222	224	189	191	188
190	144	158	110	130	166	168	191	193	188	192
253	255	214	214	139	145	165	165			
NBOP19	NBOP	-66.66477222		45.95601667		224	224	187	189	188
206	158	176	134	136	166	168	191	193	192	200
251	251	198	214	139	145	149	165			
NBOP20	NBOP	-66.66477222		45.95601667		222	224	187	189	190
194	158	172	106	130	166	168	189	193	198	198
253	253	214	216	139	145	165	167			
NBOP21	NBOP	-66.66477222		45.95601667		222	222	185	187	190
194	158	170	142	144	166	170	191	191	198	198
251	251	214	214	137	145	165	165			
NBOP22	NBOP	-66.66477222		45.95601667		222	222	187	187	200
208	106	160	130	142	164	166	189	191	204	206
253	253	214	214	139	147	165	165			
NBOP23	NBOP	-66.66477222		45.95601667		214	214	175	187	210
212	158	168	128	130	166	170	189	191	188	198
253	253	212	216	139	147	163	167			
NBOP24	NBOP	-66.66477222		45.95601667		222	222	187	189	172
178	106	158	144	146	164	168	189	191	188	188
251	251	214	216	139	145	165	167			
NBOP25	NBOP	-66.66477222		45.95601667		246	246	187	189	194
206	158	178	130	132	164	168	191	191	188	188
251	251	214	214	139	145	165	165			
NBOP26	NBOP	-66.66477222		45.95601667		222	222	187	189	208
214	106	158	128	130	164	166	191	193	190	190
253	255	214	214	139	145	165	165			
NBOP27	NBOP	-66.66477222		45.95601667		222	224	175	187	206
208	106	158	128	134	166	170	191	201	190	190
255	255	214	214	139	145	165	165			
NBOP28	NBOP	-66.66477222		45.95601667		230	246	175	189	192
208	106	160	128	128	164	166	191	193	194	194
253	253	214	214	139	141	165	165			
NBOP29	NBOP	-66.66477222		45.95601667		222	222	189	191	202
206	156	158	134	138	164	168	189	191	192	192
253	253	216	216	137	145	167	167			
NBOP30	NBOP	-66.66477222		45.95601667		246	246	187	189	202
208	170	174	106	130	164	166	191	193	160	200
253	253	214	216	139	145	165	167			
NBOP31	NBOP	-66.66477222		45.95601667		222	222	175	185	190
194	158	174	130	130	166	168	189	191	176	190
251	253	198	214	139	145	149	165			
NBOP32	NBOP	-66.66477222		45.95601667		244	244	187	187	210
214	160	176	136	146	164	168	193	201	190	196
251	253	214	214	139	145	165	165			
NBOP33	NBOP	-66.66477222		45.95601667		232	234	189	193	186
188	162	172	128	130	166	168	191	193	192	192
251	257	214	214	139	147	165	165			
NBOP34	NBOP	-66.66477222		45.95601667		232	234	175	187	190
192	156	158	132	136	164	168	191	191	188	188
253	253	198	214	139	145	149	165			
NBOP35	NBOP	-66.66477222		45.95601667		232	246	175	189	192
196	158	172	118	132	164	166	191	191	188	188
253	253	NA	NA	139	145	NA	NA			
NBOP36	NBOP	-66.66477222		45.95601667		214	214	187	187	188

216	160	176	132	136	164	168	191	193	188	188
253	253	214	214	135	145	165	165			
NBOP37	NBOP	-66.66477222		45.95601667		232	236	187	187	190
210	160	176	132	134	164	166	191	191	186	190
253	255	212	214	139	145	163	165			
NBOP38	NBOP	-66.66477222		45.95601667		236	236	175	189	204
210	106	158	132	136	164	168	193	193	192	192
251	253	214	214	139	145	165	165			
NBOP39	NBOP	-66.66477222		45.95601667		238	238	187	189	210
214	144	160	132	134	166	168	193	193	192	198
253	253	198	214	139	145	149	165			
NBOP40	NBOP	-66.66477222		45.95601667		238	238	189	189	208
208	158	176	132	132	166	170	193	193	192	192
253	253	214	218	139	145	165	169			
NBOP41	NBOP	-66.66477222		45.95601667		238	238	187	191	200
204	158	174	130	134	164	166	191	191	160	200
253	255	198	214	139	145	149	165			
NBOP42	NBOP	-66.66477222		45.95601667		246	246	189	191	174
178	106	158	118	150	164	168	189	193	202	204
253	255	216	216	139	145	167	167			
NBOP43	NBOP	-66.66477222		45.95601667		246	246	189	189	174
210	160	178	134	136	166	168	189	191	202	202
253	255	212	214	139	145	163	165			
NBOP44	NBOP	-66.66477222		45.95601667		222	222	187	187	200
204	160	174	108	134	166	168	193	193	200	202
251	251	214	214	139	145	165	165			
NBOP45	NBOP	-66.66477222		45.95601667		222	222	177	177	206
208	160	176	130	154	166	168	191	191	200	202
253	253	198	214	139	145	149	165			
NBOP46	NBOP	-66.66477222		45.95601667		214	214	175	177	190
194	158	174	120	134	162	166	191	191	202	202
253	255	198	214	139	145	149	165			
NBOP47	NBOP	-66.66477222		45.95601667		214	214	175	189	190
212	162	172	134	134	166	168	193	193	186	190
251	251	214	216	135	145	165	167			
NBOP48	NBOP	-66.66477222		45.95601667		238	238	187	187	190
192	162	182	134	138	164	168	193	193	202	204
253	255	198	214	139	145	149	165			
NBOP49	NBOP	-66.66477222		45.95601667		244	244	187	187	202
204	160	176	136	144	162	166	193	193	198	200
251	251	214	216	135	145	165	167			
NBOP50	NBOP	-66.66477222		45.95601667		232	236	187	187	190
212	160	174	122	138	166	168	193	193	160	200
253	253	214	218	135	145	165	169			
NSSMB01	NSSMB	-63.86819167		44.63948611		230	236	183	185	204
206	172	174	134	136	154	178	191	201	196	210
251	251	214	214	139	145	165	165			
NSSMB02	NSSMB	-63.86819167		44.63948611		230	230	181	185	208
210	158	168	124	130	154	154	193	193	194	206
253	253	214	214	139	145	165	165			
NSSMB03	NSSMB	-63.86819167		44.63948611		230	230	185	185	206
208	156	160	130	136	182	182	191	191	196	212
253	269	214	216	139	145	165	167			
NSSMB04	NSSMB	-63.86819167		44.63948611		230	230	185	185	206
210	156	160	128	132	154	156	191	201	206	206
253	271	214	216	139	145	165	167			
NSSMB05	NSSMB	-63.86819167		44.63948611		230	230	181	185	206
210	156	158	132	134	134	154	191	191	196	196
253	253	214	216	139	145	165	167			
NSSMB06	NSSMB	-63.86819167		44.63948611		230	234	185	185	206
210	160	172	136	138	154	154	195	195	194	194
251	253	214	214	137	145	165	165			
NSSMB07	NSSMB	-63.86819167		44.63948611		230	230	183	185	206

210	158	162	136	138	152	152	191	191	196	204
253	267	214	214	139	145	165	165			
NSSMB08	NSSMB	-63.86819167		44.63948611		230	232	183	183	182
212	158	172	132	134	150	154	193	193	200	212
253	267	214	216	139	145	165	167			
NSSMB09	NSSMB	-63.86819167		44.63948611		228	230	181	183	208
208	160	172	134	138	152	152	191	193	196	204
251	255	214	214	137	141	165	165			
NSSMB10	NSSMB	-63.86819167		44.63948611		232	232	183	185	204
210	160	166	132	132	154	154	191	191	196	204
253	269	214	214	139	145	165	165			
NSSMB11	NSSMB	-63.86819167		44.63948611		232	232	183	185	206
210	158	174	128	130	150	158	193	193	196	204
253	253	214	214	139	145	165	165			
NSSMB12	NSSMB	-63.86819167		44.63948611		232	232	183	185	206
210	168	174	130	132	154	154	191	193	198	200
253	253	214	214	139	145	165	165			
NSSMB13	NSSMB	-63.86819167		44.63948611		232	232	183	191	174
210	158	174	130	132	154	154	191	191	206	212
253	253	214	216	139	145	165	167			
NSSMB14	NSSMB	-63.86819167		44.63948611		232	238	185	185	174
188	158	162	128	132	154	154	191	193	212	212
253	269	214	214	139	145	165	165			
NSSMB15	NSSMB	-63.86819167		44.63948611		232	232	183	185	188
192	158	160	128	134	154	154	197	201	196	204
255	271	214	214	141	141	165	165			
NSSMB16	NSSMB	-63.86819167		44.63948611		230	230	183	183	188
188	156	174	130	134	154	154	191	191	204	204
253	269	214	214	139	145	165	165			
NSSMB17	NSSMB	-63.86819167		44.63948611		232	232	183	185	188
188	158	158	130	134	154	154	191	193	194	196
253	253	214	214	139	145	165	165			
NSSMB18	NSSMB	-63.86819167		44.63948611		232	238	183	183	200
210	162	172	134	142	150	154	189	191	192	194
255	255	214	214	139	145	165	165			
NSSMB19	NSSMB	-63.86819167		44.63948611		226	232	185	185	200
210	158	164	130	134	154	154	189	191	194	206
253	253	216	216	145	145	167	167			
NSSMB20	NSSMB	-63.86819167		44.63948611		222	232	181	185	200
210	158	174	132	134	154	154	191	191	196	198
253	253	214	216	139	145	165	167			
NSSMB21	NSSMB	-63.86819167		44.63948611		234	234	183	185	176
200	160	174	136	140	152	154	191	191	200	200
253	253	214	216	139	145	165	167			
NSSMB22	NSSMB	-63.86819167		44.63948611		232	232	183	183	198
200	162	170	140	142	154	156	189	191	198	204
253	269	214	214	139	145	165	165			
NSSMB23	NSSMB	-63.86819167		44.63948611		232	232	183	185	206
208	158	162	134	136	154	156	189	193	196	196
253	269	214	214	135	145	165	165			
NSSMB24	NSSMB	-63.86819167		44.63948611		222	232	183	185	202
208	158	162	132	134	154	154	191	193	198	204
253	269	214	214	139	145	165	165			
NSSMB25	NSSMB	-63.86819167		44.63948611		232	232	181	183	188
192	160	176	134	136	154	156	189	193	196	208
253	253	214	214	139	145	165	165			
NSSMB26	NSSMB	-63.86819167		44.63948611		230	236	183	185	206
208	158	172	134	134	154	154	189	193	196	200
253	253	214	216	139	145	165	167			
NSSMB27	NSSMB	-63.86819167		44.63948611		232	234	181	183	188
208	164	164	134	138	152	158	189	193	200	204
253	253	214	240	139	145	165	165			
NSSMB28	NSSMB	-63.86819167		44.63948611		230	234	183	185	204

206	160	164	132	136	154	158	189	193	194	194
253	253	214	216	139	145	165	167			
NSSMB29	NSSMB	-63.86819167		44.63948611		230	232	181	183	204
208	156	164	132	132	134	156	191	195	196	204
253	253	214	286	139	145	165	165			
NSSMB30	NSSMB	-63.86819167		44.63948611		230	232	183	185	204
208	160	164	132	134	152	158	189	193	194	196
253	253	214	214	137	141	165	165			
NSSMB31	NSSMB	-63.86819167		44.63948611		230	230	185	185	188
208	172	176	134	136	152	154	189	193	194	204
253	253	214	216	139	145	165	167			
NSSMB32	NSSMB	-63.86819167		44.63948611		230	232	183	185	200
202	164	168	130	130	152	154	191	195	196	204
253	269	214	216	139	145	165	167			
NSSMB33	NSSMB	-63.86819167		44.63948611		228	232	183	185	184
204	160	166	132	134	152	156	189	193	194	204
253	253	214	216	139	145	165	167			
NSSMB34	NSSMB	-63.86819167		44.63948611		230	230	183	183	202
204	158	168	134	138	158	178	193	195	194	202
255	271	214	214	139	145	165	165			
NSSMB35	NSSMB	-63.86819167		44.63948611		230	230	181	183	208
208	158	172	134	134	134	156	195	195	194	200
253	253	216	216	139	145	167	167			
NSSMB36	NSSMB	-63.86819167		44.63948611		230	230	183	183	188
204	158	174	120	132	154	156	191	195	198	204
253	267	214	216	139	145	165	167			
NSSMB37	NSSMB	-63.86819167		44.63948611		230	230	169	185	188
204	156	174	134	134	154	156	189	193	196	196
253	269	214	214	139	145	165	165			
NSSMB38	NSSMB	-63.86819167		44.63948611		228	228	169	185	200
204	158	158	134	134	154	154	191	193	194	196
253	269	216	218	139	145	167	169			
NSSMB39	NSSMB	-63.86819167		44.63948611		228	228	169	183	188
188	158	176	132	132	154	154	195	195	196	204
253	253	214	214	139	145	165	165			
NSSMB40	NSSMB	-63.86819167		44.63948611		228	228	181	183	188
204	156	158	128	130	154	156	191	193	196	196
253	267	214	214	139	145	165	165			
NSSMB41	NSSMB	-63.86819167		44.63948611		228	232	183	185	188
188	158	174	132	134	152	152	189	191	196	198
255	269	214	216	137	145	165	167			
NSSMB42	NSSMB	-63.86819167		44.63948611		228	230	183	185	188
188	170	174	132	134	154	154	189	191	196	198
255	269	214	214	137	145	165	165			
NSSMB43	NSSMB	-63.86819167		44.63948611		230	230	183	183	210
210	174	186	130	134	154	154	191	191	196	198
255	269	214	214	139	145	165	165			
NSSMB44	NSSMB	-63.86819167		44.63948611		230	230	171	171	174
174	156	186	130	130	154	154	189	191	200	200
255	269	216	216	139	145	167	167			
NSSMB45	NSSMB	-63.86819167		44.63948611		230	230	185	193	190
190	156	186	130	132	152	152	189	191	202	204
253	269	214	216	139	145	165	167			
NSSMB46	NSSMB	-63.86819167		44.63948611		228	228	185	185	190
190	158	172	130	132	154	154	193	195	194	198
253	253	214	214	137	137	165	165			
NSSMB47	NSSMB	-63.86819167		44.63948611		228	228	185	187	206
208	156	156	128	132	142	156	189	193	196	198
253	253	216	216	139	145	167	167			
NSSMB48	NSSMB	-63.86819167		44.63948611		228	234	185	185	188
190	158	158	132	132	154	154	191	195	196	200
253	269	214	214	139	145	165	165			
NSSMB49	NSSMB	-63.86819167		44.63948611		228	230	183	183	190

190	158	172	128	130	154	154	195	195	196	206
253	269	214	214	139	145	165	165			
NSSMB50	NSSMB	-63.86819167		44.63948611		228	228	181	183	208
210	168	172	130	132	154	154	191	191	208	210
253	267	214	214	139	145	165	165			
NSRL01	NSRL	-65.14228333		44.27316667		228	228	183	185	204
208	160	160	134	136	166	168	191	193	196	196
255	259	212	212	139	145	163	163			
NSRL02	NSRL	-65.14228333		44.27316667		228	228	183	191	182
208	160	162	128	152	168	168	191	193	184	186
257	257	212	212	137	145	163	163			
NSRL03	NSRL	-65.14228333		44.27316667		228	228	185	185	186
208	162	164	128	128	166	166	191	193	186	196
255	259	212	212	139	145	163	163			
NSRL04	NSRL	-65.14228333		44.27316667		226	226	185	187	212
212	160	176	128	130	166	166	191	191	184	184
255	259	212	212	139	145	163	163			
NSRL05	NSRL	-65.14228333		44.27316667		228	228	185	187	182
212	160	160	132	136	164	166	191	191	194	196
255	259	212	212	137	145	163	163			
NSRL06	NSRL	-65.14228333		44.27316667		222	228	185	185	202
210	160	160	128	130	166	168	189	193	194	196
257	257	212	212	139	145	163	163			
NSRL07	NSRL	-65.14228333		44.27316667		228	228	185	187	208
208	160	162	122	126	166	166	191	191	192	196
257	273	212	212	139	145	163	163			
NSRL08	NSRL	-65.14228333		44.27316667		228	228	183	185	182
182	160	174	128	130	166	166	191	201	182	196
257	273	212	212	139	145	163	163			
NSRL09	NSRL	-65.14228333		44.27316667		228	228	185	185	204
208	162	166	128	134	166	166	191	201	184	184
255	273	212	214	141	145	163	165			
NSRL10	NSRL	-65.14228333		44.27316667		228	228	183	187	212
212	162	176	130	134	166	166	191	193	186	186
257	273	214	216	139	145	165	167			
NSRL11	NSRL	-65.14228333		44.27316667		222	222	185	185	182
206	162	162	130	132	166	166	191	191	184	188
255	259	214	216	139	145	165	167			
NSRL12	NSRL	-65.14228333		44.27316667		228	228	185	187	202
208	160	160	130	132	166	166	191	195	192	196
255	259	214	214	139	145	165	165			
NSRL13	NSRL	-65.14228333		44.27316667		228	228	185	187	204
208	160	160	128	130	166	166	193	193	192	194
257	259	212	214	141	145	163	165			
NSRL14	NSRL	-65.14228333		44.27316667		228	232	185	185	204
208	162	162	126	130	166	166	193	193	192	198
257	273	214	214	139	145	165	165			
NSRL15	NSRL	-65.14228333		44.27316667		228	234	185	193	182
206	158	160	122	128	166	166	193	193	184	184
255	261	214	214	139	145	165	165			
NSRL16	NSRL	-65.14228333		44.27316667		232	232	165	185	204
210	178	178	128	130	166	166	193	195	184	198
255	273	214	214	139	145	165	165			
NSRL17	NSRL	-65.14228333		44.27316667		228	228	183	185	182
202	162	162	128	130	166	166	193	193	184	202
255	259	214	216	137	145	165	167			
NSRL18	NSRL	-65.14228333		44.27316667		228	228	183	183	170
208	162	170	132	142	148	166	193	193	186	192
235	257	212	214	139	145	163	165			
NSRL19	NSRL	-65.14228333		44.27316667		228	228	173	173	182
202	162	164	130	134	166	166	193	203	202	204
255	259	214	214	139	145	165	165			
NSRL20	NSRL	-65.14228333		44.27316667		228	228	185	193	208

208	160	164	130	132	166	166	193	195	184	196
255	259	214	214	139	145	165	165			
NSRL21	NSRL	-65.14228333		44.27316667		228	228	183	183	182
212	160	176	128	128	148	166	193	193	184	204
235	257	214	216	139	145	165	167			
NSRL22	NSRL	-65.14228333		44.27316667		228	228	191	191	176
214	162	162	132	136	166	168	193	195	194	194
255	273	214	214	137	145	165	165			
NSRL23	NSRL	-65.14228333		44.27316667		228	228	183	191	206
208	162	162	126	130	166	166	195	195	186	198
255	255	214	214	139	145	165	165			
NSRL24	NSRL	-65.14228333		44.27316667		228	228	183	191	210
222	178	178	128	132	166	166	197	197	184	186
255	255	214	214	139	145	165	165			
NSRL25	NSRL	-65.14228333		44.27316667		228	234	185	187	168
208	160	162	132	136	166	166	195	197	198	198
257	257	212	216	139	145	163	167			
NSRL26	NSRL	-65.14228333		44.27316667		228	228	185	189	182
182	160	160	126	130	148	166	195	195	198	198
255	255	212	214	139	145	163	165			
NSRL27	NSRL	-65.14228333		44.27316667		222	222	185	185	182
184	160	162	128	130	166	166	195	197	188	198
257	259	214	214	137	145	165	165			
NSRL28	NSRL	-65.14228333		44.27316667		228	228	185	185	182
208	160	160	134	134	168	168	197	199	186	198
257	261	214	214	139	145	165	165			
NSRL29	NSRL	-65.14228333		44.27316667		226	230	185	185	218
222	160	172	128	130	166	168	195	197	186	206
235	259	212	214	139	145	163	165			
NSRL30	NSRL	-65.14228333		44.27316667		228	228	187	187	192
212	160	172	134	134	168	168	199	199	186	198
235	257	214	214	139	145	165	165			
NSRL31	NSRL	-65.14228333		44.27316667		228	228	185	185	210
210	176	178	130	132	168	168	197	197	198	198
257	257	214	214	139	145	165	165			
NSRL32	NSRL	-65.14228333		44.27316667		228	228	185	193	170
206	160	176	128	130	168	168	197	199	186	194
257	257	212	214	139	145	163	165			
NSRL33	NSRL	-65.14228333		44.27316667		222	228	185	185	168
212	162	174	126	128	166	170	197	197	188	188
235	257	214	216	137	145	165	167			
NSRL34	NSRL	-65.14228333		44.27316667		222	228	185	193	206
212	160	176	128	130	166	170	197	197	188	188
255	259	214	216	139	145	165	167			
NSRL35	NSRL	-65.14228333		44.27316667		228	228	185	185	194
200	172	178	128	130	168	168	197	199	188	190
235	259	212	214	139	145	163	165			
NSRL36	NSRL	-65.14228333		44.27316667		228	228	185	185	206
212	160	160	128	130	166	168	197	197	186	186
257	273	214	214	139	145	165	165			
NSRL37	NSRL	-65.14228333		44.27316667		228	228	183	189	206
212	160	176	128	130	168	168	199	199	190	194
259	259	214	214	139	145	165	165			
NSRL38	NSRL	-65.14228333		44.27316667		228	234	183	193	168
212	160	174	102	130	168	170	199	199	192	194
257	257	214	214	137	145	165	165			
NSRL39	NSRL	-65.14228333		44.27316667		234	234	183	193	206
212	160	160	128	130	166	166	199	201	196	196
257	257	214	216	139	145	165	167			
NSRL40	NSRL	-65.14228333		44.27316667		228	228	185	187	206
212	160	160	130	130	168	168	199	201	242	242
257	257	214	216	139	145	165	167			
NSRL41	NSRL	-65.14228333		44.27316667		228	228	185	193	206

214	170	176	126	130	168	168	199	199	202	202
257	273	216	216	139	145	167	167			
NSRL42	NSRL	-65.14228333		44.27316667		228	228	185	189	168
212	158	174	124	126	146	168	195	199	186	186
257	259	214	214	137	137	165	165			
NSRL43	NSRL	-65.14228333		44.27316667		228	228	185	193	182
182	158	178	124	128	170	170	197	197	188	188
257	273	214	214	139	145	165	165			
NSRL44	NSRL	-65.14228333		44.27316667		222	228	185	187	168
212	158	168	124	124	150	168	195	197	188	200
235	259	214	216	139	145	165	167			
NSRL45	NSRL	-65.14228333		44.27316667		228	228	185	187	182
202	158	158	128	130	166	168	197	199	186	186
259	273	214	214	139	145	165	165			
NSRL46	NSRL	-65.14228333		44.27316667		228	228	193	195	208
212	158	160	128	136	168	168	199	201	196	198
257	257	212	214	137	141	163	165			
NSRL47	NSRL	-65.14228333		44.27316667		228	228	193	195	208
208	156	156	126	126	168	168	199	207	194	194
257	257	214	214	139	145	165	165			
NSRL48	NSRL	-65.14228333		44.27316667		228	234	191	193	206
212	156	156	130	134	168	168	199	207	186	186
255	259	214	214	139	145	165	165			
NSRL49	NSRL	-65.14228333		44.27316667		228	228	185	187	206
212	156	174	NA	NA	170	170	197	197	192	198
235	257	214	216	139	141	165	167			
NSRL50	NSRL	-65.14228333		44.27316667		228	228	185	187	206
210	156	156	134	138	152	168	195	197	192	198
235	259	214	216	139	145	165	167			
NSDL01	NSDL	-64.40985278		44.50441944		226	226	183	183	192
210	154	154	126	130	166	168	189	189	186	194
249	253	214	216	139	141	165	167			
NSDL02	NSDL	-64.40985278		44.50441944		226	226	181	187	178
178	142	150	126	130	166	168	191	191	188	192
251	251	214	218	139	145	165	169			
NSDL03	NSDL	-64.40985278		44.50441944		226	228	185	187	208
214	140	140	132	132	166	166	189	189	188	190
249	251	216	216	139	145	167	167			
NSDL04	NSDL	-64.40985278		44.50441944		226	228	185	187	188
208	138	138	128	128	166	166	189	191	190	192
251	251	218	218	137	145	169	169			
NSDL05	NSDL	-64.40985278		44.50441944		228	228	179	185	210
210	154	154	128	130	166	168	191	191	190	192
251	253	218	218	139	145	169	169			
NSDL06	NSDL	-64.40985278		44.50441944		226	226	175	185	176
208	154	154	130	132	166	166	187	191	188	192
253	255	214	216	139	145	165	167			
NSDL07	NSDL	-64.40985278		44.50441944		228	228	183	185	206
210	138	138	130	132	166	166	191	191	180	194
251	253	216	220	137	145	167	171			
NSDL08	NSDL	-64.40985278		44.50441944		226	228	181	181	186
208	138	152	130	130	166	166	189	189	192	198
251	253	214	216	139	145	165	167			
NSDL09	NSDL	-64.40985278		44.50441944		236	236	183	183	208
208	138	138	126	126	166	166	189	191	196	198
251	255	216	216	139	145	167	167			
NSDL10	NSDL	-64.40985278		44.50441944		228	230	185	185	206
208	138	180	126	140	166	166	191	191	196	198
253	253	214	214	139	145	165	165			
NSDL11	NSDL	-64.40985278		44.50441944		228	228	183	187	190
212	142	152	128	128	166	184	187	191	198	204
253	255	214	214	139	145	165	165			
NSDL12	NSDL	-64.40985278		44.50441944		228	230	183	187	202

202	138	138	128	128	166	166	187	191	194	198
253	255	214	216	139	145	165	167			
NSDL13	NSDL	-64.40985278		44.50441944		228	230	185	185	208
210	138	138	124	130	166	166	191	191	190	196
251	253	194	214	139	145	165	165			
NSDL14	NSDL	-64.40985278		44.50441944		228	228	183	183	206
208	148	148	126	144	166	166	189	189	188	192
253	255	216	216	135	145	167	167			
NSDL15	NSDL	-64.40985278		44.50441944		228	230	181	181	186
186	136	140	128	128	166	166	187	191	186	190
251	253	214	216	139	145	165	167			
NSDL16	NSDL	-64.40985278		44.50441944		228	230	183	183	190
190	138	154	124	126	164	166	189	191	186	188
253	253	212	214	139	145	163	165			
NSDL17	NSDL	-64.40985278		44.50441944		228	230	183	183	182
210	136	154	126	126	166	166	187	189	190	190
255	255	214	214	139	145	165	165			
NSDL18	NSDL	-64.40985278		44.50441944		226	226	185	189	206
210	142	142	126	128	164	164	189	193	190	194
255	255	214	216	135	141	165	167			
NSDL19	NSDL	-64.40985278		44.50441944		228	234	179	185	190
190	140	140	126	128	166	166	189	189	184	190
253	255	214	216	139	145	165	167			
NSDL20	NSDL	-64.40985278		44.50441944		228	238	183	183	202
210	140	146	144	144	166	166	189	189	190	198
253	255	212	216	139	145	163	167			
NSDL21	NSDL	-64.40985278		44.50441944		230	234	183	183	204
206	140	142	110	130	166	166	189	199	190	194
253	253	212	216	139	145	163	167			
NSDL22	NSDL	-64.40985278		44.50441944		230	234	183	183	206
206	140	140	126	126	166	166	189	199	188	194
251	253	214	214	139	145	165	165			
NSDL23	NSDL	-64.40985278		44.50441944		228	232	181	181	206
206	140	140	126	128	166	166	191	191	182	190
251	253	214	216	135	145	165	167			
NSDL24	NSDL	-64.40985278		44.50441944		228	228	167	181	182
210	138	154	130	132	168	168	189	191	188	192
251	253	214	214	139	145	165	165			
NSDL25	NSDL	-64.40985278		44.50441944		226	230	187	189	190
190	148	152	126	130	168	168	189	193	196	198
251	253	212	216	139	145	163	167			
NSDL26	NSDL	-64.40985278		44.50441944		228	230	183	183	184
188	140	152	126	130	166	168	189	189	190	196
247	255	214	216	139	145	165	167			
NSDL27	NSDL	-64.40985278		44.50441944		228	230	183	187	192
212	140	148	124	130	166	166	189	189	190	194
251	253	214	214	139	141	165	165			
NSDL28	NSDL	-64.40985278		44.50441944		228	230	183	183	210
210	140	140	124	126	166	166	187	189	190	194
251	253	214	216	139	141	165	167			
NSDL29	NSDL	-64.40985278		44.50441944		228	230	185	187	210
210	140	140	124	126	166	166	187	189	188	192
253	253	214	214	139	145	165	165			
NSDL30	NSDL	-64.40985278		44.50441944		226	230	183	183	192
212	144	152	124	128	166	166	189	191	188	188
253	253	214	216	139	145	165	167			
NSDL31	NSDL	-64.40985278		44.50441944		228	228	185	189	210
210	140	154	110	130	166	184	189	189	190	194
251	253	214	216	139	145	165	167			
NSDL32	NSDL	-64.40985278		44.50441944		226	238	185	189	206
210	140	140	122	122	170	170	189	199	190	194
251	253	214	214	137	145	165	165			
NSDL33	NSDL	-64.40985278		44.50441944		226	228	181	181	206

206	140	142	128	128	166	166	191	191	190	194
251	253	214	216	139	145	165	167			
NSDL34	NSDL	-64.40985278		44.50441944		226	228	183	183	212
212	140	140	126	126	166	166	189	189	192	198
253	261	214	216	139	145	165	167			
NSDL35	NSDL	-64.40985278		44.50441944		240	242	181	185	208
208	140	140	124	126	166	166	189	191	194	196
253	253	216	216	139	145	167	167			
NSDL36	NSDL	-64.40985278		44.50441944		240	242	183	187	204
204	140	142	130	130	166	166	191	191	192	194
253	255	216	216	139	145	167	167			
NSDL37	NSDL	-64.40985278		44.50441944		240	242	183	187	208
214	140	142	128	130	166	166	191	191	190	194
253	253	214	218	135	145	165	169			
NSDL38	NSDL	-64.40985278		44.50441944		240	242	183	183	212
212	140	140	138	138	166	166	189	189	192	194
251	253	216	218	139	145	167	169			
NSDL39	NSDL	-64.40985278		44.50441944		240	242	183	187	192
210	140	140	126	128	166	166	189	189	190	196
251	253	214	218	139	145	165	169			
NSDL40	NSDL	-64.40985278		44.50441944		240	242	183	187	210
212	140	152	130	130	166	166	189	189	188	192
253	253	216	222	137	145	167	173			
NSDL41	NSDL	-64.40985278		44.50441944		228	228	183	187	204
204	140	140	122	122	166	166	189	189	194	198
251	253	214	216	139	145	165	167			
NSDL42	NSDL	-64.40985278		44.50441944		226	230	185	185	208
212	144	154	194	194	166	166	191	193	192	196
253	255	216	216	137	145	167	167			
NSDL43	NSDL	-64.40985278		44.50441944		228	232	183	185	194
210	140	150	128	130	166	166	191	191	192	196
251	253	214	218	139	145	165	169			
NSDL44	NSDL	-64.40985278		44.50441944		228	230	183	183	190
214	138	140	124	126	166	166	189	191	188	204
253	255	216	216	139	145	167	167			
NSDL45	NSDL	-64.40985278		44.50441944		228	228	183	185	212
212	140	146	126	128	166	166	193	193	192	196
251	255	216	216	139	145	167	167			
NSDL46	NSDL	-64.40985278		44.50441944		228	228	185	191	220
220	140	140	132	132	166	166	191	191	200	204
253	255	216	216	139	145	167	167			
NSDL47	NSDL	-64.40985278		44.50441944		228	228	181	181	212
212	142	154	132	132	164	164	191	193	190	194
253	255	218	218	139	145	169	169			
NSDL48	NSDL	-64.40985278		44.50441944		228	228	181	185	204
210	140	140	124	124	166	166	191	193	190	194
251	253	216	216	137	145	167	167			
NSDL49	NSDL	-64.40985278		44.50441944		228	230	179	183	204
204	142	140	132	132	166	166	191	191	192	198
253	255	218	218	139	145	169	169			
NSDL50	NSDL	-64.40985278		44.50441944		228	230	181	181	192
208	140	140	128	128	168	168	191	191	192	194
255	257	216	216	139	145	167	167			
NSUM01	NSUM	-63.60874167		44.95438056		230	236	187	199	210
210	160	160	130	134	182	182	191	191	190	192
251	251	214	216	137	145	165	167			
NSUM02	NSUM	-63.60874167		44.95438056		230	230	187	199	200
210	158	162	130	134	182	182	187	187	190	190
251	251	214	216	139	145	165	167			
NSUM03	NSUM	-63.60874167		44.95438056		230	230	181	183	206
210	160	176	130	132	182	182	187	187	188	198
251	267	214	214	139	145	165	165			
NSUM04	NSUM	-63.60874167		44.95438056		230	230	181	185	202

204	160	176	132	136	182	184	191	191	188	190
251	269	214	214	139	145	165	165			
NSUM05	NSUM	-63.60874167		44.95438056		230	230	181	183	210
210	162	162	130	134	180	180	191	191	190	190
251	251	212	214	139	145	163	165			
NSUM06	NSUM	-63.60874167		44.95438056		230	234	179	183	206
206	160	172	128	130	180	180	187	189	188	190
251	251	212	214	139	145	163	165			
NSUM07	NSUM	-63.60874167		44.95438056		230	230	181	183	190
216	158	160	128	132	182	182	187	187	188	194
251	267	212	214	139	145	163	165			
NSUM08	NSUM	-63.60874167		44.95438056		230	232	181	183	200
204	156	160	130	132	180	182	187	187	190	194
251	267	214	214	135	145	165	165			
NSUM09	NSUM	-63.60874167		44.95438056		230	230	183	185	194
202	156	160	128	134	180	182	187	187	188	188
251	251	212	214	137	145	163	165			
NSUM10	NSUM	-63.60874167		44.95438056		232	232	183	185	206
206	158	158	128	134	182	182	189	189	190	190
253	267	212	214	135	145	163	165			
NSUM11	NSUM	-63.60874167		44.95438056		232	232	183	185	194
216	160	176	126	130	180	180	187	187	194	194
253	253	212	214	135	145	163	165			
NSUM12	NSUM	-63.60874167		44.95438056		232	232	183	183	194
216	160	160	128	132	182	182	187	189	190	198
253	253	210	214	139	145	161	165			
NSUM13	NSUM	-63.60874167		44.95438056		232	232	181	183	190
218	176	176	130	130	180	180	189	189	188	188
253	271	214	214	139	145	165	165			
NSUM14	NSUM	-63.60874167		44.95438056		232	238	183	185	196
196	160	176	130	134	178	180	189	199	196	198
253	253	212	214	139	145	163	165			
NSUM15	NSUM	-63.60874167		44.95438056		232	232	183	185	206
206	160	160	132	136	180	180	189	199	188	198
253	269	210	214	137	145	161	165			
NSUM16	NSUM	-63.60874167		44.95438056		230	232	183	185	208
210	160	160	132	134	180	180	187	189	190	198
255	271	214	216	139	145	165	167			
NSUM17	NSUM	-63.60874167		44.95438056		232	232	183	185	208
210	160	174	132	134	178	178	185	199	188	196
255	255	212	216	139	145	163	167			
NSUM18	NSUM	-63.60874167		44.95438056		232	238	181	183	208
208	158	174	132	134	176	178	185	201	188	192
255	255	212	214	139	145	163	165			
NSUM19	NSUM	-63.60874167		44.95438056		228	232	185	185	188
210	158	160	134	134	176	178	185	185	192	198
255	255	214	216	139	145	165	167			
NSUM20	NSUM	-63.60874167		44.95438056		222	232	183	183	174
206	160	174	132	134	178	180	185	185	194	200
255	255	212	214	139	145	163	165			
NSUM21	NSUM	-63.60874167		44.95438056		234	234	183	185	206
210	160	160	130	130	176	178	185	199	188	194
255	255	214	214	137	145	165	165			
NSUM22	NSUM	-63.60874167		44.95438056		232	232	185	185	206
210	158	158	130	130	178	178	185	185	188	188
255	271	214	214	139	145	165	165			
NSUM23	NSUM	-63.60874167		44.95438056		232	232	183	183	206
206	158	158	128	130	154	178	155	187	188	192
255	271	214	216	139	145	165	167			
NSUM24	NSUM	-63.60874167		44.95438056		232	232	165	173	206
206	158	158	130	132	178	180	185	187	190	192
255	271	214	214	137	145	165	165			
NSUM25	NSUM	-63.60874167		44.95438056		232	232	183	183	210

210	160	160	134	134	178	178	185	185	188	198
253	253	214	214	139	145	165	165			
NSUM26	NSUM	-63.60874167		44.95438056		232	236	181	183	190
206	160	160	134	140	156	178	187	189	194	196
253	253	214	214	139	145	165	165			
NSUM27	NSUM	-63.60874167		44.95438056		228	234	181	185	176
190	160	160	130	130	170	180	185	189	198	202
253	253	214	214	139	145	165	165			
NSUM28	NSUM	-63.60874167		44.95438056		230	232	181	183	206
212	160	162	130	130	162	174	189	189	192	206
253	253	214	216	139	145	165	167			
NSUM29	NSUM	-63.60874167		44.95438056		230	232	185	187	178
214	160	164	136	136	156	178	161	187	198	200
253	253	214	214	137	145	165	165			
NSUM30	NSUM	-63.60874167		44.95438056		230	232	183	187	210
210	162	178	138	138	176	180	155	187	194	198
253	253	210	214	139	145	161	165			
NSUM31	NSUM	-63.60874167		44.95438056		230	230	147	147	208
212	158	174	128	128	180	180	185	185	186	188
253	253	214	214	139	145	165	165			
NSUM32	NSUM	-63.60874167		44.95438056		228	232	183	189	190
212	140	160	136	136	178	180	161	187	196	206
253	269	214	214	139	145	165	165			
NSUM33	NSUM	-63.60874167		44.95438056		228	232	169	183	208
208	158	176	136	136	180	182	185	199	196	198
253	253	212	216	141	145	163	167			
NSUM34	NSUM	-63.60874167		44.95438056		230	230	183	185	204
210	158	158	136	136	178	178	185	189	188	198
253	269	212	214	139	145	163	165			
NSUM35	NSUM	-63.60874167		44.95438056		230	230	183	183	206
212	160	160	132	132	178	178	185	185	200	200
251	251	212	214	139	145	163	165			
NSUM36	NSUM	-63.60874167		44.95438056		230	230	183	185	210
210	158	158	136	156	180	180	185	189	188	198
253	267	214	216	139	145	165	167			
NSUM37	NSUM	-63.60874167		44.95438056		230	230	183	187	210
210	160	164	156	156	180	180	185	189	194	194
251	267	214	214	135	145	165	165			
NSUM38	NSUM	-63.60874167		44.95438056		228	232	183	185	208
210	158	174	130	132	180	180	189	189	190	198
253	269	212	214	139	145	163	165			
NSUM39	NSUM	-63.60874167		44.95438056		230	230	183	185	208
210	156	160	130	132	182	182	185	185	196	196
251	251	212	214	139	145	163	165			
NSUM40	NSUM	-63.60874167		44.95438056		230	230	183	183	190
190	158	174	130	134	178	178	183	185	190	194
253	267	214	214	139	145	165	165			
NSUM41	NSUM	-63.60874167		44.95438056		230	232	183	183	190
210	158	174	132	136	178	182	183	185	188	198
253	267	214	216	137	141	165	167			
NSUM42	NSUM	-63.60874167		44.95438056		230	232	185	185	198
210	158	160	136	138	170	180	185	187	188	192
253	267	214	216	139	145	165	167			
NSUM43	NSUM	-63.60874167		44.95438056		230	232	183	187	198
210	158	174	128	128	180	180	187	199	188	190
253	267	214	214	139	145	165	165			
NSUM44	NSUM	-63.60874167		44.95438056		230	230	183	197	190
210	158	158	128	128	180	180	185	199	190	190
253	267	214	214	139	145	165	165			
NSUM45	NSUM	-63.60874167		44.95438056		228	230	183	197	208
210	160	168	134	136	180	180	187	189	190	198
253	267	214	214	139	145	165	165			
NSUM46	NSUM	-63.60874167		44.95438056		224	230	185	197	210

210	160	172	136	136	182	182	187	189	190	190
251	251	214	216	139	141	165	167			
NSUM47	NSUM	-63.60874167		44.95438056		230	230	183	185	208
218	160	160	130	134	182	182	183	187	180	188
253	253	214	214	139	145	165	165			
NSUM48	NSUM	-63.60874167		44.95438056		228	234	181	197	208
208	160	162	132	132	182	182	185	187	194	194
253	267	214	216	139	145	165	167			
NSUM49	NSUM	-63.60874167		44.95438056		228	230	183	183	204
208	158	174	132	134	174	182	185	187	180	188
253	267	214	214	139	145	165	165			
NSUM50	NSUM	-63.60874167		44.95438056		230	230	183	183	208
208	158	174	126	128	182	182	185	187	192	198
253	267	214	216	137	141	165	167			
PQCT01	PQCT	-70.80452222		47.07675556		228	228	183	183	204
204	158	158	132	132	192	192	189	191	188	188
255	257	214	216	139	145	165	167			
PQCT02	PQCT	-70.80452222		47.07675556		228	228	181	183	194
206	158	158	136	136	192	192	189	191	190	192
257	257	214	214	139	145	165	165			
PQCT03	PQCT	-70.80452222		47.07675556		232	232	183	185	188
204	158	158	130	130	192	192	189	191	190	190
255	273	214	216	139	145	165	167			
PQCT04	PQCT	-70.80452222		47.07675556		228	232	183	183	170
188	160	160	132	132	190	194	189	189	190	198
255	257	210	216	137	143	161	167			
PQCT05	PQCT	-70.80452222		47.07675556		222	228	185	185	194
206	174	174	132	132	188	190	189	191	194	198
255	271	212	216	137	145	163	167			
PQCT06	PQCT	-70.80452222		47.07675556		228	230	183	185	204
206	164	174	128	128	190	190	177	191	202	202
255	273	214	216	137	145	165	167			
PQCT07	PQCT	-70.80452222		47.07675556		228	230	181	181	202
210	170	170	128	130	194	194	191	193	192	198
255	257	214	216	141	145	165	167			
PQCT08	PQCT	-70.80452222		47.07675556		226	230	185	187	194
206	158	172	128	130	184	190	175	191	186	188
257	271	206	216	141	145	157	167			
PQCT09	PQCT	-70.80452222		47.07675556		228	230	169	185	188
206	158	172	128	132	190	190	189	191	186	188
257	255	212	216	141	145	163	167			
PQCT10	PQCT	-70.80452222		47.07675556		228	232	185	187	204
208	158	158	132	132	192	192	191	191	188	192
255	273	214	214	141	141	165	165			
PQCT11	PQCT	-70.80452222		47.07675556		234	234	183	183	204
204	176	176	134	134	188	190	175	191	188	190
255	255	214	214	141	145	165	165			
PQCT12	PQCT	-70.80452222		47.07675556		228	230	183	187	204
208	182	182	130	130	186	190	177	191	198	204
269	273	212	214	141	145	163	165			
PQCT13	PQCT	-70.80452222		47.07675556		228	232	185	187	190
200	158	176	130	134	190	190	189	195	188	200
269	269	214	214	139	145	165	165			
PQCT14	PQCT	-70.80452222		47.07675556		228	228	183	185	206
206	158	160	130	134	190	190	189	193	190	190
253	253	214	218	139	145	165	169			
PQCT15	PQCT	-70.80452222		47.07675556		228	228	171	183	182
184	158	162	130	130	188	188	179	193	188	198
253	257	212	214	139	145	163	165			
PQCT16	PQCT	-70.80452222		47.07675556		228	228	183	187	190
206	160	176	132	136	190	190	193	193	192	200
253	271	212	216	141	145	163	167			
PQCT17	PQCT	-70.80452222		47.07675556		228	228	183	183	202

206	174	174	132	136	190	190	191	199	188	194
257	269	212	218	141	145	163	169			
PQCT18	PQCT	-70.80452222		47.07675556		228	228	183	185	204
204	162	176	136	136	186	188	191	193	190	190
267	271	214	218	139	145	165	169			
PQCT19	PQCT	-70.80452222		47.07675556		228	228	185	191	192
192	176	176	134	134	186	188	191	191	196	196
251	251	214	216	139	145	165	167			
PQCT20	PQCT	-70.80452222		47.07675556		228	228	183	185	194
208	160	176	134	134	188	188	189	193	190	200
269	271	212	216	135	145	163	167			
PQCT21	PQCT	-70.80452222		47.07675556		226	228	181	185	194
210	160	160	134	134	190	192	189	191	192	194
257	269	212	216	139	145	163	167			
PQCT22	PQCT	-70.80452222		47.07675556		228	228	183	183	194
208	158	158	130	134	186	188	177	185	188	190
255	255	214	214	141	145	165	165			
PQCT23	PQCT	-70.80452222		47.07675556		222	228	183	183	194
194	150	150	134	134	188	188	187	191	198	198
253	277	212	218	141	145	163	169			
PQCT24	PQCT	-70.80452222		47.07675556		228	228	183	185	198
202	158	160	140	140	188	188	189	193	188	200
253	253	214	216	141	141	165	167			
PQCT25	PQCT	-70.80452222		47.07675556		226	228	183	185	188
188	144	160	132	132	188	222	177	187	190	190
255	257	214	214	141	145	165	165			
PQCT26	PQCT	-70.80452222		47.07675556		226	230	183	185	204
204	160	160	132	140	188	188	189	195	192	198
253	255	214	214	139	141	165	165			
PQCT27	PQCT	-70.80452222		47.07675556		226	228	169	183	180
206	176	176	130	134	188	214	177	185	190	198
253	271	216	216	139	145	167	167			
PQCT28	PQCT	-70.80452222		47.07675556		228	230	183	185	204
206	160	160	136	136	188	188	189	193	192	192
253	253	214	214	141	145	165	165			
PQCT29	PQCT	-70.80452222		47.07675556		226	230	181	181	206
208	176	176	134	136	190	190	187	191	188	200
253	257	214	214	141	145	165	165			
PQCT30	PQCT	-70.80452222		47.07675556		228	230	169	185	204
204	174	174	136	136	186	190	185	193	188	200
257	253	214	214	139	145	165	165			
PQCT31	PQCT	-70.80452222		47.07675556		228	228	181	183	202
206	158	164	134	134	192	192	185	193	188	190
253	253	214	216	139	145	165	167			
PQCT32	PQCT	-70.80452222		47.07675556		228	228	181	185	200
200	174	174	130	130	186	190	187	193	190	198
255	257	212	218	139	145	163	169			
PQCT33	PQCT	-70.80452222		47.07675556		228	228	183	185	206
206	158	158	132	132	186	186	189	189	190	190
253	253	214	216	139	145	165	167			
PQCT34	PQCT	-70.80452222		47.07675556		228	234	183	185	206
206	158	172	130	130	192	192	189	191	192	192
253	257	214	218	141	145	165	169			
PQCT35	PQCT	-70.80452222		47.07675556		228	234	181	183	206
208	144	158	128	128	190	190	189	191	192	194
253	253	214	218	139	145	165	169			
PQCT36	PQCT	-70.80452222		47.07675556		228	228	169	185	206
206	168	172	134	134	186	190	189	199	188	190
253	261	214	216	141	145	165	167			
PQCT37	PQCT	-70.80452222		47.07675556		226	228	181	183	204
208	162	162	128	128	186	190	191	191	198	198
253	255	214	216	141	145	165	167			
PQCT38	PQCT	-70.80452222		47.07675556		226	228	183	185	202

```
204	160	174	134	134	188	190	191	191	188	198
255	271	214	216	141	145	165	167
PQCT39	PQCT	-70.80452222	47.07675556	226	228	183	185	202
206	158	158	130	130	190	190	189	193	188	200
255	255	214	226	141	145	165	177
PQCT40	PQCT	-70.80452222	47.07675556	226	228	185	185	206
206	158	158	134	134	218	218	191	201	188	190
255	273	216	218	139	145	167	169
PQCT41	PQCT	-70.80452222	47.07675556	228	228	169	181	202
208	158	158	130	130	186	190	189	191	194	200
255	269	216	220	139	145	167	171
PQCT42	PQCT	-70.80452222	47.07675556	228	228	185	185	188
200	174	174	138	138	190	190	189	191	204	204
255	255	218	222	139	145	169	173
PQCT43	PQCT	-70.80452222	47.07675556	228	228	183	183	206
208	158	178	130	130	188	192	189	191	198	200
255	255	216	222	139	145	167	173
PQCT44	PQCT	-70.80452222	47.07675556	228	228	185	185	188
198	158	178	132	132	190	190	191	193	198	200
255	271	218	222	139	145	169	173
PQCT45	PQCT	-70.80452222	47.07675556	228	228	185	185	204
206	168	168	132	132	190	190	191	191	198	200
255	269	216	218	139	145	167	169
PQCT46	PQCT	-70.80452222	47.07675556	228	228	185	185	202
206	170	174	132	134	190	190	191	191	198	200
255	255	216	220	139	145	167	171
PQCT47	PQCT	-70.80452222	47.07675556	228	228	185	185	202
202	170	170	132	134	190	190	189	189	198	200
255	261	214	216	141	145	165	167
PQCT48	PQCT	-70.80452222	47.07675556	228	228	185	185	206
206	172	172	130	130	190	190	191	191	198	200
255	261	212	216	141	145	163	167
PQCT49	PQCT	-70.80452222	47.07675556	228	228	185	185	204
204	172	172	132	132	190	190	191	193	198	200
255	261	214	216	139	145	165	167
PQCT50	PQCT	-70.80452222	47.07675556	228	228	185	185	204
204	172	172	140	140	190	190	195	195	198	200
255	261	214	216	139	145	165	167
PQSR01	PQSR	-71.01358333	46.01428889	228	230	183	183	206
206	164	176	132	132	150	150	195	195	186	190
255	257	214	214	139	141	165	165
PQSR02	PQSR	-71.01358333	46.01428889	226	228	183	183	196
208	162	170	132	132	162	168	195	195	186	190
257	259	214	214	139	141	165	165
PQSR03	PQSR	-71.01358333	46.01428889	226	230	183	183	190
206	176	176	132	132	156	170	185	191	184	192
255	259	212	212	139	145	163	163
PQSR04	PQSR	-71.01358333	46.01428889	228	232	185	185	172
190	156	176	128	130	156	166	189	195	190	198
255	271	212	216	137	143	163	167
PQSR05	PQSR	-71.01358333	46.01428889	226	230	185	185	196
208	160	170	130	132	164	168	191	191	190	198
255	259	214	214	139	145	165	165
PQSR06	PQSR	-71.01358333	46.01428889	228	232	183	189	206
208	160	176	132	136	166	168	191	191	184	192
255	259	214	214	141	145	165	165
PQSR07	PQSR	-71.01358333	46.01428889	228	232	183	183	204
212	160	170	132	132	168	168	191	197	184	192
255	257	214	214	141	145	165	165
PQSR08	PQSR	-71.01358333	46.01428889	230	230	183	183	196
208	174	176	132	132	156	168	189	189	186	194
253	261	214	214	141	145	165	165
PQSR09	PQSR	-71.01358333	46.01428889	228	232	183	183	190
```

208	160	160	134	134	166	166	189	191	186	192
257	273	214	214	141	145	165	165			
PQSR10	PQSR	-71.01358333		46.01428889		226	232	183	183	206
210	162	176	132	136	166	166	189	189	188	196
255	259	214	214	139	141	165	165			
PQSR11	PQSR	-71.01358333		46.01428889		230	230	185	185	206
206	160	176	132	132	158	166	189	189	190	196
257	273	214	214	141	145	165	165			
PQSR12	PQSR	-71.01358333		46.01428889		230	230	183	183	206
210	160	160	134	134	166	166	189	191	190	190
255	259	214	214	141	145	165	165			
PQSR13	PQSR	-71.01358333		46.01428889		228	230	183	183	192
202	160	160	136	144	166	166	191	195	192	192
257	257	214	214	139	145	165	165			
PQSR14	PQSR	-71.01358333		46.01428889		228	230	183	185	208
208	160	174	136	144	166	168	193	193	192	198
255	259	214	216	139	145	165	167			
PQSR15	PQSR	-71.01358333		46.01428889		230	230	183	183	184
186	148	160	134	136	164	166	189	191	188	190
257	293	214	214	139	145	165	165			
PQSR16	PQSR	-71.01358333		46.01428889		228	232	183	183	192
208	160	162	134	136	166	166	191	191	196	196
255	255	214	214	141	141	165	165			
PQSR17	PQSR	-71.01358333		46.01428889		228	230	185	185	204
208	160	160	134	136	166	166	193	193	200	204
255	261	214	216	141	141	165	167			
PQSR18	PQSR	-71.01358333		46.01428889		228	230	185	185	206
206	160	176	132	134	154	166	187	193	188	190
255	267	214	216	139	145	165	167			
PQSR19	PQSR	-71.01358333		46.01428889		228	228	185	187	194
194	136	140	126	134	166	166	191	191	188	198
255	259	214	214	139	145	165	165			
PQSR20	PQSR	-71.01358333		46.01428889		228	230	169	183	196
210	158	160	130	134	166	166	191	193	188	198
257	263	214	214	135	145	165	165			
PQSR21	PQSR	-71.01358333		46.01428889		230	230	183	185	196
212	164	176	132	136	166	166	191	193	160	192
255	259	214	214	139	145	165	165			
PQSR22	PQSR	-71.01358333		46.01428889		228	230	181	203	196
210	174	176	130	134	156	168	189	189	198	198
255	259	214	216	141	145	165	167			
PQSR23	PQSR	-71.01358333		46.01428889		228	230	173	203	196
210	160	174	134	134	166	166	191	191	198	198
257	257	214	216	141	145	165	167			
PQSR24	PQSR	-71.01358333		46.01428889		226	230	183	185	200
204	162	162	130	130	166	166	197	197	192	202
255	255	216	216	139	141	167	167			
PQSR25	PQSR	-71.01358333		46.01428889		226	228	183	183	190
190	150	152	130	130	170	180	201	203	NA	NA
255	255	214	214	141	145	165	165			
PQSR26	PQSR	-71.01358333		46.01428889		228	230	183	183	206
206	160	162	132	134	170	180	197	197	188	194
253	253	214	216	139	141	165	167			
PQSR27	PQSR	-71.01358333		46.01428889		228	228	185	185	182
208	160	176	132	132	170	178	191	191	196	200
253	253	214	214	139	145	165	165			
PQSR28	PQSR	-71.01358333		46.01428889		228	230	183	183	206
208	162	162	134	134	176	180	191	195	192	200
253	253	216	216	141	145	167	167			
PQSR29	PQSR	-71.01358333		46.01428889		226	232	183	183	208
210	160	160	130	130	156	168	189	189	164	208
253	253	216	216	139	145	167	167			
PQSR30	PQSR	-71.01358333		46.01428889		228	230	183	183	206

206	162	176	132	136	168	180	191	193	168	204
253	271	216	216	141	145	167	167			
PQSR31	PQSR	-71.01358333		46.01428889		226	230	185	185	204
208	164	164	132	132	168	172	191	207	166	200
255	269	216	216	141	145	167	167			
PQSR32	PQSR	-71.01358333		46.01428889		228	230	183	185	202
202	160	162	132	134	168	168	201	205	166	192
267	271	214	216	139	145	165	167			
PQSR33	PQSR	-71.01358333		46.01428889		214	228	181	183	208
208	148	160	130	136	164	166	191	191	164	194
251	251	212	214	139	145	163	165			
PQSR34	PQSR	-71.01358333		46.01428889		228	228	183	183	208
208	176	176	130	130	166	166	191	191	162	192
255	271	214	216	141	143	165	167			
PQSR35	PQSR	-71.01358333		46.01428889		226	228	183	183	208
210	160	170	132	134	150	150	191	191	162	192
251	269	214	214	139	145	165	165			
PQSR36	PQSR	-71.01358333		46.01428889		226	230	183	185	208
208	160	162	132	132	166	166	201	201	160	194
255	255	214	214	141	145	165	165			
PQSR37	PQSR	-71.01358333		46.01428889		226	228	185	185	206
210	160	162	130	132	168	168	191	201	160	194
253	277	214	214	141	143	165	165			
PQSR38	PQSR	-71.01358333		46.01428889		228	230	183	183	204
206	162	176	108	134	166	168	191	193	160	192
253	253	214	214	141	145	165	165			
PQSR39	PQSR	-71.01358333		46.01428889		226	230	183	185	204
208	166	176	134	134	166	166	193	193	162	194
253	253	214	216	141	141	165	167			
PQSR40	PQSR	-71.01358333		46.01428889		228	230	181	187	208
208	174	176	142	142	166	166	191	191	194	194
253	255	214	216	139	145	165	167			
PQSR41	PQSR	-71.01358333		46.01428889		228	228	183	183	204
210	160	162	134	134	166	166	193	193	160	194
253	253	214	214	139	145	165	165			
PQSR42	PQSR	-71.01358333		46.01428889		228	228	183	183	190
202	166	170	134	142	166	168	191	191	160	192
253	253	214	216	139	145	165	167			
PQSR43	PQSR	-71.01358333		46.01428889		228	228	183	183	208
210	160	162	136	136	166	166	191	191	194	200
253	257	214	214	139	145	165	165			
PQSR44	PQSR	-71.01358333		46.01428889		228	234	183	183	190
200	162	166	132	132	166	166	191	193	164	192
253	253	214	216	139	145	165	167			
PQSR45	PQSR	-71.01358333		46.01428889		228	234	183	183	206
208	160	178	134	134	166	166	191	191	194	208
253	253	216	216	139	145	167	167			
PQSR46	PQSR	-71.01358333		46.01428889		228	228	185	185	204
208	160	162	132	132	166	166	191	191	190	196
255	257	216	216	139	145	167	167			
PQSR47	PQSR	-71.01358333		46.01428889		214	228	181	183	204
204	160	178	132	132	144	166	193	201	190	190
253	253	218	216	141	145	169	167			
PQSR48	PQSR	-71.01358333		46.01428889		228	228	183	187	208
208	158	160	130	132	166	166	191	191	186	190
253	253	214	214	141	145	165	165			
PQSR49	PQSR	-71.01358333		46.01428889		232	232	183	183	206
206	162	162	132	132	144	166	191	191	186	190
253	255	214	214	139	145	165	165			
PQSR50	PQSR	-71.01358333		46.01428889		226	226	183	183	206
206	166	178	140	140	168	168	191	191	190	192
253	261	214	214	139	145	165	165			
PQSS01	PQSS	-72.28671667		46.64313333		228	228	181	183	206

210	146	162	130	130	156	166	191	199	188	194
257	257	214	214	139	145	165	165			
PQSS02	PQSS	-72.28671667		46.64313333		228	228	181	181	172
210	148	156	130	130	158	162	187	193	194	198
257	257	214	216	139	145	165	167			
PQSS03	PQSS	-72.28671667		46.64313333		234	234	181	183	190
214	144	158	126	132	154	164	187	195	186	188
255	273	214	214	139	145	165	165			
PQSS04	PQSS	-72.28671667		46.64313333		216	216	181	181	182
212	144	168	130	132	160	164	193	199	188	190
257	257	216	216	137	143	167	167			
PQSS05	PQSS	-72.28671667		46.64313333		222	228	183	191	192
208	154	154	130	132	162	166	191	197	196	198
255	255	214	216	137	145	165	167			
PQSS06	PQSS	-72.28671667		46.64313333		228	228	183	187	208
210	156	156	130	132	154	164	193	195	190	206
255	273	212	214	137	145	163	165			
PQSS07	PQSS	-72.28671667		46.64313333		228	228	175	185	212
212	152	162	120	134	164	164	187	193	186	190
255	255	212	216	141	145	163	167			
PQSS08	PQSS	-72.28671667		46.64313333		226	228	183	183	208
210	154	160	120	130	160	166	187	193	190	194
255	271	212	214	141	145	163	165			
PQSS09	PQSS	-72.28671667		46.64313333		228	228	183	183	206
210	142	162	132	132	154	164	193	203	190	194
255	255	212	214	141	145	163	165			
PQSS10	PQSS	-72.28671667		46.64313333		228	234	175	185	210
214	158	162	132	132	162	164	187	193	188	200
255	255	212	214	141	141	163	165			
PQSS11	PQSS	-72.28671667		46.64313333		234	234	183	183	210
210	156	156	120	130	158	166	193	197	188	190
255	255	214	216	141	145	165	167			
PQSS12	PQSS	-72.28671667		46.64313333		228	228	183	185	194
194	144	158	130	132	158	158	191	193	188	192
255	273	214	214	141	145	165	165			
PQSS13	PQSS	-72.28671667		46.64313333		228	248	183	183	190
210	152	174	130	130	160	160	205	209	190	196
255	255	214	214	139	145	165	165			
PQSS14	PQSS	-72.28671667		46.64313333		228	228	183	183	210
210	144	154	128	132	156	166	191	195	190	194
255	255	214	214	139	145	165	165			
PQSS15	PQSS	-72.28671667		46.64313333		228	228	175	175	190
212	144	156	120	136	164	164	193	207	190	194
255	255	214	216	139	145	165	167			
PQSS16	PQSS	-72.28671667		46.64313333		228	228	183	185	196
198	142	176	130	130	154	166	193	193	190	202
255	255	214	216	141	145	165	167			
PQSS17	PQSS	-72.28671667		46.64313333		228	228	183	183	210
214	144	156	130	132	162	166	191	191	190	192
255	255	214	214	141	145	165	165			
PQSS18	PQSS	-72.28671667		46.64313333		228	228	183	183	198
202	150	150	120	134	160	166	195	207	174	194
255	259	214	216	139	145	165	167			
PQSS19	PQSS	-72.28671667		46.64313333		228	228	183	185	214
214	144	156	134	136	158	164	193	199	190	192
255	259	214	216	139	145	165	167			
PQSS20	PQSS	-72.28671667		46.64313333		228	228	175	185	176
208	142	174	130	130	164	166	189	193	190	196
255	255	216	216	135	145	167	167			
PQSS21	PQSS	-72.28671667		46.64313333		226	228	175	185	182
196	144	170	130	130	158	166	189	193	190	202
255	255	214	216	139	145	165	167			
PQSS22	PQSS	-72.28671667		46.64313333		228	228	183	185	208

212	146	164	120	134	160	166	189	193	190	200
255	255	214	214	141	145	165	165			
PQSS23	PQSS	-72.28671667		46.64313333		222	228	183	183	212
212	142	152	130	130	150	158	189	191	194	196
255	261	214	214	141	145	165	165			
PQSS24	PQSS	-72.28671667		46.64313333		228	228	183	183	192
214	146	158	120	130	154	164	189	191	190	194
255	273	214	216	141	141	165	167			
PQSS25	PQSS	-72.28671667		46.64313333		228	228	181	181	212
214	142	142	120	136	152	158	189	191	160	190
255	271	214	216	141	145	165	167			
PQSS26	PQSS	-72.28671667		46.64313333		226	228	181	187	176
212	142	142	120	136	154	164	187	189	202	206
255	261	216	216	139	141	167	167			
PQSS27	PQSS	-72.28671667		46.64313333		228	228	179	179	188
188	142	160	130	134	156	160	189	191	190	192
255	261	214	214	139	145	165	165			
PQSS28	PQSS	-72.28671667		46.64313333		228	228	181	181	212
212	144	156	130	134	152	160	191	195	192	200
255	273	214	214	141	145	165	165			
PQSS29	PQSS	-72.28671667		46.64313333		228	228	183	183	192
210	142	156	130	134	166	168	191	195	196	208
255	255	214	214	141	145	165	165			
PQSS30	PQSS	-72.28671667		46.64313333		228	228	181	185	192
206	142	148	128	128	152	160	191	193	196	206
255	255	214	214	139	145	165	165			
PQSS31	PQSS	-72.28671667		46.64313333		226	228	183	183	210
212	144	154	128	132	160	160	187	191	190	194
257	261	214	216	139	145	165	167			
PQSS32	PQSS	-72.28671667		46.64313333		226	228	181	183	192
192	146	164	132	132	152	158	189	193	192	202
257	275	212	214	139	145	163	165			
PQSS33	PQSS	-72.28671667		46.64313333		226	228	185	185	194
194	156	156	118	134	162	168	185	191	192	206
257	257	214	214	139	145	165	165			
PQSS34	PQSS	-72.28671667		46.64313333		226	228	183	183	170
190	144	156	120	132	164	166	189	191	190	192
257	257	214	214	141	145	165	165			
PQSS35	PQSS	-72.28671667		46.64313333		226	228	183	183	204
204	144	156	130	130	164	166	189	189	190	208
257	257	214	214	139	145	165	165			
PQSS36	PQSS	-72.28671667		46.64313333		226	228	183	183	194
202	142	156	130	130	158	168	189	191	192	194
255	255	214	214	141	145	165	165			
PQSS37	PQSS	-72.28671667		46.64313333		226	228	183	183	196
206	156	156	120	128	156	166	185	191	192	194
255	255	212	214	141	145	163	165			
PQSS38	PQSS	-72.28671667		46.64313333		226	228	183	183	196
200	142	156	130	130	158	166	189	189	192	196
255	255	212	214	141	145	163	165			
PQSS39	PQSS	-72.28671667		46.64313333		226	228	169	183	196
196	144	154	130	130	156	166	185	189	174	208
255	255	212	214	141	145	163	165			
PQSS40	PQSS	-72.28671667		46.64313333		226	228	183	183	186
208	144	156	130	130	156	166	185	189	190	202
255	273	212	214	139	145	163	165			
PQSS41	PQSS	-72.28671667		46.64313333		228	228	183	183	192
210	142	156	120	134	162	168	187	191	192	194
255	255	190	214	139	145	165	165			
PQSS42	PQSS	-72.28671667		46.64313333		228	228	185	187	196
212	154	154	130	130	158	166	189	191	192	202
255	255	216	216	139	145	167	167			
PQSS43	PQSS	-72.28671667		46.64313333		228	228	183	187	204

216	142	156	130	130	186	186	201	201	192	200
255	255	214	214	139	145	165	165			
PQSS44	PQSS	-72.28671667		46.64313333		228	228	183	183	196
200	144	158	130	130	164	170	187	191	192	200
255	271	216	216	139	145	167	167			
PQSS45	PQSS	-72.28671667		46.64313333		228	228	183	183	196
200	144	158	130	130	168	168	187	191	192	198
255	255	214	216	139	145	165	167			
PQSS46	PQSS	-72.28671667		46.64313333		228	228	175	183	200
206	142	172	130	130	168	168	185	189	188	196
255	255	214	214	139	145	165	165			
PQSS47	PQSS	-72.28671667		46.64313333		228	228	183	183	214
214	142	140	130	130	168	186	185	189	192	198
255	261	214	214	141	145	165	165			
PQSS48	PQSS	-72.28671667		46.64313333		228	228	175	175	194
202	146	146	130	130	168	186	191	191	192	196
255	261	212	214	141	145	163	165			
PQSS49	PQSS	-72.28671667		46.64313333		228	228	183	183	186
192	144	158	130	130	164	168	187	191	190	196
255	261	214	214	139	145	165	165			
PQSS50	PQSS	-72.28671667		46.64313333		228	228	175	175	204
214	154	156	130	130	160	166	193	197	194	196
255	261	212	214	139	145	163	165			
PQLP01	PQLP	-75.90966389		45.56239722		226	228	181	183	208
208	160	160	122	122	188	192	191	195	196	196
255	259	212	214	139	145	165	167			
PQLP02	PQLP	-75.90966389		45.56239722		228	234	183	183	198
210	176	178	130	136	188	188	189	191	190	202
255	275	212	212	141	141	165	165			
PQLP03	PQLP	-75.90966389		45.56239722		228	228	183	183	192
208	174	188	138	138	188	192	191	197	186	196
255	273	212	214	141	141	165	167			
PQLP04	PQLP	-75.90966389		45.56239722		226	230	179	181	174
192	160	170	130	130	188	192	189	191	188	198
253	257	212	212	141	145	165	165			
PQLP05	PQLP	-75.90966389		45.56239722		228	228	171	183	198
210	160	160	132	132	178	192	191	193	186	188
255	257	214	214	143	145	167	167			
PQLP06	PQLP	-75.90966389		45.56239722		228	228	173	183	208
210	162	162	136	140	188	188	191	191	190	192
255	271	212	212	139	145	165	165			
PQLP07	PQLP	-75.90966389		45.56239722		228	228	171	183	206
214	160	160	138	138	186	190	171	193	186	192
255	255	214	214	141	145	167	167			
PQLP08	PQLP	-75.90966389		45.56239722		226	230	173	183	198
210	176	176	140	140	180	180	191	193	186	188
255	259	212	214	139	145	165	167			
PQLP09	PQLP	-75.90966389		45.56239722		228	228	171	183	192
210	162	178	134	134	188	188	191	193	186	192
255	261	212	214	141	145	165	167			
PQLP10	PQLP	-75.90966389		45.56239722		228	228	183	183	208
212	162	178	134	138	188	188	191	191	188	188
253	257	214	214	139	145	167	167			
PQLP11	PQLP	-75.90966389		45.56239722		228	228	181	187	208
208	164	164	134	134	188	188	191	193	188	188
255	259	212	212	141	147	165	165			
PQLP12	PQLP	-75.90966389		45.56239722		228	230	181	189	208
212	176	176	130	130	188	188	191	193	196	204
255	259	214	214	139	145	167	167			
PQLP13	PQLP	-75.90966389		45.56239722		228	234	173	183	194
204	162	162	120	120	188	188	189	191	186	192
255	257	214	214	141	145	167	167			
PQLP14	PQLP	-75.90966389		45.56239722		228	228	171	183	210

210	166	176	130	134	188	192	189	191	188	190
257	257	212	212	141	145	165	165			
PQLP15	PQLP	-75.90966389		45.56239722		228	244	183	185	186
188	162	162	130	134	176	188	191	193	188	188
255	269	212	214	141	145	165	167			
PQLP16	PQLP	-75.90966389		45.56239722		226	228	183	187	194
210	172	176	128	128	188	188	189	191	186	188
257	273	212	212	139	145	165	165			
PQLP17	PQLP	-75.90966389		45.56239722		226	228	181	185	206
210	162	164	126	132	184	186	189	191	190	198
255	273	212	214	143	145	165	167			
PQLP18	PQLP	-75.90966389		45.56239722		212	228	185	191	208
208	164	176	126	132	184	188	199	201	186	188
257	263	214	214	143	145	167	167			
PQLP19	PQLP	-75.90966389		45.56239722		226	230	181	185	196
196	174	178	130	130	188	188	191	193	186	196
255	273	212	212	139	145	165	165			
PQLP20	PQLP	-75.90966389		45.56239722		228	234	181	185	198
212	174	178	130	130	188	188	191	193	186	196
253	275	212	214	141	145	165	167			
PQLP21	PQLP	-75.90966389		45.56239722		228	228	183	183	198
214	176	176	130	132	188	188	189	191	192	192
255	257	214	216	141	145	167	169			
PQLP22	PQLP	-75.90966389		45.56239722		226	228	183	185	198
212	162	162	130	130	188	188	189	191	186	188
253	261	NA	NA	143	147	NA	NA			
PQLP23	PQLP	-75.90966389		45.56239722		200	214	183	191	198
212	162	164	130	130	186	188	189	191	186	190
255	259	214	214	143	145	167	167			
PQLP24	PQLP	-75.90966389		45.56239722		226	228	181	185	202
206	162	162	136	136	186	188	191	193	186	196
253	257	214	214	141	145	167	167			
PQLP25	PQLP	-75.90966389		45.56239722		228	246	181	185	192
192	160	160	120	136	184	188	191	193	192	196
253	273	214	214	141	145	167	167			
PQLP26	PQLP	-75.90966389		45.56239722		228	230	183	183	208
208	160	188	120	136	188	188	191	193	186	196
257	273	214	214	141	145	167	167			
PQLP27	PQLP	-75.90966389		45.56239722		228	228	183	185	184
210	164	178	134	134	188	188	193	193	188	204
255	259	212	212	139	145	165	165			
PQLP28	PQLP	-75.90966389		45.56239722		230	230	183	185	208
210	176	176	130	130	188	188	191	193	158	188
255	255	212	214	141	145	165	167			
PQLP29	PQLP	-75.90966389		45.56239722		226	228	183	185	210
212	162	178	134	134	188	188	191	191	186	188
255	255	212	212	143	145	165	165			
PQLP30	PQLP	-75.90966389		45.56239722		228	228	181	185	208
208	162	162	134	134	184	188	189	191	196	202
257	257	214	214	139	145	167	167			
PQLP31	PQLP	-75.90966389		45.56239722		222	228	169	183	206
210	166	166	136	136	186	192	189	193	186	188
257	257	212	214	137	145	165	167			
PQLP32	PQLP	-75.90966389		45.56239722		228	234	169	183	204
204	172	176	138	140	188	190	193	195	186	188
257	273	212	214	139	145	165	167			
PQLP33	PQLP	-75.90966389		45.56239722		228	244	179	185	210
210	158	176	138	138	188	188	191	193	190	192
255	273	212	212	141	145	165	165			
PQLP34	PQLP	-75.90966389		45.56239722		228	228	169	183	210
210	160	184	134	134	188	188	189	191	194	204
255	273	212	214	141	145	165	167			
PQLP35	PQLP	-75.90966389		45.56239722		228	230	169	183	210

212	160	184	138	138	188	188	189	193	194	204
255	259	212	214	139	145	165	167			
PQLP36	PQLP	-75.90966389		45.56239722		228	228	185	185	210
210	162	178	132	136	188	188	191	191	190	202
255	259	212	216	143	145	165	169			
PQLP37	PQLP	-75.90966389		45.56239722		228	228	183	183	208
212	160	160	130	130	186	188	193	193	202	202
247	255	212	214	139	145	165	167			
PQLP38	PQLP	-75.90966389		45.56239722		228	228	185	185	206
208	160	186	130	130	188	188	189	191	192	204
247	257	212	212	143	145	165	165			
PQLP39	PQLP	-75.90966389		45.56239722		226	228	171	183	206
210	162	162	134	134	186	190	189	191	186	190
255	271	212	214	141	145	165	167			
PQLP40	PQLP	-75.90966389		45.56239722		226	228	183	185	210
210	160	176	132	132	188	188	191	191	186	188
247	259	214	214	143	145	167	167			
PQLP41	PQLP	-75.90966389		45.56239722		228	228	173	183	206
212	160	176	138	138	188	190	191	191	186	188
247	259	NA	NA	143	145	NA	NA			
PQLP42	PQLP	-75.90966389		45.56239722		230	240	183	187	192
204	162	162	130	138	186	188	189	193	186	188
255	259	214	214	139	145	167	167			
PQLP43	PQLP	-75.90966389		45.56239722		226	230	183	187	210
212	160	176	134	134	188	188	189	193	196	202
255	273	212	214	143	145	165	167			
PQLP44	PQLP	-75.90966389		45.56239722		228	228	183	187	192
202	160	178	128	132	188	192	191	193	196	196
249	259	212	212	141	145	165	165			
PQLP45	PQLP	-75.90966389		45.56239722		226	230	183	187	208
210	160	162	128	132	190	190	191	193	192	194
255	273	212	214	139	145	165	167			
PQLP46	PQLP	-75.90966389		45.56239722		228	228	185	189	206
210	160	160	128	134	190	190	189	193	186	188
255	275	212	214	145	145	165	167			
PQLP47	PQLP	-75.90966389		45.56239722		216	228	185	187	206
206	162	162	130	130	190	192	191	191	204	204
255	255	214	214	139	145	167	167			
PQLP48	PQLP	-75.90966389		45.56239722		228	230	185	187	210
210	164	170	130	130	190	190	191	191	196	196
255	255	214	214	145	145	167	167			
PQLP49	PQLP	-75.90966389		45.56239722		228	230	179	185	208
208	164	170	128	128	188	188	191	191	196	196
255	255	214	214	139	145	167	167			
PQLP50	PQLP	-75.90966389		45.56239722		228	228	183	183	208
208	160	174	130	130	188	188	189	191	186	196
249	259	214	214	143	147	167	167			
ONML01	ONML	-79.66028056		45.02016667		228	228	183	189	196
200	166	172	130	130	166	168	195	197	188	190
253	261	214	214	139	145	167	167			
ONML02	ONML	-79.66028056		45.02016667		220	228	175	185	186
188	170	172	130	132	164	168	193	193	190	200
253	261	214	216	141	141	167	169			
ONML03	ONML	-79.66028056		45.02016667		228	228	183	185	210
212	170	172	132	142	166	172	195	197	190	198
251	261	214	214	143	143	167	167			
ONML04	ONML	-79.66028056		45.02016667		228	228	183	183	196
198	182	190	134	134	168	172	201	203	190	198
255	261	214	216	143	147	167	169			
ONML05	ONML	-79.66028056		45.02016667		228	228	181	183	180
206	172	188	130	132	168	170	189	191	192	198
255	261	214	214	141	145	167	167			
ONML06	ONML	-79.66028056		45.02016667		192	228	185	187	202

210	166	188	130	134	166	170	193	195	188	194
261	261	216	216	141	145	169	169			
ONML07	ONML	-79.66028056		45.02016667		228	228	173	187	202
206	170	184	134	134	166	170	191	195	190	202
253	261	214	214	143	143	167	167			
ONML08	ONML	-79.66028056		45.02016667		228	228	173	185	200
206	170	196	132	132	168	170	191	193	190	192
253	263	214	214	143	147	167	167			
ONML09	ONML	-79.66028056		45.02016667		194	228	185	187	190
212	166	186	128	132	168	186	189	191	192	198
253	261	214	216	139	143	167	169			
ONML10	ONML	-79.66028056		45.02016667		194	228	183	185	208
210	166	184	132	132	172	186	189	191	160	198
253	261	214	214	141	143	167	167			
ONML11	ONML	-79.66028056		45.02016667		232	232	183	185	190
214	166	184	132	132	168	172	193	195	160	190
253	263	214	214	141	143	167	167			
ONML12	ONML	-79.66028056		45.02016667		228	228	183	185	172
182	166	188	134	134	172	190	193	195	188	198
253	261	214	214	143	143	167	167			
ONML13	ONML	-79.66028056		45.02016667		228	228	173	185	190
192	166	184	134	134	170	174	189	193	190	190
253	261	214	216	143	145	167	169			
ONML14	ONML	-79.66028056		45.02016667		228	228	181	185	192
200	166	196	130	130	172	190	189	195	188	208
253	261	214	216	141	145	167	169			
ONML15	ONML	-79.66028056		45.02016667		234	234	185	191	190
220	166	184	132	132	172	178	193	193	192	194
251	263	214	214	143	145	167	167			
ONML16	ONML	-79.66028056		45.02016667		228	234	183	185	180
208	168	180	134	134	168	174	191	195	190	206
253	273	214	216	143	147	167	169			
ONML17	ONML	-79.66028056		45.02016667		228	228	185	187	208
210	166	188	130	134	170	180	191	195	186	190
253	261	214	214	143	147	167	167			
ONML18	ONML	-79.66028056		45.02016667		226	228	185	187	208
210	174	174	134	138	168	172	193	195	190	194
255	257	214	216	141	147	167	169			
ONML19	ONML	-79.66028056		45.02016667		226	226	183	187	200
204	168	178	132	132	166	172	193	195	190	198
253	261	214	214	143	147	167	167			
ONML20	ONML	-79.66028056		45.02016667		228	228	173	185	194
204	166	172	136	136	170	174	189	189	188	190
251	261	214	214	141	143	167	167			
ONML21	ONML	-79.66028056		45.02016667		228	228	183	185	210
212	170	178	136	140	168	172	195	195	188	192
261	261	214	216	141	143	167	169			
ONML22	ONML	-79.66028056		45.02016667		228	228	183	185	186
224	168	174	136	136	168	170	191	193	190	194
247	263	214	216	143	143	167	169			
ONML23	ONML	-79.66028056		45.02016667		228	228	173	173	190
210	170	172	130	130	170	174	191	193	190	194
259	259	214	214	143	145	167	167			
ONML24	ONML	-79.66028056		45.02016667		226	228	183	185	216
216	168	196	130	140	166	184	193	199	190	198
259	259	214	214	141	147	167	167			
ONML25	ONML	-79.66028056		45.02016667		228	228	183	185	204
206	170	184	130	130	168	170	201	203	192	194
259	271	212	216	143	145	165	169			
ONML26	ONML	-79.66028056		45.02016667		228	228	181	181	190
202	166	166	130	132	168	170	201	203	188	192
259	259	216	216	143	145	169	169			
ONML27	ONML	-79.66028056		45.02016667		228	228	187	187	192

208	166	166	130	132	166	170	201	203	192	198
257	271	214	214	143	147	167	167			
ONML28	ONML	-79.66028056		45.02016667		228	228	185	189	208
210	174	182	130	130	164	170	201	203	190	190
255	257	214	214	141	147	167	167			
ONML29	ONML	-79.66028056		45.02016667		234	234	183	187	202
210	166	200	130	132	168	172	191	193	190	190
255	257	214	214	143	145	167	167			
ONML30	ONML	-79.66028056		45.02016667		228	228	181	185	206
210	166	170	130	130	166	172	189	193	194	202
257	273	214	216	139	145	167	169			
ONML31	ONML	-79.66028056		45.02016667		228	228	185	187	206
210	166	170	132	136	166	170	191	193	188	198
259	271	214	216	141	143	167	169			
ONML32	ONML	-79.66028056		45.02016667		228	228	185	187	202
206	168	172	128	132	168	170	193	193	188	192
259	271	214	214	141	145	167	167			
ONML33	ONML	-79.66028056		45.02016667		228	228	171	185	188
206	164	170	132	134	168	170	189	191	188	192
255	273	214	214	143	143	167	167			
ONML34	ONML	-79.66028056		45.02016667		228	228	183	185	216
216	164	170	130	132	166	174	189	193	200	202
259	271	214	214	143	145	167	167			
ONML35	ONML	-79.66028056		45.02016667		228	228	181	187	204
218	164	200	132	134	166	172	187	191	190	194
259	271	216	216	141	145	169	169			
ONML36	ONML	-79.66028056		45.02016667		226	228	173	185	190
208	164	170	132	136	166	170	189	191	190	198
253	257	214	216	143	145	167	169			
ONML37	ONML	-79.66028056		45.02016667		228	228	181	183	186
186	164	168	130	132	168	172	187	189	188	190
257	271	216	216	143	145	169	169			
ONML38	ONML	-79.66028056		45.02016667		228	228	173	185	188
196	164	182	132	132	168	172	189	189	186	192
251	259	214	216	143	143	167	169			
ONML39	ONML	-79.66028056		45.02016667		226	228	183	185	174
190	168	174	134	134	166	172	187	189	186	190
251	259	214	214	141	143	167	167			
ONML40	ONML	-79.66028056		45.02016667		228	228	173	185	188
210	164	168	132	134	168	172	181	191	194	198
253	261	216	216	143	145	169	169			
ONML41	ONML	-79.66028056		45.02016667		228	228	183	191	206
212	164	200	132	132	166	172	181	191	194	198
253	261	216	216	143	147	169	169			
ONML42	ONML	-79.66028056		45.02016667		228	228	183	185	190
190	170	174	130	132	170	174	189	193	188	190
253	261	214	214	139	145	167	167			
ONML43	ONML	-79.66028056		45.02016667		228	228	185	189	190
208	164	198	132	132	170	192	181	191	200	202
259	259	214	214	143	147	167	167			
ONML44	ONML	-79.66028056		45.02016667		228	228	183	187	218
218	164	180	130	132	170	184	179	193	200	202
253	259	214	214	143	147	167	167			
ONML45	ONML	-79.66028056		45.02016667		228	228	173	185	190
200	166	166	132	132	168	172	189	191	190	198
253	271	214	214	143	147	167	167			
ONML46	ONML	-79.66028056		45.02016667		228	228	183	187	210
210	164	164	126	130	168	172	193	197	198	198
269	269	216	218	143	145	169	171			
ONML47	ONML	-79.66028056		45.02016667		228	228	173	185	182
206	166	184	132	132	166	172	179	195	192	192
271	271	214	214	143	145	167	167			
ONML48	ONML	-79.66028056		45.02016667		228	228	183	185	206

206	168	184	132	132	164	170	183	193	192	192
253	271	214	214	145	145	167	167			
ONML49	ONML	-79.66028056		45.02016667		228	228	175	187	206
210	166	184	132	132	166	170	191	191	190	208
253	257	214	214	141	147	167	167			
ONML50	ONML	-79.66028056		45.02016667		228	228	185	189	210
210	174	182	132	134	164	170	191	193	198	204
259	259	214	216	143	147	167	169			
ONFR01	ONFR	-80.28425833		46.05228889		228	230	183	185	188
208	174	186	134	138	168	172	195	197	188	190
243	259	214	214	141	145	167	167			
ONFR02	ONFR	-80.28425833		46.05228889		226	230	183	185	180
206	180	180	140	140	168	168	191	195	192	206
243	259	214	214	141	141	167	167			
ONFR03	ONFR	-80.28425833		46.05228889		226	228	171	177	180
208	168	168	124	136	170	172	191	191	190	196
243	259	214	214	143	143	167	167			
ONFR04	ONFR	-80.28425833		46.05228889		226	230	171	185	204
206	170	182	134	138	168	172	191	191	188	196
243	261	214	214	143	147	167	167			
ONFR05	ONFR	-80.28425833		46.05228889		228	230	179	183	170
208	174	178	136	138	166	166	191	191	188	190
245	261	214	214	141	145	167	167			
ONFR06	ONFR	-80.28425833		46.05228889		226	228	183	185	176
206	162	182	136	138	166	166	191	191	184	186
243	261	216	216	141	145	169	169			
ONFR07	ONFR	-80.28425833		46.05228889		228	228	181	185	206
206	190	192	138	140	168	168	189	193	184	186
243	259	216	216	143	143	169	169			
ONFR08	ONFR	-80.28425833		46.05228889		226	228	183	185	176
210	178	186	134	138	168	168	191	191	170	184
243	261	216	216	143	143	169	169			
ONFR09	ONFR	-80.28425833		46.05228889		226	228	185	189	188
220	190	192	138	138	168	168	191	193	186	206
243	261	214	214	139	143	167	167			
ONFR10	ONFR	-80.28425833		46.05228889		226	230	183	187	180
210	176	186	138	140	168	168	191	193	190	196
243	261	214	214	141	143	167	167			
ONFR11	ONFR	-80.28425833		46.05228889		226	228	183	187	198
200	168	180	120	138	168	168	193	193	196	202
243	261	214	214	141	143	167	167			
ONFR12	ONFR	-80.28425833		46.05228889		226	230	185	189	170
172	174	176	138	140	168	168	193	193	184	196
245	261	NA	NA	143	143	NA	NA			
ONFR13	ONFR	-80.28425833		46.05228889		226	228	171	189	188
210	174	190	138	142	168	168	193	193	196	198
245	263	212	216	143	147	165	169			
ONFR14	ONFR	-80.28425833		46.05228889		228	230	183	185	206
208	184	184	142	144	170	170	191	191	186	196
245	261	214	214	141	145	167	167			
ONFR15	ONFR	-80.28425833		46.05228889		226	230	183	187	188
210	170	184	138	144	168	168	191	191	186	196
247	261	214	216	143	145	167	169			
ONFR16	ONFR	-80.28425833		46.05228889		226	228	183	185	188
188	166	174	130	134	166	166	187	191	188	204
245	261	214	216	145	147	167	169			
ONFR17	ONFR	-80.28425833		46.05228889		222	228	181	185	186
206	176	192	136	140	168	168	189	191	186	206
247	259	214	214	143	143	167	167			
ONFR18	ONFR	-80.28425833		46.05228889		228	232	185	189	198
202	170	170	136	140	168	172	191	191	184	188
251	259	214	216	141	143	167	169			
ONFR19	ONFR	-80.28425833		46.05228889		214	228	177	181	188

188	184	190	140	140	170	170	191	195	188	192
251	261	214	216	143	145	167	169			
ONFR20	ONFR	-80.28425833		46.05228889		226	230	183	185	206
210	174	174	138	142	170	174	193	205	186	192
243	263	216	216	141	147	169	169			
ONFR21	ONFR	-80.28425833		46.05228889		224	228	171	183	188
206	174	188	136	140	168	168	195	195	170	192
249	261	214	214	141	143	167	167			
ONFR22	ONFR	-80.28425833		46.05228889		226	230	183	187	180
202	142	140	140	142	170	170	193	207	194	206
245	263	214	216	143	143	167	169			
ONFR23	ONFR	-80.28425833		46.05228889		226	228	181	185	188
204	174	188	138	140	164	164	187	191	186	190
249	259	214	214	143	145	167	167			
ONFR24	ONFR	-80.28425833		46.05228889		222	230	177	181	188
210	186	190	134	136	166	166	189	193	188	190
249	259	214	216	141	145	167	169			
ONFR25	ONFR	-80.28425833		46.05228889		226	230	185	189	202
210	174	184	140	142	168	170	195	201	188	200
251	257	214	214	143	145	167	167			
ONFR26	ONFR	-80.28425833		46.05228889		222	228	177	183	188
206	172	180	136	142	170	172	193	199	186	198
253	257	214	214	143	147	167	167			
ONFR27	ONFR	-80.28425833		46.05228889		226	230	181	183	188
226	188	188	138	142	168	172	195	197	186	186
251	259	214	214	143	143	167	167			
ONFR28	ONFR	-80.28425833		46.05228889		228	230	183	185	208
216	174	192	136	138	168	170	193	197	186	204
249	259	214	214	141	143	167	167			
ONFR29	ONFR	-80.28425833		46.05228889		228	228	183	185	208
216	172	176	134	134	168	172	195	197	186	186
247	263	214	216	143	147	167	169			
ONFR30	ONFR	-80.28425833		46.05228889		228	234	183	185	208
216	166	166	136	138	170	172	195	197	190	196
245	245	214	216	139	145	167	169			
ONFR31	ONFR	-80.28425833		46.05228889		228	232	185	187	188
208	170	180	120	142	170	174	195	195	182	188
243	243	214	216	141	143	167	169			
ONFR32	ONFR	-80.28425833		46.05228889		226	230	183	185	206
226	176	184	140	142	168	170	197	203	184	192
243	263	214	214	141	147	167	167			
ONFR33	ONFR	-80.28425833		46.05228889		226	230	185	189	194
208	186	190	142	142	168	168	191	193	186	198
243	269	214	214	141	143	167	167			
ONFR34	ONFR	-80.28425833		46.05228889		226	230	183	185	202
204	170	176	138	140	168	168	195	203	186	194
247	261	214	214	141	143	167	167			
ONFR35	ONFR	-80.28425833		46.05228889		226	230	183	185	188
210	180	180	136	140	168	172	191	195	186	196
247	261	214	216	143	145	167	169			
ONFR36	ONFR	-80.28425833		46.05228889		222	228	183	185	184
186	172	190	142	142	170	170	191	195	184	188
245	263	214	216	143	147	167	169			
ONFR37	ONFR	-80.28425833		46.05228889		226	228	183	185	180
210	168	178	136	140	168	174	191	193	188	188
245	245	214	214	139	145	167	167			
ONFR38	ONFR	-80.28425833		46.05228889		228	232	183	185	204
204	172	190	136	136	168	170	193	193	184	190
249	261	214	214	143	145	167	167			
ONFR39	ONFR	-80.28425833		46.05228889		228	234	183	185	204
206	168	168	136	138	166	170	191	191	186	196
249	261	214	214	141	145	167	167			
ONFR40	ONFR	-80.28425833		46.05228889		226	228	185	187	204

218	166	168	138	142	168	170	189	189	170	186
255	259	214	214	143	147	167	167			
ONFR41	ONFR	-80.28425833		46.05228889		226	228	171	187	180
180	168	176	110	138	168	168	189	191	184	188
253	257	214	216	143	147	167	169			
ONFR42	ONFR	-80.28425833		46.05228889		228	232	183	187	188
188	168	174	134	136	166	168	189	193	196	206
253	257	214	214	139	145	167	167			
ONFR43	ONFR	-80.28425833		46.05228889		226	230	183	187	208
210	168	174	138	140	166	168	191	193	190	190
251	255	214	214	143	147	167	167			
ONFR44	ONFR	-80.28425833		46.05228889		228	230	183	185	188
194	170	174	138	138	164	170	193	205	186	188
245	245	214	216	143	147	167	169			
ONFR45	ONFR	-80.28425833		46.05228889		228	230	183	185	208
208	192	198	136	140	170	170	193	195	172	186
245	263	214	214	143	147	167	167			
ONFR46	ONFR	-80.28425833		46.05228889		228	230	171	185	204
206	190	190	144	146	168	168	191	195	184	186
245	263	214	214	143	145	167	167			
ONFR47	ONFR	-80.28425833		46.05228889		226	228	187	191	188
188	174	190	140	144	170	170	191	195	172	188
245	261	214	214	141	145	167	167			
ONFR48	ONFR	-80.28425833		46.05228889		228	230	183	185	214
216	142	180	138	142	168	168	191	193	186	196
247	261	214	216	145	145	167	169			
ONFR49	ONFR	-80.28425833		46.05228889		226	230	183	187	188
206	174	192	140	142	166	166	189	193	186	196
247	261	214	214	141	147	167	167			
ONFR50	ONFR	-80.28425833		46.05228889		222	228	183	185	204
206	180	194	142	146	166	170	187	191	188	196
247	261	214	214	143	147	167	167			
ONHF01	ONHF	-78.07988333		44.59736667		228	228	185	187	198
208	162	176	134	136	180	180	195	195	188	192
255	269	214	214	139	145	167	167			
ONHF02	ONHF	-78.07988333		44.59736667		228	236	183	183	208
212	148	160	134	136	176	176	191	195	188	190
255	255	214	216	141	143	167	169			
ONHF03	ONHF	-78.07988333		44.59736667		226	230	171	171	208
212	148	160	132	134	176	176	193	201	192	192
255	255	214	216	143	143	167	169			
ONHF04	ONHF	-78.07988333		44.59736667		230	236	189	207	192
210	148	160	134	134	170	178	181	191	188	188
249	255	214	214	143	147	167	167			
ONHF05	ONHF	-78.07988333		44.59736667		228	228	171	185	210
212	148	158	132	134	142	166	177	191	190	200
255	269	212	212	143	145	165	165			
ONHF06	ONHF	-78.07988333		44.59736667		230	230	189	189	208
212	148	158	130	134	166	166	179	191	196	196
253	253	214	214	141	143	167	167			
ONHF07	ONHF	-78.07988333		44.59736667		230	230	185	209	208
208	150	178	130	132	154	166	185	185	192	200
255	271	214	214	143	145	167	167			
ONHF08	ONHF	-78.07988333		44.59736667		230	230	185	187	184
190	158	160	130	132	164	168	179	189	192	206
249	257	214	216	143	145	167	169			
ONHF09	ONHF	-78.07988333		44.59736667		228	230	185	185	206
210	158	172	128	130	164	164	189	191	190	190
253	253	NA	NA	143	143	NA	NA			
ONHF10	ONHF	-78.07988333		44.59736667		230	230	185	207	206
206	146	160	126	128	164	166	181	191	190	190
255	255	214	214	141	145	167	167			
ONHF11	ONHF	-78.07988333		44.59736667		230	230	185	205	206

210	160	174	132	132	164	166	187	187	194	200
255	255	214	216	143	147	167	169			
ONHF12	ONHF	-78.07988333		44.59736667		230	230	185	187	200
210	148	160	132	134	166	166	189	191	190	190
253	253	214	214	143	145	167	167			
ONHF13	ONHF	-78.07988333		44.59736667		230	230	185	185	190
208	148	158	128	128	166	166	191	191	158	190
253	253	214	214	143	143	167	167			
ONHF14	ONHF	-78.07988333		44.59736667		230	230	185	205	200
210	148	160	130	134	164	166	187	191	190	190
255	255	214	214	141	145	167	167			
ONHF15	ONHF	-78.07988333		44.59736667		228	230	187	205	208
210	148	160	128	130	160	170	181	191	194	204
259	271	214	214	143	143	167	167			
ONHF16	ONHF	-78.07988333		44.59736667		222	228	169	183	188
208	150	158	128	128	166	166	187	191	190	198
255	255	214	214	141	143	167	167			
ONHF17	ONHF	-78.07988333		44.59736667		222	228	169	183	206
206	150	160	134	136	164	166	181	189	192	194
253	253	214	214	143	143	167	167			
ONHF18	ONHF	-78.07988333		44.59736667		226	228	169	183	180
208	152	160	130	134	166	168	201	201	192	194
249	257	216	216	143	143	169	169			
ONHF19	ONHF	-78.07988333		44.59736667		228	228	183	191	174
208	158	160	130	132	164	168	181	189	192	196
255	255	214	214	141	143	167	167			
ONHF20	ONHF	-78.07988333		44.59736667		228	228	183	203	170
186	148	158	130	132	166	166	189	189	190	190
253	269	214	216	141	143	167	169			
ONHF21	ONHF	-78.07988333		44.59736667		228	228	185	203	206
206	160	172	132	136	156	166	189	189	192	206
253	271	214	214	141	143	167	167			
ONHF22	ONHF	-78.07988333		44.59736667		228	228	201	203	206
206	160	178	130	132	156	166	189	189	184	190
249	257	216	216	143	143	169	169			
ONHF23	ONHF	-78.07988333		44.59736667		222	228	181	181	186
196	160	170	130	132	154	166	183	191	192	198
253	269	216	216	143	145	169	169			
ONHF24	ONHF	-78.07988333		44.59736667		228	228	185	201	206
206	158	172	130	132	166	166	179	189	194	196
253	269	214	216	141	145	167	169			
ONHF25	ONHF	-78.07988333		44.59736667		226	228	181	199	196
206	146	162	130	132	166	170	191	191	198	202
249	255	214	216	143	145	167	169			
ONHF26	ONHF	-78.07988333		44.59736667		228	230	181	199	188
188	156	166	130	136	166	172	191	191	210	210
253	253	214	214	143	145	167	167			
ONHF27	ONHF	-78.07988333		44.59736667		228	228	181	181	200
206	150	164	134	134	166	166	191	191	194	208
253	273	214	214	143	143	167	167			
ONHF28	ONHF	-78.07988333		44.59736667		228	228	171	183	176
208	150	158	134	136	166	166	191	191	192	196
253	255	214	214	143	143	167	167			
ONHF29	ONHF	-78.07988333		44.59736667		228	228	181	183	202
204	144	170	132	132	166	166	191	193	190	192
253	255	214	214	143	145	167	167			
ONHF30	ONHF	-78.07988333		44.59736667		226	228	183	199	202
204	148	160	130	132	166	166	193	193	192	192
249	257	214	214	139	145	167	167			
ONHF31	ONHF	-78.07988333		44.59736667		226	228	185	199	190
208	146	158	130	130	164	166	191	191	190	206
251	257	214	216	141	143	167	169			
ONHF32	ONHF	-78.07988333		44.59736667		228	228	185	199	206

206	154	182	130	130	164	166	181	193	198	200
255	255	214	216	141	145	167	169			
ONHF33	ONHF	-78.07988333	44.59736667	228	228	183	201	190		
210	158	174	128	130	166	166	189	199	190	194
255	255	214	216	143	147	167	169			
ONHF34	ONHF	-78.07988333	44.59736667	226	228	183	185	184		
210	160	174	128	130	142	166	179	189	192	198
251	257	214	216	141	143	167	169			
ONHF35	ONHF	-78.07988333	44.59736667	228	228	183	183	210		
210	166	166	134	140	142	166	187	191	192	198
253	253	214	214	139	145	167	167			
ONHF36	ONHF	-78.07988333	44.59736667	228	228	183	199	202		
206	158	166	128	130	142	166	191	191	192	194
255	255	214	216	143	147	167	169			
ONHF37	ONHF	-78.07988333	44.59736667	226	228	183	201	202		
206	146	158	126	128	166	166	189	191	196	200
255	255	214	214	139	145	167	167			
ONHF38	ONHF	-78.07988333	44.59736667	228	228	183	201	190		
208	158	172	128	128	166	166	189	189	194	202
255	255	214	214	143	145	167	167			
ONHF39	ONHF	-78.07988333	44.59736667	226	228	185	201	178		
208	158	178	126	128	168	184	189	189	194	198
257	257	214	214	141	145	167	167			
ONHF40	ONHF	-78.07988333	44.59736667	226	230	185	201	206		
206	146	156	132	134	142	164	187	189	190	194
255	255	214	214	143	147	167	167			
ONHF41	ONHF	-78.07988333	44.59736667	228	230	183	183	190		
210	148	158	132	134	166	166	179	189	190	210
253	257	214	216	143	147	167	169			
ONHF42	ONHF	-78.07988333	44.59736667	228	228	185	185	188		
216	146	158	132	134	166	166	189	193	194	198
255	271	214	214	139	145	167	167			
ONHF43	ONHF	-78.07988333	44.59736667	224	230	183	185	218		
218	146	158	132	132	166	166	189	189	202	206
255	271	214	214	143	147	167	167			
ONHF44	ONHF	-78.07988333	44.59736667	230	230	183	201	190		
210	158	172	130	132	166	166	191	191	190	202
251	257	216	216	143	147	169	169			
ONHF45	ONHF	-78.07988333	44.59736667	190	230	201	203	210		
210	146	158	130	132	166	166	187	191	190	202
253	257	216	216	143	147	169	169			
ONHF46	ONHF	-78.07988333	44.59736667	230	230	183	203	210		
210	156	174	128	128	166	166	179	191	194	200
251	255	214	214	143	145	167	167			
ONHF47	ONHF	-78.07988333	44.59736667	230	230	185	203	178		
206	156	170	128	130	164	168	187	191	190	202
255	271	214	214	141	147	167	167			
ONHF48	ONHF	-78.07988333	44.59736667	230	236	183	185	178		
206	162	172	128	130	166	166	187	191	202	202
255	255	216	216	145	145	169	169			
ONHF49	ONHF	-78.07988333	44.59736667	230	232	183	185	190		
210	164	176	132	136	144	168	187	191	194	206
255	255	214	214	141	147	167	167			
ONHF50	ONHF	-78.07988333	44.59736667	228	228	183	185	180		
206	160	174	130	130	168	168	189	189	190	190
255	255	214	214	143	147	167	167			
ONGR01	ONGR	-84.22216111	46.749225	230	232	185	185	206		
206	160	166	130	134	164	166	191	191	190	192
255	259	214	214	143	147	167	167			
ONGR02	ONGR	-84.22216111	46.749225	230	230	181	185	206		
208	160	174	130	132	164	166	195	201	190	192
255	261	214	214	143	143	167	167			
ONGR03	ONGR	-84.22216111	46.749225	230	232	179	183	216		

216	164	172	132	134	166	166	193	193	188	190
257	259	214	214	145	145	167	167			
ONGR04	ONGR	-84.22216111		46.749225		230	234	179	185	206
206	156	166	130	132	162	164	191	191	190	190
257	259	214	214	145	149	167	167			
ONGR05	ONGR	-84.22216111		46.749225		232	234	183	187	204
204	156	156	130	134	164	166	193	193	192	196
259	261	214	214	143	147	167	167			
ONGR06	ONGR	-84.22216111		46.749225		230	232	181	187	206
210	148	148	132	136	130	168	193	193	190	198
259	261	214	214	143	147	167	167			
ONGR07	ONGR	-84.22216111		46.749225		232	246	185	189	206
206	158	172	128	132	164	166	191	191	188	192
257	275	214	214	143	145	167	167			
ONGR08	ONGR	-84.22216111		46.749225		204	232	183	187	206
210	158	158	132	134	164	166	193	209	160	186
263	265	214	216	141	145	167	169			
ONGR09	ONGR	-84.22216111		46.749225		232	232	183	187	204
208	158	174	128	146	166	168	193	193	188	188
259	271	212	216	141	145	165	169			
ONGR10	ONGR	-84.22216111		46.749225		232	232	185	187	204
208	156	158	132	134	166	166	191	191	188	198
259	279	214	216	143	145	167	169			
ONGR11	ONGR	-84.22216111		46.749225		232	234	185	187	206
210	174	178	134	136	166	166	191	201	190	192
259	263	214	216	143	145	167	169			
ONGR12	ONGR	-84.22216111		46.749225		232	232	183	185	208
212	160	186	132	134	162	164	191	193	188	194
263	263	214	216	145	145	167	169			
ONGR13	ONGR	-84.22216111		46.749225		232	232	175	187	206
212	156	160	126	128	166	192	191	191	190	192
259	263	214	216	143	149	167	169			
ONGR14	ONGR	-84.22216111		46.749225		232	232	183	187	208
212	162	164	126	128	166	192	193	201	192	194
259	279	214	216	143	147	167	169			
ONGR15	ONGR	-84.22216111		46.749225		232	232	183	187	206
208	172	172	124	128	166	168	193	193	192	194
263	265	216	218	145	147	169	171			
ONGR16	ONGR	-84.22216111		46.749225		232	238	183	185	206
210	158	174	126	146	166	168	191	191	190	196
261	267	216	218	143	149	169	171			
ONGR17	ONGR	-84.22216111		46.749225		232	232	185	185	206
208	154	158	130	132	166	166	189	189	190	194
267	269	214	218	141	145	167	171			
ONGR18	ONGR	-84.22216111		46.749225		228	232	183	185	204
208	156	156	128	132	166	168	191	193	192	196
265	269	216	220	143	145	169	173			
ONGR19	ONGR	-84.22216111		46.749225		228	232	179	187	204
206	158	158	130	132	166	168	191	191	194	196
259	259	218	222	145	147	171	175			
ONGR20	ONGR	-84.22216111		46.749225		230	232	183	183	202
210	156	174	130	140	166	166	193	193	188	192
261	277	214	218	143	149	167	171			
ONGR21	ONGR	-84.22216111		46.749225		230	230	183	187	204
208	156	174	128	128	166	166	191	195	188	192
263	265	214	218	143	145	167	171			
ONGR22	ONGR	-84.22216111		46.749225		228	230	187	187	204
208	158	174	128	132	166	166	191	193	186	188
261	267	214	220	145	145	167	173			
ONGR23	ONGR	-84.22216111		46.749225		228	230	183	183	210
212	158	158	130	132	166	166	189	189	190	192
263	267	214	218	145	147	167	171			
ONGR24	ONGR	-84.22216111		46.749225		230	230	183	187	206

210	164	176	126	130	164	166	191	191	188	192
265	269	216	218	143	147	169	171			
ONGR25	ONGR	-84.22216111		46.749225		230	230	181	187	194
210	154	158	130	132	166	166	189	193	190	194
265	267	214	216	145	147	167	169			
ONGR26	ONGR	-84.22216111		46.749225		228	230	185	187	208
214	158	162	130	132	166	166	195	195	188	190
257	263	214	216	145	149	167	169			
ONGR27	ONGR	-84.22216111		46.749225		230	230	185	193	204
210	156	158	122	130	166	168	193	201	188	196
259	265	214	216	145	145	167	169			
ONGR28	ONGR	-84.22216111		46.749225		230	230	185	191	206
206	178	180	130	130	168	168	193	193	190	192
259	263	214	218	143	145	167	171			
ONGR29	ONGR	-84.22216111		46.749225		232	236	185	187	208
208	162	168	130	134	166	168	191	195	188	190
259	263	214	216	143	149	167	169			
ONGR30	ONGR	-84.22216111		46.749225		230	230	185	187	202
204	160	168	132	132	164	166	191	191	188	192
257	273	214	216	141	147	167	169			
ONGR31	ONGR	-84.22216111		46.749225		228	230	171	185	182
192	158	172	130	132	164	166	191	193	162	188
257	267	214	218	143	145	167	171			
ONGR32	ONGR	-84.22216111		46.749225		230	230	181	185	190
206	160	170	128	132	144	166	189	191	186	190
265	269	214	216	141	149	167	169			
ONGR33	ONGR	-84.22216111		46.749225		222	230	181	183	204
204	158	176	134	134	162	166	191	193	186	188
267	271	210	214	141	145	163	167			
ONGR34	ONGR	-84.22216111		46.749225		230	230	171	185	204
208	156	176	134	134	166	166	191	193	186	188
265	269	214	216	143	145	167	169			
ONGR35	ONGR	-84.22216111		46.749225		228	230	183	187	204
204	162	168	130	132	140	166	191	191	186	190
255	257	214	216	145	147	167	169			
ONGR36	ONGR	-84.22216111		46.749225		228	230	187	187	206
208	160	174	108	128	164	168	193	201	186	190
257	261	214	218	145	149	167	171			
ONGR37	ONGR	-84.22216111		46.749225		230	230	187	187	208
212	156	172	128	128	148	166	193	193	160	188
257	263	214	218	141	147	167	171			
ONGR38	ONGR	-84.22216111		46.749225		230	230	181	183	196
210	156	172	130	146	144	166	191	195	180	186
255	259	214	216	145	147	167	169			
ONGR39	ONGR	-84.22216111		46.749225		228	230	171	183	196
210	158	174	128	132	166	166	191	193	186	190
259	265	214	216	143	147	167	169			
ONGR40	ONGR	-84.22216111		46.749225		228	230	183	185	188
210	170	170	130	130	164	166	193	195	186	192
255	265	212	214	143	149	165	167			
ONGR41	ONGR	-84.22216111		46.749225		230	232	181	185	210
210	158	172	130	130	164	166	191	193	186	192
261	265	214	216	143	149	167	169			
ONGR42	ONGR	-84.22216111		46.749225		230	230	179	187	206
210	160	164	130	134	168	168	191	199	186	190
257	263	214	216	141	147	167	169			
ONGR43	ONGR	-84.22216111		46.749225		230	232	187	187	210
210	162	164	134	134	164	166	191	199	188	188
257	265	214	218	145	149	167	171			
ONGR44	ONGR	-84.22216111		46.749225		226	232	183	185	210
214	156	156	134	134	166	168	191	191	186	196
259	265	214	214	145	149	167	167			
ONGR45	ONGR	-84.22216111		46.749225		232	232	185	187	208

214	158	174	130	134	166	166	193	193	160	188
259	265	214	216	145	149	167	169			
ONGR46	ONGR	-84.22216111		46.749225		220	232	187	189	206
210	160	174	128	130	166	166	191	193	186	192
257	267	212	214	145	147	165	167			
ONGR47	ONGR	-84.22216111		46.749225		232	232	185	187	206
210	158	158	128	130	166	166	191	201	184	192
253	253	212	214	143	147	165	167			
ONGR48	ONGR	-84.22216111		46.749225		230	230	185	185	188
210	160	172	130	136	166	168	191	191	160	188
253	253	212	214	147	147	165	167			
ONGR49	ONGR	-84.22216111		46.749225		226	230	183	185	204
206	156	176	128	130	166	166	191	191	192	196
253	253	212	214	143	149	165	167			
ONGR50	ONGR	-84.22216111		46.749225		232	232	181	183	190
208	158	172	128	128	166	166	191	191	190	192
253	253	212	218	145	149	165	171			
ONMW01	ONMWF	-81.72323056		46.08695		228	228	183	183	208
212	180	184	130	146	166	166	163	191	190	190
255	271	214	216	145	149	167	169			
ONMW02	ONMWF	-81.72323056		46.08695		222	228	181	183	208
212	150	160	136	148	164	166	191	191	188	198
255	271	214	216	143	147	167	169			
ONMW03	ONMWF	-81.72323056		46.08695		200	200	183	183	204
210	158	160	148	148	164	164	191	193	188	198
255	255	214	214	143	147	167	167			
ONMW04	ONMWF	-81.72323056		46.08695		200	228	171	183	210
212	164	174	130	150	164	164	191	191	188	192
255	271	212	216	145	145	165	169			
ONMW05	ONMWF	-81.72323056		46.08695		226	228	183	183	210
212	160	160	132	150	150	164	191	193	188	196
255	255	214	214	145	145	167	167			
ONMW06	ONMWF	-81.72323056		46.08695		204	228	183	183	208
224	148	160	128	130	144	164	191	193	192	198
255	255	214	216	139	145	167	169			
ONMW07	ONMWF	-81.72323056		46.08695		228	242	183	183	210
212	158	174	132	148	162	168	193	199	192	198
255	273	214	214	143	145	167	167			
ONMW08	ONMWF	-81.72323056		46.08695		200	228	181	183	206
208	162	174	160	168	160	166	191	191	198	200
255	255	214	214	139	145	167	167			
ONMW09	ONMWF	-81.72323056		46.08695		228	228	185	187	206
212	158	160	128	148	152	166	191	193	188	190
255	255	214	216	143	143	167	169			
ONMW10	ONMWF	-81.72323056		46.08695		228	228	183	183	210
212	158	160	130	144	162	164	193	193	190	192
255	255	212	216	139	145	165	169			
ONMW11	ONMWF	-81.72323056		46.08695		228	230	183	185	206
208	158	160	144	154	164	166	193	195	188	198
255	273	214	216	143	147	167	169			
ONMW12	ONMWF	-81.72323056		46.08695		228	228	181	183	210
210	160	160	128	130	162	166	193	193	188	190
255	255	214	214	139	145	167	167			
ONMW13	ONMWF	-81.72323056		46.08695		228	228	183	185	206
206	160	160	128	146	162	166	193	193	194	194
255	255	214	214	143	143	167	167			
ONMW14	ONMWF	-81.72323056		46.08695		228	228	183	183	210
210	160	160	130	146	162	164	193	193	192	198
255	255	214	216	141	145	167	169			
ONMW15	ONMWF	-81.72323056		46.08695		212	212	183	185	192
208	150	160	130	146	162	164	191	195	190	206
255	273	214	216	145	149	167	169			
ONMW16	ONMWF	-81.72323056		46.08695		228	234	183	185	204

208	162	168	130	146	164	166	191	191	200	204
255	255	214	216	143	147	167	169			
ONMW17	ONMWF	-81.72323056		46.08695		228	228	183	183	206
208	158	158	132	146	144	166	191	193	198	206
255	255	214	216	145	147	167	169			
ONMW18	ONMWF	-81.72323056		46.08695		224	228	183	185	172
190	158	174	146	148	144	164	193	193	202	206
255	255	214	214	143	143	167	167			
ONMW19	ONMWF	-81.72323056		46.08695		224	228	179	183	194
194	174	174	116	148	144	164	191	191	194	198
253	271	214	214	139	143	167	167			
ONMW20	ONMWF	-81.72323056		46.08695		226	228	169	179	212
216	150	158	128	132	166	166	193	193	188	188
255	271	214	216	141	143	167	169			
ONMW21	ONMWF	-81.72323056		46.08695		214	226	183	183	190
212	158	158	136	146	166	166	193	193	206	206
255	271	214	214	141	143	167	167			
ONMW22	ONMWF	-81.72323056		46.08695		204	204	183	183	194
204	148	158	136	140	144	166	191	191	194	200
255	255	214	216	143	143	167	169			
ONMW23	ONMWF	-81.72323056		46.08695		190	190	183	183	198
206	158	172	138	148	166	166	193	193	198	206
255	255	214	214	143	145	167	167			
ONMW24	ONMWF	-81.72323056		46.08695		212	226	181	183	190
206	160	174	134	144	164	168	191	193	174	188
255	255	214	214	141	145	167	167			
ONMW25	ONMWF	-81.72323056		46.08695		226	226	181	181	204
206	152	160	132	136	166	166	191	193	190	206
255	271	214	218	143	145	167	171			
ONMW26	ONMWF	-81.72323056		46.08695		212	226	181	181	182
216	148	158	134	144	168	170	191	193	190	200
255	255	214	214	143	145	167	167			
ONMW27	ONMWF	-81.72323056		46.08695		200	226	181	181	214
216	148	160	136	146	168	168	191	195	190	198
255	255	214	216	143	143	167	169			
ONMW28	ONMWF	-81.72323056		46.08695		226	226	183	183	190
208	148	160	130	132	168	168	191	191	198	198
255	255	214	216	141	143	167	169			
ONMW29	ONMWF	-81.72323056		46.08695		228	232	179	181	208
210	158	160	132	132	168	170	191	201	190	190
253	253	214	214	143	145	167	167			
ONMW30	ONMWF	-81.72323056		46.08695		226	226	169	179	188
206	150	158	128	134	168	168	191	191	190	206
253	253	214	214	139	145	167	167			
ONMW31	ONMWF	-81.72323056		46.08695		200	226	181	181	208
210	150	158	116	146	166	168	169	191	192	198
255	255	214	216	141	143	167	169			
ONMW32	ONMWF	-81.72323056		46.08695		226	226	183	189	188
206	148	158	128	148	132	140	189	191	190	200
255	255	214	214	143	145	167	167			
ONMW33	ONMWF	-81.72323056		46.08695		218	226	181	181	174
184	158	172	138	146	162	166	191	195	192	192
255	255	214	214	145	145	167	167			
ONMW34	ONMWF	-81.72323056		46.08695		226	226	181	181	208
208	146	158	130	146	152	168	191	193	190	190
255	255	216	216	145	147	169	169			
ONMW35	ONMWF	-81.72323056		46.08695		200	226	181	183	192
206	148	158	130	134	162	168	191	193	190	196
255	255	214	214	143	147	167	167			
ONMW36	ONMWF	-81.72323056		46.08695		224	226	183	185	208
210	156	158	144	146	152	168	191	193	202	202
255	255	216	216	145	147	169	169			
ONMW37	ONMWF	-81.72323056		46.08695		226	226	183	183	190

208	160	184	132	148	166	168	191	195	188	190
255	255	214	216	145	149	167	169			
ONMW38	ONMWF	-81.72323056		46.08695		226	226	183	183	208
218	148	174	128	146	162	166	189	191	194	196
255	255	214	214	145	145	167	167			
ONMW39	ONMWF	-81.72323056		46.08695		224	226	183	185	190
194	152	152	128	144	166	166	193	193	190	198
255	273	214	214	143	145	167	167			
ONMW40	ONMWF	-81.72323056		46.08695		200	226	169	191	190
204	160	178	134	134	158	166	189	191	190	198
255	271	214	214	145	149	167	167			
ONMW41	ONMWF	-81.72323056		46.08695		226	228	181	183	178
204	158	174	128	148	160	166	189	191	198	198
255	255	214	214	141	147	167	167			
ONMW42	ONMWF	-81.72323056		46.08695		226	226	183	183	206
208	160	162	130	150	156	166	191	191	190	192
255	255	214	214	143	145	167	167			
ONMW43	ONMWF	-81.72323056		46.08695		226	228	183	183	204
208	164	174	130	134	164	164	191	201	174	198
255	255	214	216	143	149	167	169			
ONMW44	ONMWF	-81.72323056		46.08695		222	228	183	183	192
218	158	174	132	132	164	166	201	201	190	208
255	255	214	214	143	147	167	167			
ONMW45	ONMWF	-81.72323056		46.08695		228	228	183	183	206
208	158	172	148	148	164	166	193	195	190	198
255	255	214	214	143	147	167	167			
ONMW46	ONMWF	-81.72323056		46.08695		216	228	183	183	190
194	148	158	132	148	152	166	193	193	192	192
255	255	214	216	143	145	167	169			
ONMW47	ONMWF	-81.72323056		46.08695		228	228	183	183	190
196	168	174	134	146	164	166	193	193	194	198
255	273	214	214	141	145	167	167			
ONMW48	ONMWF	-81.72323056		46.08695		226	226	183	185	208
208	158	172	128	138	166	166	195	195	188	188
255	255	214	216	145	145	167	169			
ONMW49	ONMWF	-81.72323056		46.08695		222	226	183	183	206
216	154	182	132	144	164	166	195	195	188	188
255	255	214	216	141	147	167	169			
ONMW50	ONMWF	-81.72323056		46.08695		228	228	181	183	200
200	150	158	130	130	162	168	193	193	190	192
255	271	214	216	143	147	167	169			
ONRC01	ONRC	-77.39611944		45.66363056		226	226	185	185	202
206	166	166	126	128	168	170	191	191	192	192
255	255	212	216	139	145	167	171			
ONRC02	ONRC	-77.39611944		45.66363056		226	226	181	185	202
206	168	172	134	134	166	168	191	191	192	192
257	259	210	214	141	141	165	169			
ONRC03	ONRC	-77.39611944		45.66363056		224	228	187	191	192
206	164	170	134	134	168	168	191	191	192	192
255	259	212	214	143	143	167	169			
ONRC04	ONRC	-77.39611944		45.66363056		226	228	183	185	192
208	166	168	132	134	166	168	191	191	192	204
255	259	210	214	143	147	165	169			
ONRC05	ONRC	-77.39611944		45.66363056		226	228	187	199	204
204	166	170	130	130	166	166	191	191	194	194
257	257	212	214	143	145	167	169			
ONRC06	ONRC	-77.39611944		45.66363056		226	226	183	185	188
204	166	168	128	128	164	164	191	191	194	194
255	255	210	214	139	145	165	169			
ONRC07	ONRC	-77.39611944		45.66363056		226	226	183	201	208
208	172	172	130	132	164	166	191	191	192	192
255	261	212	214	143	145	167	169			
ONRC08	ONRC	-77.39611944		45.66363056		224	226	187	191	204

208	168	170	132	136	166	166	191	193	192	194
257	257	214	214	139	145	169	169			
ONRC09	ONRC	-77.39611944		45.66363056		226	228	181	185	206
212	166	172	130	136	166	166	191	191	192	192
257	257	214	218	143	143	169	169			
ONRC10	ONRC	-77.39611944		45.66363056		226	226	181	187	204
208	164	172	130	132	166	166	189	189	192	192
257	261	214	218	139	145	169	173			
ONRC11	ONRC	-77.39611944		45.66363056		226	226	181	183	204
206	168	168	132	132	166	184	191	191	190	194
257	263	212	218	143	147	167	169			
ONRC12	ONRC	-77.39611944		45.66363056		212	226	181	183	202
206	164	172	122	122	164	168	191	191	194	194
257	263	212	216	139	145	167	171			
ONRC13	ONRC	-77.39611944		45.66363056		226	226	183	187	200
206	168	170	130	130	168	168	193	193	192	194
257	261	214	216	143	143	169	169			
ONRC14	ONRC	-77.39611944		45.66363056		226	226	185	191	202
202	168	172	130	130	168	168	189	189	192	192
259	261	214	218	141	145	169	173			
ONRC15	ONRC	-77.39611944		45.66363056		220	226	181	181	190
214	170	170	130	134	168	168	189	189	190	194
257	265	214	216	141	145	169	171			
ONRC16	ONRC	-77.39611944		45.66363056		228	230	183	183	172
182	168	172	130	134	168	170	189	189	192	192
257	263	214	218	141	145	169	173			
ONRC17	ONRC	-77.39611944		45.66363056		226	230	183	189	204
208	168	170	132	134	168	168	189	189	192	192
259	259	212	216	143	143	167	167			
ONRC18	ONRC	-77.39611944		45.66363056		220	228	183	185	208
208	168	172	128	132	166	166	191	191	192	192
259	263	208	214	143	143	163	169			
ONRC19	ONRC	-77.39611944		45.66363056		220	220	183	185	204
212	168	172	128	132	168	170	189	189	192	192
259	259	212	216	139	143	167	167			
ONRC20	ONRC	-77.39611944		45.66363056		228	228	181	181	208
210	168	172	130	134	168	168	191	191	168	194
259	263	210	216	141	143	165	171			
ONRC21	ONRC	-77.39611944		45.66363056		228	228	183	189	200
204	166	170	130	132	168	168	189	189	192	192
259	263	212	214	141	143	167	169			
ONRC22	ONRC	-77.39611944		45.66363056		228	228	185	187	208
208	168	170	128	132	158	168	189	189	192	192
259	259	212	216	143	143	167	171			
ONRC23	ONRC	-77.39611944		45.66363056		222	228	183	183	204
208	168	172	126	128	168	186	177	177	180	192
261	263	210	216	143	145	165	171			
ONRC24	ONRC	-77.39611944		45.66363056		228	232	183	189	200
200	170	170	128	128	166	168	191	191	194	194
259	261	214	216	141	145	169	171			
ONRC25	ONRC	-77.39611944		45.66363056		226	228	183	183	210
210	170	172	128	128	166	170	191	191	192	192
259	261	210	216	143	145	165	171			
ONRC26	ONRC	-77.39611944		45.66363056		228	228	183	183	208
208	168	168	128	128	166	168	191	191	164	164
259	265	212	212	143	145	167	167			
ONRC27	ONRC	-77.39611944		45.66363056		230	230	181	181	186
208	166	172	130	130	166	170	183	183	184	184
259	261	212	216	143	143	167	167			
ONRC28	ONRC	-77.39611944		45.66363056		230	230	185	187	204
210	166	172	130	132	168	168	191	191	194	194
259	261	214	218	141	143	169	169			
ONRC29	ONRC	-77.39611944		45.66363056		230	230	195	197	210

210	168	172	132	142	168	168	191	191	194	194
257	259	210	216	143	145	165	171			
ONRC30	ONRC	-77.39611944		45.66363056		228	230	185	197	194
206	168	172	136	136	168	168	191	191	192	192
259	259	212	216	139	145	167	171			
ONRC31	ONRC	-77.39611944		45.66363056		230	230	185	189	190
190	168	172	132	134	166	170	189	189	192	192
257	259	212	216	141	143	167	171			
ONRC32	ONRC	-77.39611944		45.66363056		228	228	185	189	190
206	166	170	132	134	168	194	189	189	192	194
257	261	212	216	141	145	167	169			
ONRC33	ONRC	-77.39611944		45.66363056		226	230	185	191	206
210	168	172	130	142	168	168	189	189	192	194
257	261	212	216	141	143	167	169			
ONRC34	ONRC	-77.39611944		45.66363056		230	230	183	183	206
206	168	168	134	134	166	170	191	191	188	194
257	261	214	218	141	143	169	173			
ONRC35	ONRC	-77.39611944		45.66363056		230	230	183	183	172
190	170	172	128	134	168	168	189	199	190	202
259	261	212	216	137	145	167	171			
ONRC36	ONRC	-77.39611944		45.66363056		230	232	183	183	206
210	170	170	130	130	166	168	169	169	188	192
257	261	210	216	143	143	165	169			
ONRC37	ONRC	-77.39611944		45.66363056		226	230	183	187	184
200	172	172	138	138	166	170	165	165	190	192
259	259	212	214	139	145	167	169			
ONRC38	ONRC	-77.39611944		45.66363056		226	230	185	187	190
208	170	170	130	130	168	168	189	189	190	192
259	259	214	214	143	145	169	169			
ONRC39	ONRC	-77.39611944		45.66363056		228	228	185	185	210
210	170	170	122	126	168	168	167	191	190	194
259	259	216	216	141	145	171	171			
ONRC40	ONRC	-77.39611944		45.66363056		230	230	183	183	202
202	168	172	116	116	166	170	187	187	190	194
259	261	212	216	143	143	167	171			
ONRC41	ONRC	-77.39611944		45.66363056		230	230	183	187	184
202	168	172	128	128	166	170	187	187	190	194
259	261	212	216	143	147	167	171			
ONRC42	ONRC	-77.39611944		45.66363056		228	238	183	185	184
184	168	172	130	130	168	168	187	189	190	194
257	261	214	216	139	145	169	171			
ONRC43	ONRC	-77.39611944		45.66363056		228	228	181	181	190
190	168	172	134	134	166	170	173	173	192	194
259	261	214	214	143	147	169	169			
ONRC44	ONRC	-77.39611944		45.66363056		228	228	181	181	208
208	168	174	134	134	190	190	175	175	190	194
259	265	212	216	143	147	167	171			
ONRC45	ONRC	-77.39611944		45.66363056		230	230	181	183	208
208	168	172	130	130	168	170	189	189	192	192
259	265	218	218	143	147	161	163			
ONRC46	ONRC	-77.39611944		45.66363056		230	230	181	183	204
208	170	172	130	130	168	168	191	191	192	194
259	261	214	216	143	145	169	171			
ONRC47	ONRC	-77.39611944		45.66363056		206	230	183	183	204
204	174	174	130	130	168	168	183	193	196	196
259	259	212	214	141	145	167	169			
ONRC48	ONRC	-77.39611944		45.66363056		228	228	183	187	192
196	170	172	132	132	168	168	191	191	192	194
259	259	210	216	145	145	165	171			
ONRC49	ONRC	-77.39611944		45.66363056		228	228	183	187	190
190	170	172	132	132	168	168	191	191	192	196
261	263	212	216	141	147	165	167			
ONRC50	ONRC	-77.39611944		45.66363056		228	228	183	183	206

206	170	170	128	132	168	194	191	191	192	194
259	261	212	214	143	147	167	169			
ONWL01	ONWL	-80.65286389		46.84320833		230	230	185	185	202
206	156	172	136	136	168	168	191	193	194	196
255	271	214	216	143	147	165	167			
ONWL02	ONWL	-80.65286389		46.84320833		230	230	183	185	192
210	156	176	124	136	164	164	191	195	192	202
255	271	214	216	145	147	167	169			
ONWL03	ONWL	-80.65286389		46.84320833		228	232	185	189	190
202	156	156	124	136	164	164	191	193	192	202
255	255	214	214	147	149	167	167			
ONWL04	ONWL	-80.65286389		46.84320833		230	232	171	183	210
212	156	156	136	136	150	164	189	191	192	198
255	271	214	214	145	145	167	167			
ONWL05	ONWL	-80.65286389		46.84320833		230	232	185	191	206
208	160	162	136	136	144	164	191	193	190	202
255	255	214	214	143	145	167	167			
ONWL06	ONWL	-80.65286389		46.84320833		230	230	183	183	208
224	148	162	128	136	162	168	191	193	196	200
255	255	214	214	145	147	167	167			
ONWL07	ONWL	-80.65286389		46.84320833		230	230	185	185	194
212	164	182	134	134	160	166	193	193	194	202
255	273	214	214	143	149	167	167			
ONWL08	ONWL	-80.65286389		46.84320833		228	230	183	185	190
208	166	166	128	136	152	166	187	191	202	204
255	255	214	214	139	145	167	167			
ONWL09	ONWL	-80.65286389		46.84320833		230	232	185	187	206
212	150	162	128	132	162	164	189	191	190	190
255	255	214	216	143	143	167	169			
ONWL10	ONWL	-80.65286389		46.84320833		230	230	183	183	210
212	162	166	136	136	164	166	187	193	190	190
255	255	214	214	139	145	167	167			
ONWL11	ONWL	-80.65286389		46.84320833		230	230	171	185	182
206	164	190	136	136	162	166	189	193	192	202
255	273	214	214	143	147	167	167			
ONWL12	ONWL	-80.65286389		46.84320833		216	230	181	183	210
210	156	156	128	136	162	166	191	191	190	190
255	255	214	216	143	145	167	169			
ONWL13	ONWL	-80.65286389		46.84320833		230	230	187	189	206
210	162	162	136	136	162	164	189	191	192	196
255	255	214	216	143	143	167	169			
ONWL14	ONWL	-80.65286389		46.84320833		230	230	185	189	190
196	160	160	136	136	162	164	189	209	194	200
255	255	214	214	141	145	167	167			
ONWL15	ONWL	-80.65286389		46.84320833		224	230	183	185	192
208	160	160	136	136	164	166	189	189	194	198
255	273	214	214	141	145	167	167			
ONWL16	ONWL	-80.65286389		46.84320833		232	234	183	185	204
208	178	178	136	136	144	166	189	191	194	196
255	255	214	214	141	145	167	167			
ONWL17	ONWL	-80.65286389		46.84320833		230	234	183	187	190
206	150	160	136	136	144	164	187	193	198	208
255	255	214	216	143	143	167	169			
ONWL18	ONWL	-80.65286389		46.84320833		224	232	165	183	172
190	166	166	136	136	144	164	187	191	192	210
255	255	212	214	143	143	165	167			
ONWL19	ONWL	-80.65286389		46.84320833		224	224	183	183	190
208	172	172	124	136	166	166	189	191	190	192
253	271	214	214	143	143	167	167			
ONWL20	ONWL	-80.65286389		46.84320833		232	232	169	179	212
216	158	160	136	136	166	166	189	189	190	190
255	271	214	214	141	143	167	167			
ONWL21	ONWL	-80.65286389		46.84320833		232	232	183	183	190

212	160	160	136	136	144	166	189	189	194	194
255	271	214	216	141	143	167	169			
ONWL22	ONWL	-80.65286389		46.84320833		232	232	183	183	194
204	160	176	136	140	166	166	191	191	198	202
255	255	214	216	143	143	167	169			
ONWL23	ONWL	-80.65286389		46.84320833		226	232	183	183	198
206	160	174	136	136	164	168	189	191	190	192
255	255	214	214	143	145	167	167			
ONWL24	ONWL	-80.65286389		46.84320833		232	236	183	185	194
212	162	178	136	136	166	166	191	195	192	192
255	255	214	214	141	145	167	167			
ONWL25	ONWL	-80.65286389		46.84320833		230	232	183	183	182
206	180	190	136	142	168	170	191	193	192	208
255	271	214	216	143	145	167	169			
ONWL26	ONWL	-80.65286389		46.84320833		232	232	181	181	182
216	154	168	136	136	168	168	191	193	194	204
255	255	214	214	143	145	167	167			
ONWL27	ONWL	-80.65286389		46.84320833		234	234	181	181	214
216	156	156	136	136	168	168	191	193	194	202
255	255	214	216	143	143	167	169			
ONWL28	ONWL	-80.65286389		46.84320833		234	234	183	183	190
208	162	164	136	136	168	170	191	193	194	202
255	255	214	214	141	143	167	167			
ONWL29	ONWL	-80.65286389		46.84320833		234	234	187	187	190
208	180	198	136	136	168	168	191	193	196	202
253	253	214	214	143	145	167	167			
ONWL30	ONWL	-80.65286389		46.84320833		232	234	169	179	188
206	158	178	136	142	166	168	191	193	194	194
253	253	214	214	143	145	167	167			
ONWL31	ONWL	-80.65286389		46.84320833		234	234	169	189	188
206	154	154	132	140	132	140	191	193	198	198
255	255	214	214	141	145	167	167			
ONWL32	ONWL	-80.65286389		46.84320833		232	232	183	189	208
210	170	170	118	136	162	166	189	189	194	200
255	255	214	216	143	147	167	169			
ONWL33	ONWL	-80.65286389		46.84320833		230	234	185	191	190
206	158	164	136	136	152	168	189	199	192	202
255	255	212	212	143	145	165	165			
ONWL34	ONWL	-80.65286389		46.84320833		234	234	181	181	208
208	162	180	136	136	162	168	187	189	194	196
255	255	214	214	143	145	167	167			
ONWL35	ONWL	-80.65286389		46.84320833		234	234	181	183	192
206	164	196	136	136	152	168	191	191	192	194
255	255	212	212	137	145	165	165			
ONWL36	ONWL	-80.65286389		46.84320833		234	236	189	189	182
206	150	162	136	136	166	168	191	191	192	198
255	255	214	214	143	143	167	167			
ONWL37	ONWL	-80.65286389		46.84320833		230	234	183	183	190
208	164	192	136	136	162	166	191	191	200	204
255	255	214	214	139	145	167	167			
ONWL38	ONWL	-80.65286389		46.84320833		230	234	183	183	208
218	164	192	136	136	166	166	189	189	190	192
255	255	214	216	143	145	167	169			
ONWL39	ONWL	-80.65286389		46.84320833		232	232	183	185	190
194	152	164	136	136	158	166	191	191	196	198
255	273	214	214	141	145	167	167			
ONWL40	ONWL	-80.65286389		46.84320833		234	234	169	191	190
204	156	192	136	136	160	166	189	189	194	200
255	271	214	214	143	143	167	167			
ONWL41	ONWL	-80.65286389		46.84320833		234	234	169	191	202
202	166	166	136	136	156	166	189	189	194	202
255	255	214	214	143	147	167	167			
ONWL42	ONWL	-80.65286389		46.84320833		232	242	183	183	206

208	150	174	136	136	164	164	191	191	202	202
255	255	214	214	139	145	167	167			
ONWL43	ONWL	-80.65286389		46.84320833		232	232	183	189	184
206	162	162	136	136	164	166	191	199	NA	NA
255	255	214	214	141	141	167	167			
ONWL44	ONWL	-80.65286389		46.84320833		232	232	183	183	192
218	170	180	136	136	164	166	191	193	178	202
255	255	214	216	143	143	167	169			
ONWL45	ONWL	-80.65286389		46.84320833		234	234	185	185	216
216	162	182	136	136	152	166	191	193	194	210
255	255	214	214	143	147	167	167			
ONWL46	ONWL	-80.65286389		46.84320833		234	234	183	183	190
194	164	180	136	136	164	166	191	195	194	202
255	255	214	214	141	145	167	167			
ONWL47	ONWL	-80.65286389		46.84320833		210	234	183	189	184
190	164	168	132	136	166	166	191	193	194	198
255	273	214	214	141	145	167	167			
ONWL48	ONWL	-80.65286389		46.84320833		232	232	179	189	200
206	170	176	132	132	164	166	191	193	192	192
255	255	214	216	145	145	167	169			
ONWL49	ONWL	-80.65286389		46.84320833		232	232	185	187	206
206	162	180	132	132	162	168	191	193	192	192
255	255	214	214	141	147	167	167			
ONWL50	ONWL	-80.65286389		46.84320833		232	232	183	183	204
216	160	166	132	134	162	168	191	193	192	192
255	271	214	216	143	147	167	169			
ONCL01	ONCL	-94.27326944		49.08432222		228	232	193	197	200
204	154	166	122	126	164	166	181	191	194	198
265	269	216	218	147	151	167	169			
ONCL02	ONCL	-94.27326944		49.08432222		226	228	195	197	202
208	148	152	126	128	166	166	195	195	194	196
257	263	214	216	143	145	165	167			
ONCL03	ONCL	-94.27326944		49.08432222		230	234	195	197	200
204	148	148	124	126	166	166	193	193	190	198
253	253	212	214	141	145	163	165			
ONCL04	ONCL	-94.27326944		49.08432222		228	232	193	197	200
204	154	166	122	126	164	166	181	191	194	198
265	269	216	218	147	151	167	169			
ONCL05	ONCL	-94.27326944		49.08432222		228	228	179	187	204
206	158	158	130	132	166	168	191	191	194	196
259	259	218	222	145	147	171	175			
ONCL06	ONCL	-94.27326944		49.08432222		228	230	195	197	196
198	150	158	128	128	164	166	191	191	194	198
257	273	214	216	145	151	165	167			
ONCL07	ONCL	-94.27326944		49.08432222		224	228	183	187	206
210	158	158	132	134	164	166	193	209	160	186
263	265	214	216	141	145	167	169			
ONCL08	ONCL	-94.27326944		49.08432222		226	230	179	187	204
206	158	158	130	132	166	168	191	191	194	196
259	259	218	222	145	147	171	175			
ONCL09	ONCL	-94.27326944		49.08432222		226	228	193	195	212
212	150	156	118	118	164	166	189	193	188	194
255	255	214	216	145	149	165	167			
ONCL10	ONCL	-94.27326944		49.08432222		228	230	191	193	184
202	148	162	126	128	166	166	191	191	196	198
253	253	212	218	149	153	163	169			
ONCL11	ONCL	-94.27326944		49.08432222		226	228	183	187	204
208	158	174	128	146	166	168	193	193	188	188
259	271	212	216	141	145	165	169			
ONCL12	ONCL	-94.27326944		49.08432222		226	232	183	195	208
212	148	152	122	126	166	168	191	193	190	194
257	259	214	220	143	145	163	167			
ONCL13	ONCL	-94.27326944		49.08432222		224	228	193	195	198

200	146	166	126	128	166	166	191	191	198	202
253	253	212	214	147	153	163	165			
ONCL14	ONCL	-94.27326944		49.08432222		226	230	183	187	204
208	158	174	128	146	166	168	193	193	188	188
259	271	212	216	141	145	165	169			
ONCL15	ONCL	-94.27326944		49.08432222		226	228	193	195	206
212	152	158	124	126	164	168	189	193	188	194
255	259	214	218	147	149	165	167			
ONCL16	ONCL	-94.27326944		49.08432222		228	230	185	187	206
210	174	178	134	136	166	166	191	201	190	192
259	263	214	216	143	145	167	169			
ONCL17	ONCL	-94.27326944		49.08432222		228	230	185	187	206
210	174	178	134	136	166	166	191	201	190	192
259	263	214	216	143	145	167	169			
ONCL18	ONCL	-94.27326944		49.08432222		230	230	195	197	202
202	152	158	126	130	166	168	191	195	194	196
259	263	214	216	147	153	165	167			
ONCL19	ONCL	-94.27326944		49.08432222		224	228	197	199	200
204	150	164	124	126	166	166	193	201	192	198
257	267	212	214	149	151	163	165			
ONCL20	ONCL	-94.27326944		49.08432222		228	230	183	183	202
210	156	174	130	140	166	166	193	193	188	192
261	277	214	218	143	149	167	171			
ONCL21	ONCL	-94.27326944		49.08432222		224	228	193	197	200
204	154	166	122	126	164	166	181	191	194	198
265	269	216	218	147	151	167	169			
ONCL22	ONCL	-94.27326944		49.08432222		224	230	183	187	208
212	162	164	126	128	166	192	193	201	192	194
259	279	214	216	143	147	167	169			
ONCL23	ONCL	-94.27326944		49.08432222		226	234	195	195	182
204	150	162	122	126	166	168	191	195	166	194
253	253	212	214	141	141	163	165			
ONCL24	ONCL	-94.27326944		49.08432222		226	232	195	197	202
208	148	152	126	128	166	166	195	195	194	196
257	263	214	216	143	145	165	167			
ONCL25	ONCL	-94.27326944		49.08432222		226	228	183	187	208
212	162	164	126	128	166	192	193	201	192	194
259	279	214	216	143	147	167	169			
ONCL26	ONCL	-94.27326944		49.08432222		228	228	195	195	188
206	148	154	122	126	164	164	187	191	160	188
255	255	214	218	147	149	165	165			
ONCL27	ONCL	-94.27326944		49.08432222		226	230	193	193	204
206	148	148	126	128	166	166	189	189	196	198
259	271	214	218	149	151	165	169			
ONCL28	ONCL	-94.27326944		49.08432222		226	228	195	201	200
200	168	170	126	126	168	168	193	193	196	198
259	263	214	218	147	149	165	169			
ONCL29	ONCL	-94.27326944		49.08432222		224	228	191	197	188
204	144	148	126	128	166	166	189	193	196	200
265	267	214	216	149	151	165	167			
ONCL30	ONCL	-94.27326944		49.08432222		226	228	185	185	206
208	154	158	130	132	166	166	189	189	190	194
267	269	214	218	141	145	167	171			
ONCL31	ONCL	-94.27326944		49.08432222		224	230	183	187	206
210	158	158	132	134	164	166	193	209	160	186
263	265	214	216	141	145	167	169			
ONCL32	ONCL	-94.27326944		49.08432222		232	232	185	187	204
208	156	158	132	134	166	166	191	191	188	198
259	279	214	216	143	145	167	169			
ONCL33	ONCL	-94.27326944		49.08432222		226	228	183	185	208
212	160	186	132	134	162	164	191	193	188	194
263	263	214	216	145	145	167	169			
ONCL34	ONCL	-94.27326944		49.08432222		226	230	183	187	206

208	172	172	124	128	166	168	193	193	192	194
263	265	216	218	145	147	169	171			
ONCL35	ONCL	-94.27326944		49.08432222		228	234	195	203	198
204	146	148	118	126	166	168	193	201	194	202
259	265	200	216	149	149	151	167			
ONCL36	ONCL	-94.27326944		49.08432222		224	228	183	187	204
208	158	174	128	146	166	168	193	193	188	188
259	271	212	216	141	145	165	169			
ONCL37	ONCL	-94.27326944		49.08432222		228	232	183	185	204
208	156	156	128	132	166	168	191	193	192	196
265	269	216	220	143	145	169	173			
ONCL38	ONCL	-94.27326944		49.08432222		226	230	197	199	200
204	150	164	124	126	166	166	193	201	192	198
257	267	212	214	149	151	163	165			
ONCL39	ONCL	-94.27326944		49.08432222		228	228	183	185	208
212	160	186	132	134	162	164	191	193	188	194
263	263	214	216	145	145	167	169			
ONCL40	ONCL	-94.27326944		49.08432222		226	230	197	197	190
206	156	162	120	130	164	166	191	195	188	204
255	257	214	218	145	149	151	165			
ONCL41	ONCL	-94.27326944		49.08432222		226	230	193	193	204
206	148	148	126	128	166	166	189	189	196	198
259	271	214	218	149	151	165	169			
ONCL42	ONCL	-94.27326944		49.08432222		226	234	191	197	188
204	144	148	126	128	166	166	189	193	196	200
265	267	214	216	149	151	165	167			
ONCL43	ONCL	-94.27326944		49.08432222		228	228	195	197	208
212	150	154	124	126	166	168	191	193	190	194
257	261	212	216	145	149	163	167			
ONCL44	ONCL	-94.27326944		49.08432222		226	230	197	197	198
202	148	164	124	128	166	166	191	191	192	194
263	265	214	220	143	149	165	171			
ONCL45	ONCL	-94.27326944		49.08432222		224	228	183	183	202
210	156	174	130	140	166	166	193	193	188	192
261	277	214	218	143	149	167	171			
ONCL46	ONCL	-94.27326944		49.08432222		228	232	175	187	206
212	156	160	126	128	166	192	191	191	190	192
259	263	214	216	143	149	167	169			
ONCL47	ONCL	-94.27326944		49.08432222		228	232	183	185	206
210	158	174	126	146	166	168	191	191	190	196
261	267	216	218	143	149	169	171			
ONCL48	ONCL	-94.27326944		49.08432222		228	228	175	187	206
212	156	160	126	128	166	192	191	191	190	192
259	263	214	216	143	149	167	169			
ONCL49	ONCL	-94.27326944		49.08432222		228	232	185	187	204
208	156	158	132	134	166	166	191	191	188	198
259	279	214	216	143	145	167	169			
ONCL50	ONCL	-94.27326944		49.08432222		226	230	185	187	204
208	156	158	132	134	166	166	191	191	188	198
259	279	214	216	143	145	167	169			
ONTO01	ONTO	-79.47838333		47.13071667		230	232	183	187	206
208	156	160	132	138	166	168	191	193	188	192
257	271	214	216	139	145	167	169			
ONTO02	ONTO	-79.47838333		47.13071667		230	230	183	183	202
204	156	160	120	136	164	168	189	193	190	192
255	271	214	214	145	147	167	167			
ONTO03	ONTO	-79.47838333		47.13071667		230	230	181	185	200
208	160	164	136	162	166	168	189	193	194	200
257	255	214	216	147	149	167	169			
ONTO04	ONTO	-79.47838333		47.13071667		216	230	179	183	176
208	154	154	120	132	164	168	191	193	192	196
255	271	212	214	145	145	165	167			
ONTO05	ONTO	-79.47838333		47.13071667		230	230	185	185	194

212	152	160	134	138	166	168	189	193	186	188
255	255	212	214	143	145	165	167			
ONTO06	ONTO	-79.47838333		47.13071667		230	230	181	185	200
222	152	160	132	134	166	168	187	189	188	190
255	255	214	214	145	147	167	167			
ONTO07	ONTO	-79.47838333		47.13071667		224	230	183	187	206
208	152	160	130	132	166	168	189	191	194	200
257	273	214	214	141	149	167	167			
ONTO08	ONTO	-79.47838333		47.13071667		228	230	183	185	204
206	152	160	130	134	166	168	191	191	192	196
255	255	212	214	139	145	165	167			
ONTO09	ONTO	-79.47838333		47.13071667		230	232	183	187	202
214	152	160	136	136	166	168	189	189	186	188
255	255	214	216	143	143	167	169			
ONTO10	ONTO	-79.47838333		47.13071667		230	230	183	187	206
206	138	138	132	136	164	166	189	191	188	190
255	255	210	214	137	145	163	167			
ONTO11	ONTO	-79.47838333		47.13071667		230	230	185	187	182
212	158	164	134	134	164	168	189	195	188	190
257	273	212	214	143	147	165	167			
ONTO12	ONTO	-79.47838333		47.13071667		216	230	187	191	194
194	156	164	132	136	166	170	187	189	176	190
255	255	212	214	143	145	165	167			
ONTO13	ONTO	-79.47838333		47.13071667		230	230	181	193	206
210	162	164	134	138	164	166	189	191	192	194
255	255	214	214	143	143	167	167			
ONTO14	ONTO	-79.47838333		47.13071667		230	230	183	187	206
210	162	166	136	140	166	168	191	193	194	200
255	255	210	214	139	145	163	167			
ONTO15	ONTO	-79.47838333		47.13071667		224	230	191	197	208
210	162	164	134	136	164	168	189	191	192	196
257	273	212	214	141	145	165	167			
ONTO16	ONTO	-79.47838333		47.13071667		232	234	191	199	202
210	162	166	140	144	166	168	187	191	186	188
255	255	214	216	141	145	167	169			
ONTO17	ONTO	-79.47838333		47.13071667		230	234	185	187	198
206	162	164	132	134	164	168	189	191	188	190
255	255	212	216	143	143	165	169			
ONTO18	ONTO	-79.47838333		47.13071667		224	232	185	189	174
204	162	162	130	160	166	178	189	191	188	190
255	255	214	214	139	143	167	167			
ONTO19	ONTO	-79.47838333		47.13071667		224	224	187	191	202
208	158	162	132	136	164	168	195	197	188	190
257	271	216	216	143	143	169	169			
ONTO20	ONTO	-79.47838333		47.13071667		232	232	185	189	202
208	152	162	134	138	164	168	189	193	188	190
255	271	214	216	139	143	167	169			
ONTO21	ONTO	-79.47838333		47.13071667		232	232	183	189	202
208	154	164	132	136	166	168	189	191	176	190
255	271	212	214	141	143	165	167			
ONTO22	ONTO	-79.47838333		47.13071667		232	232	185	193	200
210	156	158	130	132	164	168	189	191	192	194
255	255	214	214	143	143	167	167			
ONTO23	ONTO	-79.47838333		47.13071667		226	232	181	187	202
204	148	154	128	134	166	168	191	197	194	200
253	255	214	216	143	147	167	169			
ONTO24	ONTO	-79.47838333		47.13071667		232	236	183	185	208
212	152	160	130	136	164	168	193	195	192	196
255	255	214	216	141	145	167	169			
ONTO25	ONTO	-79.47838333		47.13071667		230	232	185	191	208
214	138	138	130	134	164	170	191	193	186	188
255	271	212	214	143	145	165	167			
ONTO26	ONTO	-79.47838333		47.13071667		232	232	183	187	176

200	158	164	132	136	168	186	189	191	188	190
255	255	214	218	143	145	167	171			
ONTO27	ONTO	-79.47838333		47.13071667		234	234	181	187	198
204	156	164	132	140	164	166	189	193	188	190
253	255	214	216	139	143	167	169			
ONTO28	ONTO	-79.47838333		47.13071667		234	234	183	185	198
200	144	156	134	136	166	168	193	195	176	190
255	255	214	214	141	143	167	167			
ONTO29	ONTO	-79.47838333		47.13071667		234	234	183	191	200
206	162	164	134	138	166	168	189	193	192	194
253	253	214	216	143	145	167	169			
ONTO30	ONTO	-79.47838333		47.13071667		232	234	183	187	178
204	162	166	120	134	164	168	189	193	194	200
251	253	214	214	143	145	167	167			
ONTO31	ONTO	-79.47838333		47.13071667		234	234	181	187	172
202	162	164	134	142	162	170	191	193	192	196
255	255	214	216	139	145	167	169			
ONTO32	ONTO	-79.47838333		47.13071667		232	232	181	185	188
188	162	166	138	142	166	168	191	197	186	188
255	255	214	216	143	147	167	169			
ONTO33	ONTO	-79.47838333		47.13071667		230	234	183	189	204
208	162	164	132	136	168	184	189	193	188	190
251	255	214	216	143	145	167	169			
ONTO34	ONTO	-79.47838333		47.13071667		234	234	185	189	204
204	162	162	128	132	164	168	193	193	198	200
255	255	212	216	143	145	165	169			
ONTO35	ONTO	-79.47838333		47.13071667		234	234	181	187	200
204	162	164	130	136	166	168	187	193	186	192
255	255	214	216	137	145	167	169			
ONTO36	ONTO	-79.47838333		47.13071667		234	236	183	187	202
204	164	164	134	138	166	168	191	193	188	192
251	255	214	216	143	143	167	169			
ONTO37	ONTO	-79.47838333		47.13071667		230	234	185	189	198
200	162	162	130	134	166	168	189	193	190	192
255	255	214	216	139	145	167	169			
ONTO38	ONTO	-79.47838333		47.13071667		230	234	183	189	190
194	162	164	130	132	166	170	191	195	188	190
255	255	214	214	143	145	167	167			
ONTO39	ONTO	-79.47838333		47.13071667		232	232	187	193	170
186	164	172	128	134	166	168	191	195	188	190
247	273	214	216	141	145	167	169			
ONTO40	ONTO	-79.47838333		47.13071667		234	234	187	197	186
190	162	162	130	132	166	168	191	197	176	190
255	271	214	214	139	143	167	167			
ONTO41	ONTO	-79.47838333		47.13071667		234	234	187	193	182
206	166	174	130	134	166	170	191	195	192	194
255	255	214	216	143	147	167	169			
ONTO42	ONTO	-79.47838333		47.13071667		232	242	187	193	196
206	168	168	128	132	166	168	193	197	194	200
253	255	212	214	139	145	165	167			
ONTO43	ONTO	-79.47838333		47.13071667		232	232	179	183	202
206	170	170	132	134	166	168	191	195	192	196
255	255	212	214	141	141	165	167			
ONTO44	ONTO	-79.47838333		47.13071667		232	232	181	185	172
172	158	158	128	134	166	168	191	199	186	188
255	255	212	216	143	143	165	169			
ONTO45	ONTO	-79.47838333		47.13071667		232	232	185	189	198
202	154	158	132	136	168	168	201	203	188	190
251	255	214	216	143	147	167	169			
ONTO46	ONTO	-79.47838333		47.13071667		232	232	181	193	202
204	158	158	132	136	168	170	195	197	196	198
255	255	214	214	141	145	167	167			
ONTO47	ONTO	-79.47838333		47.13071667		226	232	183	187	200

206	158	158	132	138	166	168	191	195	196	202
257	273	214	216	141	145	167	169			
ONTO48	ONTO	-79.47838333		47.13071667		232	236	181	185	202
206	158	160	130	136	168	168	193	195	186	190
255	255	214	214	145	145	167	167			
ONTO49	ONTO	-79.47838333		47.13071667		230	232	179	189	204
208	158	160	132	134	168	170	193	197	186	194
255	255	216	218	141	147	169	171			
ONTO50	ONTO	-79.47838333		47.13071667		232	232	183	187	194
200	154	158	132	136	166	170	193	195	192	200
247	271	214	216	143	147	167	169			
MEEB01	MEEB	-69.16459444		44.79020278		228	228	187	189	188
206	158	174	130	132	160	164	191	199	190	198
257	257	216	218	141	143	167	169			
MEEB02	MEEB	-69.16459444		44.79020278		214	214	185	193	208
208	160	174	128	132	160	166	187	193	184	192
259	259	212	216	137	145	163	167			
MEEB03	MEEB	-69.16459444		44.79020278		230	230	187	187	178
204	156	174	138	138	162	164	191	201	184	192
257	257	216	216	137	145	167	167			
MEEB04	MEEB	-69.16459444		44.79020278		228	230	187	187	178
204	158	176	138	140	162	166	189	191	186	192
257	261	216	218	139	145	167	169			
MEEB05	MEEB	-69.16459444		44.79020278		230	230	185	191	196
196	156	174	118	130	164	164	191	193	190	192
255	255	212	214	139	145	163	165			
MEEB06	MEEB	-69.16459444		44.79020278		236	238	185	187	180
204	160	174	134	138	164	164	187	191	188	194
255	257	200	216	139	145	151	167			
MEEB07	MEEB	-69.16459444		44.79020278		232	232	187	187	200
200	160	168	130	134	162	164	189	195	192	196
257	259	216	218	139	145	167	169			
MEEB08	MEEB	-69.16459444		44.79020278		230	230	185	187	188
214	172	172	140	142	164	164	189	197	190	198
255	255	218	220	139	145	169	171			
MEEB09	MEEB	-69.16459444		44.79020278		234	240	185	189	214
214	158	162	126	130	164	164	191	195	194	198
261	263	216	218	137	145	167	169			
MEEB10	MEEB	-69.16459444		44.79020278		232	240	189	189	214
214	158	160	136	136	164	164	191	195	190	194
261	261	214	216	139	145	165	167			
MEEB11	MEEB	-69.16459444		44.79020278		222	222	185	189	214
214	162	162	128	130	164	164	189	197	192	194
261	261	214	214	139	145	165	165			
MEEB12	MEEB	-69.16459444		44.79020278		234	238	187	189	178
204	156	158	132	138	162	164	187	191	190	196
257	257	216	216	137	145	167	167			
MEEB13	MEEB	-69.16459444		44.79020278		230	230	187	189	174
204	166	168	130	132	164	164	187	191	188	192
255	257	216	218	139	145	167	169			
MEEB14	MEEB	-69.16459444		44.79020278		232	232	173	187	214
214	158	158	136	140	164	164	189	197	188	194
261	261	214	214	139	145	165	165			
MEEB15	MEEB	-69.16459444		44.79020278		230	230	183	187	196
196	156	170	132	134	164	164	187	195	186	190
253	253	214	214	139	145	165	165			
MEEB16	MEEB	-69.16459444		44.79020278		230	236	187	187	178
204	158	176	128	136	164	164	191	193	192	192
257	257	216	218	139	145	167	169			
MEEB17	MEEB	-69.16459444		44.79020278		230	238	187	189	180
204	158	182	132	134	162	164	189	195	190	192
255	255	214	214	139	145	165	165			
MEEB18	MEEB	-69.16459444		44.79020278		224	230	185	193	186

186	158	172	126	130	162	164	189	193	192	196
259	259	214	216	137	145	165	167			
MEEB19	MEEB	-69.16459444		44.79020278		232	232	189	191	182
204	158	174	132	136	162	164	187	191	186	194
257	257	216	218	141	145	167	169			
MEEB20	MEEB	-69.16459444		44.79020278		230	232	185	187	202
226	158	158	132	134	164	166	187	195	188	192
259	259	216	216	139	145	167	167			
MEEB21	MEEB	-69.16459444		44.79020278		230	236	187	189	178
188	158	176	130	138	164	164	189	193	192	192
257	257	216	218	139	145	167	169			
MEEB22	MEEB	-69.16459444		44.79020278		232	238	187	189	188
214	158	158	138	138	164	166	191	191	188	194
261	261	214	214	139	145	165	165			
MEEB23	MEEB	-69.16459444		44.79020278		232	232	185	187	182
210	158	174	132	132	164	164	189	193	186	192
259	259	214	214	139	145	165	165			
MEEB24	MEEB	-69.16459444		44.79020278		214	214	185	193	178
208	156	160	128	130	162	162	189	197	192	196
259	259	216	218	139	145	167	169			
MEEB25	MEEB	-69.16459444		44.79020278		232	232	185	185	184
212	160	174	130	134	162	162	189	197	190	192
259	259	218	218	137	145	169	169			
MEEB26	MEEB	-69.16459444		44.79020278		246	246	185	185	182
218	174	176	130	158	164	164	189	197	188	194
259	259	216	218	139	145	167	169			
MEEB27	MEEB	-69.16459444		44.79020278		232	232	185	187	190
216	156	158	126	130	164	164	189	197	184	192
259	259	216	216	139	145	167	167			
MEEB28	MEEB	-69.16459444		44.79020278		232	238	183	185	190
216	158	160	132	134	164	164	191	193	186	192
259	259	218	218	139	145	169	169			
MEEB29	MEEB	-69.16459444		44.79020278		230	230	185	187	182
198	158	172	112	134	162	164	187	197	188	192
257	259	194	214	139	145	165	165			
MEEB30	MEEB	-69.16459444		44.79020278		232	232	185	189	188
214	158	168	126	130	164	164	189	197	188	194
259	259	202	216	139	145	153	167			
MEEB31	MEEB	-69.16459444		44.79020278		230	230	171	187	182
204	162	176	130	132	162	166	187	191	186	194
259	259	208	216	139	145	159	167			
MEEB32	MEEB	-69.16459444		44.79020278		230	236	187	189	180
204	158	174	132	136	162	164	187	195	184	190
255	257	214	214	139	147	165	165			
MEEB33	MEEB	-69.16459444		44.79020278		230	238	185	187	180
204	158	158	128	132	164	164	189	197	188	194
255	255	214	218	137	145	165	169			
MEEB34	MEEB	-69.16459444		44.79020278		224	230	185	187	180
204	158	182	130	130	164	166	189	195	184	192
255	255	216	216	139	145	167	167			
MEEB35	MEEB	-69.16459444		44.79020278		214	214	185	187	200
200	160	174	130	134	164	164	189	193	188	190
255	257	200	214	139	145	151	165			
MEEB36	MEEB	-69.16459444		44.79020278		232	232	187	189	178
188	160	174	130	132	162	162	187	189	190	194
257	257	216	216	139	145	167	167			
MEEB37	MEEB	-69.16459444		44.79020278		230	230	183	191	198
224	158	160	112	130	162	166	189	193	190	196
257	257	214	216	139	145	165	167			
MEEB38	MEEB	-69.16459444		44.79020278		228	230	185	187	196
196	158	158	128	134	162	166	187	191	184	192
255	259	214	216	141	147	165	167			
MEEB39	MEEB	-69.16459444		44.79020278		230	230	189	189	178

178	158	174	128	132	160	166	187	193	192	196
259	259	216	218	139	145	167	169			
MEEB40	MEEB	-69.16459444		44.79020278		220	232	185	187	190
218	156	160	132	134	164	164	193	201	190	192
259	259	214	214	139	145	165	165			
MEEB41	MEEB	-69.16459444		44.79020278		222	222	187	189	200
200	158	174	130	130	164	166	191	193	188	192
255	255	216	216	137	145	167	167			
MEEB42	MEEB	-69.16459444		44.79020278		230	232	185	185	188
214	160	160	142	146	NA	NA	189	197	190	192
259	259	214	214	139	145	165	165			
MEEB43	MEEB	-69.16459444		44.79020278		228	230	189	189	178
188	156	158	132	134	164	166	187	197	190	192
257	257	216	216	137	145	167	167			
MEEB44	MEEB	-69.16459444		44.79020278		228	230	187	187	204
204	166	174	128	134	162	162	187	191	186	194
259	261	214	216	139	145	165	167			
MEEB45	MEEB	-69.16459444		44.79020278		222	222	185	189	186
218	158	170	130	158	164	164	189	195	188	194
259	259	216	218	139	147	167	169			
MEEB46	MEEB	-69.16459444		44.79020278		218	230	185	187	208
208	158	172	128	130	162	166	189	193	192	198
259	259	214	218	139	145	165	169			
MEEB47	MEEB	-69.16459444		44.79020278		246	246	187	193	178
210	158	174	130	136	164	166	189	193	186	192
259	259	214	216	139	145	165	167			
MEEB48	MEEB	-69.16459444		44.79020278		230	232	189	189	182
204	158	176	132	134	160	164	189	201	194	194
257	257	216	216	139	145	167	167			
MEEB49	MEEB	-69.16459444		44.79020278		230	230	187	189	178
188	168	176	128	132	162	164	189	195	192	196
257	257	204	216	139	147	155	167			
MEEB50	MEEB	-69.16459444		44.79020278		226	232	185	187	188
214	174	174	140	144	164	164	189	197	190	194
259	261	212	214	139	145	163	165			
MEBSP01	MEBSP	-68.63551389		45.67254167		228	232	187	189	188
206	158	174	130	132	162	164	189	193	190	194
257	257	218	218	139	145	169	169			
MEBSP02	MEBSP	-68.63551389		45.67254167		220	220	185	187	182
210	158	174	132	132	164	180	189	195	194	206
259	259	214	214	137	145	165	165			
MEBSP03	MEBSP	-68.63551389		45.67254167		230	230	171	187	182
204	162	176	124	130	162	164	189	193	188	194
259	259	216	216	139	145	167	167			
MEBSP04	MEBSP	-68.63551389		45.67254167		222	222	187	189	178
208	174	176	128	130	164	164	187	193	186	190
259	259	214	214	139	145	165	165			
MEBSP05	MEBSP	-68.63551389		45.67254167		238	238	175	189	178
204	158	160	126	126	164	168	191	195	188	198
257	257	214	216	139	145	165	167			
MEBSP06	MEBSP	-68.63551389		45.67254167		232	238	183	185	190
208	158	160	120	124	162	166	197	205	188	192
253	253	214	214	141	145	165	165			
MEBSP07	MEBSP	-68.63551389		45.67254167		230	230	185	187	178
188	148	162	128	132	164	164	187	197	188	192
251	251	214	216	139	145	165	167			
MEBSP08	MEBSP	-68.63551389		45.67254167		230	236	185	187	194
208	160	174	130	132	142	166	187	195	184	190
255	257	214	216	137	145	165	167			
MEBSP09	MEBSP	-68.63551389		45.67254167		238	238	175	189	214
214	158	162	126	130	164	166	187	197	190	198
261	263	216	218	137	145	167	169			
MEBSP10	MEBSP	-68.63551389		45.67254167		230	230	183	187	196

196	156	170	132	134	164	166	193	195	190	200
253	253	214	214	139	145	165	165			
MEBSP11	MEBSP	-68.63551389		45.67254167		236	236	175	187	178
188	148	160	122	124	160	164	195	195	190	198
251	253	216	218	139	145	167	169			
MEBSP12	MEBSP	-68.63551389		45.67254167		244	244	187	193	178
210	158	166	130	136	164	166	191	193	188	196
259	259	214	214	139	145	165	165			
MEBSP13	MEBSP	-68.63551389		45.67254167		230	234	187	187	204
204	166	174	126	128	164	164	193	193	190	196
259	261	216	216	137	145	167	167			
MEBSP14	MEBSP	-68.63551389		45.67254167		232	232	175	187	200
200	160	168	130	134	164	164	187	191	188	202
253	255	212	216	141	141	163	167			
MEBSP15	MEBSP	-68.63551389		45.67254167		230	230	187	189	178
188	148	162	130	132	164	166	191	193	194	198
251	251	216	216	139	145	167	167			
MEBSP16	MEBSP	-68.63551389		45.67254167		234	238	187	189	210
212	156	158	130	132	164	164	189	193	160	188
257	257	214	218	137	137	165	169			
MEBSP17	MEBSP	-68.63551389		45.67254167		232	232	185	187	208
210	156	158	126	130	162	164	191	193	188	192
259	259	214	216	139	145	165	167			
MEBSP18	MEBSP	-68.63551389		45.67254167		230	230	185	187	174
204	150	162	130	132	164	164	191	203	188	204
251	251	214	216	137	141	165	167			
MEBSP19	MEBSP	-68.63551389		45.67254167		228	230	185	187	200
200	160	174	130	134	162	166	191	195	182	196
251	251	216	216	137	141	167	167			
MEBSP20	MEBSP	-68.63551389		45.67254167		238	238	187	189	204
208	158	182	132	134	164	164	193	195	160	188
255	255	214	214	139	145	165	165			
MEBSP21	MEBSP	-68.63551389		45.67254167		228	230	185	187	196
196	158	158	128	134	162	164	191	193	190	198
253	253	212	214	139	145	163	165			
MEBSP22	MEBSP	-68.63551389		45.67254167		232	232	187	189	208
208	146	162	128	130	164	166	199	203	192	198
259	259	214	214	139	145	165	165			
MEBSP23	MEBSP	-68.63551389		45.67254167		226	232	185	187	208
208	174	174	140	144	160	164	191	203	188	196
259	261	214	218	139	145	165	169			
MEBSP24	MEBSP	-68.63551389		45.67254167		220	232	185	187	176
176	156	160	122	124	164	164	193	195	188	192
253	255	214	214	139	145	165	165			
MEBSP25	MEBSP	-68.63551389		45.67254167		232	232	187	189	186
186	160	160	126	130	164	166	189	191	190	198
259	259	214	216	137	145	165	167			
MEBSP26	MEBSP	-68.63551389		45.67254167		230	232	189	189	182
204	158	176	132	134	162	164	191	193	188	206
251	255	216	216	139	145	167	167			
MEBSP27	MEBSP	-68.63551389		45.67254167		228	230	189	189	178
178	158	174	128	132	162	166	191	193	188	192
259	259	216	216	139	145	167	167			
MEBSP28	MEBSP	-68.63551389		45.67254167		232	232	185	189	206
210	158	168	130	132	164	164	187	195	188	200
259	259	214	214	139	145	165	165			
MEBSP29	MEBSP	-68.63551389		45.67254167		230	230	187	187	178
204	144	158	122	122	162	164	191	195	192	198
257	257	214	216	139	145	165	167			
MEBSP30	MEBSP	-68.63551389		45.67254167		230	236	187	189	206
210	158	174	132	136	164	164	193	195	188	198
255	257	214	216	139	145	165	167			
MEBSP31	MEBSP	-68.63551389		45.67254167		232	240	187	189	214

214	158	160	136	136	162	166	187	197	182	192
261	261	216	216	137	145	167	167			
MEBSP32	MEBSP	-68.63551389		45.67254167		232	238	187	189	188
214	158	158	138	138	162	164	189	191	192	196
261	261	216	216	139	145	167	167			
MEBSP33	MEBSP	-68.63551389		45.67254167		230	230	185	187	182
198	158	172	112	134	164	164	193	195	190	194
251	251	214	214	139	145	165	165			
MEBSP34	MEBSP	-68.63551389		45.67254167		238	238	185	187	206
212	158	158	128	132	162	166	189	193	194	198
255	255	214	214	139	145	165	165			
MEBSP35	MEBSP	-68.63551389		45.67254167		232	232	175	187	208
210	174	176	130	158	162	164	187	195	188	194
253	253	218	218	139	145	169	169			
MEBSP36	MEBSP	-68.63551389		45.67254167		232	232	189	189	214
214	158	158	136	140	164	164	191	199	188	200
261	261	218	218	137	137	169	169			
MEBSP37	MEBSP	-68.63551389		45.67254167		230	232	175	187	202
226	164	166	132	134	164	166	187	197	186	190
259	259	214	214	139	145	165	165			
MEBSP38	MEBSP	-68.63551389		45.67254167		238	238	185	187	178
188	158	168	126	126	164	166	191	197	188	206
257	257	214	214	139	145	165	165			
MEBSP39	MEBSP	-68.63551389		45.67254167		214	214	175	187	214
214	162	162	128	130	164	164	191	195	190	204
253	253	216	216	137	145	167	167			
MEBSP40	MEBSP	-68.63551389		45.67254167		232	232	185	189	198
208	158	170	130	132	160	164	187	195	186	192
253	253	216	216	139	145	167	167			
MEBSP41	MEBSP	-68.63551389		45.67254167		222	224	187	189	200
200	158	174	130	130	164	164	191	195	188	198
255	255	214	214	139	145	165	165			
MEBSP42	MEBSP	-68.63551389		45.67254167		230	230	185	191	196
196	156	174	118	130	162	166	187	195	188	192
251	251	212	214	139	145	163	165			
MEBSP43	MEBSP	-68.63551389		45.67254167		230	230	187	189	198
224	158	160	112	130	164	168	191	193	188	196
251	251	214	216	139	145	165	167			
MEBSP44	MEBSP	-68.63551389		45.67254167		228	230	187	187	202
208	160	174	130	132	164	164	199	209	188	196
257	261	214	216	139	145	165	167			
MEBSP45	MEBSP	-68.63551389		45.67254167		230	230	185	187	208
210	172	172	140	142	164	164	197	199	194	196
255	255	216	216	137	145	167	167			
MEBSP46	MEBSP	-68.63551389		45.67254167		230	230	189	191	182
204	144	160	132	136	164	168	187	195	186	190
251	251	216	216	139	145	167	167			
MEBSP47	MEBSP	-68.63551389		45.67254167		246	246	185	185	188
192	160	174	130	134	164	164	191	191	184	198
259	259	214	214	139	145	165	165			
MEBSP48	MEBSP	-68.63551389		45.67254167		230	232	175	187	208
208	166	166	128	132	162	168	191	195	184	196
259	259	214	216	139	145	165	167			
MEBSP49	MEBSP	-68.63551389		45.67254167		230	232	185	185	208
210	160	160	142	146	164	164	193	201	190	198
259	259	214	216	139	145	165	167			
MEBSP50	MEBSP	-68.63551389		45.67254167		222	222	185	187	192
200	158	182	130	130	164	164	191	191	196	196
255	255	214	214	139	145	165	165			
MASB01	MASB	-73.28606667		42.26363333		228	228	187	187	186
206	158	172	128	132	168	186	193	201	192	200
257	257	214	214	139	145	165	165			
MASB02	MASB	-73.28606667		42.26363333		230	230	187	187	206

208	158	158	128	132	170	188	191	193	162	192
259	259	212	214	139	145	163	165			
MASB03	MASB	-73.28606667		42.26363333		228	230	187	189	204
208	158	172	126	130	166	188	193	203	162	190
259	261	212	214	139	145	163	165			
MASB04	MASB	-73.28606667		42.26363333		230	230	187	189	174
210	158	202	134	138	170	188	193	195	160	190
259	259	212	214	137	143	163	165			
MASB05	MASB	-73.28606667		42.26363333		230	230	187	187	174
212	158	172	128	136	170	188	193	195	160	190
255	257	212	212	137	145	163	163			
MASB06	MASB	-73.28606667		42.26363333		230	230	187	189	208
208	158	174	130	132	170	188	193	193	160	188
255	271	212	212	137	145	163	163			
MASB07	MASB	-73.28606667		42.26363333		230	230	187	189	192
206	158	174	130	132	170	188	193	195	160	188
255	255	212	212	141	145	163	163			
MASB08	MASB	-73.28606667		42.26363333		230	230	187	189	202
202	160	172	118	118	170	188	193	195	160	190
255	259	212	212	141	145	163	163			
MASB09	MASB	-73.28606667		42.26363333		230	230	187	187	186
226	186	198	120	132	170	188	193	195	160	188
255	261	212	212	141	145	163	163			
MASB10	MASB	-73.28606667		42.26363333		228	230	187	189	182
186	158	198	130	134	170	188	189	191	164	190
253	257	212	212	137	141	163	163			
MASB11	MASB	-73.28606667		42.26363333		230	236	187	189	192
204	158	176	132	138	176	194	191	193	192	200
257	257	210	212	137	145	161	163			
MASB12	MASB	-73.28606667		42.26363333		230	236	187	189	202
206	158	194	130	134	170	194	191	191	164	194
257	257	210	210	141	145	161	161			
MASB13	MASB	-73.28606667		42.26363333		230	230	177	187	208
216	158	194	122	146	170	188	171	193	192	200
257	257	212	214	139	145	163	165			
MASB14	MASB	-73.28606667		42.26363333		234	238	187	189	190
208	160	186	128	130	174	194	191	193	168	190
253	253	212	212	139	145	163	163			
MASB15	MASB	-73.28606667		42.26363333		228	230	187	187	194
206	158	194	130	134	170	188	191	193	188	202
253	255	210	210	139	145	161	161			
MASB16	MASB	-73.28606667		42.26363333		230	236	187	189	202
206	158	176	132	132	170	194	193	195	188	202
253	253	212	214	141	145	163	165			
MASB17	MASB	-73.28606667		42.26363333		230	236	187	189	194
204	158	184	130	134	170	194	193	193	160	188
253	253	214	214	137	139	165	165			
MASB18	MASB	-73.28606667		42.26363333		230	230	185	187	200
204	158	158	146	146	170	188	193	193	188	192
253	257	214	214	139	145	165	165			
MASB19	MASB	-73.28606667		42.26363333		230	230	187	187	202
204	160	174	132	132	170	188	193	195	188	198
255	255	212	212	139	145	163	163			
MASB20	MASB	-73.28606667		42.26363333		230	230	187	187	170
206	158	180	128	134	170	188	193	195	190	194
255	255	212	212	135	145	163	163			
MASB21	MASB	-73.28606667		42.26363333		230	230	187	187	170
204	160	174	132	146	170	188	195	197	160	188
255	255	212	214	139	145	163	165			
MASB22	MASB	-73.28606667		42.26363333		228	230	187	189	202
212	190	200	130	136	170	188	193	195	192	206
255	257	216	216	137	139	167	167			
MASB23	MASB	-73.28606667		42.26363333		230	230	187	187	170

206	160	166	130	142	168	186	195	195	192	206
255	255	212	212	141	145	163	163			
MASB24	MASB	-73.28606667		42.26363333		230	230	187	187	204
204	158	192	130	132	168	186	193	195	188	188
253	253	212	212	139	139	163	163			
MASB25	MASB	-73.28606667		42.26363333		228	230	187	187	188
190	160	192	126	132	168	188	193	195	160	188
255	259	214	214	141	145	165	165			
MASB26	MASB	-73.28606667		42.26363333		230	230	187	187	188
204	160	174	130	132	170	188	193	193	162	192
261	261	212	214	139	141	163	165			
MASB27	MASB	-73.28606667		42.26363333		230	230	187	189	182
208	158	174	130	132	170	188	193	193	194	200
261	261	212	214	139	145	163	165			
MASB28	MASB	-73.28606667		42.26363333		232	232	187	189	190
206	158	172	140	146	170	188	193	193	164	206
259	261	212	212	141	145	163	163			
MASB29	MASB	-73.28606667		42.26363333		230	232	187	189	182
202	158	176	134	134	172	190	193	197	190	198
259	259	212	214	141	145	163	165			
MASB30	MASB	-73.28606667		42.26363333		230	232	187	187	210
210	158	158	132	136	172	190	193	193	192	212
257	273	212	214	139	145	163	165			
MASB31	MASB	-73.28606667		42.26363333		232	232	179	189	182
182	192	196	132	146	172	196	189	191	162	194
259	259	212	214	137	137	163	165			
MASB32	MASB	-73.28606667		42.26363333		232	232	187	189	194
206	158	174	132	134	170	188	189	191	178	190
259	259	212	214	139	145	163	165			
MASB33	MASB	-73.28606667		42.26363333		232	232	187	187	198
210	158	160	146	154	170	188	191	191	160	188
259	259	214	214	139	145	165	165			
MASB34	MASB	-73.28606667		42.26363333		232	232	187	189	208
208	166	174	148	154	170	188	191	191	162	190
259	259	212	212	141	145	163	163			
MASB35	MASB	-73.28606667		42.26363333		232	232	187	189	192
192	158	174	122	134	172	190	189	193	162	192
259	259	212	212	139	145	163	163			
MASB36	MASB	-73.28606667		42.26363333		232	232	189	191	182
222	168	172	130	134	172	190	197	197	162	192
259	259	212	212	137	139	163	163			
MASB37	MASB	-73.28606667		42.26363333		232	232	187	189	208
208	160	174	132	140	172	190	195	195	190	200
253	277	214	214	141	145	165	165			
MASB38	MASB	-73.28606667		42.26363333		232	232	187	199	194
208	166	168	120	132	172	190	195	197	192	196
253	253	212	214	141	145	163	165			
MASB39	MASB	-73.28606667		42.26363333		232	238	191	199	208
208	174	194	128	130	172	196	195	197	192	192
253	253	212	214	141	145	163	165			
MASB40	MASB	-73.28606667		42.26363333		220	232	187	201	196
208	158	174	130	148	158	190	187	191	162	196
253	255	214	214	139	145	165	165			
MASB41	MASB	-73.28606667		42.26363333		232	232	187	189	192
192	160	174	132	132	172	190	187	197	162	194
253	253	212	212	139	145	163	163			
MASB42	MASB	-73.28606667		42.26363333		232	232	189	189	208
208	158	190	132	134	172	190	189	193	202	202
253	253	212	214	139	139	163	165			
MASB43	MASB	-73.28606667		42.26363333		234	234	189	191	186
210	174	192	132	132	172	190	191	193	192	200
253	257	212	212	139	139	163	163			
MASB44	MASB	-73.28606667		42.26363333		234	240	189	191	210

210	174	192	128	130	174	198	191	201	162	192
261	263	212	212	139	145	163	163			
MASB45	MASB	-73.28606667		42.26363333		232	240	189	191	192
212	160	194	136	146	172	198	187	191	190	202
261	261	212	212	139	145	163	163			
MASB46	MASB	-73.28606667		42.26363333		232	232	187	191	208
208	158	190	134	134	174	192	189	191	190	200
261	261	212	212	139	145	163	163			
MASB47	MASB	-73.28606667		42.26363333		232	238	199	199	210
212	156	194	130	130	172	198	187	191	162	194
261	261	212	212	141	145	163	163			
MASB48	MASB	-73.28606667		42.26363333		226	232	187	189	184
210	172	190	132	134	164	190	187	195	162	192
259	261	212	212	137	137	163	163			
MASB49	MASB	-73.28606667		42.26363333		230	232	187	189	194
198	158	190	128	132	170	188	189	195	194	200
259	259	212	214	139	145	163	165			
MASB50	MASB	-73.28606667		42.26363333		230	230	187	189	210
210	160	174	132	132	170	188	189	197	162	198
255	255	212	212	139	145	163	163			
NYCM01	NYCM	-74.16803889		41.9465	228	228	177	185	190	190
158	186	138	142	182	194	193	201	192	196	243
257	212	212	143	145	163	163				
NYCM02	NYCM	-74.16803889		41.9465	228	230	185	187	204	208
158	192	132	134	180	184	189	191	192	196	243
257	206	214	139	145	157	165				
NYCM03	NYCM	-74.16803889		41.9465	228	228	187	187	202	210
160	164	132	134	162	184	191	197	200	208	243
253	216	220	139	145	167	171				
NYCM04	NYCM	-74.16803889		41.9465	228	230	177	191	204	204
154	158	138	144	182	194	189	191	192	196	239
257	220	220	137	143	171	171				
NYCM05	NYCM	-74.16803889		41.9465	228	228	191	193	184	208
158	174	144	144	182	186	191	193	184	202	237
253	214	216	141	145	165	167				
NYCM06	NYCM	-74.16803889		41.9465	230	234	185	191	190	210
158	176	138	144	184	184	191	191	194	194	239
253	216	216	137	145	167	167				
NYCM07	NYCM	-74.16803889		41.9465	228	228	175	185	172	208
158	194	122	138	160	184	171	193	164	194	239
257	216	216	141	143	167	167				
NYCM08	NYCM	-74.16803889		41.9465	228	230	175	189	210	210
160	182	134	134	162	182	191	193	194	196	239
259	218	220	141	145	169	171				
NYCM09	NYCM	-74.16803889		41.9465	228	230	177	187	204	208
166	166	126	128	186	196	191	193	192	204	239
245	218	220	141	143	169	171				
NYCM10	NYCM	-74.16803889		41.9465	230	230	187	189	182	186
160	176	128	128	154	186	191	191	202	202	239
245	218	220	143	143	169	171				
NYCM11	NYCM	-74.16803889		41.9465	230	230	187	193	208	212
160	198	132	132	188	188	191	193	196	198	237
251	218	220	139	145	169	171				
NYCM12	NYCM	-74.16803889		41.9465	230	232	173	187	210	214
160	176	132	132	160	186	191	193	166	192	241
253	218	224	141	145	169	175				
NYCM13	NYCM	-74.16803889		41.9465	230	230	187	187	208	208
162	180	128	130	184	188	189	191	166	202	241
257	220	220	139	145	171	171				
NYCM14	NYCM	-74.16803889		41.9465	230	236	187	189	192	210
160	160	128	132	180	186	189	191	190	202	241
257	220	220	139	145	171	171				
NYCM15	NYCM	-74.16803889		41.9465	230	230	187	187	192	194

162	178	130	132	164	184	193	195	192	196	241
257	220	222	143	143	171	173				
NYCM16	NYCM	-74.16803889	41.9465	230	230	187	187	192	210	
160	178	128	132	180	184	193	195	194	200	239
257	218	222	143	145	169	173				
NYCM17	NYCM	-74.16803889	41.9465	230	230	189	193	192	202	
160	192	154	154	184	186	193	193	192	198	241
257	220	222	137	139	171	173				
NYCM18	NYCM	-74.16803889	41.9465	230	230	177	187	206	208	
160	168	154	154	184	198	193	193	194	194	239
257	218	222	143	145	169	173				
NYCM19	NYCM	-74.16803889	41.9465	230	230	189	191	192	208	
162	178	132	132	184	184	193	195	200	202	241
249	218	222	139	145	169	173				
NYCM20	NYCM	-74.16803889	41.9465	228	228	187	195	208	212	
158	176	130	130	184	186	193	195	194	194	239
257	216	220	135	145	167	171				
NYCM21	NYCM	-74.16803889	41.9465	222	228	179	187	188	208	
160	162	124	130	184	186	195	197	190	190	237
261	218	218	139	145	169	169				
NYCM22	NYCM	-74.16803889	41.9465	228	228	179	187	192	214	
156	166	124	146	182	186	193	195	194	196	239
261	218	218	141	143	169	169				
NYCM23	NYCM	-74.16803889	41.9465	222	228	185	189	208	212	
160	174	118	130	184	184	193	193	194	196	239
257	220	220	141	145	171	171				
NYCM24	NYCM	-74.16803889	41.9465	228	228	185	185	176	186	
160	166	120	130	180	186	191	193	194	196	237
257	218	220	139	139	169	171				
NYCM25	NYCM	-74.16803889	41.9465	228	228	177	187	206	210	
160	190	136	140	182	186	191	191	192	200	239
255	218	218	141	145	169	169				
NYCM26	NYCM	-74.16803889	41.9465	230	230	177	189	186	210	
162	178	136	138	162	184	189	191	192	192	241
255	218	222	139	141	169	173				
NYCM27	NYCM	-74.16803889	41.9465	230	230	187	187	186	208	
162	178	134	140	156	184	189	193	198	200	241
251	220	222	139	145	171	173				
NYCM28	NYCM	-74.16803889	41.9465	230	236	187	187	178	208	
162	176	132	134	186	186	193	195	202	202	239
253	222	222	141	145	173	173				
NYCM29	NYCM	-74.16803889	41.9465	230	230	187	187	208	222	
158	166	132	136	164	186	191	193	192	194	237
255	222	226	141	145	173	177				
NYCM30	NYCM	-74.16803889	41.9465	228	230	187	187	206	210	
162	166	132	132	164	184	189	191	192	196	237
257	222	226	143	145	173	177				
NYCM31	NYCM	-74.16803889	41.9465	228	230	187	189	178	182	
160	174	134	138	186	190	189	193	192	202	241
257	224	226	137	137	175	177				
NYCM32	NYCM	-74.16803889	41.9465	228	230	185	187	190	208	
162	174	128	134	180	186	193	193	192	198	239
253	222	224	139	145	173	175				
NYCM33	NYCM	-74.16803889	41.9465	230	236	185	193	204	208	
174	178	132	134	156	182	195	195	192	202	239
251	220	220	139	145	171	171				
NYCM34	NYCM	-74.16803889	41.9465	224	230	185	193	190	192	
162	176	132	134	160	184	197	197	192	200	243
253	220	220	141	143	171	171				
NYCM35	NYCM	-74.16803889	41.9465	230	230	185	187	192	192	
158	176	128	130	180	188	195	197	188	192	243
253	232	240	139	145	167	169				
NYCM36	NYCM	-74.16803889	41.9465	232	232	187	189	192	208	

162	172	130	134	182	186	197	197	190	194	243
253	222	232	137	139	173	183				
NYCM37	NYCM	-74.16803889	41.9465	232	248	185	187	192	210	
172	180	134	148	158	186	195	195	190	192	243
253	222	224	141	145	173	175				
NYCM38	NYCM	-74.16803889	41.9465	230	230	185	185	192	208	
158	178	130	130	184	186	195	197	192	192	243
253	222	224	143	145	173	175				
NYCM39	NYCM	-74.16803889	41.9465	230	230	183	187	192	206	
158	176	130	130	182	186	189	193	190	204	243
257	222	224	141	145	173	175				
NYCM40	NYCM	-74.16803889	41.9465	230	230	185	185	208	212	
158	174	128	132	162	186	189	193	190	194	243
257	218	220	139	145	169	171				
NYCM41	NYCM	-74.16803889	41.9465	230	232	185	187	206	208	
162	182	132	132	162	184	191	193	162	194	231
257	218	220	143	145	169	171				
NYCM42	NYCM	-74.16803889	41.9465	230	230	185	189	208	212	
160	172	126	126	132	132	191	193	198	202	231
257	218	220	139	139	169	171				
NYCM43	NYCM	-74.16803889	41.9465	230	232	183	187	206	224	
158	176	128	132	202	204	189	193	198	198	239
257	216	220	143	139	167	171				
NYCM44	NYCM	-74.16803889	41.9465	230	232	185	187	208	220	
160	172	132	132	182	182	191	191	190	190	239
259	216	216	139	145	167	167				
NYCM45	NYCM	-74.16803889	41.9465	230	230	185	187	206	214	
160	162	130	132	162	162	191	191	188	196	233
243	216	216	139	145	167	167				
NYCM46	NYCM	-74.16803889	41.9465	230	230	185	187	192	220	
158	162	132	154	182	182	195	197	188	196	239
259	216	216	143	145	167	167				
NYCM47	NYCM	-74.16803889	41.9465	230	230	185	185	208	220	
160	162	128	128	162	194	195	195	194	194	239
263	214	214	141	145	165	165				
NYCM48	NYCM	-74.16803889	41.9465	230	230	185	185	172	172	
158	162	126	130	184	184	195	195	186	186	243
257	216	216	141	137	167	167				
NYCM49	NYCM	-74.16803889	41.9465	230	230	181	183	206	212	
172	172	136	138	180	180	193	193	186	186	243
257	214	214	139	145	165	165				
NYCM50	NYCM	-74.16803889	41.9465	230	230	179	185	206	214	
158	174	132	134	178	192	193	193	184	184	239
249	212	212	141	143	163	163				
NHDF01	NHDF	-71.26134722	43.10903889	228	228	183	187	204		
210	166	168	138	142	166	168	193	201	186	188
257	257	214	218	141	143	165	169			
NHDF02	NHDF	-71.26134722	43.10903889	230	230	185	185	190		
208	166	166	142	144	168	168	191	193	186	194
259	259	214	218	139	145	165	169			
NHDF03	NHDF	-71.26134722	43.10903889	228	230	175	185	188		
200	166	166	138	156	166	168	193	203	186	200
255	255	214	218	139	145	165	169			
NHDF04	NHDF	-71.26134722	43.10903889	230	230	185	187	202		
208	166	168	160	160	166	168	193	195	188	192
255	261	212	216	137	143	163	167			
NHDF05	NHDF	-71.26134722	43.10903889	230	230	183	183	200		
208	166	168	130	144	168	168	193	195	190	194
255	267	212	216	141	145	163	167			
NHDF06	NHDF	-71.26134722	43.10903889	230	230	183	185	198		
206	166	166	146	148	166	168	193	193	194	196
255	259	214	216	137	145	165	167			
NHDF07	NHDF	-71.26134722	43.10903889	230	230	185	187	202		

208	164	166	156	158	166	166	193	195	190	196
257	263	212	220	141	145	163	171			
NHDF08	NHDF	-71.26134722		43.10903889		228	230	183	191	200
210	166	166	154	154	166	168	193	195	188	190
257	257	214	216	141	145	165	167			
NHDF09	NHDF	-71.26134722		43.10903889		230	236	183	183	204
206	166	166	154	154	166	168	193	195	188	196
255	257	216	218	141	145	167	169			
NHDF10	NHDF	-71.26134722		43.10903889		230	236	183	191	200
204	166	166	158	160	166	168	193	195	188	188
255	259	212	214	137	141	163	165			
NHDF11	NHDF	-71.26134722		43.10903889		230	230	185	189	202
204	164	166	132	136	168	168	193	195	186	188
255	259	216	218	139	145	167	169			
NHDF12	NHDF	-71.26134722		43.10903889		230	236	185	187	202
204	164	166	154	156	166	166	193	193	188	204
255	257	212	218	141	145	163	169			
NHDF13	NHDF	-71.26134722		43.10903889		230	230	185	193	200
200	164	166	138	138	164	168	193	195	192	196
253	261	214	218	139	145	165	169			
NHDF14	NHDF	-71.26134722		43.10903889		234	238	185	185	202
204	166	166	138	142	168	168	193	193	192	192
257	273	216	218	141	141	167	169			
NHDF15	NHDF	-71.26134722		43.10903889		228	230	175	187	204
204	168	170	120	160	166	170	193	195	188	202
255	259	214	216	141	143	165	167			
NHDF16	NHDF	-71.26134722		43.10903889		230	236	183	185	198
202	170	174	138	138	160	166	193	195	186	188
257	273	214	216	141	145	165	167			
NHDF17	NHDF	-71.26134722		43.10903889		230	236	175	187	172
206	166	168	138	156	168	168	193	193	186	196
255	257	214	216	137	139	165	167			
NHDF18	NHDF	-71.26134722		43.10903889		230	230	173	185	188
202	166	168	120	138	166	166	193	193	190	192
255	255	214	220	139	145	165	171			
NHDF19	NHDF	-71.26134722		43.10903889		230	230	185	187	206
210	164	166	138	156	166	166	193	195	188	200
255	255	212	220	141	145	163	171			
NHDF20	NHDF	-71.26134722		43.10903889		230	230	183	187	196
208	168	174	152	154	166	168	193	195	188	188
255	255	214	220	141	145	165	171			
NHDF21	NHDF	-71.26134722		43.10903889		230	230	185	187	206
210	166	168	154	156	162	166	195	197	188	194
255	255	214	218	139	145	165	169			
NHDF22	NHDF	-71.26134722		43.10903889		228	230	183	185	198
212	166	168	156	158	166	168	193	195	196	196
255	257	214	218	137	139	165	169			
NHDF23	NHDF	-71.26134722		43.10903889		230	230	183	187	200
212	168	170	136	156	166	166	195	195	188	206
255	255	216	220	141	145	167	171			
NHDF24	NHDF	-71.26134722		43.10903889		230	230	183	187	180
206	168	170	142	150	166	166	193	195	194	196
253	253	214	220	139	139	165	171			
NHDF25	NHDF	-71.26134722		43.10903889		228	230	183	185	190
204	168	170	136	136	164	164	193	195	184	186
255	259	214	218	141	145	165	169			
NHDF26	NHDF	-71.26134722		43.10903889		230	230	183	183	188
202	168	170	136	156	166	166	193	193	184	188
257	259	214	218	139	141	165	169			
NHDF27	NHDF	-71.26134722		43.10903889		230	230	169	183	190
202	166	168	154	154	168	170	193	193	190	196
257	257	214	220	141	145	165	171			
NHDF28	NHDF	-71.26134722		43.10903889		230	230	183	185	190

190	164	166	138	142	168	168	193	193	196	198
257	259	216	220	141	145	167	171			
NHDF29	NHDF	-71.26134722		43.10903889		230	230	183	191	192
196	166	166	138	154	168	170	193	197	190	196
259	259	214	218	141	145	165	169			
NHDF30	NHDF	-71.26134722		43.10903889		232	232	185	187	176
188	166	174	154	156	166	166	193	193	190	192
259	259	214	216	139	145	165	167			
NHDF31	NHDF	-71.26134722		43.10903889		230	232	185	187	188
190	164	166	156	156	166	166	193	197	186	196
259	259	214	218	137	137	165	169			
NHDF32	NHDF	-71.26134722		43.10903889		214	214	183	185	188
204	166	168	138	156	168	170	193	193	194	194
259	259	214	216	143	145	165	167			
NHDF33	NHDF	-71.26134722		43.10903889		218	230	183	185	196
204	166	166	138	142	168	168	195	195	186	186
259	259	216	216	139	145	167	167			
NHDF34	NHDF	-71.26134722		43.10903889		224	230	181	183	198
202	166	166	126	126	168	170	197	197	186	194
259	259	214	216	141	145	165	167			
NHDF35	NHDF	-71.26134722		43.10903889		214	214	185	185	188
202	166	168	138	138	168	170	195	197	190	204
253	253	212	216	139	145	163	167			
NHDF36	NHDF	-71.26134722		43.10903889		246	246	185	187	188
206	166	168	154	158	168	168	197	197	180	196
253	253	214	216	137	139	165	167			
NHDF37	NHDF	-71.26134722		43.10903889		232	232	183	187	184
192	166	170	156	156	168	170	195	195	186	196
253	253	212	216	143	145	163	167			
NHDF38	NHDF	-71.26134722		43.10903889		232	232	185	185	188
206	166	168	134	154	170	170	195	197	186	190
253	253	212	218	141	145	163	169			
NHDF39	NHDF	-71.26134722		43.10903889		232	238	183	185	184
190	166	168	152	156	170	170	195	197	184	190
253	271	212	222	141	145	163	173			
NHDF40	NHDF	-71.26134722		43.10903889		220	232	183	183	172
202	164	168	138	156	170	170	195	195	190	194
255	269	214	218	141	145	165	169			
NHDF41	NHDF	-71.26134722		43.10903889		232	232	183	185	202
208	166	170	138	156	170	170	193	195	190	196
259	259	208	220	139	145	159	171			
NHDF42	NHDF	-71.26134722		43.10903889		232	232	183	185	188
200	166	168	156	156	166	170	195	195	194	196
259	259	214	216	139	139	165	167			
NHDF43	NHDF	-71.26134722		43.10903889		222	222	185	187	204
206	166	168	158	160	168	168	195	197	188	196
261	261	214	214	143	139	165	165			
NHDF44	NHDF	-71.26134722		43.10903889		234	240	181	185	186
188	166	170	158	160	168	168	197	197	186	190
261	263	212	216	139	145	163	167			
NHDF45	NHDF	-71.26134722		43.10903889		232	240	183	185	196
200	166	168	120	136	168	168	195	197	188	190
261	261	212	216	139	145	163	167			
NHDF46	NHDF	-71.26134722		43.10903889		232	232	183	183	198
200	166	170	138	138	168	168	195	197	192	202
261	261	216	216	143	143	167	167			
NHDF47	NHDF	-71.26134722		43.10903889		232	238	183	185	198
206	152	168	136	156	168	168	195	195	184	188
261	261	214	216	141	145	165	167			
NHDF48	NHDF	-71.26134722		43.10903889		226	232	185	185	198
208	160	166	154	156	168	170	195	195	186	186
259	261	214	216	141	141	165	167			
NHDF49	NHDF	-71.26134722		43.10903889		230	232	183	185	196

208	166	168	158	158	160	170	193	193	184	188
259	259	212	218	139	145	163	169			
NHDF50	NHDF	-71.26134722		43.10903889		230	230	183	185	196
206	168	168	138	146	160	162	193	193	188	194
255	255	218	218	139	145	169	169			
MNBL01	MNBL	-93.12775		45.32922222		226	232	197	197	190
206	156	162	120	130	164	166	191	195	188	204
255	257	214	218	145	149	151	165			
MNBL02	MNBL	-93.12775		45.32922222		228	230	195	199	190
206	148	152	120	130	166	168	189	193	206	206
257	261	200	220	143	147	165	165			
MNBL03	MNBL	-93.12775		45.32922222		224	230	195	195	190
212	134	146	116	126	164	168	191	193	160	200
257	261	218	222	149	149	165	165			
MNBL04	MNBL	-93.12775		45.32922222		228	232	183	195	182
194	146	148	122	124	182	184	193	195	160	192
275	295	214	218	149	153	165	165			
MNBL05	MNBL	-93.12775		45.32922222		228	234	183	195	190
214	152	152	126	126	164	168	189	201	158	192
257	259	214	218	147	151	165	167			
MNBL06	MNBL	-93.12775		45.32922222		230	232	183	195	208
212	148	152	122	126	166	168	191	193	190	194
257	259	214	220	143	145	163	167			
MNBL07	MNBL	-93.12775		45.32922222		228	234	195	195	188
206	148	154	122	126	164	164	187	191	160	188
255	255	214	218	147	149	165	165			
MNBL08	MNBL	-93.12775		45.32922222		228	228	193	195	212
212	150	156	118	118	164	166	189	193	188	194
255	255	214	216	145	149	165	167			
MNBL09	MNBL	-93.12775		45.32922222		228	230	195	197	208
212	150	154	124	126	166	168	191	193	190	194
257	261	212	216	145	149	163	167			
MNBL10	MNBL	-93.12775		45.32922222		226	230	193	195	206
212	152	158	124	126	164	168	189	193	188	194
255	259	214	218	147	149	165	167			
MNBL11	MNBL	-93.12775		45.32922222		228	228	195	195	204
208	144	148	122	124	164	166	191	193	188	190
255	255	200	220	141	145	151	167			
MNBL12	MNBL	-93.12775		45.32922222		226	230	193	199	188
204	132	146	114	126	164	166	185	193	158	188
255	259	218	222	149	149	165	167			
MNBL13	MNBL	-93.12775		45.32922222		228	230	195	195	196
202	146	150	124	126	166	168	189	191	184	188
255	257	214	218	143	147	165	167			
MNBL14	MNBL	-93.12775		45.32922222		228	228	195	195	200
212	146	160	124	124	166	168	187	191	188	188
159	253	214	218	149	149	151	167			
MNBL15	MNBL	-93.12775		45.32922222		230	230	193	197	198
206	146	150	122	126	170	172	187	189	184	190
255	259	214	220	149	153	165	167			
MNBL16	MNBL	-93.12775		45.32922222		224	230	197	199	200
206	130	144	124	126	156	168	187	189	158	188
255	259	214	218	147	151	165	167			
MNBL17	MNBL	-93.12775		45.32922222		226	228	195	251	198
202	146	146	124	126	168	170	191	191	188	190
253	293	214	218	143	145	165	167			
MNBL18	MNBL	-93.12775		45.32922222		226	228	195	197	200
204	146	160	112	122	156	168	189	193	194	196
255	257	200	220	149	153	151	171			
MNBL19	MNBL	-93.12775		45.32922222		226	230	195	199	176
188	132	146	124	124	156	168	187	191	160	194
255	257	218	222	147	151	169	173			
MNBL20	MNBL	-93.12775		45.32922222		226	230	195	197	176

188	146	148	124	126	166	170	189	199	188	198
253	257	214	218	143	145	165	167			
MNBL21	MNBL	-93.12775		45.32922222		228	228	195	195	194
200	144	150	120	128	166	168	191	197	158	188
253	253	214	218	147	149	151	167			
MNBL22	MNBL	-93.12775		45.32922222		226	228	195	195	202
214	146	146	112	124	166	168	191	201	160	190
253	253	214	220	143	149	165	169			
MNBL23	MNBL	-93.12775		45.32922222		228	232	195	197	212
216	132	146	124	128	166	168	189	193	160	194
253	253	214	218	149	151	165	167			
MNBL24	MNBL	-93.12775		45.32922222		228	230	197	197	206
216	144	148	124	126	166	168	191	193	118	194
253	257	214	216	147	151	165	167			
MNBL25	MNBL	-93.12775		45.32922222		222	228	195	197	202
210	146	160	124	124	166	170	187	193	200	204
253	255	216	218	149	151	165	167			
MNBL26	MNBL	-93.12775		45.32922222		228	228	195	197	190
202	146	152	126	126	166	170	189	193	194	198
253	253	200	218	143	145	165	167			
MNBL27	MNBL	-93.12775		45.32922222		230	234	195	197	160
190	148	152	122	128	164	168	191	193	188	194
255	257	214	216	149	149	151	167			
MNBL28	MNBL	-93.12775		45.32922222		228	234	195	197	190
190	144	148	122	128	166	168	189	195	160	190
253	257	214	218	147	149	165	169			
MNBL29	MNBL	-93.12775		45.32922222		224	228	195	195	200
208	130	144	122	126	146	168	191	191	160	192
253	253	214	218	147	153	165	167			
MNBL30	MNBL	-93.12775		45.32922222		226	230	193	195	200
214	130	144	124	126	168	172	191	201	160	192
253	253	214	216	145	151	165	167			
MNBL31	MNBL	-93.12775		45.32922222		228	230	193	195	204
214	130	144	122	128	168	170	191	191	162	194
253	253	214	216	145	149	165	169			
MNBL32	MNBL	-93.12775		45.32922222		226	230	193	197	204
208	132	144	128	128	168	170	193	193	162	196
255	255	212	214	143	149	165	167			
MNBL33	MNBL	-93.12775		45.32922222		228	228	183	195	200
208	152	158	124	126	166	170	193	201	198	206
253	253	216	218	149	151	167	169			
MNBL34	MNBL	-93.12775		45.32922222		222	228	195	197	208
214	152	158	126	268	164	166	191	191	186	188
253	253	200	218	147	151	151	169			
MNBL35	MNBL	-93.12775		45.32922222		228	228	195	197	202
208	146	150	124	124	166	168	191	191	192	194
253	253	214	216	149	151	165	169			
MNBL36	MNBL	-93.12775		45.32922222		228	232	193	197	204
214	146	150	124	126	166	166	191	195	194	204
253	253	214	218	143	145	165	165			
MNBL37	MNBL	-93.12775		45.32922222		228	228	195	197	208
208	152	160	126	126	164	166	191	191	188	198
253	253	214	218	149	149	165	167			
MNBL38	MNBL	-93.12775		45.32922222		228	228	181	195	176
202	144	144	126	128	166	168	193	193	190	198
253	253	214	216	149	151	163	165			
MNBL39	MNBL	-93.12775		45.32922222		228	228	195	197	208
208	144	148	124	126	164	168	191	195	188	188
253	253	214	216	147	151	163	165			
MNBL40	MNBL	-93.12775		45.32922222		226	228	195	197	200
204	144	148	114	126	166	168	187	195	194	194
253	257	212	214	147	153	163	165			
MNBL41	MNBL	-93.12775		45.32922222		224	228	165	195	196

198	146	160	124	128	166	168	117	191	160	190
251	287	214	216	147	153	165	167			
MNBL42	MNBL	-93.12775		45.32922222		226	228	193	195	198
202	146	148	126	128	166	168	191	195	186	190
253	257	214	216	141	145	165	167			
MNBL43	MNBL	-93.12775		45.32922222		228	232	193	195	194
198	146	146	124	128	164	168	191	195	188	188
251	255	214	218	149	153	165	169			
MNBL44	MNBL	-93.12775		45.32922222		228	228	191	195	196
198	146	160	124	128	166	168	187	195	194	194
251	287	214	214	149	153	165	165			
MNBL45	MNBL	-93.12775		45.32922222		228	228	195	197	198
202	146	148	126	128	166	168	117	191	160	190
253	257	214	216	149	153	163	165			
MNBL46	MNBL	-93.12775		45.32922222		228	228	193	197	194
198	146	146	124	128	166	168	191	195	186	190
251	255	214	218	147	153	163	165			
MNBL47	MNBL	-93.12775		45.32922222		226	228	193	195	196
198	146	160	124	128	164	168	191	195	188	188
251	287	214	214	147	153	163	169			
MNBL48	MNBL	-93.12775		45.32922222		224	228	195	197	198
202	146	148	126	128	166	168	187	195	194	194
253	257	214	216	141	145	163	165			
MNBL49	MNBL	-93.12775		45.32922222		226	228	195	197	194
198	146	146	124	128	166	168	117	191	160	190
251	255	212	214	147	153	163	165			
MNBL50	MNBL	-93.12775		45.32922222		226	228	191	195	194
198	146	146	124	128	166	168	191	195	186	190
251	255	212	214	149	153	163	169			
VABS01	VASB	-80.01676111		37.38003611		226	226	183	185	196
206	160	174	132	134	178	178	193	193	186	190
253	267	212	212	137	143	165	165			
VABS02	VASB	-80.01676111		37.38003611		230	230	173	185	204
214	168	168	130	140	162	164	189	189	186	186
255	271	210	214	137	143	163	165			
VABS03	VASB	-80.01676111		37.38003611		232	236	181	187	212
212	168	172	134	144	164	164	189	189	192	194
259	265	210	210	141	141	161	161			
VABS04	VASB	-80.01676111		37.38003611		226	228	185	189	202
204	148	156	150	154	160	164	187	187	188	194
261	263	212	216	143	143	167	169			
VABS05	VASB	-80.01676111		37.38003611		228	228	181	197	204
208	156	156	154	154	162	182	187	189	186	190
253	253	212	214	135	141	165	169			
VABS06	VASB	-80.01676111		37.38003611		228	234	173	187	182
204	154	168	152	152	172	190	189	193	188	190
257	257	212	214	135	143	169	173			
VABS07	VASB	-80.01676111		37.38003611		228	228	183	185	194
194	156	156	120	124	160	164	193	195	192	198
257	259	212	212	137	141	163	167			
VABS08	VASB	-80.01676111		37.38003611		228	228	181	181	192
196	158	174	130	132	166	166	193	195	190	192
251	251	210	214	137	145	165	169			
VABS09	VASB	-80.01676111		37.38003611		230	236	183	191	204
210	154	174	124	124	168	186	191	191	194	194
253	253	212	214	137	143	163	167			
VABS10	VASB	-80.01676111		37.38003611		230	230	185	187	188
188	156	170	138	140	164	164	189	191	184	186
235	255	214	214	137	143	165	167			
VABS11	VASB	-80.01676111		37.38003611		230	230	179	179	186
188	166	170	114	146	180	184	185	193	194	194
251	251	210	210	137	143	163	163			
VABS12	VASB	-80.01676111		37.38003611		226	226	181	181	188

204	146	160	128	156	142	162	189	195	196	200
253	253	210	210	139	143	163	163			
VABS13	VASB	-80.01676111		37.38003611		224	224	183	187	204
210	164	168	120	124	162	166	187	193	190	192
253	271	214	216	141	141	163	163			
VABS14	VASB	-80.01676111		37.38003611		226	228	197	197	198
204	158	190	132	132	166	166	189	191	188	196
237	249	214	214	137	143	163	165			
VABS15	VASB	-80.01676111		37.38003611		232	232	183	187	200
200	166	168	126	130	180	180	187	187	190	202
257	257	214	216	141	145	165	167			
VABS16	VASB	-80.01676111		37.38003611		230	230	185	185	186
204	158	166	128	156	166	166	191	193	198	206
257	269	210	212	141	147	163	167			
VABS17	VASB	-80.01676111		37.38003611		224	224	169	183	178
202	160	176	124	126	160	164	191	193	186	192
255	255	214	214	139	143	165	167			
VABS18	VASB	-80.01676111		37.38003611		228	228	191	195	206
206	158	172	122	126	162	166	191	193	188	190
259	259	212	216	137	143	165	165			
VABS19	VASB	-80.01676111		37.38003611		232	232	185	185	214
214	168	168	146	152	162	162	189	189	190	190
251	251	212	214	141	141	165	165			
VABS20	VASB	-80.01676111		37.38003611		222	226	181	181	204
206	170	170	128	138	162	166	187	191	192	198
251	251	208	214	139	143	165	169			
VABS21	VASB	-80.01676111		37.38003611		228	232	185	197	212
214	158	172	128	130	164	166	189	191	186	198
253	253	212	212	143	145	161	165			
VABS22	VASB	-80.01676111		37.38003611		226	226	183	185	190
194	152	156	130	144	182	182	191	191	190	194
261	265	212	212	139	145	163	163			
VABS23	VASB	-80.01676111		37.38003611		226	226	177	183	198
204	166	168	128	130	180	192	189	189	190	192
255	259	212	212	139	139	165	165			
VABS24	VASB	-80.01676111		37.38003611		228	228	181	187	196
208	164	166	126	144	164	164	185	193	190	190
253	269	212	212	137	143	161	161			
VABS25	VASB	-80.01676111		37.38003611		226	226	179	179	194
194	152	152	126	128	172	192	191	193	188	190
253	253	212	216	141	147	161	163			
VABS26	VASB	-80.01676111		37.38003611		218	224	183	185	188
202	146	158	128	128	142	164	189	189	184	188
253	253	212	212	137	143	165	165			
VABS27	VASB	-80.01676111		37.38003611		224	230	185	185	202
202	148	172	126	130	164	166	189	191	190	200
253	253	212	214	139	145	165	165			
VABS28	VASB	-80.01676111		37.38003611		226	228	185	195	176
202	156	156	128	128	168	186	193	193	188	196
253	253	210	214	137	143	165	167			
VABS29	VASB	-80.01676111		37.38003611		228	228	181	185	176
208	156	158	130	130	162	164	191	193	184	186
253	257	212	212	137	143	163	167			
VABS30	VASB	-80.01676111		37.38003611		226	228	181	185	176
202	156	172	130	132	164	164	187	191	190	192
253	253	212	212	137	143	165	165			
VABS31	VASB	-80.01676111		37.38003611		230	230	185	185	200
206	144	156	136	136	142	166	189	191	186	194
255	257	202	214	139	143	165	165			
VABS32	VASB	-80.01676111		37.38003611		212	212	169	181	192
206	156	156	120	124	164	164	185	189	192	196
253	253	212	212	137	143	161	163			
VABS33	VASB	-80.01676111		37.38003611		226	228	183	185	206

206	156	172	130	134	164	164	193	193	186	192
259	259	212	216	137	143	167	169			
VABS34	VASB	-80.01676111		37.38003611		226	226	189	191	200
202	156	166	130	132	166	182	187	189	188	188
253	253	212	214	141	145	165	167			
VABS35	VASB	-80.01676111		37.38003611		230	230	183	189	206
208	166	170	134	134	164	164	189	191	184	188
251	251	216	216	137	143	167	169			
VABS36	VASB	-80.01676111		37.38003611		226	228	183	187	196
206	158	170	122	124	162	164	189	189	192	192
249	253	214	214	137	143	165	167			
VABS37	VASB	-80.01676111		37.38003611		224	230	181	183	170
200	164	164	126	134	164	166	189	189	158	184
253	253	210	212	143	143	163	163			
VABS38	VASB	-80.01676111		37.38003611		228	228	183	185	202
206	150	156	130	132	166	168	189	189	190	192
241	251	210	214	137	141	165	167			
VABS39	VASB	-80.01676111		37.38003611		228	230	181	187	188
204	156	190	132	136	162	166	193	195	188	198
239	249	210	212	143	145	161	161			
VABS40	VASB	-80.01676111		37.38003611		226	226	169	185	204
204	168	168	130	138	168	168	191	193	192	196
253	259	212	212	137	143	163	165			
VABS41	VASB	-80.01676111		37.38003611		228	228	185	187	172
202	158	164	126	132	164	164	191	193	190	198
239	255	212	212	137	143	163	167			
VABS42	VASB	-80.01676111		37.38003611		228	228	181	181	206
206	146	158	124	126	160	164	177	187	158	186
253	253	216	216	139	143	171	173			
VABS43	VASB	-80.01676111		37.38003611		226	232	179	183	180
200	154	172	130	134	138	164	193	193	192	192
253	253	208	212	137	143	165	169			
VABS44	VASB	-80.01676111		37.38003611		228	230	181	183	202
206	160	176	126	146	168	186	189	189	188	190
253	253	212	214	139	143	165	165			
VABS45	VASB	-80.01676111		37.38003611		220	220	183	185	180
180	162	190	124	128	164	164	187	191	186	188
261	263	212	214	139	143	165	169			
VABS46	VASB	-80.01676111		37.38003611		226	230	181	181	200
212	146	156	134	134	166	168	189	191	164	190
237	261	216	216	137	143	163	169			
VABS47	VASB	-80.01676111		37.38003611		228	230	185	191	188
192	170	170	130	134	162	162	187	187	166	192
257	257	214	214	135	143	163	167			
VABS48	VASB	-80.01676111		37.38003611		214	228	173	185	202
204	164	164	128	132	142	162	191	191	190	198
257	259	212	214	135	139	165	165			
VABS49	VASB	-80.01676111		37.38003611		226	226	183	183	190
210	156	156	130	132	164	166	191	193	190	192
253	259	216	218	147	149	165	165			
VABS50	VASB	-80.01676111		37.38003611		226	226	179	179	198
202	158	172	134	134	154	164	191	193	184	188
253	267	216	216	141	141	163	167			
NCAV01	NCAV	-82.53184167		35.61639722		234	234	187	189	196
210	168	202	128	132	168	168	195	195	196	204
257	257	194	218	141	149	151	167			
NCAV02	NCAV	-82.53184167		35.61639722		234	236	185	197	208
212	150	160	134	136	170	172	191	191	196	196
257	273	218	218	141	147	169	169			
NCAV03	NCAV	-82.53184167		35.61639722		234	236	185	197	200
200	172	172	132	138	168	172	193	193	196	202
255	257	216	216	141	147	169	171			
NCAV04	NCAV	-82.53184167		35.61639722		216	216	185	197	190

202	150	166	132	132	168	170	195	195	192	202
255	255	216	216	151	153	167	167			
NCAV05	NCAV	-82.53184167		35.61639722		240	240	189	191	186
192	150	162	128	132	168	168	191	193	190	192
257	271	218	218	141	147	167	171			
NCAV06	NCAV	-82.53184167		35.61639722		246	246	185	185	210
214	168	170	134	136	192	194	195	195	196	200
271	275	216	216	141	149	167	167			
NCAV07	NCAV	-82.53184167		35.61639722		234	238	197	197	200
204	148	176	130	150	144	168	191	191	200	206
261	261	NA	NA	141	147	169	171			
NCAV08	NCAV	-82.53184167		35.61639722		232	238	195	197	192
202	160	176	134	136	166	166	193	203	188	202
255	257	214	216	141	147	151	167			
NCAV09	NCAV	-82.53184167		35.61639722		232	232	197	199	190
222	146	158	134	134	158	168	199	207	194	202
257	263	216	216	143	147	169	171			
NCAV10	NCAV	-82.53184167		35.61639722		232	232	185	191	198
200	162	162	126	130	164	166	195	195	202	204
257	257	214	218	141	147	159	169			
NCAV11	NCAV	-82.53184167		35.61639722		230	230	177	187	206
206	162	176	132	132	178	180	197	199	166	166
257	257	216	218	139	139	165	165			
NCAV12	NCAV	-82.53184167		35.61639722		230	236	199	199	192
204	150	166	130	132	168	168	195	203	190	196
255	255	214	214	141	147	169	173			
NCAV13	NCAV	-82.53184167		35.61639722		230	230	197	201	196
208	160	176	130	132	166	168	193	193	166	192
241	263	216	218	139	147	167	167			
NCAV14	NCAV	-82.53184167		35.61639722		230	230	197	197	208
214	148	168	134	134	164	170	193	193	190	200
259	259	216	216	141	147	169	171			
NCAV15	NCAV	-82.53184167		35.61639722		228	228	171	181	188
212	160	162	134	134	168	168	189	195	198	200
263	263	212	216	145	145	167	169			
NCAV16	NCAV	-82.53184167		35.61639722		228	228	171	191	188
210	162	162	132	132	166	170	179	193	196	198
259	259	214	216	145	147	167	167			
NCAV17	NCAV	-82.53184167		35.61639722		230	236	185	191	190
212	160	162	124	124	172	176	189	195	190	192
257	259	216	218	141	147	169	169			
NCAV18	NCAV	-82.53184167		35.61639722		230	230	185	185	206
208	162	178	140	140	164	186	191	193	198	200
257	257	214	216	143	147	169	171			
NCAV19	NCAV	-82.53184167		35.61639722		228	228	185	187	206
206	162	176	128	132	168	168	197	197	192	194
255	255	216	216	137	147	167	167			
NCAV20	NCAV	-82.53184167		35.61639722		228	228	185	189	194
214	160	178	124	124	162	166	195	195	190	200
257	257	216	216	145	147	165	167			
NCAV21	NCAV	-82.53184167		35.61639722		228	228	185	189	198
202	164	164	130	136	160	168	197	199	194	196
255	271	216	218	143	145	167	167			
NCAV22	NCAV	-82.53184167		35.61639722		230	230	173	185	208
208	164	180	124	126	168	168	191	195	192	192
259	259	216	220	145	147	167	169			
NCAV23	NCAV	-82.53184167		35.61639722		230	230	175	185	208
208	176	176	124	128	190	194	209	209	190	190
253	253	216	216	141	147	169	171			
NCAV24	NCAV	-82.53184167		35.61639722		230	230	185	185	188
198	144	152	132	160	168	172	195	195	194	200
265	267	218	222	141	147	169	169			
NCAV25	NCAV	-82.53184167		35.61639722		230	230	185	185	190

208	168	170	126	130	168	168	119	193	188	190
245	263	216	218	143	147	167	167			
NCAV26	NCAV	-82.53184167		35.61639722		234	234	185	201	186
194	160	180	120	132	154	158	191	191	196	200
257	257	216	216	141	147	167	169			
NCAV27	NCAV	-82.53184167		35.61639722		230	234	185	203	208
212	158	158	132	136	174	192	191	197	164	194
259	263	216	218	141	147	167	169			
NCAV28	NCAV	-82.53184167		35.61639722		230	230	181	183	208
214	184	192	134	134	190	190	193	195	188	190
253	255	218	218	145	145	167	167			
NCAV29	NCAV	-82.53184167		35.61639722		228	232	173	185	194
206	160	184	142	142	168	170	193	193	196	196
259	259	216	218	141	147	171	175			
NCAV30	NCAV	-82.53184167		35.61639722		234	234	185	185	212
214	170	172	130	132	166	168	193	203	192	196
261	281	216	218	145	147	169	169			
NCAV31	NCAV	-82.53184167		35.61639722		230	230	185	185	212
212	162	174	130	132	146	170	193	197	198	204
255	255	200	216	141	147	167	171			
NCAV32	NCAV	-82.53184167		35.61639722		230	238	183	183	212
212	158	160	140	158	166	166	193	195	190	192
261	265	216	218	141	147	167	169			
NCAV33	NCAV	-82.53184167		35.61639722		232	232	187	189	210
212	158	176	140	144	168	168	179	193	192	196
257	261	214	220	145	149	169	171			
NCAV34	NCAV	-82.53184167		35.61639722		232	234	177	189	210
212	160	174	124	128	170	172	189	191	194	198
251	259	214	224	145	151	153	171			
NCAV35	NCAV	-82.53184167		35.61639722		232	236	177	191	192
192	160	174	130	132	168	170	191	193	174	188
257	259	216	216	145	145	167	169			
NCAV36	NCAV	-82.53184167		35.61639722		234	236	189	191	180
210	160	166	132	134	166	168	197	197	196	196
255	255	218	218	141	147	163	169			
NCAV37	NCAV	-82.53184167		35.61639722		234	234	187	189	210
224	170	174	130	134	168	194	191	191	202	208
257	261	220	222	141	147	171	173			
NCAV38	NCAV	-82.53184167		35.61639722		232	232	187	195	208
212	162	162	128	132	168	170	193	193	200	200
257	275	216	218	147	147	169	169			
NCAV39	NCAV	-82.53184167		35.61639722		232	232	185	203	180
184	168	174	132	134	168	170	195	195	202	206
259	259	216	216	149	151	167	167			
NCAV40	NCAV	-82.53184167		35.61639722		230	232	185	203	190
190	174	182	130	132	166	168	195	205	186	204
257	259	216	218	141	147	167	171			
NCAV41	NCAV	-82.53184167		35.61639722		232	232	187	203	212
212	164	178	132	134	170	172	193	205	194	198
255	255	216	216	145	151	171	171			
NCAV42	NCAV	-82.53184167		35.61639722		230	230	185	185	176
176	168	168	132	136	158	168	191	191	204	204
257	257	216	220	143	143	169	171			
NCAV43	NCAV	-82.53184167		35.61639722		230	230	181	183	172
174	170	172	126	130	168	172	193	195	194	200
257	257	214	216	145	149	165	165			
NCAV44	NCAV	-82.53184167		35.61639722		224	230	173	185	190
212	162	194	126	126	168	168	189	201	192	194
249	257	216	216	143	147	169	171			
NCAV45	NCAV	-82.53184167		35.61639722		230	230	185	185	208
210	158	162	132	138	168	168	193	195	192	196
255	255	216	218	139	139	165	167			
NCAV46	NCAV	-82.53184167		35.61639722		230	230	185	187	190

212	170	170	134	138	164	196	193	203	188	192
255	255	216	218	141	147	169	169			
NCAV47	NCAV	-82.53184167		35.61639722		230	230	183	189	212
216	176	176	126	128	170	188	193	193	192	192
253	257	218	220	145	145	169	171			
NCAV48	NCAV	-82.53184167		35.61639722		230	230	185	203	210
216	154	154	130	132	176	178	191	191	196	198
255	255	216	216	141	147	169	169			
NCAV49	NCAV	-82.53184167		35.61639722		228	228	187	203	204
208	142	148	132	132	166	170	201	203	192	192
257	257	220	222	151	155	167	169			
NCAV50	NCAV	-82.53184167		35.61639722		228	228	185	185	178
208	142	148	132	132	166	170	201	203	192	192
257	257	220	222	151	155	167	169			
