# Supplementary material for: Single-Locus versus Multilocus Patterns of Local Adaptation to Climate in Eastern White Pine (Pinus strobus, Pinaceae)
Source: PLoS One. 2016 Jul 7;11(7):e0158691. doi: 10.1371/journal.pone.0158691 (PMC4936701; doi:10.1371/journal.pone.0158691)
Supplement: S10 Table — (PDF) [file pone.0158691.s016.pdf]

Single-locus versus Multilocus Patterns of Local Adaptation to Climate in Eastern White Pine (Pinus strobus, Pinaceae)

Om P. Rajora, Andrew J. Eckert, and John W. R. Zinck

Bioclimatic Factors Data

Sample	Population	Longitude	Latitude	bio1	bio2	bio3	bio4	bio5	bio6	bio7	bio8	bio9	bio10	bio11	bio12	bio13	bio14	bio15	bio16	bio17	bio18	bio19
NBPH01	NBPMH	-65.29570278	45.78598611	49	109	28	9389	244	-139	383	-44	91	167	-76	1205	123	89	12	361	270	280	338
NBPH02	NBPMH	-65.29570278	45.78598611	49	109	28	9389	244	-139	383	-44	91	167	-76	1205	123	89	12	361	270	280	338
NBPH03	NBPMH	-65.29570278	45.78598611	49	109	28	9389	244	-139	383	-44	91	167	-76	1205	123	89	12	361	270	280	338
NBPH04	NBPMH	-65.29570278	45.78598611	49	109	28	9389	244	-139	383	-44	91	167	-76	1205	123	89	12	361	270	280	338
NBPH05	NBPMH	-65.29570278	45.78598611	49	109	28	9389	244	-139	383	-44	91	167	-76	1205	123	89	12	361	270	280	338
NBPH06	NBPMH	-65.29570278	45.78598611	49	109	28	9389	244	-139	383	-44	91	167	-76	1205	123	89	12	361	270	280	338
NBPH07	NBPMH	-65.29570278	45.78598611	49	109	28	9389	244	-139	383	-44	91	167	-76	1205	123	89	12	361	270	280	338
NBPH08	NBPMH	-65.29570278	45.78598611	49	109	28	9389	244	-139	383	-44	91	167	-76	1205	123	89	12	361	270	280	338
NBPH09	NBPMH	-65.29570278	45.78598611	49	109	28	9389	244	-139	383	-44	91	167	-76	1205	123	89	12	361	270	280	338
NBPH10	NBPMH	-65.29570278	45.78598611	49	109	28	9389	244	-139	383	-44	91	167	-76	1205	123	89	12	361	270	280	338
NBPH11	NBPMH	-65.29570278	45.78598611	49	109	28	9389	244	-139	383	-44	91	167	-76	1205	123	89	12	361	270	280	338
NBPH12	NBPMH	-65.29570278	45.78598611	49	109	28	9389	244	-139	383	-44	91	167	-76	1205	123	89	12	361	270	280	338
NBPH13	NBPMH	-65.29570278	45.78598611	49	109	28	9389	244	-139	383	-44	91	167	-76	1205	123	89	12	361	270	280	338
NBPH14	NBPMH	-65.29570278	45.78598611	49	109	28	9389	244	-139	383	-44	91	167	-76	1205	123	89	12	361	270	280	338
NBPH15	NBPMH	-65.29570278	45.78598611	49	109	28	9389	244	-139	383	-44	91	167	-76	1205	123	89	12	361	270	280	338
NBPH16	NBPMH	-65.29570278	45.78598611	49	109	28	9389	244	-139	383	-44	91	167	-76	1205	123	89	12	361	270	280	338
NBPH17	NBPMH	-65.29570278	45.78598611	49	109	28	9389	244	-139	383	-44	91	167	-76	1205	123	89	12	361	270	280	338
NBPH18	NBPMH	-65.29570278	45.78598611	49	109	28	9389	244	-139	383	-44	91	167	-76	1205	123	89	12	361			

270	280	338								
NBPH19	NBPMH	-65.29570278	45.78598611	49	109	28	9389	244		
-139	383	-44	91	167	-76	1205	123	89	12	361
270	280	338								
NBPH20	NBPMH	-65.29570278	45.78598611	49	109	28	9389	244		
-139	383	-44	91	167	-76	1205	123	89	12	361
270	280	338								
NBPH21	NBPMH	-65.29570278	45.78598611	49	109	28	9389	244		
-139	383	-44	91	167	-76	1205	123	89	12	361
270	280	338								
NBPH22	NBPMH	-65.29570278	45.78598611	49	109	28	9389	244		
-139	383	-44	91	167	-76	1205	123	89	12	361
270	280	338								
NBPH23	NBPMH	-65.29570278	45.78598611	49	109	28	9389	244		
-139	383	-44	91	167	-76	1205	123	89	12	361
270	280	338								
NBPH24	NBPMH	-65.29570278	45.78598611	49	109	28	9389	244		
-139	383	-44	91	167	-76	1205	123	89	12	361
270	280	338								
NBPH25	NBPMH	-65.29570278	45.78598611	49	109	28	9389	244		
-139	383	-44	91	167	-76	1205	123	89	12	361
270	280	338								
NBPH26	NBPMH	-65.29570278	45.78598611	49	109	28	9389	244		
-139	383	-44	91	167	-76	1205	123	89	12	361
270	280	338								
NBPH27	NBPMH	-65.29570278	45.78598611	49	109	28	9389	244		
-139	383	-44	91	167	-76	1205	123	89	12	361
270	280	338								
NBPH28	NBPMH	-65.29570278	45.78598611	49	109	28	9389	244		
-139	383	-44	91	167	-76	1205	123	89	12	361
270	280	338								
NBPH29	NBPMH	-65.29570278	45.78598611	49	109	28	9389	244		
-139	383	-44	91	167	-76	1205	123	89	12	361
270	280	338								
NBPH30	NBPMH	-65.29570278	45.78598611	49	109	28	9389	244		
-139	383	-44	91	167	-76	1205	123	89	12	361
270	280	338								
NBPH31	NBPMH	-65.29570278	45.78598611	49	109	28	9389	244		
-139	383	-44	91	167	-76	1205	123	89	12	361
270	280	338								
NBPH32	NBPMH	-65.29570278	45.78598611	49	109	28	9389	244		
-139	383	-44	91	167	-76	1205	123	89	12	361
270	280	338								
NBPH33	NBPMH	-65.29570278	45.78598611	49	109	28	9389	244		
-139	383	-44	91	167	-76	1205	123	89	12	361
270	280	338								
NBPH34	NBPMH	-65.29570278	45.78598611	49	109	28	9389	244		
-139	383	-44	91	167	-76	1205	123	89	12	361
270	280	338								
NBPH35	NBPMH	-65.29570278	45.78598611	49	109	28	9389	244		
-139	383	-44	91	167	-76	1205	123	89	12	361
270	280	338								
NBPH36	NBPMH	-65.29570278	45.78598611	49	109	28	9389	244		
-139	383	-44	91	167	-76	1205	123	89	12	361
270	280	338								
NBPH37	NBPMH	-65.29570278	45.78598611	49	109	28	9389	244		
-139	383	-44	91	167	-76	1205	123	89	12	361
270	280	338								
NBPH38	NBPMH	-65.29570278	45.78598611	49	109	28	9389	244		
-139	383	-44	91	167	-76	1205	123	89	12	361
270	280	338								
NBPH39	NBPMH	-65.29570278	45.78598611	49	109	28	9389	244		
-139	383	-44	91	167	-76	1205	123	89	12	361

270	280	338								
NBPH40	NBPMH	-65.29570278		45.78598611		49	109	28	9389	244
-139	383	-44	91	167	-76	1205	123	89	12	361
270	280	338								
NBPH41	NBPMH	-65.29570278		45.78598611		49	109	28	9389	244
-139	383	-44	91	167	-76	1205	123	89	12	361
270	280	338								
NBPH42	NBPMH	-65.29570278		45.78598611		49	109	28	9389	244
-139	383	-44	91	167	-76	1205	123	89	12	361
270	280	338								
NBPH43	NBPMH	-65.29570278		45.78598611		49	109	28	9389	244
-139	383	-44	91	167	-76	1205	123	89	12	361
270	280	338								
NBPH44	NBPMH	-65.29570278		45.78598611		49	109	28	9389	244
-139	383	-44	91	167	-76	1205	123	89	12	361
270	280	338								
NBPH45	NBPMH	-65.29570278		45.78598611		49	109	28	9389	244
-139	383	-44	91	167	-76	1205	123	89	12	361
270	280	338								
NBPH46	NBPMH	-65.29570278		45.78598611		49	109	28	9389	244
-139	383	-44	91	167	-76	1205	123	89	12	361
270	280	338								
NBPH47	NBPMH	-65.29570278		45.78598611		49	109	28	9389	244
-139	383	-44	91	167	-76	1205	123	89	12	361
270	280	338								
NBPH48	NBPMH	-65.29570278		45.78598611		49	109	28	9389	244
-139	383	-44	91	167	-76	1205	123	89	12	361
270	280	338								
NBPH49	NBPMH	-65.29570278		45.78598611		49	109	28	9389	244
-139	383	-44	91	167	-76	1205	123	89	12	361
270	280	338								
NBPH50	NBPMH	-65.29570278		45.78598611		49	109	28	9389	244
-139	383	-44	91	167	-76	1205	123	89	12	361
270	280	338								
NBCI01	NBCI	-65.59094167		46.14589722		48	114	28	9797	250
-150	400	-50	94	171	-83	1149	116	83	12	341
259	277	320								
NBCI02	NBCI	-65.59094167		46.14589722		48	114	28	9797	250
-150	400	-50	94	171	-83	1149	116	83	12	341
259	277	320								
NBCI03	NBCI	-65.59094167		46.14589722		48	114	28	9797	250
-150	400	-50	94	171	-83	1149	116	83	12	341
259	277	320								
NBCI04	NBCI	-65.59094167		46.14589722		48	114	28	9797	250
-150	400	-50	94	171	-83	1149	116	83	12	341
259	277	320								
NBCI05	NBCI	-65.59094167		46.14589722		48	114	28	9797	250
-150	400	-50	94	171	-83	1149	116	83	12	341
259	277	320								
NBCI06	NBCI	-65.59094167		46.14589722		48	114	28	9797	250
-150	400	-50	94	171	-83	1149	116	83	12	341
259	277	320								
NBCI07	NBCI	-65.59094167		46.14589722		48	114	28	9797	250
-150	400	-50	94	171	-83	1149	116	83	12	341
259	277	320								
NBCI08	NBCI	-65.59094167		46.14589722		48	114	28	9797	250
-150	400	-50	94	171	-83	1149	116	83	12	341
259	277	320								
NBCI09	NBCI	-65.59094167		46.14589722		48	114	28	9797	250
-150	400	-50	94	171	-83	1149	116	83	12	341
259	277	320								
NBCI10	NBCI	-65.59094167		46.14589722		48	114	28	9797	250
-150	400	-50	94	171	-83	1149	116	83	12	341

```
259     277     320
NBCI11  NBCI    -65.59094167    46.14589722     48      114     28      9797    250
-150    400     -50     94      171     -83     1149    116     83      12      341
259     277     320
NBCI12  NBCI    -65.59094167    46.14589722     48      114     28      9797    250
-150    400     -50     94      171     -83     1149    116     83      12      341
259     277     320
NBCI13  NBCI    -65.59094167    46.14589722     48      114     28      9797    250
-150    400     -50     94      171     -83     1149    116     83      12      341
259     277     320
NBCI14  NBCI    -65.59094167    46.14589722     48      114     28      9797    250
-150    400     -50     94      171     -83     1149    116     83      12      341
259     277     320
NBCI15  NBCI    -65.59094167    46.14589722     48      114     28      9797    250
-150    400     -50     94      171     -83     1149    116     83      12      341
259     277     320
NBCI16  NBCI    -65.59094167    46.14589722     48      114     28      9797    250
-150    400     -50     94      171     -83     1149    116     83      12      341
259     277     320
NBCI17  NBCI    -65.59094167    46.14589722     48      114     28      9797    250
-150    400     -50     94      171     -83     1149    116     83      12      341
259     277     320
NBCI18  NBCI    -65.59094167    46.14589722     48      114     28      9797    250
-150    400     -50     94      171     -83     1149    116     83      12      341
259     277     320
NBCI19  NBCI    -65.59094167    46.14589722     48      114     28      9797    250
-150    400     -50     94      171     -83     1149    116     83      12      341
259     277     320
NBCI20  NBCI    -65.59094167    46.14589722     48      114     28      9797    250
-150    400     -50     94      171     -83     1149    116     83      12      341
259     277     320
NBCI21  NBCI    -65.59094167    46.14589722     48      114     28      9797    250
-150    400     -50     94      171     -83     1149    116     83      12      341
259     277     320
NBCI22  NBCI    -65.59094167    46.14589722     48      114     28      9797    250
-150    400     -50     94      171     -83     1149    116     83      12      341
259     277     320
NBCI23  NBCI    -65.59094167    46.14589722     48      114     28      9797    250
-150    400     -50     94      171     -83     1149    116     83      12      341
259     277     320
NBCI24  NBCI    -65.59094167    46.14589722     48      114     28      9797    250
-150    400     -50     94      171     -83     1149    116     83      12      341
259     277     320
NBCI25  NBCI    -65.59094167    46.14589722     48      114     28      9797    250
-150    400     -50     94      171     -83     1149    116     83      12      341
259     277     320
NBCI26  NBCI    -65.59094167    46.14589722     48      114     28      9797    250
-150    400     -50     94      171     -83     1149    116     83      12      341
259     277     320
NBCI27  NBCI    -65.59094167    46.14589722     48      114     28      9797    250
-150    400     -50     94      171     -83     1149    116     83      12      341
259     277     320
NBCI28  NBCI    -65.59094167    46.14589722     48      114     28      9797    250
-150    400     -50     94      171     -83     1149    116     83      12      341
259     277     320
NBCI29  NBCI    -65.59094167    46.14589722     48      114     28      9797    250
-150    400     -50     94      171     -83     1149    116     83      12      341
259     277     320
NBCI30  NBCI    -65.59094167    46.14589722     48      114     28      9797    250
-150    400     -50     94      171     -83     1149    116     83      12      341
259     277     320
NBCI31  NBCI    -65.59094167    46.14589722     48      114     28      9797    250
-150    400     -50     94      171     -83     1149    116     83      12      341
```

259	277	320								
NBCI32	NBCI	-65.59094167	46.14589722	48	114	28	9797	250		
-150	400	-50	94	171	-83	1149	116	83	12	341
259	277	320								
NBCI33	NBCI	-65.59094167	46.14589722	48	114	28	9797	250		
-150	400	-50	94	171	-83	1149	116	83	12	341
259	277	320								
NBCI34	NBCI	-65.59094167	46.14589722	48	114	28	9797	250		
-150	400	-50	94	171	-83	1149	116	83	12	341
259	277	320								
NBCI35	NBCI	-65.59094167	46.14589722	48	114	28	9797	250		
-150	400	-50	94	171	-83	1149	116	83	12	341
259	277	320								
NBCI36	NBCI	-65.59094167	46.14589722	48	114	28	9797	250		
-150	400	-50	94	171	-83	1149	116	83	12	341
259	277	320								
NBCI37	NBCI	-65.59094167	46.14589722	48	114	28	9797	250		
-150	400	-50	94	171	-83	1149	116	83	12	341
259	277	320								
NBCI38	NBCI	-65.59094167	46.14589722	48	114	28	9797	250		
-150	400	-50	94	171	-83	1149	116	83	12	341
259	277	320								
NBCI39	NBCI	-65.59094167	46.14589722	48	114	28	9797	250		
-150	400	-50	94	171	-83	1149	116	83	12	341
259	277	320								
NBCI40	NBCI	-65.59094167	46.14589722	48	114	28	9797	250		
-150	400	-50	94	171	-83	1149	116	83	12	341
259	277	320								
NBCI41	NBCI	-65.59094167	46.14589722	48	114	28	9797	250		
-150	400	-50	94	171	-83	1149	116	83	12	341
259	277	320								
NBCI42	NBCI	-65.59094167	46.14589722	48	114	28	9797	250		
-150	400	-50	94	171	-83	1149	116	83	12	341
259	277	320								
NBCI43	NBCI	-65.59094167	46.14589722	48	114	28	9797	250		
-150	400	-50	94	171	-83	1149	116	83	12	341
259	277	320								
NBCI44	NBCI	-65.59094167	46.14589722	48	114	28	9797	250		
-150	400	-50	94	171	-83	1149	116	83	12	341
259	277	320								
NBCI45	NBCI	-65.59094167	46.14589722	48	114	28	9797	250		
-150	400	-50	94	171	-83	1149	116	83	12	341
259	277	320								
NBCI46	NBCI	-65.59094167	46.14589722	48	114	28	9797	250		
-150	400	-50	94	171	-83	1149	116	83	12	341
259	277	320								
NBCI47	NBCI	-65.59094167	46.14589722	48	114	28	9797	250		
-150	400	-50	94	171	-83	1149	116	83	12	341
259	277	320								
NBCI48	NBCI	-65.59094167	46.14589722	48	114	28	9797	250		
-150	400	-50	94	171	-83	1149	116	83	12	341
259	277	320								
NBCI49	NBCI	-65.59094167	46.14589722	48	114	28	9797	250		
-150	400	-50	94	171	-83	1149	116	83	12	341
259	277	320								
NBCI50	NBCI	-65.59094167	46.14589722	48	114	28	9797	250		
-150	400	-50	94	171	-83	1149	116	83	12	341
259	277	320								
NBCR01	NBCR	-65.929375	46.32062222	44	113	27	10027	247		
-159	406	-58	29	169	-91	1132	115	84	11	329
255	281	305								
NBCR02	NBCR	-65.929375	46.32062222	44	113	27	10027	247		
-159	406	-58	29	169	-91	1132	115	84	11	329

```
255     281     305
NBCR03  NBCR    -65.929375      46.32062222     44      113     27      10027   247
-159    406     -58     29      169     -91     1132    115     84      11      329
255     281     305
NBCR04  NBCR    -65.929375      46.32062222     44      113     27      10027   247
-159    406     -58     29      169     -91     1132    115     84      11      329
255     281     305
NBCR05  NBCR    -65.929375      46.32062222     44      113     27      10027   247
-159    406     -58     29      169     -91     1132    115     84      11      329
255     281     305
NBCR06  NBCR    -65.929375      46.32062222     44      113     27      10027   247
-159    406     -58     29      169     -91     1132    115     84      11      329
255     281     305
NBCR07  NBCR    -65.929375      46.32062222     44      113     27      10027   247
-159    406     -58     29      169     -91     1132    115     84      11      329
255     281     305
NBCR08  NBCR    -65.929375      46.32062222     44      113     27      10027   247
-159    406     -58     29      169     -91     1132    115     84      11      329
255     281     305
NBCR09  NBCR    -65.929375      46.32062222     44      113     27      10027   247
-159    406     -58     29      169     -91     1132    115     84      11      329
255     281     305
NBCR10  NBCR    -65.929375      46.32062222     44      113     27      10027   247
-159    406     -58     29      169     -91     1132    115     84      11      329
255     281     305
NBCR11  NBCR    -65.929375      46.32062222     44      113     27      10027   247
-159    406     -58     29      169     -91     1132    115     84      11      329
255     281     305
NBCR12  NBCR    -65.929375      46.32062222     44      113     27      10027   247
-159    406     -58     29      169     -91     1132    115     84      11      329
255     281     305
NBCR13  NBCR    -65.929375      46.32062222     44      113     27      10027   247
-159    406     -58     29      169     -91     1132    115     84      11      329
255     281     305
NBCR14  NBCR    -65.929375      46.32062222     44      113     27      10027   247
-159    406     -58     29      169     -91     1132    115     84      11      329
255     281     305
NBCR15  NBCR    -65.929375      46.32062222     44      113     27      10027   247
-159    406     -58     29      169     -91     1132    115     84      11      329
255     281     305
NBCR16  NBCR    -65.929375      46.32062222     44      113     27      10027   247
-159    406     -58     29      169     -91     1132    115     84      11      329
255     281     305
NBCR17  NBCR    -65.929375      46.32062222     44      113     27      10027   247
-159    406     -58     29      169     -91     1132    115     84      11      329
255     281     305
NBCR18  NBCR    -65.929375      46.32062222     44      113     27      10027   247
-159    406     -58     29      169     -91     1132    115     84      11      329
255     281     305
NBCR19  NBCR    -65.929375      46.32062222     44      113     27      10027   247
-159    406     -58     29      169     -91     1132    115     84      11      329
255     281     305
NBCR20  NBCR    -65.929375      46.32062222     44      113     27      10027   247
-159    406     -58     29      169     -91     1132    115     84      11      329
255     281     305
NBCR21  NBCR    -65.929375      46.32062222     44      113     27      10027   247
-159    406     -58     29      169     -91     1132    115     84      11      329
255     281     305
NBCR22  NBCR    -65.929375      46.32062222     44      113     27      10027   247
-159    406     -58     29      169     -91     1132    115     84      11      329
255     281     305
NBCR23  NBCR    -65.929375      46.32062222     44      113     27      10027   247
-159    406     -58     29      169     -91     1132    115     84      11      329
```

255	281	305								
NBCR24	NBCR	-65.929375	46.32062222	44	113	27	10027	247		
-159	406	-58	29	169	-91	1132	115	84	11	329
255	281	305								
NBCR25	NBCR	-65.929375	46.32062222	44	113	27	10027	247		
-159	406	-58	29	169	-91	1132	115	84	11	329
255	281	305								
NBCR26	NBCR	-65.929375	46.32062222	44	113	27	10027	247		
-159	406	-58	29	169	-91	1132	115	84	11	329
255	281	305								
NBCR27	NBCR	-65.929375	46.32062222	44	113	27	10027	247		
-159	406	-58	29	169	-91	1132	115	84	11	329
255	281	305								
NBCR28	NBCR	-65.929375	46.32062222	44	113	27	10027	247		
-159	406	-58	29	169	-91	1132	115	84	11	329
255	281	305								
NBCR29	NBCR	-65.929375	46.32062222	44	113	27	10027	247		
-159	406	-58	29	169	-91	1132	115	84	11	329
255	281	305								
NBCR30	NBCR	-65.929375	46.32062222	44	113	27	10027	247		
-159	406	-58	29	169	-91	1132	115	84	11	329
255	281	305								
NBCR31	NBCR	-65.929375	46.32062222	44	113	27	10027	247		
-159	406	-58	29	169	-91	1132	115	84	11	329
255	281	305								
NBCR32	NBCR	-65.929375	46.32062222	44	113	27	10027	247		
-159	406	-58	29	169	-91	1132	115	84	11	329
255	281	305								
NBCR33	NBCR	-65.929375	46.32062222	44	113	27	10027	247		
-159	406	-58	29	169	-91	1132	115	84	11	329
255	281	305								
NBCR34	NBCR	-65.929375	46.32062222	44	113	27	10027	247		
-159	406	-58	29	169	-91	1132	115	84	11	329
255	281	305								
NBCR35	NBCR	-65.929375	46.32062222	44	113	27	10027	247		
-159	406	-58	29	169	-91	1132	115	84	11	329
255	281	305								
NBCR36	NBCR	-65.929375	46.32062222	44	113	27	10027	247		
-159	406	-58	29	169	-91	1132	115	84	11	329
255	281	305								
NBCR37	NBCR	-65.929375	46.32062222	44	113	27	10027	247		
-159	406	-58	29	169	-91	1132	115	84	11	329
255	281	305								
NBCR38	NBCR	-65.929375	46.32062222	44	113	27	10027	247		
-159	406	-58	29	169	-91	1132	115	84	11	329
255	281	305								
NBCR39	NBCR	-65.929375	46.32062222	44	113	27	10027	247		
-159	406	-58	29	169	-91	1132	115	84	11	329
255	281	305								
NBCR40	NBCR	-65.929375	46.32062222	44	113	27	10027	247		
-159	406	-58	29	169	-91	1132	115	84	11	329
255	281	305								
NBCR41	NBCR	-65.929375	46.32062222	44	113	27	10027	247		
-159	406	-58	29	169	-91	1132	115	84	11	329
255	281	305								
NBCR42	NBCR	-65.929375	46.32062222	44	113	27	10027	247		
-159	406	-58	29	169	-91	1132	115	84	11	329
255	281	305								
NBCR43	NBCR	-65.929375	46.32062222	44	113	27	10027	247		
-159	406	-58	29	169	-91	1132	115	84	11	329
255	281	305								
NBCR44	NBCR	-65.929375	46.32062222	44	113	27	10027	247		
-159	406	-58	29	169	-91	1132	115	84	11	329

255	281	305								
NBCR45	NBCR	-65.929375		46.32062222		44	113	27	10027	247
-159	406	-58	29	169	-91	1132	115	84	11	329
255	281	305								
NBCR46	NBCR	-65.929375		46.32062222		44	113	27	10027	247
-159	406	-58	29	169	-91	1132	115	84	11	329
255	281	305								
NBCR47	NBCR	-65.929375		46.32062222		44	113	27	10027	247
-159	406	-58	29	169	-91	1132	115	84	11	329
255	281	305								
NBCR48	NBCR	-65.929375		46.32062222		44	113	27	10027	247
-159	406	-58	29	169	-91	1132	115	84	11	329
255	281	305								
NBCR49	NBCR	-65.929375		46.32062222		44	113	27	10027	247
-159	406	-58	29	169	-91	1132	115	84	11	329
255	281	305								
NBCR50	NBCR	-65.929375		46.32062222		44	113	27	10027	247
-159	406	-58	29	169	-91	1132	115	84	11	329
255	281	305								
NBOP01	NBOP	-66.66477222		45.95601667		54	109	27	9918	252
-145	397	9	-22	178	-80	1082	110	78	11	310
237	265	279								
NBOP02	NBOP	-66.66477222		45.95601667		54	109	27	9918	252
-145	397	9	-22	178	-80	1082	110	78	11	310
237	265	279								
NBOP03	NBOP	-66.66477222		45.95601667		54	109	27	9918	252
-145	397	9	-22	178	-80	1082	110	78	11	310
237	265	279								
NBOP04	NBOP	-66.66477222		45.95601667		54	109	27	9918	252
-145	397	9	-22	178	-80	1082	110	78	11	310
237	265	279								
NBOP05	NBOP	-66.66477222		45.95601667		54	109	27	9918	252
-145	397	9	-22	178	-80	1082	110	78	11	310
237	265	279								
NBOP06	NBOP	-66.66477222		45.95601667		54	109	27	9918	252
-145	397	9	-22	178	-80	1082	110	78	11	310
237	265	279								
NBOP07	NBOP	-66.66477222		45.95601667		54	109	27	9918	252
-145	397	9	-22	178	-80	1082	110	78	11	310
237	265	279								
NBOP08	NBOP	-66.66477222		45.95601667		54	109	27	9918	252
-145	397	9	-22	178	-80	1082	110	78	11	310
237	265	279								
NBOP09	NBOP	-66.66477222		45.95601667		54	109	27	9918	252
-145	397	9	-22	178	-80	1082	110	78	11	310
237	265	279								
NBOP10	NBOP	-66.66477222		45.95601667		54	109	27	9918	252
-145	397	9	-22	178	-80	1082	110	78	11	310
237	265	279								
NBOP11	NBOP	-66.66477222		45.95601667		54	109	27	9918	252
-145	397	9	-22	178	-80	1082	110	78	11	310
237	265	279								
NBOP12	NBOP	-66.66477222		45.95601667		54	109	27	9918	252
-145	397	9	-22	178	-80	1082	110	78	11	310
237	265	279								
NBOP13	NBOP	-66.66477222		45.95601667		54	109	27	9918	252
-145	397	9	-22	178	-80	1082	110	78	11	310
237	265	279								
NBOP14	NBOP	-66.66477222		45.95601667		54	109	27	9918	252
-145	397	9	-22	178	-80	1082	110	78	11	310
237	265	279								
NBOP15	NBOP	-66.66477222		45.95601667		54	109	27	9918	252
-145	397	9	-22	178	-80	1082	110	78	11	310

237	265	279								
NBOP16	NBOP	-66.66477222		45.95601667		54	109	27	9918	252
-145	397	9	-22	178	-80	1082	110	78	11	310
237	265	279								
NBOP17	NBOP	-66.66477222		45.95601667		54	109	27	9918	252
-145	397	9	-22	178	-80	1082	110	78	11	310
237	265	279								
NBOP18	NBOP	-66.66477222		45.95601667		54	109	27	9918	252
-145	397	9	-22	178	-80	1082	110	78	11	310
237	265	279								
NBOP19	NBOP	-66.66477222		45.95601667		54	109	27	9918	252
-145	397	9	-22	178	-80	1082	110	78	11	310
237	265	279								
NBOP20	NBOP	-66.66477222		45.95601667		54	109	27	9918	252
-145	397	9	-22	178	-80	1082	110	78	11	310
237	265	279								
NBOP21	NBOP	-66.66477222		45.95601667		54	109	27	9918	252
-145	397	9	-22	178	-80	1082	110	78	11	310
237	265	279								
NBOP22	NBOP	-66.66477222		45.95601667		54	109	27	9918	252
-145	397	9	-22	178	-80	1082	110	78	11	310
237	265	279								
NBOP23	NBOP	-66.66477222		45.95601667		54	109	27	9918	252
-145	397	9	-22	178	-80	1082	110	78	11	310
237	265	279								
NBOP24	NBOP	-66.66477222		45.95601667		54	109	27	9918	252
-145	397	9	-22	178	-80	1082	110	78	11	310
237	265	279								
NBOP25	NBOP	-66.66477222		45.95601667		54	109	27	9918	252
-145	397	9	-22	178	-80	1082	110	78	11	310
237	265	279								
NBOP26	NBOP	-66.66477222		45.95601667		54	109	27	9918	252
-145	397	9	-22	178	-80	1082	110	78	11	310
237	265	279								
NBOP27	NBOP	-66.66477222		45.95601667		54	109	27	9918	252
-145	397	9	-22	178	-80	1082	110	78	11	310
237	265	279								
NBOP28	NBOP	-66.66477222		45.95601667		54	109	27	9918	252
-145	397	9	-22	178	-80	1082	110	78	11	310
237	265	279								
NBOP29	NBOP	-66.66477222		45.95601667		54	109	27	9918	252
-145	397	9	-22	178	-80	1082	110	78	11	310
237	265	279								
NBOP30	NBOP	-66.66477222		45.95601667		54	109	27	9918	252
-145	397	9	-22	178	-80	1082	110	78	11	310
237	265	279								
NBOP31	NBOP	-66.66477222		45.95601667		54	109	27	9918	252
-145	397	9	-22	178	-80	1082	110	78	11	310
237	265	279								
NBOP32	NBOP	-66.66477222		45.95601667		54	109	27	9918	252
-145	397	9	-22	178	-80	1082	110	78	11	310
237	265	279								
NBOP33	NBOP	-66.66477222		45.95601667		54	109	27	9918	252
-145	397	9	-22	178	-80	1082	110	78	11	310
237	265	279								
NBOP34	NBOP	-66.66477222		45.95601667		54	109	27	9918	252
-145	397	9	-22	178	-80	1082	110	78	11	310
237	265	279								
NBOP35	NBOP	-66.66477222		45.95601667		54	109	27	9918	252
-145	397	9	-22	178	-80	1082	110	78	11	310
237	265	279								
NBOP36	NBOP	-66.66477222		45.95601667		54	109	27	9918	252
-145	397	9	-22	178	-80	1082	110	78	11	310

237	265	279								
NBOP37	NBOP	-66.66477222		45.95601667		54	109	27	9918	252
-145	397	9	-22	178	-80	1082	110	78	11	310
237	265	279								
NBOP38	NBOP	-66.66477222		45.95601667		54	109	27	9918	252
-145	397	9	-22	178	-80	1082	110	78	11	310
237	265	279								
NBOP39	NBOP	-66.66477222		45.95601667		54	109	27	9918	252
-145	397	9	-22	178	-80	1082	110	78	11	310
237	265	279								
NBOP40	NBOP	-66.66477222		45.95601667		54	109	27	9918	252
-145	397	9	-22	178	-80	1082	110	78	11	310
237	265	279								
NBOP41	NBOP	-66.66477222		45.95601667		54	109	27	9918	252
-145	397	9	-22	178	-80	1082	110	78	11	310
237	265	279								
NBOP42	NBOP	-66.66477222		45.95601667		54	109	27	9918	252
-145	397	9	-22	178	-80	1082	110	78	11	310
237	265	279								
NBOP43	NBOP	-66.66477222		45.95601667		54	109	27	9918	252
-145	397	9	-22	178	-80	1082	110	78	11	310
237	265	279								
NBOP44	NBOP	-66.66477222		45.95601667		54	109	27	9918	252
-145	397	9	-22	178	-80	1082	110	78	11	310
237	265	279								
NBOP45	NBOP	-66.66477222		45.95601667		54	109	27	9918	252
-145	397	9	-22	178	-80	1082	110	78	11	310
237	265	279								
NBOP46	NBOP	-66.66477222		45.95601667		54	109	27	9918	252
-145	397	9	-22	178	-80	1082	110	78	11	310
237	265	279								
NBOP47	NBOP	-66.66477222		45.95601667		54	109	27	9918	252
-145	397	9	-22	178	-80	1082	110	78	11	310
237	265	279								
NBOP48	NBOP	-66.66477222		45.95601667		54	109	27	9918	252
-145	397	9	-22	178	-80	1082	110	78	11	310
237	265	279								
NBOP49	NBOP	-66.66477222		45.95601667		54	109	27	9918	252
-145	397	9	-22	178	-80	1082	110	78	11	310
237	265	279								
NBOP50	NBOP	-66.66477222		45.95601667		54	109	27	9918	252
-145	397	9	-22	178	-80	1082	110	78	11	310
237	265	279								
NSSMB01	NSSMB	-63.86819167		44.63948611		62	92	28	8220	227
-99	326	-14	164	165	-44	1399	152	93	17	441
291	294	411								
NSSMB02	NSSMB	-63.86819167		44.63948611		62	92	28	8220	227
-99	326	-14	164	165	-44	1399	152	93	17	441
291	294	411								
NSSMB03	NSSMB	-63.86819167		44.63948611		62	92	28	8220	227
-99	326	-14	164	165	-44	1399	152	93	17	441
291	294	411								
NSSMB04	NSSMB	-63.86819167		44.63948611		62	92	28	8220	227
-99	326	-14	164	165	-44	1399	152	93	17	441
291	294	411								
NSSMB05	NSSMB	-63.86819167		44.63948611		62	92	28	8220	227
-99	326	-14	164	165	-44	1399	152	93	17	441
291	294	411								
NSSMB06	NSSMB	-63.86819167		44.63948611		62	92	28	8220	227
-99	326	-14	164	165	-44	1399	152	93	17	441
291	294	411								
NSSMB07	NSSMB	-63.86819167		44.63948611		62	92	28	8220	227
-99	326	-14	164	165	-44	1399	152	93	17	441

291	294	411								
NSSMB08	NSSMB	-63.86819167		44.63948611		62	92	28	8220	227
-99	326	-14	164	165	-44	1399	152	93	17	441
291	294	411								
NSSMB09	NSSMB	-63.86819167		44.63948611		62	92	28	8220	227
-99	326	-14	164	165	-44	1399	152	93	17	441
291	294	411								
NSSMB10	NSSMB	-63.86819167		44.63948611		62	92	28	8220	227
-99	326	-14	164	165	-44	1399	152	93	17	441
291	294	411								
NSSMB11	NSSMB	-63.86819167		44.63948611		62	92	28	8220	227
-99	326	-14	164	165	-44	1399	152	93	17	441
291	294	411								
NSSMB12	NSSMB	-63.86819167		44.63948611		62	92	28	8220	227
-99	326	-14	164	165	-44	1399	152	93	17	441
291	294	411								
NSSMB13	NSSMB	-63.86819167		44.63948611		62	92	28	8220	227
-99	326	-14	164	165	-44	1399	152	93	17	441
291	294	411								
NSSMB14	NSSMB	-63.86819167		44.63948611		62	92	28	8220	227
-99	326	-14	164	165	-44	1399	152	93	17	441
291	294	411								
NSSMB15	NSSMB	-63.86819167		44.63948611		62	92	28	8220	227
-99	326	-14	164	165	-44	1399	152	93	17	441
291	294	411								
NSSMB16	NSSMB	-63.86819167		44.63948611		62	92	28	8220	227
-99	326	-14	164	165	-44	1399	152	93	17	441
291	294	411								
NSSMB17	NSSMB	-63.86819167		44.63948611		62	92	28	8220	227
-99	326	-14	164	165	-44	1399	152	93	17	441
291	294	411								
NSSMB18	NSSMB	-63.86819167		44.63948611		62	92	28	8220	227
-99	326	-14	164	165	-44	1399	152	93	17	441
291	294	411								
NSSMB19	NSSMB	-63.86819167		44.63948611		62	92	28	8220	227
-99	326	-14	164	165	-44	1399	152	93	17	441
291	294	411								
NSSMB20	NSSMB	-63.86819167		44.63948611		62	92	28	8220	227
-99	326	-14	164	165	-44	1399	152	93	17	441
291	294	411								
NSSMB21	NSSMB	-63.86819167		44.63948611		62	92	28	8220	227
-99	326	-14	164	165	-44	1399	152	93	17	441
291	294	411								
NSSMB22	NSSMB	-63.86819167		44.63948611		62	92	28	8220	227
-99	326	-14	164	165	-44	1399	152	93	17	441
291	294	411								
NSSMB23	NSSMB	-63.86819167		44.63948611		62	92	28	8220	227
-99	326	-14	164	165	-44	1399	152	93	17	441
291	294	411								
NSSMB24	NSSMB	-63.86819167		44.63948611		62	92	28	8220	227
-99	326	-14	164	165	-44	1399	152	93	17	441
291	294	411								
NSSMB25	NSSMB	-63.86819167		44.63948611		62	92	28	8220	227
-99	326	-14	164	165	-44	1399	152	93	17	441
291	294	411								
NSSMB26	NSSMB	-63.86819167		44.63948611		62	92	28	8220	227
-99	326	-14	164	165	-44	1399	152	93	17	441
291	294	411								
NSSMB27	NSSMB	-63.86819167		44.63948611		62	92	28	8220	227
-99	326	-14	164	165	-44	1399	152	93	17	441
291	294	411								
NSSMB28	NSSMB	-63.86819167		44.63948611		62	92	28	8220	227
-99	326	-14	164	165	-44	1399	152	93	17	441

291	294	411								
NSSMB29	NSSMB	-63.86819167		44.63948611		62	92	28	8220	227
-99	326	-14	164	165	-44	1399	152	93	17	441
291	294	411								
NSSMB30	NSSMB	-63.86819167		44.63948611		62	92	28	8220	227
-99	326	-14	164	165	-44	1399	152	93	17	441
291	294	411								
NSSMB31	NSSMB	-63.86819167		44.63948611		62	92	28	8220	227
-99	326	-14	164	165	-44	1399	152	93	17	441
291	294	411								
NSSMB32	NSSMB	-63.86819167		44.63948611		62	92	28	8220	227
-99	326	-14	164	165	-44	1399	152	93	17	441
291	294	411								
NSSMB33	NSSMB	-63.86819167		44.63948611		62	92	28	8220	227
-99	326	-14	164	165	-44	1399	152	93	17	441
291	294	411								
NSSMB34	NSSMB	-63.86819167		44.63948611		62	92	28	8220	227
-99	326	-14	164	165	-44	1399	152	93	17	441
291	294	411								
NSSMB35	NSSMB	-63.86819167		44.63948611		62	92	28	8220	227
-99	326	-14	164	165	-44	1399	152	93	17	441
291	294	411								
NSSMB36	NSSMB	-63.86819167		44.63948611		62	92	28	8220	227
-99	326	-14	164	165	-44	1399	152	93	17	441
291	294	411								
NSSMB37	NSSMB	-63.86819167		44.63948611		62	92	28	8220	227
-99	326	-14	164	165	-44	1399	152	93	17	441
291	294	411								
NSSMB38	NSSMB	-63.86819167		44.63948611		62	92	28	8220	227
-99	326	-14	164	165	-44	1399	152	93	17	441
291	294	411								
NSSMB39	NSSMB	-63.86819167		44.63948611		62	92	28	8220	227
-99	326	-14	164	165	-44	1399	152	93	17	441
291	294	411								
NSSMB40	NSSMB	-63.86819167		44.63948611		62	92	28	8220	227
-99	326	-14	164	165	-44	1399	152	93	17	441
291	294	411								
NSSMB41	NSSMB	-63.86819167		44.63948611		62	92	28	8220	227
-99	326	-14	164	165	-44	1399	152	93	17	441
291	294	411								
NSSMB42	NSSMB	-63.86819167		44.63948611		62	92	28	8220	227
-99	326	-14	164	165	-44	1399	152	93	17	441
291	294	411								
NSSMB43	NSSMB	-63.86819167		44.63948611		62	92	28	8220	227
-99	326	-14	164	165	-44	1399	152	93	17	441
291	294	411								
NSSMB44	NSSMB	-63.86819167		44.63948611		62	92	28	8220	227
-99	326	-14	164	165	-44	1399	152	93	17	441
291	294	411								
NSSMB45	NSSMB	-63.86819167		44.63948611		62	92	28	8220	227
-99	326	-14	164	165	-44	1399	152	93	17	441
291	294	411								
NSSMB46	NSSMB	-63.86819167		44.63948611		62	92	28	8220	227
-99	326	-14	164	165	-44	1399	152	93	17	441
291	294	411								
NSSMB47	NSSMB	-63.86819167		44.63948611		62	92	28	8220	227
-99	326	-14	164	165	-44	1399	152	93	17	441
291	294	411								
NSSMB48	NSSMB	-63.86819167		44.63948611		62	92	28	8220	227
-99	326	-14	164	165	-44	1399	152	93	17	441
291	294	411								
NSSMB49	NSSMB	-63.86819167		44.63948611		62	92	28	8220	227
-99	326	-14	164	165	-44	1399	152	93	17	441

```
291     294     411
NSSMB50 NSSMB   -63.86819167    44.63948611     62      92      28      8220    227
-99     326     -14     164     165     -44     1399    152     93      17      441
291     294     411
NSRL01  NSRL    -65.14228333    44.27316667     62      81      28      7218    206
-82     288     -2      126     150     -32     1339    150     84      19      434
267     276     408
NSRL02  NSRL    -65.14228333    44.27316667     62      81      28      7218    206
-82     288     -2      126     150     -32     1339    150     84      19      434
267     276     408
NSRL03  NSRL    -65.14228333    44.27316667     62      81      28      7218    206
-82     288     -2      126     150     -32     1339    150     84      19      434
267     276     408
NSRL04  NSRL    -65.14228333    44.27316667     62      81      28      7218    206
-82     288     -2      126     150     -32     1339    150     84      19      434
267     276     408
NSRL05  NSRL    -65.14228333    44.27316667     62      81      28      7218    206
-82     288     -2      126     150     -32     1339    150     84      19      434
267     276     408
NSRL06  NSRL    -65.14228333    44.27316667     62      81      28      7218    206
-82     288     -2      126     150     -32     1339    150     84      19      434
267     276     408
NSRL07  NSRL    -65.14228333    44.27316667     62      81      28      7218    206
-82     288     -2      126     150     -32     1339    150     84      19      434
267     276     408
NSRL08  NSRL    -65.14228333    44.27316667     62      81      28      7218    206
-82     288     -2      126     150     -32     1339    150     84      19      434
267     276     408
NSRL09  NSRL    -65.14228333    44.27316667     62      81      28      7218    206
-82     288     -2      126     150     -32     1339    150     84      19      434
267     276     408
NSRL10  NSRL    -65.14228333    44.27316667     62      81      28      7218    206
-82     288     -2      126     150     -32     1339    150     84      19      434
267     276     408
NSRL11  NSRL    -65.14228333    44.27316667     62      81      28      7218    206
-82     288     -2      126     150     -32     1339    150     84      19      434
267     276     408
NSRL12  NSRL    -65.14228333    44.27316667     62      81      28      7218    206
-82     288     -2      126     150     -32     1339    150     84      19      434
267     276     408
NSRL13  NSRL    -65.14228333    44.27316667     62      81      28      7218    206
-82     288     -2      126     150     -32     1339    150     84      19      434
267     276     408
NSRL14  NSRL    -65.14228333    44.27316667     62      81      28      7218    206
-82     288     -2      126     150     -32     1339    150     84      19      434
267     276     408
NSRL15  NSRL    -65.14228333    44.27316667     62      81      28      7218    206
-82     288     -2      126     150     -32     1339    150     84      19      434
267     276     408
NSRL16  NSRL    -65.14228333    44.27316667     62      81      28      7218    206
-82     288     -2      126     150     -32     1339    150     84      19      434
267     276     408
NSRL17  NSRL    -65.14228333    44.27316667     62      81      28      7218    206
-82     288     -2      126     150     -32     1339    150     84      19      434
267     276     408
NSRL18  NSRL    -65.14228333    44.27316667     62      81      28      7218    206
-82     288     -2      126     150     -32     1339    150     84      19      434
267     276     408
NSRL19  NSRL    -65.14228333    44.27316667     62      81      28      7218    206
-82     288     -2      126     150     -32     1339    150     84      19      434
267     276     408
NSRL20  NSRL    -65.14228333    44.27316667     62      81      28      7218    206
-82     288     -2      126     150     -32     1339    150     84      19      434
```

```
267	276	408
NSRL21	NSRL	-65.14228333	44.27316667	62	81	28	7218	206
-82	288	-2	126	150	-32	1339	150	84	19	434
267	276	408
NSRL22	NSRL	-65.14228333	44.27316667	62	81	28	7218	206
-82	288	-2	126	150	-32	1339	150	84	19	434
267	276	408
NSRL23	NSRL	-65.14228333	44.27316667	62	81	28	7218	206
-82	288	-2	126	150	-32	1339	150	84	19	434
267	276	408
NSRL24	NSRL	-65.14228333	44.27316667	62	81	28	7218	206
-82	288	-2	126	150	-32	1339	150	84	19	434
267	276	408
NSRL25	NSRL	-65.14228333	44.27316667	62	81	28	7218	206
-82	288	-2	126	150	-32	1339	150	84	19	434
267	276	408
NSRL26	NSRL	-65.14228333	44.27316667	62	81	28	7218	206
-82	288	-2	126	150	-32	1339	150	84	19	434
267	276	408
NSRL27	NSRL	-65.14228333	44.27316667	62	81	28	7218	206
-82	288	-2	126	150	-32	1339	150	84	19	434
267	276	408
NSRL28	NSRL	-65.14228333	44.27316667	62	81	28	7218	206
-82	288	-2	126	150	-32	1339	150	84	19	434
267	276	408
NSRL29	NSRL	-65.14228333	44.27316667	62	81	28	7218	206
-82	288	-2	126	150	-32	1339	150	84	19	434
267	276	408
NSRL30	NSRL	-65.14228333	44.27316667	62	81	28	7218	206
-82	288	-2	126	150	-32	1339	150	84	19	434
267	276	408
NSRL31	NSRL	-65.14228333	44.27316667	62	81	28	7218	206
-82	288	-2	126	150	-32	1339	150	84	19	434
267	276	408
NSRL32	NSRL	-65.14228333	44.27316667	62	81	28	7218	206
-82	288	-2	126	150	-32	1339	150	84	19	434
267	276	408
NSRL33	NSRL	-65.14228333	44.27316667	62	81	28	7218	206
-82	288	-2	126	150	-32	1339	150	84	19	434
267	276	408
NSRL34	NSRL	-65.14228333	44.27316667	62	81	28	7218	206
-82	288	-2	126	150	-32	1339	150	84	19	434
267	276	408
NSRL35	NSRL	-65.14228333	44.27316667	62	81	28	7218	206
-82	288	-2	126	150	-32	1339	150	84	19	434
267	276	408
NSRL36	NSRL	-65.14228333	44.27316667	62	81	28	7218	206
-82	288	-2	126	150	-32	1339	150	84	19	434
267	276	408
NSRL37	NSRL	-65.14228333	44.27316667	62	81	28	7218	206
-82	288	-2	126	150	-32	1339	150	84	19	434
267	276	408
NSRL38	NSRL	-65.14228333	44.27316667	62	81	28	7218	206
-82	288	-2	126	150	-32	1339	150	84	19	434
267	276	408
NSRL39	NSRL	-65.14228333	44.27316667	62	81	28	7218	206
-82	288	-2	126	150	-32	1339	150	84	19	434
267	276	408
NSRL40	NSRL	-65.14228333	44.27316667	62	81	28	7218	206
-82	288	-2	126	150	-32	1339	150	84	19	434
267	276	408
NSRL41	NSRL	-65.14228333	44.27316667	62	81	28	7218	206
-82	288	-2	126	150	-32	1339	150	84	19	434
```

267	276	408								
NSRL42	NSRL	-65.14228333		44.27316667		62	81	28	7218	206
-82	288	-2	126	150	-32	1339	150	84	19	434
267	276	408								
NSRL43	NSRL	-65.14228333		44.27316667		62	81	28	7218	206
-82	288	-2	126	150	-32	1339	150	84	19	434
267	276	408								
NSRL44	NSRL	-65.14228333		44.27316667		62	81	28	7218	206
-82	288	-2	126	150	-32	1339	150	84	19	434
267	276	408								
NSRL45	NSRL	-65.14228333		44.27316667		62	81	28	7218	206
-82	288	-2	126	150	-32	1339	150	84	19	434
267	276	408								
NSRL46	NSRL	-65.14228333		44.27316667		62	81	28	7218	206
-82	288	-2	126	150	-32	1339	150	84	19	434
267	276	408								
NSRL47	NSRL	-65.14228333		44.27316667		62	81	28	7218	206
-82	288	-2	126	150	-32	1339	150	84	19	434
267	276	408								
NSRL48	NSRL	-65.14228333		44.27316667		62	81	28	7218	206
-82	288	-2	126	150	-32	1339	150	84	19	434
267	276	408								
NSRL49	NSRL	-65.14228333		44.27316667		62	81	28	7218	206
-82	288	-2	126	150	-32	1339	150	84	19	434
267	276	408								
NSRL50	NSRL	-65.14228333		44.27316667		62	81	28	7218	206
-82	288	-2	126	150	-32	1339	150	84	19	434
267	276	408								
NSDL01	NSDL	-64.40985278		44.50441944		64	91	28	7928	223
-93	316	-10	136	162	-39	1344	148	87	18	427
273	275	399								
NSDL02	NSDL	-64.40985278		44.50441944		64	91	28	7928	223
-93	316	-10	136	162	-39	1344	148	87	18	427
273	275	399								
NSDL03	NSDL	-64.40985278		44.50441944		64	91	28	7928	223
-93	316	-10	136	162	-39	1344	148	87	18	427
273	275	399								
NSDL04	NSDL	-64.40985278		44.50441944		64	91	28	7928	223
-93	316	-10	136	162	-39	1344	148	87	18	427
273	275	399								
NSDL05	NSDL	-64.40985278		44.50441944		64	91	28	7928	223
-93	316	-10	136	162	-39	1344	148	87	18	427
273	275	399								
NSDL06	NSDL	-64.40985278		44.50441944		64	91	28	7928	223
-93	316	-10	136	162	-39	1344	148	87	18	427
273	275	399								
NSDL07	NSDL	-64.40985278		44.50441944		64	91	28	7928	223
-93	316	-10	136	162	-39	1344	148	87	18	427
273	275	399								
NSDL08	NSDL	-64.40985278		44.50441944		64	91	28	7928	223
-93	316	-10	136	162	-39	1344	148	87	18	427
273	275	399								
NSDL09	NSDL	-64.40985278		44.50441944		64	91	28	7928	223
-93	316	-10	136	162	-39	1344	148	87	18	427
273	275	399								
NSDL10	NSDL	-64.40985278		44.50441944		64	91	28	7928	223
-93	316	-10	136	162	-39	1344	148	87	18	427
273	275	399								
NSDL11	NSDL	-64.40985278		44.50441944		64	91	28	7928	223
-93	316	-10	136	162	-39	1344	148	87	18	427
273	275	399								
NSDL12	NSDL	-64.40985278		44.50441944		64	91	28	7928	223
-93	316	-10	136	162	-39	1344	148	87	18	427

273	275	399								
NSDL13	NSDL	-64.40985278		44.50441944		64	91	28	7928	223
-93	316	-10	136	162	-39	1344	148	87	18	427
273	275	399								
NSDL14	NSDL	-64.40985278		44.50441944		64	91	28	7928	223
-93	316	-10	136	162	-39	1344	148	87	18	427
273	275	399								
NSDL15	NSDL	-64.40985278		44.50441944		64	91	28	7928	223
-93	316	-10	136	162	-39	1344	148	87	18	427
273	275	399								
NSDL16	NSDL	-64.40985278		44.50441944		64	91	28	7928	223
-93	316	-10	136	162	-39	1344	148	87	18	427
273	275	399								
NSDL17	NSDL	-64.40985278		44.50441944		64	91	28	7928	223
-93	316	-10	136	162	-39	1344	148	87	18	427
273	275	399								
NSDL18	NSDL	-64.40985278		44.50441944		64	91	28	7928	223
-93	316	-10	136	162	-39	1344	148	87	18	427
273	275	399								
NSDL19	NSDL	-64.40985278		44.50441944		64	91	28	7928	223
-93	316	-10	136	162	-39	1344	148	87	18	427
273	275	399								
NSDL20	NSDL	-64.40985278		44.50441944		64	91	28	7928	223
-93	316	-10	136	162	-39	1344	148	87	18	427
273	275	399								
NSDL21	NSDL	-64.40985278		44.50441944		64	91	28	7928	223
-93	316	-10	136	162	-39	1344	148	87	18	427
273	275	399								
NSDL22	NSDL	-64.40985278		44.50441944		64	91	28	7928	223
-93	316	-10	136	162	-39	1344	148	87	18	427
273	275	399								
NSDL23	NSDL	-64.40985278		44.50441944		64	91	28	7928	223
-93	316	-10	136	162	-39	1344	148	87	18	427
273	275	399								
NSDL24	NSDL	-64.40985278		44.50441944		64	91	28	7928	223
-93	316	-10	136	162	-39	1344	148	87	18	427
273	275	399								
NSDL25	NSDL	-64.40985278		44.50441944		64	91	28	7928	223
-93	316	-10	136	162	-39	1344	148	87	18	427
273	275	399								
NSDL26	NSDL	-64.40985278		44.50441944		64	91	28	7928	223
-93	316	-10	136	162	-39	1344	148	87	18	427
273	275	399								
NSDL27	NSDL	-64.40985278		44.50441944		64	91	28	7928	223
-93	316	-10	136	162	-39	1344	148	87	18	427
273	275	399								
NSDL28	NSDL	-64.40985278		44.50441944		64	91	28	7928	223
-93	316	-10	136	162	-39	1344	148	87	18	427
273	275	399								
NSDL29	NSDL	-64.40985278		44.50441944		64	91	28	7928	223
-93	316	-10	136	162	-39	1344	148	87	18	427
273	275	399								
NSDL30	NSDL	-64.40985278		44.50441944		64	91	28	7928	223
-93	316	-10	136	162	-39	1344	148	87	18	427
273	275	399								
NSDL31	NSDL	-64.40985278		44.50441944		64	91	28	7928	223
-93	316	-10	136	162	-39	1344	148	87	18	427
273	275	399								
NSDL32	NSDL	-64.40985278		44.50441944		64	91	28	7928	223
-93	316	-10	136	162	-39	1344	148	87	18	427
273	275	399								
NSDL33	NSDL	-64.40985278		44.50441944		64	91	28	7928	223
-93	316	-10	136	162	-39	1344	148	87	18	427

```
273     275     399
NSDL34  NSDL    -64.40985278    44.50441944     64      91      28      7928    223
-93     316     -10     136     162     -39     1344    148     87      18      427
273     275     399
NSDL35  NSDL    -64.40985278    44.50441944     64      91      28      7928    223
-93     316     -10     136     162     -39     1344    148     87      18      427
273     275     399
NSDL36  NSDL    -64.40985278    44.50441944     64      91      28      7928    223
-93     316     -10     136     162     -39     1344    148     87      18      427
273     275     399
NSDL37  NSDL    -64.40985278    44.50441944     64      91      28      7928    223
-93     316     -10     136     162     -39     1344    148     87      18      427
273     275     399
NSDL38  NSDL    -64.40985278    44.50441944     64      91      28      7928    223
-93     316     -10     136     162     -39     1344    148     87      18      427
273     275     399
NSDL39  NSDL    -64.40985278    44.50441944     64      91      28      7928    223
-93     316     -10     136     162     -39     1344    148     87      18      427
273     275     399
NSDL40  NSDL    -64.40985278    44.50441944     64      91      28      7928    223
-93     316     -10     136     162     -39     1344    148     87      18      427
273     275     399
NSDL41  NSDL    -64.40985278    44.50441944     64      91      28      7928    223
-93     316     -10     136     162     -39     1344    148     87      18      427
273     275     399
NSDL42  NSDL    -64.40985278    44.50441944     64      91      28      7928    223
-93     316     -10     136     162     -39     1344    148     87      18      427
273     275     399
NSDL43  NSDL    -64.40985278    44.50441944     64      91      28      7928    223
-93     316     -10     136     162     -39     1344    148     87      18      427
273     275     399
NSDL44  NSDL    -64.40985278    44.50441944     64      91      28      7928    223
-93     316     -10     136     162     -39     1344    148     87      18      427
273     275     399
NSDL45  NSDL    -64.40985278    44.50441944     64      91      28      7928    223
-93     316     -10     136     162     -39     1344    148     87      18      427
273     275     399
NSDL46  NSDL    -64.40985278    44.50441944     64      91      28      7928    223
-93     316     -10     136     162     -39     1344    148     87      18      427
273     275     399
NSDL47  NSDL    -64.40985278    44.50441944     64      91      28      7928    223
-93     316     -10     136     162     -39     1344    148     87      18      427
273     275     399
NSDL48  NSDL    -64.40985278    44.50441944     64      91      28      7928    223
-93     316     -10     136     162     -39     1344    148     87      18      427
273     275     399
NSDL49  NSDL    -64.40985278    44.50441944     64      91      28      7928    223
-93     316     -10     136     162     -39     1344    148     87      18      427
273     275     399
NSDL50  NSDL    -64.40985278    44.50441944     64      91      28      7928    223
-93     316     -10     136     162     -39     1344    148     87      18      427
273     275     399
NSUM01  NSUM    -63.60874167    44.95438056     61      97      28      8569    237
-107    344     -19     140     169     -50     1394    153     92      17      442
290     293     415
NSUM02  NSUM    -63.60874167    44.95438056     61      97      28      8569    237
-107    344     -19     140     169     -50     1394    153     92      17      442
290     293     415
NSUM03  NSUM    -63.60874167    44.95438056     61      97      28      8569    237
-107    344     -19     140     169     -50     1394    153     92      17      442
290     293     415
NSUM04  NSUM    -63.60874167    44.95438056     61      97      28      8569    237
-107    344     -19     140     169     -50     1394    153     92      17      442
```

290	293	415								
NSUM05	NSUM	-63.60874167		44.95438056		61	97	28	8569	237
-107	344	-19	140	169	-50	1394	153	92	17	442
290	293	415								
NSUM06	NSUM	-63.60874167		44.95438056		61	97	28	8569	237
-107	344	-19	140	169	-50	1394	153	92	17	442
290	293	415								
NSUM07	NSUM	-63.60874167		44.95438056		61	97	28	8569	237
-107	344	-19	140	169	-50	1394	153	92	17	442
290	293	415								
NSUM08	NSUM	-63.60874167		44.95438056		61	97	28	8569	237
-107	344	-19	140	169	-50	1394	153	92	17	442
290	293	415								
NSUM09	NSUM	-63.60874167		44.95438056		61	97	28	8569	237
-107	344	-19	140	169	-50	1394	153	92	17	442
290	293	415								
NSUM10	NSUM	-63.60874167		44.95438056		61	97	28	8569	237
-107	344	-19	140	169	-50	1394	153	92	17	442
290	293	415								
NSUM11	NSUM	-63.60874167		44.95438056		61	97	28	8569	237
-107	344	-19	140	169	-50	1394	153	92	17	442
290	293	415								
NSUM12	NSUM	-63.60874167		44.95438056		61	97	28	8569	237
-107	344	-19	140	169	-50	1394	153	92	17	442
290	293	415								
NSUM13	NSUM	-63.60874167		44.95438056		61	97	28	8569	237
-107	344	-19	140	169	-50	1394	153	92	17	442
290	293	415								
NSUM14	NSUM	-63.60874167		44.95438056		61	97	28	8569	237
-107	344	-19	140	169	-50	1394	153	92	17	442
290	293	415								
NSUM15	NSUM	-63.60874167		44.95438056		61	97	28	8569	237
-107	344	-19	140	169	-50	1394	153	92	17	442
290	293	415								
NSUM16	NSUM	-63.60874167		44.95438056		61	97	28	8569	237
-107	344	-19	140	169	-50	1394	153	92	17	442
290	293	415								
NSUM17	NSUM	-63.60874167		44.95438056		61	97	28	8569	237
-107	344	-19	140	169	-50	1394	153	92	17	442
290	293	415								
NSUM18	NSUM	-63.60874167		44.95438056		61	97	28	8569	237
-107	344	-19	140	169	-50	1394	153	92	17	442
290	293	415								
NSUM19	NSUM	-63.60874167		44.95438056		61	97	28	8569	237
-107	344	-19	140	169	-50	1394	153	92	17	442
290	293	415								
NSUM20	NSUM	-63.60874167		44.95438056		61	97	28	8569	237
-107	344	-19	140	169	-50	1394	153	92	17	442
290	293	415								
NSUM21	NSUM	-63.60874167		44.95438056		61	97	28	8569	237
-107	344	-19	140	169	-50	1394	153	92	17	442
290	293	415								
NSUM22	NSUM	-63.60874167		44.95438056		61	97	28	8569	237
-107	344	-19	140	169	-50	1394	153	92	17	442
290	293	415								
NSUM23	NSUM	-63.60874167		44.95438056		61	97	28	8569	237
-107	344	-19	140	169	-50	1394	153	92	17	442
290	293	415								
NSUM24	NSUM	-63.60874167		44.95438056		61	97	28	8569	237
-107	344	-19	140	169	-50	1394	153	92	17	442
290	293	415								
NSUM25	NSUM	-63.60874167		44.95438056		61	97	28	8569	237
-107	344	-19	140	169	-50	1394	153	92	17	442

290	293	415								
NSUM26	NSUM	-63.60874167		44.95438056		61	97	28	8569	237
-107	344	-19	140	169	-50	1394	153	92	17	442
290	293	415								
NSUM27	NSUM	-63.60874167		44.95438056		61	97	28	8569	237
-107	344	-19	140	169	-50	1394	153	92	17	442
290	293	415								
NSUM28	NSUM	-63.60874167		44.95438056		61	97	28	8569	237
-107	344	-19	140	169	-50	1394	153	92	17	442
290	293	415								
NSUM29	NSUM	-63.60874167		44.95438056		61	97	28	8569	237
-107	344	-19	140	169	-50	1394	153	92	17	442
290	293	415								
NSUM30	NSUM	-63.60874167		44.95438056		61	97	28	8569	237
-107	344	-19	140	169	-50	1394	153	92	17	442
290	293	415								
NSUM31	NSUM	-63.60874167		44.95438056		61	97	28	8569	237
-107	344	-19	140	169	-50	1394	153	92	17	442
290	293	415								
NSUM32	NSUM	-63.60874167		44.95438056		61	97	28	8569	237
-107	344	-19	140	169	-50	1394	153	92	17	442
290	293	415								
NSUM33	NSUM	-63.60874167		44.95438056		61	97	28	8569	237
-107	344	-19	140	169	-50	1394	153	92	17	442
290	293	415								
NSUM34	NSUM	-63.60874167		44.95438056		61	97	28	8569	237
-107	344	-19	140	169	-50	1394	153	92	17	442
290	293	415								
NSUM35	NSUM	-63.60874167		44.95438056		61	97	28	8569	237
-107	344	-19	140	169	-50	1394	153	92	17	442
290	293	415								
NSUM36	NSUM	-63.60874167		44.95438056		61	97	28	8569	237
-107	344	-19	140	169	-50	1394	153	92	17	442
290	293	415								
NSUM37	NSUM	-63.60874167		44.95438056		61	97	28	8569	237
-107	344	-19	140	169	-50	1394	153	92	17	442
290	293	415								
NSUM38	NSUM	-63.60874167		44.95438056		61	97	28	8569	237
-107	344	-19	140	169	-50	1394	153	92	17	442
290	293	415								
NSUM39	NSUM	-63.60874167		44.95438056		61	97	28	8569	237
-107	344	-19	140	169	-50	1394	153	92	17	442
290	293	415								
NSUM40	NSUM	-63.60874167		44.95438056		61	97	28	8569	237
-107	344	-19	140	169	-50	1394	153	92	17	442
290	293	415								
NSUM41	NSUM	-63.60874167		44.95438056		61	97	28	8569	237
-107	344	-19	140	169	-50	1394	153	92	17	442
290	293	415								
NSUM42	NSUM	-63.60874167		44.95438056		61	97	28	8569	237
-107	344	-19	140	169	-50	1394	153	92	17	442
290	293	415								
NSUM43	NSUM	-63.60874167		44.95438056		61	97	28	8569	237
-107	344	-19	140	169	-50	1394	153	92	17	442
290	293	415								
NSUM44	NSUM	-63.60874167		44.95438056		61	97	28	8569	237
-107	344	-19	140	169	-50	1394	153	92	17	442
290	293	415								
NSUM45	NSUM	-63.60874167		44.95438056		61	97	28	8569	237
-107	344	-19	140	169	-50	1394	153	92	17	442
290	293	415								
NSUM46	NSUM	-63.60874167		44.95438056		61	97	28	8569	237
-107	344	-19	140	169	-50	1394	153	92	17	442

290	293	415								
NSUM47	NSUM	-63.60874167		44.95438056		61	97	28	8569	237
-107	344	-19	140	169	-50	1394	153	92	17	442
290	293	415								
NSUM48	NSUM	-63.60874167		44.95438056		61	97	28	8569	237
-107	344	-19	140	169	-50	1394	153	92	17	442
290	293	415								
NSUM49	NSUM	-63.60874167		44.95438056		61	97	28	8569	237
-107	344	-19	140	169	-50	1394	153	92	17	442
290	293	415								
NSUM50	NSUM	-63.60874167		44.95438056		61	97	28	8569	237
-107	344	-19	140	169	-50	1394	153	92	17	442
290	293	415								
PQCT01	PQCT	-70.80452222		47.07675556		46	98	23	10696	251
-160	411	178	-35	178	-99	1036	111	65	18	320
200	320	239								
PQCT02	PQCT	-70.80452222		47.07675556		46	98	23	10696	251
-160	411	178	-35	178	-99	1036	111	65	18	320
200	320	239								
PQCT03	PQCT	-70.80452222		47.07675556		46	98	23	10696	251
-160	411	178	-35	178	-99	1036	111	65	18	320
200	320	239								
PQCT04	PQCT	-70.80452222		47.07675556		46	98	23	10696	251
-160	411	178	-35	178	-99	1036	111	65	18	320
200	320	239								
PQCT05	PQCT	-70.80452222		47.07675556		46	98	23	10696	251
-160	411	178	-35	178	-99	1036	111	65	18	320
200	320	239								
PQCT06	PQCT	-70.80452222		47.07675556		46	98	23	10696	251
-160	411	178	-35	178	-99	1036	111	65	18	320
200	320	239								
PQCT07	PQCT	-70.80452222		47.07675556		46	98	23	10696	251
-160	411	178	-35	178	-99	1036	111	65	18	320
200	320	239								
PQCT08	PQCT	-70.80452222		47.07675556		46	98	23	10696	251
-160	411	178	-35	178	-99	1036	111	65	18	320
200	320	239								
PQCT09	PQCT	-70.80452222		47.07675556		46	98	23	10696	251
-160	411	178	-35	178	-99	1036	111	65	18	320
200	320	239								
PQCT10	PQCT	-70.80452222		47.07675556		46	98	23	10696	251
-160	411	178	-35	178	-99	1036	111	65	18	320
200	320	239								
PQCT11	PQCT	-70.80452222		47.07675556		46	98	23	10696	251
-160	411	178	-35	178	-99	1036	111	65	18	320
200	320	239								
PQCT12	PQCT	-70.80452222		47.07675556		46	98	23	10696	251
-160	411	178	-35	178	-99	1036	111	65	18	320
200	320	239								
PQCT13	PQCT	-70.80452222		47.07675556		46	98	23	10696	251
-160	411	178	-35	178	-99	1036	111	65	18	320
200	320	239								
PQCT14	PQCT	-70.80452222		47.07675556		46	98	23	10696	251
-160	411	178	-35	178	-99	1036	111	65	18	320
200	320	239								
PQCT15	PQCT	-70.80452222		47.07675556		46	98	23	10696	251
-160	411	178	-35	178	-99	1036	111	65	18	320
200	320	239								
PQCT16	PQCT	-70.80452222		47.07675556		46	98	23	10696	251
-160	411	178	-35	178	-99	1036	111	65	18	320
200	320	239								
PQCT17	PQCT	-70.80452222		47.07675556		46	98	23	10696	251
-160	411	178	-35	178	-99	1036	111	65	18	320

200 320 239
PQCT18 PQCT -70.80452222 47.07675556 46 98 23 10696 251
-160 411 178 -35 178 -99 1036 111 65 18 320
200 320 239
PQCT19 PQCT -70.80452222 47.07675556 46 98 23 10696 251
-160 411 178 -35 178 -99 1036 111 65 18 320
200 320 239
PQCT20 PQCT -70.80452222 47.07675556 46 98 23 10696 251
-160 411 178 -35 178 -99 1036 111 65 18 320
200 320 239
PQCT21 PQCT -70.80452222 47.07675556 46 98 23 10696 251
-160 411 178 -35 178 -99 1036 111 65 18 320
200 320 239
PQCT22 PQCT -70.80452222 47.07675556 46 98 23 10696 251
-160 411 178 -35 178 -99 1036 111 65 18 320
200 320 239
PQCT23 PQCT -70.80452222 47.07675556 46 98 23 10696 251
-160 411 178 -35 178 -99 1036 111 65 18 320
200 320 239
PQCT24 PQCT -70.80452222 47.07675556 46 98 23 10696 251
-160 411 178 -35 178 -99 1036 111 65 18 320
200 320 239
PQCT25 PQCT -70.80452222 47.07675556 46 98 23 10696 251
-160 411 178 -35 178 -99 1036 111 65 18 320
200 320 239
PQCT26 PQCT -70.80452222 47.07675556 46 98 23 10696 251
-160 411 178 -35 178 -99 1036 111 65 18 320
200 320 239
PQCT27 PQCT -70.80452222 47.07675556 46 98 23 10696 251
-160 411 178 -35 178 -99 1036 111 65 18 320
200 320 239
PQCT28 PQCT -70.80452222 47.07675556 46 98 23 10696 251
-160 411 178 -35 178 -99 1036 111 65 18 320
200 320 239
PQCT29 PQCT -70.80452222 47.07675556 46 98 23 10696 251
-160 411 178 -35 178 -99 1036 111 65 18 320
200 320 239
PQCT30 PQCT -70.80452222 47.07675556 46 98 23 10696 251
-160 411 178 -35 178 -99 1036 111 65 18 320
200 320 239
PQCT31 PQCT -70.80452222 47.07675556 46 98 23 10696 251
-160 411 178 -35 178 -99 1036 111 65 18 320
200 320 239
PQCT32 PQCT -70.80452222 47.07675556 46 98 23 10696 251
-160 411 178 -35 178 -99 1036 111 65 18 320
200 320 239
PQCT33 PQCT -70.80452222 47.07675556 46 98 23 10696 251
-160 411 178 -35 178 -99 1036 111 65 18 320
200 320 239
PQCT34 PQCT -70.80452222 47.07675556 46 98 23 10696 251
-160 411 178 -35 178 -99 1036 111 65 18 320
200 320 239
PQCT35 PQCT -70.80452222 47.07675556 46 98 23 10696 251
-160 411 178 -35 178 -99 1036 111 65 18 320
200 320 239
PQCT36 PQCT -70.80452222 47.07675556 46 98 23 10696 251
-160 411 178 -35 178 -99 1036 111 65 18 320
200 320 239
PQCT37 PQCT -70.80452222 47.07675556 46 98 23 10696 251
-160 411 178 -35 178 -99 1036 111 65 18 320
200 320 239
PQCT38 PQCT -70.80452222 47.07675556 46 98 23 10696 251
-160 411 178 -35 178 -99 1036 111 65 18 320

```
200	320	239
PQCT39	PQCT	-70.80452222	47.07675556	46	98	23	10696	251
-160	411	178	-35	178	-99	1036	111	65	18	320
200	320	239
PQCT40	PQCT	-70.80452222	47.07675556	46	98	23	10696	251
-160	411	178	-35	178	-99	1036	111	65	18	320
200	320	239
PQCT41	PQCT	-70.80452222	47.07675556	46	98	23	10696	251
-160	411	178	-35	178	-99	1036	111	65	18	320
200	320	239
PQCT42	PQCT	-70.80452222	47.07675556	46	98	23	10696	251
-160	411	178	-35	178	-99	1036	111	65	18	320
200	320	239
PQCT43	PQCT	-70.80452222	47.07675556	46	98	23	10696	251
-160	411	178	-35	178	-99	1036	111	65	18	320
200	320	239
PQCT44	PQCT	-70.80452222	47.07675556	46	98	23	10696	251
-160	411	178	-35	178	-99	1036	111	65	18	320
200	320	239
PQCT45	PQCT	-70.80452222	47.07675556	46	98	23	10696	251
-160	411	178	-35	178	-99	1036	111	65	18	320
200	320	239
PQCT46	PQCT	-70.80452222	47.07675556	46	98	23	10696	251
-160	411	178	-35	178	-99	1036	111	65	18	320
200	320	239
PQCT47	PQCT	-70.80452222	47.07675556	46	98	23	10696	251
-160	411	178	-35	178	-99	1036	111	65	18	320
200	320	239
PQCT48	PQCT	-70.80452222	47.07675556	46	98	23	10696	251
-160	411	178	-35	178	-99	1036	111	65	18	320
200	320	239
PQCT49	PQCT	-70.80452222	47.07675556	46	98	23	10696	251
-160	411	178	-35	178	-99	1036	111	65	18	320
200	320	239
PQCT50	PQCT	-70.80452222	47.07675556	46	98	23	10696	251
-160	411	178	-35	178	-99	1036	111	65	18	320
200	320	239
PQSR01	PQSR	-71.01358333	46.01428889	37	103	25	10458	237
-169	406	166	-43	166	-104	1009	116	59	20	325
189	325	212
PQSR02	PQSR	-71.01358333	46.01428889	37	103	25	10458	237
-169	406	166	-43	166	-104	1009	116	59	20	325
189	325	212
PQSR03	PQSR	-71.01358333	46.01428889	37	103	25	10458	237
-169	406	166	-43	166	-104	1009	116	59	20	325
189	325	212
PQSR04	PQSR	-71.01358333	46.01428889	37	103	25	10458	237
-169	406	166	-43	166	-104	1009	116	59	20	325
189	325	212
PQSR05	PQSR	-71.01358333	46.01428889	37	103	25	10458	237
-169	406	166	-43	166	-104	1009	116	59	20	325
189	325	212
PQSR06	PQSR	-71.01358333	46.01428889	37	103	25	10458	237
-169	406	166	-43	166	-104	1009	116	59	20	325
189	325	212
PQSR07	PQSR	-71.01358333	46.01428889	37	103	25	10458	237
-169	406	166	-43	166	-104	1009	116	59	20	325
189	325	212
PQSR08	PQSR	-71.01358333	46.01428889	37	103	25	10458	237
-169	406	166	-43	166	-104	1009	116	59	20	325
189	325	212
PQSR09	PQSR	-71.01358333	46.01428889	37	103	25	10458	237
-169	406	166	-43	166	-104	1009	116	59	20	325
```

189 325 212
PQSR10 PQSR -71.01358333 46.01428889 37 103 25 10458 237
-169 406 166 -43 166 -104 1009 116 59 20 325
189 325 212
PQSR11 PQSR -71.01358333 46.01428889 37 103 25 10458 237
-169 406 166 -43 166 -104 1009 116 59 20 325
189 325 212
PQSR12 PQSR -71.01358333 46.01428889 37 103 25 10458 237
-169 406 166 -43 166 -104 1009 116 59 20 325
189 325 212
PQSR13 PQSR -71.01358333 46.01428889 37 103 25 10458 237
-169 406 166 -43 166 -104 1009 116 59 20 325
189 325 212
PQSR14 PQSR -71.01358333 46.01428889 37 103 25 10458 237
-169 406 166 -43 166 -104 1009 116 59 20 325
189 325 212
PQSR15 PQSR -71.01358333 46.01428889 37 103 25 10458 237
-169 406 166 -43 166 -104 1009 116 59 20 325
189 325 212
PQSR16 PQSR -71.01358333 46.01428889 37 103 25 10458 237
-169 406 166 -43 166 -104 1009 116 59 20 325
189 325 212
PQSR17 PQSR -71.01358333 46.01428889 37 103 25 10458 237
-169 406 166 -43 166 -104 1009 116 59 20 325
189 325 212
PQSR18 PQSR -71.01358333 46.01428889 37 103 25 10458 237
-169 406 166 -43 166 -104 1009 116 59 20 325
189 325 212
PQSR19 PQSR -71.01358333 46.01428889 37 103 25 10458 237
-169 406 166 -43 166 -104 1009 116 59 20 325
189 325 212
PQSR20 PQSR -71.01358333 46.01428889 37 103 25 10458 237
-169 406 166 -43 166 -104 1009 116 59 20 325
189 325 212
PQSR21 PQSR -71.01358333 46.01428889 37 103 25 10458 237
-169 406 166 -43 166 -104 1009 116 59 20 325
189 325 212
PQSR22 PQSR -71.01358333 46.01428889 37 103 25 10458 237
-169 406 166 -43 166 -104 1009 116 59 20 325
189 325 212
PQSR23 PQSR -71.01358333 46.01428889 37 103 25 10458 237
-169 406 166 -43 166 -104 1009 116 59 20 325
189 325 212
PQSR24 PQSR -71.01358333 46.01428889 37 103 25 10458 237
-169 406 166 -43 166 -104 1009 116 59 20 325
189 325 212
PQSR25 PQSR -71.01358333 46.01428889 37 103 25 10458 237
-169 406 166 -43 166 -104 1009 116 59 20 325
189 325 212
PQSR26 PQSR -71.01358333 46.01428889 37 103 25 10458 237
-169 406 166 -43 166 -104 1009 116 59 20 325
189 325 212
PQSR27 PQSR -71.01358333 46.01428889 37 103 25 10458 237
-169 406 166 -43 166 -104 1009 116 59 20 325
189 325 212
PQSR28 PQSR -71.01358333 46.01428889 37 103 25 10458 237
-169 406 166 -43 166 -104 1009 116 59 20 325
189 325 212
PQSR29 PQSR -71.01358333 46.01428889 37 103 25 10458 237
-169 406 166 -43 166 -104 1009 116 59 20 325
189 325 212
PQSR30 PQSR -71.01358333 46.01428889 37 103 25 10458 237
-169 406 166 -43 166 -104 1009 116 59 20 325

189	325	212								
PQSR31	PQSR	-71.01358333		46.01428889		37	103	25	10458	237
-169	406	166	-43	166	-104	1009	116	59	20	325
189	325	212								
PQSR32	PQSR	-71.01358333		46.01428889		37	103	25	10458	237
-169	406	166	-43	166	-104	1009	116	59	20	325
189	325	212								
PQSR33	PQSR	-71.01358333		46.01428889		37	103	25	10458	237
-169	406	166	-43	166	-104	1009	116	59	20	325
189	325	212								
PQSR34	PQSR	-71.01358333		46.01428889		37	103	25	10458	237
-169	406	166	-43	166	-104	1009	116	59	20	325
189	325	212								
PQSR35	PQSR	-71.01358333		46.01428889		37	103	25	10458	237
-169	406	166	-43	166	-104	1009	116	59	20	325
189	325	212								
PQSR36	PQSR	-71.01358333		46.01428889		37	103	25	10458	237
-169	406	166	-43	166	-104	1009	116	59	20	325
189	325	212								
PQSR37	PQSR	-71.01358333		46.01428889		37	103	25	10458	237
-169	406	166	-43	166	-104	1009	116	59	20	325
189	325	212								
PQSR38	PQSR	-71.01358333		46.01428889		37	103	25	10458	237
-169	406	166	-43	166	-104	1009	116	59	20	325
189	325	212								
PQSR39	PQSR	-71.01358333		46.01428889		37	103	25	10458	237
-169	406	166	-43	166	-104	1009	116	59	20	325
189	325	212								
PQSR40	PQSR	-71.01358333		46.01428889		37	103	25	10458	237
-169	406	166	-43	166	-104	1009	116	59	20	325
189	325	212								
PQSR41	PQSR	-71.01358333		46.01428889		37	103	25	10458	237
-169	406	166	-43	166	-104	1009	116	59	20	325
189	325	212								
PQSR42	PQSR	-71.01358333		46.01428889		37	103	25	10458	237
-169	406	166	-43	166	-104	1009	116	59	20	325
189	325	212								
PQSR43	PQSR	-71.01358333		46.01428889		37	103	25	10458	237
-169	406	166	-43	166	-104	1009	116	59	20	325
189	325	212								
PQSR44	PQSR	-71.01358333		46.01428889		37	103	25	10458	237
-169	406	166	-43	166	-104	1009	116	59	20	325
189	325	212								
PQSR45	PQSR	-71.01358333		46.01428889		37	103	25	10458	237
-169	406	166	-43	166	-104	1009	116	59	20	325
189	325	212								
PQSR46	PQSR	-71.01358333		46.01428889		37	103	25	10458	237
-169	406	166	-43	166	-104	1009	116	59	20	325
189	325	212								
PQSR47	PQSR	-71.01358333		46.01428889		37	103	25	10458	237
-169	406	166	-43	166	-104	1009	116	59	20	325
189	325	212								
PQSR48	PQSR	-71.01358333		46.01428889		37	103	25	10458	237
-169	406	166	-43	166	-104	1009	116	59	20	325
189	325	212								
PQSR49	PQSR	-71.01358333		46.01428889		37	103	25	10458	237
-169	406	166	-43	166	-104	1009	116	59	20	325
189	325	212								
PQSR50	PQSR	-71.01358333		46.01428889		37	103	25	10458	237
-169	406	166	-43	166	-104	1009	116	59	20	325
189	325	212								
PQSS01	PQSS	-72.28671667		46.64313333		45	105	24	10975	253
-175	428	167	-35	179	-105	1050	107	65	16	312

```
206	311	238
PQSS02	PQSS	-72.28671667	46.64313333	45	105	24	10975	253
-175	428	167	-35	179	-105	1050	107	65	16	312
206	311	238
PQSS03	PQSS	-72.28671667	46.64313333	45	105	24	10975	253
-175	428	167	-35	179	-105	1050	107	65	16	312
206	311	238
PQSS04	PQSS	-72.28671667	46.64313333	45	105	24	10975	253
-175	428	167	-35	179	-105	1050	107	65	16	312
206	311	238
PQSS05	PQSS	-72.28671667	46.64313333	45	105	24	10975	253
-175	428	167	-35	179	-105	1050	107	65	16	312
206	311	238
PQSS06	PQSS	-72.28671667	46.64313333	45	105	24	10975	253
-175	428	167	-35	179	-105	1050	107	65	16	312
206	311	238
PQSS07	PQSS	-72.28671667	46.64313333	45	105	24	10975	253
-175	428	167	-35	179	-105	1050	107	65	16	312
206	311	238
PQSS08	PQSS	-72.28671667	46.64313333	45	105	24	10975	253
-175	428	167	-35	179	-105	1050	107	65	16	312
206	311	238
PQSS09	PQSS	-72.28671667	46.64313333	45	105	24	10975	253
-175	428	167	-35	179	-105	1050	107	65	16	312
206	311	238
PQSS10	PQSS	-72.28671667	46.64313333	45	105	24	10975	253
-175	428	167	-35	179	-105	1050	107	65	16	312
206	311	238
PQSS11	PQSS	-72.28671667	46.64313333	45	105	24	10975	253
-175	428	167	-35	179	-105	1050	107	65	16	312
206	311	238
PQSS12	PQSS	-72.28671667	46.64313333	45	105	24	10975	253
-175	428	167	-35	179	-105	1050	107	65	16	312
206	311	238
PQSS13	PQSS	-72.28671667	46.64313333	45	105	24	10975	253
-175	428	167	-35	179	-105	1050	107	65	16	312
206	311	238
PQSS14	PQSS	-72.28671667	46.64313333	45	105	24	10975	253
-175	428	167	-35	179	-105	1050	107	65	16	312
206	311	238
PQSS15	PQSS	-72.28671667	46.64313333	45	105	24	10975	253
-175	428	167	-35	179	-105	1050	107	65	16	312
206	311	238
PQSS16	PQSS	-72.28671667	46.64313333	45	105	24	10975	253
-175	428	167	-35	179	-105	1050	107	65	16	312
206	311	238
PQSS17	PQSS	-72.28671667	46.64313333	45	105	24	10975	253
-175	428	167	-35	179	-105	1050	107	65	16	312
206	311	238
PQSS18	PQSS	-72.28671667	46.64313333	45	105	24	10975	253
-175	428	167	-35	179	-105	1050	107	65	16	312
206	311	238
PQSS19	PQSS	-72.28671667	46.64313333	45	105	24	10975	253
-175	428	167	-35	179	-105	1050	107	65	16	312
206	311	238
PQSS20	PQSS	-72.28671667	46.64313333	45	105	24	10975	253
-175	428	167	-35	179	-105	1050	107	65	16	312
206	311	238
PQSS21	PQSS	-72.28671667	46.64313333	45	105	24	10975	253
-175	428	167	-35	179	-105	1050	107	65	16	312
206	311	238
PQSS22	PQSS	-72.28671667	46.64313333	45	105	24	10975	253
-175	428	167	-35	179	-105	1050	107	65	16	312
```

206	311	238								
PQSS23	PQSS	-72.28671667	46.64313333	45	105	24	10975	253		
-175	428	167	-35	179	-105	1050	107	65	16	312
206	311	238								
PQSS24	PQSS	-72.28671667	46.64313333	45	105	24	10975	253		
-175	428	167	-35	179	-105	1050	107	65	16	312
206	311	238								
PQSS25	PQSS	-72.28671667	46.64313333	45	105	24	10975	253		
-175	428	167	-35	179	-105	1050	107	65	16	312
206	311	238								
PQSS26	PQSS	-72.28671667	46.64313333	45	105	24	10975	253		
-175	428	167	-35	179	-105	1050	107	65	16	312
206	311	238								
PQSS27	PQSS	-72.28671667	46.64313333	45	105	24	10975	253		
-175	428	167	-35	179	-105	1050	107	65	16	312
206	311	238								
PQSS28	PQSS	-72.28671667	46.64313333	45	105	24	10975	253		
-175	428	167	-35	179	-105	1050	107	65	16	312
206	311	238								
PQSS29	PQSS	-72.28671667	46.64313333	45	105	24	10975	253		
-175	428	167	-35	179	-105	1050	107	65	16	312
206	311	238								
PQSS30	PQSS	-72.28671667	46.64313333	45	105	24	10975	253		
-175	428	167	-35	179	-105	1050	107	65	16	312
206	311	238								
PQSS31	PQSS	-72.28671667	46.64313333	45	105	24	10975	253		
-175	428	167	-35	179	-105	1050	107	65	16	312
206	311	238								
PQSS32	PQSS	-72.28671667	46.64313333	45	105	24	10975	253		
-175	428	167	-35	179	-105	1050	107	65	16	312
206	311	238								
PQSS33	PQSS	-72.28671667	46.64313333	45	105	24	10975	253		
-175	428	167	-35	179	-105	1050	107	65	16	312
206	311	238								
PQSS34	PQSS	-72.28671667	46.64313333	45	105	24	10975	253		
-175	428	167	-35	179	-105	1050	107	65	16	312
206	311	238								
PQSS35	PQSS	-72.28671667	46.64313333	45	105	24	10975	253		
-175	428	167	-35	179	-105	1050	107	65	16	312
206	311	238								
PQSS36	PQSS	-72.28671667	46.64313333	45	105	24	10975	253		
-175	428	167	-35	179	-105	1050	107	65	16	312
206	311	238								
PQSS37	PQSS	-72.28671667	46.64313333	45	105	24	10975	253		
-175	428	167	-35	179	-105	1050	107	65	16	312
206	311	238								
PQSS38	PQSS	-72.28671667	46.64313333	45	105	24	10975	253		
-175	428	167	-35	179	-105	1050	107	65	16	312
206	311	238								
PQSS39	PQSS	-72.28671667	46.64313333	45	105	24	10975	253		
-175	428	167	-35	179	-105	1050	107	65	16	312
206	311	238								
PQSS40	PQSS	-72.28671667	46.64313333	45	105	24	10975	253		
-175	428	167	-35	179	-105	1050	107	65	16	312
206	311	238								
PQSS41	PQSS	-72.28671667	46.64313333	45	105	24	10975	253		
-175	428	167	-35	179	-105	1050	107	65	16	312
206	311	238								
PQSS42	PQSS	-72.28671667	46.64313333	45	105	24	10975	253		
-175	428	167	-35	179	-105	1050	107	65	16	312
206	311	238								
PQSS43	PQSS	-72.28671667	46.64313333	45	105	24	10975	253		
-175	428	167	-35	179	-105	1050	107	65	16	312

```
206     311     238
PQSS44  PQSS    -72.28671667    46.64313333     45      105     24      10975   253
-175    428     167     -35     179     -105    1050    107     65      16      312
206     311     238
PQSS45  PQSS    -72.28671667    46.64313333     45      105     24      10975   253
-175    428     167     -35     179     -105    1050    107     65      16      312
206     311     238
PQSS46  PQSS    -72.28671667    46.64313333     45      105     24      10975   253
-175    428     167     -35     179     -105    1050    107     65      16      312
206     311     238
PQSS47  PQSS    -72.28671667    46.64313333     45      105     24      10975   253
-175    428     167     -35     179     -105    1050    107     65      16      312
206     311     238
PQSS48  PQSS    -72.28671667    46.64313333     45      105     24      10975   253
-175    428     167     -35     179     -105    1050    107     65      16      312
206     311     238
PQSS49  PQSS    -72.28671667    46.64313333     45      105     24      10975   253
-175    428     167     -35     179     -105    1050    107     65      16      312
206     311     238
PQSS50  PQSS    -72.28671667    46.64313333     45      105     24      10975   253
-175    428     167     -35     179     -105    1050    107     65      16      312
206     311     238
PQLP01  PQLP    -75.90966389    45.56239722     49      102     24      10976   254
-168    422     183     -86     183     -101    889     85      57      13      253
180     253     199
PQLP02  PQLP    -75.90966389    45.56239722     49      102     24      10976   254
-168    422     183     -86     183     -101    889     85      57      13      253
180     253     199
PQLP03  PQLP    -75.90966389    45.56239722     49      102     24      10976   254
-168    422     183     -86     183     -101    889     85      57      13      253
180     253     199
PQLP04  PQLP    -75.90966389    45.56239722     49      102     24      10976   254
-168    422     183     -86     183     -101    889     85      57      13      253
180     253     199
PQLP05  PQLP    -75.90966389    45.56239722     49      102     24      10976   254
-168    422     183     -86     183     -101    889     85      57      13      253
180     253     199
PQLP06  PQLP    -75.90966389    45.56239722     49      102     24      10976   254
-168    422     183     -86     183     -101    889     85      57      13      253
180     253     199
PQLP07  PQLP    -75.90966389    45.56239722     49      102     24      10976   254
-168    422     183     -86     183     -101    889     85      57      13      253
180     253     199
PQLP08  PQLP    -75.90966389    45.56239722     49      102     24      10976   254
-168    422     183     -86     183     -101    889     85      57      13      253
180     253     199
PQLP09  PQLP    -75.90966389    45.56239722     49      102     24      10976   254
-168    422     183     -86     183     -101    889     85      57      13      253
180     253     199
PQLP10  PQLP    -75.90966389    45.56239722     49      102     24      10976   254
-168    422     183     -86     183     -101    889     85      57      13      253
180     253     199
PQLP11  PQLP    -75.90966389    45.56239722     49      102     24      10976   254
-168    422     183     -86     183     -101    889     85      57      13      253
180     253     199
PQLP12  PQLP    -75.90966389    45.56239722     49      102     24      10976   254
-168    422     183     -86     183     -101    889     85      57      13      253
180     253     199
PQLP13  PQLP    -75.90966389    45.56239722     49      102     24      10976   254
-168    422     183     -86     183     -101    889     85      57      13      253
180     253     199
PQLP14  PQLP    -75.90966389    45.56239722     49      102     24      10976   254
-168    422     183     -86     183     -101    889     85      57      13      253
```

```
180     253     199
PQLP15  PQLP    -75.90966389    45.56239722     49      102     24      10976   254
-168    422     183     -86     183     -101    889     85      57      13      253
180     253     199
PQLP16  PQLP    -75.90966389    45.56239722     49      102     24      10976   254
-168    422     183     -86     183     -101    889     85      57      13      253
180     253     199
PQLP17  PQLP    -75.90966389    45.56239722     49      102     24      10976   254
-168    422     183     -86     183     -101    889     85      57      13      253
180     253     199
PQLP18  PQLP    -75.90966389    45.56239722     49      102     24      10976   254
-168    422     183     -86     183     -101    889     85      57      13      253
180     253     199
PQLP19  PQLP    -75.90966389    45.56239722     49      102     24      10976   254
-168    422     183     -86     183     -101    889     85      57      13      253
180     253     199
PQLP20  PQLP    -75.90966389    45.56239722     49      102     24      10976   254
-168    422     183     -86     183     -101    889     85      57      13      253
180     253     199
PQLP21  PQLP    -75.90966389    45.56239722     49      102     24      10976   254
-168    422     183     -86     183     -101    889     85      57      13      253
180     253     199
PQLP22  PQLP    -75.90966389    45.56239722     49      102     24      10976   254
-168    422     183     -86     183     -101    889     85      57      13      253
180     253     199
PQLP23  PQLP    -75.90966389    45.56239722     49      102     24      10976   254
-168    422     183     -86     183     -101    889     85      57      13      253
180     253     199
PQLP24  PQLP    -75.90966389    45.56239722     49      102     24      10976   254
-168    422     183     -86     183     -101    889     85      57      13      253
180     253     199
PQLP25  PQLP    -75.90966389    45.56239722     49      102     24      10976   254
-168    422     183     -86     183     -101    889     85      57      13      253
180     253     199
PQLP26  PQLP    -75.90966389    45.56239722     49      102     24      10976   254
-168    422     183     -86     183     -101    889     85      57      13      253
180     253     199
PQLP27  PQLP    -75.90966389    45.56239722     49      102     24      10976   254
-168    422     183     -86     183     -101    889     85      57      13      253
180     253     199
PQLP28  PQLP    -75.90966389    45.56239722     49      102     24      10976   254
-168    422     183     -86     183     -101    889     85      57      13      253
180     253     199
PQLP29  PQLP    -75.90966389    45.56239722     49      102     24      10976   254
-168    422     183     -86     183     -101    889     85      57      13      253
180     253     199
PQLP30  PQLP    -75.90966389    45.56239722     49      102     24      10976   254
-168    422     183     -86     183     -101    889     85      57      13      253
180     253     199
PQLP31  PQLP    -75.90966389    45.56239722     49      102     24      10976   254
-168    422     183     -86     183     -101    889     85      57      13      253
180     253     199
PQLP32  PQLP    -75.90966389    45.56239722     49      102     24      10976   254
-168    422     183     -86     183     -101    889     85      57      13      253
180     253     199
PQLP33  PQLP    -75.90966389    45.56239722     49      102     24      10976   254
-168    422     183     -86     183     -101    889     85      57      13      253
180     253     199
PQLP34  PQLP    -75.90966389    45.56239722     49      102     24      10976   254
-168    422     183     -86     183     -101    889     85      57      13      253
180     253     199
PQLP35  PQLP    -75.90966389    45.56239722     49      102     24      10976   254
-168    422     183     -86     183     -101    889     85      57      13      253
```

```
180	253	199
PQLP36	PQLP	-75.90966389	45.56239722	49	102	24	10976	254
-168	422	183	-86	183	-101	889	85	57	13	253
180	253	199
PQLP37	PQLP	-75.90966389	45.56239722	49	102	24	10976	254
-168	422	183	-86	183	-101	889	85	57	13	253
180	253	199
PQLP38	PQLP	-75.90966389	45.56239722	49	102	24	10976	254
-168	422	183	-86	183	-101	889	85	57	13	253
180	253	199
PQLP39	PQLP	-75.90966389	45.56239722	49	102	24	10976	254
-168	422	183	-86	183	-101	889	85	57	13	253
180	253	199
PQLP40	PQLP	-75.90966389	45.56239722	49	102	24	10976	254
-168	422	183	-86	183	-101	889	85	57	13	253
180	253	199
PQLP41	PQLP	-75.90966389	45.56239722	49	102	24	10976	254
-168	422	183	-86	183	-101	889	85	57	13	253
180	253	199
PQLP42	PQLP	-75.90966389	45.56239722	49	102	24	10976	254
-168	422	183	-86	183	-101	889	85	57	13	253
180	253	199
PQLP43	PQLP	-75.90966389	45.56239722	49	102	24	10976	254
-168	422	183	-86	183	-101	889	85	57	13	253
180	253	199
PQLP44	PQLP	-75.90966389	45.56239722	49	102	24	10976	254
-168	422	183	-86	183	-101	889	85	57	13	253
180	253	199
PQLP45	PQLP	-75.90966389	45.56239722	49	102	24	10976	254
-168	422	183	-86	183	-101	889	85	57	13	253
180	253	199
PQLP46	PQLP	-75.90966389	45.56239722	49	102	24	10976	254
-168	422	183	-86	183	-101	889	85	57	13	253
180	253	199
PQLP47	PQLP	-75.90966389	45.56239722	49	102	24	10976	254
-168	422	183	-86	183	-101	889	85	57	13	253
180	253	199
PQLP48	PQLP	-75.90966389	45.56239722	49	102	24	10976	254
-168	422	183	-86	183	-101	889	85	57	13	253
180	253	199
PQLP49	PQLP	-75.90966389	45.56239722	49	102	24	10976	254
-168	422	183	-86	183	-101	889	85	57	13	253
180	253	199
PQLP50	PQLP	-75.90966389	45.56239722	49	102	24	10976	254
-168	422	183	-86	183	-101	889	85	57	13	253
180	253	199
ONML01	ONML	-79.66028056	45.02016667	55	110	28	9944	252
-140	392	-45	-23	177	-78	1004	110	66	17	307
205	231	275
ONML02	ONML	-79.66028056	45.02016667	55	110	28	9944	252
-140	392	-45	-23	177	-78	1004	110	66	17	307
205	231	275
ONML03	ONML	-79.66028056	45.02016667	55	110	28	9944	252
-140	392	-45	-23	177	-78	1004	110	66	17	307
205	231	275
ONML04	ONML	-79.66028056	45.02016667	55	110	28	9944	252
-140	392	-45	-23	177	-78	1004	110	66	17	307
205	231	275
ONML05	ONML	-79.66028056	45.02016667	55	110	28	9944	252
-140	392	-45	-23	177	-78	1004	110	66	17	307
205	231	275
ONML06	ONML	-79.66028056	45.02016667	55	110	28	9944	252
-140	392	-45	-23	177	-78	1004	110	66	17	307
```

205	231	275								
ONML07	ONML	-79.66028056		45.02016667		55	110	28	9944	252
-140	392	-45	-23	177	-78	1004	110	66	17	307
205	231	275								
ONML08	ONML	-79.66028056		45.02016667		55	110	28	9944	252
-140	392	-45	-23	177	-78	1004	110	66	17	307
205	231	275								
ONML09	ONML	-79.66028056		45.02016667		55	110	28	9944	252
-140	392	-45	-23	177	-78	1004	110	66	17	307
205	231	275								
ONML10	ONML	-79.66028056		45.02016667		55	110	28	9944	252
-140	392	-45	-23	177	-78	1004	110	66	17	307
205	231	275								
ONML11	ONML	-79.66028056		45.02016667		55	110	28	9944	252
-140	392	-45	-23	177	-78	1004	110	66	17	307
205	231	275								
ONML12	ONML	-79.66028056		45.02016667		55	110	28	9944	252
-140	392	-45	-23	177	-78	1004	110	66	17	307
205	231	275								
ONML13	ONML	-79.66028056		45.02016667		55	110	28	9944	252
-140	392	-45	-23	177	-78	1004	110	66	17	307
205	231	275								
ONML14	ONML	-79.66028056		45.02016667		55	110	28	9944	252
-140	392	-45	-23	177	-78	1004	110	66	17	307
205	231	275								
ONML15	ONML	-79.66028056		45.02016667		55	110	28	9944	252
-140	392	-45	-23	177	-78	1004	110	66	17	307
205	231	275								
ONML16	ONML	-79.66028056		45.02016667		55	110	28	9944	252
-140	392	-45	-23	177	-78	1004	110	66	17	307
205	231	275								
ONML17	ONML	-79.66028056		45.02016667		55	110	28	9944	252
-140	392	-45	-23	177	-78	1004	110	66	17	307
205	231	275								
ONML18	ONML	-79.66028056		45.02016667		55	110	28	9944	252
-140	392	-45	-23	177	-78	1004	110	66	17	307
205	231	275								
ONML19	ONML	-79.66028056		45.02016667		55	110	28	9944	252
-140	392	-45	-23	177	-78	1004	110	66	17	307
205	231	275								
ONML20	ONML	-79.66028056		45.02016667		55	110	28	9944	252
-140	392	-45	-23	177	-78	1004	110	66	17	307
205	231	275								
ONML21	ONML	-79.66028056		45.02016667		55	110	28	9944	252
-140	392	-45	-23	177	-78	1004	110	66	17	307
205	231	275								
ONML22	ONML	-79.66028056		45.02016667		55	110	28	9944	252
-140	392	-45	-23	177	-78	1004	110	66	17	307
205	231	275								
ONML23	ONML	-79.66028056		45.02016667		55	110	28	9944	252
-140	392	-45	-23	177	-78	1004	110	66	17	307
205	231	275								
ONML24	ONML	-79.66028056		45.02016667		55	110	28	9944	252
-140	392	-45	-23	177	-78	1004	110	66	17	307
205	231	275								
ONML25	ONML	-79.66028056		45.02016667		55	110	28	9944	252
-140	392	-45	-23	177	-78	1004	110	66	17	307
205	231	275								
ONML26	ONML	-79.66028056		45.02016667		55	110	28	9944	252
-140	392	-45	-23	177	-78	1004	110	66	17	307
205	231	275								
ONML27	ONML	-79.66028056		45.02016667		55	110	28	9944	252
-140	392	-45	-23	177	-78	1004	110	66	17	307

205	231	275								
ONML28	ONML	-79.66028056		45.02016667		55	110	28	9944	252
-140	392	-45	-23	177	-78	1004	110	66	17	307
205	231	275								
ONML29	ONML	-79.66028056		45.02016667		55	110	28	9944	252
-140	392	-45	-23	177	-78	1004	110	66	17	307
205	231	275								
ONML30	ONML	-79.66028056		45.02016667		55	110	28	9944	252
-140	392	-45	-23	177	-78	1004	110	66	17	307
205	231	275								
ONML31	ONML	-79.66028056		45.02016667		55	110	28	9944	252
-140	392	-45	-23	177	-78	1004	110	66	17	307
205	231	275								
ONML32	ONML	-79.66028056		45.02016667		55	110	28	9944	252
-140	392	-45	-23	177	-78	1004	110	66	17	307
205	231	275								
ONML33	ONML	-79.66028056		45.02016667		55	110	28	9944	252
-140	392	-45	-23	177	-78	1004	110	66	17	307
205	231	275								
ONML34	ONML	-79.66028056		45.02016667		55	110	28	9944	252
-140	392	-45	-23	177	-78	1004	110	66	17	307
205	231	275								
ONML35	ONML	-79.66028056		45.02016667		55	110	28	9944	252
-140	392	-45	-23	177	-78	1004	110	66	17	307
205	231	275								
ONML36	ONML	-79.66028056		45.02016667		55	110	28	9944	252
-140	392	-45	-23	177	-78	1004	110	66	17	307
205	231	275								
ONML37	ONML	-79.66028056		45.02016667		55	110	28	9944	252
-140	392	-45	-23	177	-78	1004	110	66	17	307
205	231	275								
ONML38	ONML	-79.66028056		45.02016667		55	110	28	9944	252
-140	392	-45	-23	177	-78	1004	110	66	17	307
205	231	275								
ONML39	ONML	-79.66028056		45.02016667		55	110	28	9944	252
-140	392	-45	-23	177	-78	1004	110	66	17	307
205	231	275								
ONML40	ONML	-79.66028056		45.02016667		55	110	28	9944	252
-140	392	-45	-23	177	-78	1004	110	66	17	307
205	231	275								
ONML41	ONML	-79.66028056		45.02016667		55	110	28	9944	252
-140	392	-45	-23	177	-78	1004	110	66	17	307
205	231	275								
ONML42	ONML	-79.66028056		45.02016667		55	110	28	9944	252
-140	392	-45	-23	177	-78	1004	110	66	17	307
205	231	275								
ONML43	ONML	-79.66028056		45.02016667		55	110	28	9944	252
-140	392	-45	-23	177	-78	1004	110	66	17	307
205	231	275								
ONML44	ONML	-79.66028056		45.02016667		55	110	28	9944	252
-140	392	-45	-23	177	-78	1004	110	66	17	307
205	231	275								
ONML45	ONML	-79.66028056		45.02016667		55	110	28	9944	252
-140	392	-45	-23	177	-78	1004	110	66	17	307
205	231	275								
ONML46	ONML	-79.66028056		45.02016667		55	110	28	9944	252
-140	392	-45	-23	177	-78	1004	110	66	17	307
205	231	275								
ONML47	ONML	-79.66028056		45.02016667		55	110	28	9944	252
-140	392	-45	-23	177	-78	1004	110	66	17	307
205	231	275								
ONML48	ONML	-79.66028056		45.02016667		55	110	28	9944	252
-140	392	-45	-23	177	-78	1004	110	66	17	307

205	231	275								
ONML49	ONML	-79.66028056		45.02016667		55	110	28	9944	252
-140	392	-45	-23	177	-78	1004	110	66	17	307
205	231	275								
ONML50	ONML	-79.66028056		45.02016667		55	110	28	9944	252
-140	392	-45	-23	177	-78	1004	110	66	17	307
205	231	275								
ONFR01	ONFR	-80.28425833		46.05228889		49	102	24	10600	250
-162	412	72	-32	179	-93	925	99	61	15	272
187	231	226								
ONFR02	ONFR	-80.28425833		46.05228889		49	102	24	10600	250
-162	412	72	-32	179	-93	925	99	61	15	272
187	231	226								
ONFR03	ONFR	-80.28425833		46.05228889		49	102	24	10600	250
-162	412	72	-32	179	-93	925	99	61	15	272
187	231	226								
ONFR04	ONFR	-80.28425833		46.05228889		49	102	24	10600	250
-162	412	72	-32	179	-93	925	99	61	15	272
187	231	226								
ONFR05	ONFR	-80.28425833		46.05228889		49	102	24	10600	250
-162	412	72	-32	179	-93	925	99	61	15	272
187	231	226								
ONFR06	ONFR	-80.28425833		46.05228889		49	102	24	10600	250
-162	412	72	-32	179	-93	925	99	61	15	272
187	231	226								
ONFR07	ONFR	-80.28425833		46.05228889		49	102	24	10600	250
-162	412	72	-32	179	-93	925	99	61	15	272
187	231	226								
ONFR08	ONFR	-80.28425833		46.05228889		49	102	24	10600	250
-162	412	72	-32	179	-93	925	99	61	15	272
187	231	226								
ONFR09	ONFR	-80.28425833		46.05228889		49	102	24	10600	250
-162	412	72	-32	179	-93	925	99	61	15	272
187	231	226								
ONFR10	ONFR	-80.28425833		46.05228889		49	102	24	10600	250
-162	412	72	-32	179	-93	925	99	61	15	272
187	231	226								
ONFR11	ONFR	-80.28425833		46.05228889		49	102	24	10600	250
-162	412	72	-32	179	-93	925	99	61	15	272
187	231	226								
ONFR12	ONFR	-80.28425833		46.05228889		49	102	24	10600	250
-162	412	72	-32	179	-93	925	99	61	15	272
187	231	226								
ONFR13	ONFR	-80.28425833		46.05228889		49	102	24	10600	250
-162	412	72	-32	179	-93	925	99	61	15	272
187	231	226								
ONFR14	ONFR	-80.28425833		46.05228889		49	102	24	10600	250
-162	412	72	-32	179	-93	925	99	61	15	272
187	231	226								
ONFR15	ONFR	-80.28425833		46.05228889		49	102	24	10600	250
-162	412	72	-32	179	-93	925	99	61	15	272
187	231	226								
ONFR16	ONFR	-80.28425833		46.05228889		49	102	24	10600	250
-162	412	72	-32	179	-93	925	99	61	15	272
187	231	226								
ONFR17	ONFR	-80.28425833		46.05228889		49	102	24	10600	250
-162	412	72	-32	179	-93	925	99	61	15	272
187	231	226								
ONFR18	ONFR	-80.28425833		46.05228889		49	102	24	10600	250
-162	412	72	-32	179	-93	925	99	61	15	272
187	231	226								
ONFR19	ONFR	-80.28425833		46.05228889		49	102	24	10600	250
-162	412	72	-32	179	-93	925	99	61	15	272

187	231	226								
ONFR20	ONFR	-80.28425833		46.05228889		49	102	24	10600	250
-162	412	72	-32	179	-93	925	99	61	15	272
187	231	226								
ONFR21	ONFR	-80.28425833		46.05228889		49	102	24	10600	250
-162	412	72	-32	179	-93	925	99	61	15	272
187	231	226								
ONFR22	ONFR	-80.28425833		46.05228889		49	102	24	10600	250
-162	412	72	-32	179	-93	925	99	61	15	272
187	231	226								
ONFR23	ONFR	-80.28425833		46.05228889		49	102	24	10600	250
-162	412	72	-32	179	-93	925	99	61	15	272
187	231	226								
ONFR24	ONFR	-80.28425833		46.05228889		49	102	24	10600	250
-162	412	72	-32	179	-93	925	99	61	15	272
187	231	226								
ONFR25	ONFR	-80.28425833		46.05228889		49	102	24	10600	250
-162	412	72	-32	179	-93	925	99	61	15	272
187	231	226								
ONFR26	ONFR	-80.28425833		46.05228889		49	102	24	10600	250
-162	412	72	-32	179	-93	925	99	61	15	272
187	231	226								
ONFR27	ONFR	-80.28425833		46.05228889		49	102	24	10600	250
-162	412	72	-32	179	-93	925	99	61	15	272
187	231	226								
ONFR28	ONFR	-80.28425833		46.05228889		49	102	24	10600	250
-162	412	72	-32	179	-93	925	99	61	15	272
187	231	226								
ONFR29	ONFR	-80.28425833		46.05228889		49	102	24	10600	250
-162	412	72	-32	179	-93	925	99	61	15	272
187	231	226								
ONFR30	ONFR	-80.28425833		46.05228889		49	102	24	10600	250
-162	412	72	-32	179	-93	925	99	61	15	272
187	231	226								
ONFR31	ONFR	-80.28425833		46.05228889		49	102	24	10600	250
-162	412	72	-32	179	-93	925	99	61	15	272
187	231	226								
ONFR32	ONFR	-80.28425833		46.05228889		49	102	24	10600	250
-162	412	72	-32	179	-93	925	99	61	15	272
187	231	226								
ONFR33	ONFR	-80.28425833		46.05228889		49	102	24	10600	250
-162	412	72	-32	179	-93	925	99	61	15	272
187	231	226								
ONFR34	ONFR	-80.28425833		46.05228889		49	102	24	10600	250
-162	412	72	-32	179	-93	925	99	61	15	272
187	231	226								
ONFR35	ONFR	-80.28425833		46.05228889		49	102	24	10600	250
-162	412	72	-32	179	-93	925	99	61	15	272
187	231	226								
ONFR36	ONFR	-80.28425833		46.05228889		49	102	24	10600	250
-162	412	72	-32	179	-93	925	99	61	15	272
187	231	226								
ONFR37	ONFR	-80.28425833		46.05228889		49	102	24	10600	250
-162	412	72	-32	179	-93	925	99	61	15	272
187	231	226								
ONFR38	ONFR	-80.28425833		46.05228889		49	102	24	10600	250
-162	412	72	-32	179	-93	925	99	61	15	272
187	231	226								
ONFR39	ONFR	-80.28425833		46.05228889		49	102	24	10600	250
-162	412	72	-32	179	-93	925	99	61	15	272
187	231	226								
ONFR40	ONFR	-80.28425833		46.05228889		49	102	24	10600	250
-162	412	72	-32	179	-93	925	99	61	15	272

187	231	226								
ONFR41	ONFR	-80.28425833		46.05228889		49	102	24	10600	250
-162	412	72	-32	179	-93	925	99	61	15	272
187	231	226								
ONFR42	ONFR	-80.28425833		46.05228889		49	102	24	10600	250
-162	412	72	-32	179	-93	925	99	61	15	272
187	231	226								
ONFR43	ONFR	-80.28425833		46.05228889		49	102	24	10600	250
-162	412	72	-32	179	-93	925	99	61	15	272
187	231	226								
ONFR44	ONFR	-80.28425833		46.05228889		49	102	24	10600	250
-162	412	72	-32	179	-93	925	99	61	15	272
187	231	226								
ONFR45	ONFR	-80.28425833		46.05228889		49	102	24	10600	250
-162	412	72	-32	179	-93	925	99	61	15	272
187	231	226								
ONFR46	ONFR	-80.28425833		46.05228889		49	102	24	10600	250
-162	412	72	-32	179	-93	925	99	61	15	272
187	231	226								
ONFR47	ONFR	-80.28425833		46.05228889		49	102	24	10600	250
-162	412	72	-32	179	-93	925	99	61	15	272
187	231	226								
ONFR48	ONFR	-80.28425833		46.05228889		49	102	24	10600	250
-162	412	72	-32	179	-93	925	99	61	15	272
187	231	226								
ONFR49	ONFR	-80.28425833		46.05228889		49	102	24	10600	250
-162	412	72	-32	179	-93	925	99	61	15	272
187	231	226								
ONFR50	ONFR	-80.28425833		46.05228889		49	102	24	10600	250
-162	412	72	-32	179	-93	925	99	61	15	272
187	231	226								
ONHF01	ONHF	-78.07988333		44.59736667		57	106	26	10062	255
-143	398	78	-67	181	-79	843	81	57	10	231
179	218	192								
ONHF02	ONHF	-78.07988333		44.59736667		57	106	26	10062	255
-143	398	78	-67	181	-79	843	81	57	10	231
179	218	192								
ONHF03	ONHF	-78.07988333		44.59736667		57	106	26	10062	255
-143	398	78	-67	181	-79	843	81	57	10	231
179	218	192								
ONHF04	ONHF	-78.07988333		44.59736667		57	106	26	10062	255
-143	398	78	-67	181	-79	843	81	57	10	231
179	218	192								
ONHF05	ONHF	-78.07988333		44.59736667		57	106	26	10062	255
-143	398	78	-67	181	-79	843	81	57	10	231
179	218	192								
ONHF06	ONHF	-78.07988333		44.59736667		57	106	26	10062	255
-143	398	78	-67	181	-79	843	81	57	10	231
179	218	192								
ONHF07	ONHF	-78.07988333		44.59736667		57	106	26	10062	255
-143	398	78	-67	181	-79	843	81	57	10	231
179	218	192								
ONHF08	ONHF	-78.07988333		44.59736667		57	106	26	10062	255
-143	398	78	-67	181	-79	843	81	57	10	231
179	218	192								
ONHF09	ONHF	-78.07988333		44.59736667		57	106	26	10062	255
-143	398	78	-67	181	-79	843	81	57	10	231
179	218	192								
ONHF10	ONHF	-78.07988333		44.59736667		57	106	26	10062	255
-143	398	78	-67	181	-79	843	81	57	10	231
179	218	192								
ONHF11	ONHF	-78.07988333		44.59736667		57	106	26	10062	255
-143	398	78	-67	181	-79	843	81	57	10	231

179	218	192								
ONHF12	ONHF	-78.07988333		44.59736667		57	106	26	10062	255
-143	398	78	-67	181	-79	843	81	57	10	231
179	218	192								
ONHF13	ONHF	-78.07988333		44.59736667		57	106	26	10062	255
-143	398	78	-67	181	-79	843	81	57	10	231
179	218	192								
ONHF14	ONHF	-78.07988333		44.59736667		57	106	26	10062	255
-143	398	78	-67	181	-79	843	81	57	10	231
179	218	192								
ONHF15	ONHF	-78.07988333		44.59736667		57	106	26	10062	255
-143	398	78	-67	181	-79	843	81	57	10	231
179	218	192								
ONHF16	ONHF	-78.07988333		44.59736667		57	106	26	10062	255
-143	398	78	-67	181	-79	843	81	57	10	231
179	218	192								
ONHF17	ONHF	-78.07988333		44.59736667		57	106	26	10062	255
-143	398	78	-67	181	-79	843	81	57	10	231
179	218	192								
ONHF18	ONHF	-78.07988333		44.59736667		57	106	26	10062	255
-143	398	78	-67	181	-79	843	81	57	10	231
179	218	192								
ONHF19	ONHF	-78.07988333		44.59736667		57	106	26	10062	255
-143	398	78	-67	181	-79	843	81	57	10	231
179	218	192								
ONHF20	ONHF	-78.07988333		44.59736667		57	106	26	10062	255
-143	398	78	-67	181	-79	843	81	57	10	231
179	218	192								
ONHF21	ONHF	-78.07988333		44.59736667		57	106	26	10062	255
-143	398	78	-67	181	-79	843	81	57	10	231
179	218	192								
ONHF22	ONHF	-78.07988333		44.59736667		57	106	26	10062	255
-143	398	78	-67	181	-79	843	81	57	10	231
179	218	192								
ONHF23	ONHF	-78.07988333		44.59736667		57	106	26	10062	255
-143	398	78	-67	181	-79	843	81	57	10	231
179	218	192								
ONHF24	ONHF	-78.07988333		44.59736667		57	106	26	10062	255
-143	398	78	-67	181	-79	843	81	57	10	231
179	218	192								
ONHF25	ONHF	-78.07988333		44.59736667		57	106	26	10062	255
-143	398	78	-67	181	-79	843	81	57	10	231
179	218	192								
ONHF26	ONHF	-78.07988333		44.59736667		57	106	26	10062	255
-143	398	78	-67	181	-79	843	81	57	10	231
179	218	192								
ONHF27	ONHF	-78.07988333		44.59736667		57	106	26	10062	255
-143	398	78	-67	181	-79	843	81	57	10	231
179	218	192								
ONHF28	ONHF	-78.07988333		44.59736667		57	106	26	10062	255
-143	398	78	-67	181	-79	843	81	57	10	231
179	218	192								
ONHF29	ONHF	-78.07988333		44.59736667		57	106	26	10062	255
-143	398	78	-67	181	-79	843	81	57	10	231
179	218	192								
ONHF30	ONHF	-78.07988333		44.59736667		57	106	26	10062	255
-143	398	78	-67	181	-79	843	81	57	10	231
179	218	192								
ONHF31	ONHF	-78.07988333		44.59736667		57	106	26	10062	255
-143	398	78	-67	181	-79	843	81	57	10	231
179	218	192								
ONHF32	ONHF	-78.07988333		44.59736667		57	106	26	10062	255
-143	398	78	-67	181	-79	843	81	57	10	231

179	218	192								
ONHF33	ONHF	-78.07988333		44.59736667		57	106	26	10062	255
-143	398	78	-67	181	-79	843	81	57	10	231
179	218	192								
ONHF34	ONHF	-78.07988333		44.59736667		57	106	26	10062	255
-143	398	78	-67	181	-79	843	81	57	10	231
179	218	192								
ONHF35	ONHF	-78.07988333		44.59736667		57	106	26	10062	255
-143	398	78	-67	181	-79	843	81	57	10	231
179	218	192								
ONHF36	ONHF	-78.07988333		44.59736667		57	106	26	10062	255
-143	398	78	-67	181	-79	843	81	57	10	231
179	218	192								
ONHF37	ONHF	-78.07988333		44.59736667		57	106	26	10062	255
-143	398	78	-67	181	-79	843	81	57	10	231
179	218	192								
ONHF38	ONHF	-78.07988333		44.59736667		57	106	26	10062	255
-143	398	78	-67	181	-79	843	81	57	10	231
179	218	192								
ONHF39	ONHF	-78.07988333		44.59736667		57	106	26	10062	255
-143	398	78	-67	181	-79	843	81	57	10	231
179	218	192								
ONHF40	ONHF	-78.07988333		44.59736667		57	106	26	10062	255
-143	398	78	-67	181	-79	843	81	57	10	231
179	218	192								
ONHF41	ONHF	-78.07988333		44.59736667		57	106	26	10062	255
-143	398	78	-67	181	-79	843	81	57	10	231
179	218	192								
ONHF42	ONHF	-78.07988333		44.59736667		57	106	26	10062	255
-143	398	78	-67	181	-79	843	81	57	10	231
179	218	192								
ONHF43	ONHF	-78.07988333		44.59736667		57	106	26	10062	255
-143	398	78	-67	181	-79	843	81	57	10	231
179	218	192								
ONHF44	ONHF	-78.07988333		44.59736667		57	106	26	10062	255
-143	398	78	-67	181	-79	843	81	57	10	231
179	218	192								
ONHF45	ONHF	-78.07988333		44.59736667		57	106	26	10062	255
-143	398	78	-67	181	-79	843	81	57	10	231
179	218	192								
ONHF46	ONHF	-78.07988333		44.59736667		57	106	26	10062	255
-143	398	78	-67	181	-79	843	81	57	10	231
179	218	192								
ONHF47	ONHF	-78.07988333		44.59736667		57	106	26	10062	255
-143	398	78	-67	181	-79	843	81	57	10	231
179	218	192								
ONHF48	ONHF	-78.07988333		44.59736667		57	106	26	10062	255
-143	398	78	-67	181	-79	843	81	57	10	231
179	218	192								
ONHF49	ONHF	-78.07988333		44.59736667		57	106	26	10062	255
-143	398	78	-67	181	-79	843	81	57	10	231
179	218	192								
ONHF50	ONHF	-78.07988333		44.59736667		57	106	26	10062	255
-143	398	78	-67	181	-79	843	81	57	10	231
179	218	192								
ONGR01	ONGR	-84.22216111		46.749225		34	107	26	10146	230
-168	398	115	-46	156	-104	984	101	58	18	292
183	260	239								
ONGR02	ONGR	-84.22216111		46.749225		34	107	26	10146	230
-168	398	115	-46	156	-104	984	101	58	18	292
183	260	239								
ONGR03	ONGR	-84.22216111		46.749225		34	107	26	10146	230
-168	398	115	-46	156	-104	984	101	58	18	292

183	260	239								
ONGR04	ONGR	-84.22216111	46.749225	34	107	26	10146	230		
-168	398	115	-46	156	-104	984	101	58	18	292
183	260	239								
ONGR05	ONGR	-84.22216111	46.749225	34	107	26	10146	230		
-168	398	115	-46	156	-104	984	101	58	18	292
183	260	239								
ONGR06	ONGR	-84.22216111	46.749225	34	107	26	10146	230		
-168	398	115	-46	156	-104	984	101	58	18	292
183	260	239								
ONGR07	ONGR	-84.22216111	46.749225	34	107	26	10146	230		
-168	398	115	-46	156	-104	984	101	58	18	292
183	260	239								
ONGR08	ONGR	-84.22216111	46.749225	34	107	26	10146	230		
-168	398	115	-46	156	-104	984	101	58	18	292
183	260	239								
ONGR09	ONGR	-84.22216111	46.749225	34	107	26	10146	230		
-168	398	115	-46	156	-104	984	101	58	18	292
183	260	239								
ONGR10	ONGR	-84.22216111	46.749225	34	107	26	10146	230		
-168	398	115	-46	156	-104	984	101	58	18	292
183	260	239								
ONGR11	ONGR	-84.22216111	46.749225	34	107	26	10146	230		
-168	398	115	-46	156	-104	984	101	58	18	292
183	260	239								
ONGR12	ONGR	-84.22216111	46.749225	34	107	26	10146	230		
-168	398	115	-46	156	-104	984	101	58	18	292
183	260	239								
ONGR13	ONGR	-84.22216111	46.749225	34	107	26	10146	230		
-168	398	115	-46	156	-104	984	101	58	18	292
183	260	239								
ONGR14	ONGR	-84.22216111	46.749225	34	107	26	10146	230		
-168	398	115	-46	156	-104	984	101	58	18	292
183	260	239								
ONGR15	ONGR	-84.22216111	46.749225	34	107	26	10146	230		
-168	398	115	-46	156	-104	984	101	58	18	292
183	260	239								
ONGR16	ONGR	-84.22216111	46.749225	34	107	26	10146	230		
-168	398	115	-46	156	-104	984	101	58	18	292
183	260	239								
ONGR17	ONGR	-84.22216111	46.749225	34	107	26	10146	230		
-168	398	115	-46	156	-104	984	101	58	18	292
183	260	239								
ONGR18	ONGR	-84.22216111	46.749225	34	107	26	10146	230		
-168	398	115	-46	156	-104	984	101	58	18	292
183	260	239								
ONGR19	ONGR	-84.22216111	46.749225	34	107	26	10146	230		
-168	398	115	-46	156	-104	984	101	58	18	292
183	260	239								
ONGR20	ONGR	-84.22216111	46.749225	34	107	26	10146	230		
-168	398	115	-46	156	-104	984	101	58	18	292
183	260	239								
ONGR21	ONGR	-84.22216111	46.749225	34	107	26	10146	230		
-168	398	115	-46	156	-104	984	101	58	18	292
183	260	239								
ONGR22	ONGR	-84.22216111	46.749225	34	107	26	10146	230		
-168	398	115	-46	156	-104	984	101	58	18	292
183	260	239								
ONGR23	ONGR	-84.22216111	46.749225	34	107	26	10146	230		
-168	398	115	-46	156	-104	984	101	58	18	292
183	260	239								
ONGR24	ONGR	-84.22216111	46.749225	34	107	26	10146	230		
-168	398	115	-46	156	-104	984	101	58	18	292

183	260	239								
ONGR25	ONGR	-84.22216111		46.749225		34	107	26	10146	230
-168	398	115	-46	156	-104	984	101	58	18	292
183	260	239								
ONGR26	ONGR	-84.22216111		46.749225		34	107	26	10146	230
-168	398	115	-46	156	-104	984	101	58	18	292
183	260	239								
ONGR27	ONGR	-84.22216111		46.749225		34	107	26	10146	230
-168	398	115	-46	156	-104	984	101	58	18	292
183	260	239								
ONGR28	ONGR	-84.22216111		46.749225		34	107	26	10146	230
-168	398	115	-46	156	-104	984	101	58	18	292
183	260	239								
ONGR29	ONGR	-84.22216111		46.749225		34	107	26	10146	230
-168	398	115	-46	156	-104	984	101	58	18	292
183	260	239								
ONGR30	ONGR	-84.22216111		46.749225		34	107	26	10146	230
-168	398	115	-46	156	-104	984	101	58	18	292
183	260	239								
ONGR31	ONGR	-84.22216111		46.749225		34	107	26	10146	230
-168	398	115	-46	156	-104	984	101	58	18	292
183	260	239								
ONGR32	ONGR	-84.22216111		46.749225		34	107	26	10146	230
-168	398	115	-46	156	-104	984	101	58	18	292
183	260	239								
ONGR33	ONGR	-84.22216111		46.749225		34	107	26	10146	230
-168	398	115	-46	156	-104	984	101	58	18	292
183	260	239								
ONGR34	ONGR	-84.22216111		46.749225		34	107	26	10146	230
-168	398	115	-46	156	-104	984	101	58	18	292
183	260	239								
ONGR35	ONGR	-84.22216111		46.749225		34	107	26	10146	230
-168	398	115	-46	156	-104	984	101	58	18	292
183	260	239								
ONGR36	ONGR	-84.22216111		46.749225		34	107	26	10146	230
-168	398	115	-46	156	-104	984	101	58	18	292
183	260	239								
ONGR37	ONGR	-84.22216111		46.749225		34	107	26	10146	230
-168	398	115	-46	156	-104	984	101	58	18	292
183	260	239								
ONGR38	ONGR	-84.22216111		46.749225		34	107	26	10146	230
-168	398	115	-46	156	-104	984	101	58	18	292
183	260	239								
ONGR39	ONGR	-84.22216111		46.749225		34	107	26	10146	230
-168	398	115	-46	156	-104	984	101	58	18	292
183	260	239								
ONGR40	ONGR	-84.22216111		46.749225		34	107	26	10146	230
-168	398	115	-46	156	-104	984	101	58	18	292
183	260	239								
ONGR41	ONGR	-84.22216111		46.749225		34	107	26	10146	230
-168	398	115	-46	156	-104	984	101	58	18	292
183	260	239								
ONGR42	ONGR	-84.22216111		46.749225		34	107	26	10146	230
-168	398	115	-46	156	-104	984	101	58	18	292
183	260	239								
ONGR43	ONGR	-84.22216111		46.749225		34	107	26	10146	230
-168	398	115	-46	156	-104	984	101	58	18	292
183	260	239								
ONGR44	ONGR	-84.22216111		46.749225		34	107	26	10146	230
-168	398	115	-46	156	-104	984	101	58	18	292
183	260	239								
ONGR45	ONGR	-84.22216111		46.749225		34	107	26	10146	230
-168	398	115	-46	156	-104	984	101	58	18	292

183	260	239								
ONGR46	ONGR	-84.22216111		46.749225		34	107	26	10146	230
-168	398	115	-46	156	-104	984	101	58	18	292
183	260	239								
ONGR47	ONGR	-84.22216111		46.749225		34	107	26	10146	230
-168	398	115	-46	156	-104	984	101	58	18	292
183	260	239								
ONGR48	ONGR	-84.22216111		46.749225		34	107	26	10146	230
-168	398	115	-46	156	-104	984	101	58	18	292
183	260	239								
ONGR49	ONGR	-84.22216111		46.749225		34	107	26	10146	230
-168	398	115	-46	156	-104	984	101	58	18	292
183	260	239								
ONGR50	ONGR	-84.22216111		46.749225		34	107	26	10146	230
-168	398	115	-46	156	-104	984	101	58	18	292
183	260	239								
ONMW01	ONMWF	-81.72323056		46.08695		44	103	25	10072	239
-160	399	125	-83	167	-91	841	96	51	17	254
171	216	187								
ONMW02	ONMWF	-81.72323056		46.08695		44	103	25	10072	239
-160	399	125	-83	167	-91	841	96	51	17	254
171	216	187								
ONMW03	ONMWF	-81.72323056		46.08695		44	103	25	10072	239
-160	399	125	-83	167	-91	841	96	51	17	254
171	216	187								
ONMW04	ONMWF	-81.72323056		46.08695		44	103	25	10072	239
-160	399	125	-83	167	-91	841	96	51	17	254
171	216	187								
ONMW05	ONMWF	-81.72323056		46.08695		44	103	25	10072	239
-160	399	125	-83	167	-91	841	96	51	17	254
171	216	187								
ONMW06	ONMWF	-81.72323056		46.08695		44	103	25	10072	239
-160	399	125	-83	167	-91	841	96	51	17	254
171	216	187								
ONMW07	ONMWF	-81.72323056		46.08695		44	103	25	10072	239
-160	399	125	-83	167	-91	841	96	51	17	254
171	216	187								
ONMW08	ONMWF	-81.72323056		46.08695		44	103	25	10072	239
-160	399	125	-83	167	-91	841	96	51	17	254
171	216	187								
ONMW09	ONMWF	-81.72323056		46.08695		44	103	25	10072	239
-160	399	125	-83	167	-91	841	96	51	17	254
171	216	187								
ONMW10	ONMWF	-81.72323056		46.08695		44	103	25	10072	239
-160	399	125	-83	167	-91	841	96	51	17	254
171	216	187								
ONMW11	ONMWF	-81.72323056		46.08695		44	103	25	10072	239
-160	399	125	-83	167	-91	841	96	51	17	254
171	216	187								
ONMW12	ONMWF	-81.72323056		46.08695		44	103	25	10072	239
-160	399	125	-83	167	-91	841	96	51	17	254
171	216	187								
ONMW13	ONMWF	-81.72323056		46.08695		44	103	25	10072	239
-160	399	125	-83	167	-91	841	96	51	17	254
171	216	187								
ONMW14	ONMWF	-81.72323056		46.08695		44	103	25	10072	239
-160	399	125	-83	167	-91	841	96	51	17	254
171	216	187								
ONMW15	ONMWF	-81.72323056		46.08695		44	103	25	10072	239
-160	399	125	-83	167	-91	841	96	51	17	254
171	216	187								
ONMW16	ONMWF	-81.72323056		46.08695		44	103	25	10072	239
-160	399	125	-83	167	-91	841	96	51	17	254

171	216	187								
ONMW17	ONMWF	-81.72323056	46.08695	44	103	25	10072	239		
-160	399	125	-83	167	-91	841	96	51	17	254
171	216	187								
ONMW18	ONMWF	-81.72323056	46.08695	44	103	25	10072	239		
-160	399	125	-83	167	-91	841	96	51	17	254
171	216	187								
ONMW19	ONMWF	-81.72323056	46.08695	44	103	25	10072	239		
-160	399	125	-83	167	-91	841	96	51	17	254
171	216	187								
ONMW20	ONMWF	-81.72323056	46.08695	44	103	25	10072	239		
-160	399	125	-83	167	-91	841	96	51	17	254
171	216	187								
ONMW21	ONMWF	-81.72323056	46.08695	44	103	25	10072	239		
-160	399	125	-83	167	-91	841	96	51	17	254
171	216	187								
ONMW22	ONMWF	-81.72323056	46.08695	44	103	25	10072	239		
-160	399	125	-83	167	-91	841	96	51	17	254
171	216	187								
ONMW23	ONMWF	-81.72323056	46.08695	44	103	25	10072	239		
-160	399	125	-83	167	-91	841	96	51	17	254
171	216	187								
ONMW24	ONMWF	-81.72323056	46.08695	44	103	25	10072	239		
-160	399	125	-83	167	-91	841	96	51	17	254
171	216	187								
ONMW25	ONMWF	-81.72323056	46.08695	44	103	25	10072	239		
-160	399	125	-83	167	-91	841	96	51	17	254
171	216	187								
ONMW26	ONMWF	-81.72323056	46.08695	44	103	25	10072	239		
-160	399	125	-83	167	-91	841	96	51	17	254
171	216	187								
ONMW27	ONMWF	-81.72323056	46.08695	44	103	25	10072	239		
-160	399	125	-83	167	-91	841	96	51	17	254
171	216	187								
ONMW28	ONMWF	-81.72323056	46.08695	44	103	25	10072	239		
-160	399	125	-83	167	-91	841	96	51	17	254
171	216	187								
ONMW29	ONMWF	-81.72323056	46.08695	44	103	25	10072	239		
-160	399	125	-83	167	-91	841	96	51	17	254
171	216	187								
ONMW30	ONMWF	-81.72323056	46.08695	44	103	25	10072	239		
-160	399	125	-83	167	-91	841	96	51	17	254
171	216	187								
ONMW31	ONMWF	-81.72323056	46.08695	44	103	25	10072	239		
-160	399	125	-83	167	-91	841	96	51	17	254
171	216	187								
ONMW32	ONMWF	-81.72323056	46.08695	44	103	25	10072	239		
-160	399	125	-83	167	-91	841	96	51	17	254
171	216	187								
ONMW33	ONMWF	-81.72323056	46.08695	44	103	25	10072	239		
-160	399	125	-83	167	-91	841	96	51	17	254
171	216	187								
ONMW34	ONMWF	-81.72323056	46.08695	44	103	25	10072	239		
-160	399	125	-83	167	-91	841	96	51	17	254
171	216	187								
ONMW35	ONMWF	-81.72323056	46.08695	44	103	25	10072	239		
-160	399	125	-83	167	-91	841	96	51	17	254
171	216	187								
ONMW36	ONMWF	-81.72323056	46.08695	44	103	25	10072	239		
-160	399	125	-83	167	-91	841	96	51	17	254
171	216	187								
ONMW37	ONMWF	-81.72323056	46.08695	44	103	25	10072	239		
-160	399	125	-83	167	-91	841	96	51	17	254

171	216	187								
ONMW38	ONMWF	-81.72323056		46.08695		44	103	25	10072	239
-160	399	125	-83	167	-91	841	96	51	17	254
171	216	187								
ONMW39	ONMWF	-81.72323056		46.08695		44	103	25	10072	239
-160	399	125	-83	167	-91	841	96	51	17	254
171	216	187								
ONMW40	ONMWF	-81.72323056		46.08695		44	103	25	10072	239
-160	399	125	-83	167	-91	841	96	51	17	254
171	216	187								
ONMW41	ONMWF	-81.72323056		46.08695		44	103	25	10072	239
-160	399	125	-83	167	-91	841	96	51	17	254
171	216	187								
ONMW42	ONMWF	-81.72323056		46.08695		44	103	25	10072	239
-160	399	125	-83	167	-91	841	96	51	17	254
171	216	187								
ONMW43	ONMWF	-81.72323056		46.08695		44	103	25	10072	239
-160	399	125	-83	167	-91	841	96	51	17	254
171	216	187								
ONMW44	ONMWF	-81.72323056		46.08695		44	103	25	10072	239
-160	399	125	-83	167	-91	841	96	51	17	254
171	216	187								
ONMW45	ONMWF	-81.72323056		46.08695		44	103	25	10072	239
-160	399	125	-83	167	-91	841	96	51	17	254
171	216	187								
ONMW46	ONMWF	-81.72323056		46.08695		44	103	25	10072	239
-160	399	125	-83	167	-91	841	96	51	17	254
171	216	187								
ONMW47	ONMWF	-81.72323056		46.08695		44	103	25	10072	239
-160	399	125	-83	167	-91	841	96	51	17	254
171	216	187								
ONMW48	ONMWF	-81.72323056		46.08695		44	103	25	10072	239
-160	399	125	-83	167	-91	841	96	51	17	254
171	216	187								
ONMW49	ONMWF	-81.72323056		46.08695		44	103	25	10072	239
-160	399	125	-83	167	-91	841	96	51	17	254
171	216	187								
ONMW50	ONMWF	-81.72323056		46.08695		44	103	25	10072	239
-160	399	125	-83	167	-91	841	96	51	17	254
171	216	187								
ONRC01	ONRC	-77.39611944		45.66363056		47	112	25	10859	259
-172	431	180	-88	180	-101	850	84	52	15	244
166	244	177								
ONRC02	ONRC	-77.39611944		45.66363056		47	112	25	10859	259
-172	431	180	-88	180	-101	850	84	52	15	244
166	244	177								
ONRC03	ONRC	-77.39611944		45.66363056		47	112	25	10859	259
-172	431	180	-88	180	-101	850	84	52	15	244
166	244	177								
ONRC04	ONRC	-77.39611944		45.66363056		47	112	25	10859	259
-172	431	180	-88	180	-101	850	84	52	15	244
166	244	177								
ONRC05	ONRC	-77.39611944		45.66363056		47	112	25	10859	259
-172	431	180	-88	180	-101	850	84	52	15	244
166	244	177								
ONRC06	ONRC	-77.39611944		45.66363056		47	112	25	10859	259
-172	431	180	-88	180	-101	850	84	52	15	244
166	244	177								
ONRC07	ONRC	-77.39611944		45.66363056		47	112	25	10859	259
-172	431	180	-88	180	-101	850	84	52	15	244
166	244	177								
ONRC08	ONRC	-77.39611944		45.66363056		47	112	25	10859	259
-172	431	180	-88	180	-101	850	84	52	15	244

```
166	244	177
ONRC09	ONRC	-77.39611944	45.66363056	47	112	25	10859	259
-172	431	180	-88	180	-101	850	84	52	15	244
166	244	177
ONRC10	ONRC	-77.39611944	45.66363056	47	112	25	10859	259
-172	431	180	-88	180	-101	850	84	52	15	244
166	244	177
ONRC11	ONRC	-77.39611944	45.66363056	47	112	25	10859	259
-172	431	180	-88	180	-101	850	84	52	15	244
166	244	177
ONRC12	ONRC	-77.39611944	45.66363056	47	112	25	10859	259
-172	431	180	-88	180	-101	850	84	52	15	244
166	244	177
ONRC13	ONRC	-77.39611944	45.66363056	47	112	25	10859	259
-172	431	180	-88	180	-101	850	84	52	15	244
166	244	177
ONRC14	ONRC	-77.39611944	45.66363056	47	112	25	10859	259
-172	431	180	-88	180	-101	850	84	52	15	244
166	244	177
ONRC15	ONRC	-77.39611944	45.66363056	47	112	25	10859	259
-172	431	180	-88	180	-101	850	84	52	15	244
166	244	177
ONRC16	ONRC	-77.39611944	45.66363056	47	112	25	10859	259
-172	431	180	-88	180	-101	850	84	52	15	244
166	244	177
ONRC17	ONRC	-77.39611944	45.66363056	47	112	25	10859	259
-172	431	180	-88	180	-101	850	84	52	15	244
166	244	177
ONRC18	ONRC	-77.39611944	45.66363056	47	112	25	10859	259
-172	431	180	-88	180	-101	850	84	52	15	244
166	244	177
ONRC19	ONRC	-77.39611944	45.66363056	47	112	25	10859	259
-172	431	180	-88	180	-101	850	84	52	15	244
166	244	177
ONRC20	ONRC	-77.39611944	45.66363056	47	112	25	10859	259
-172	431	180	-88	180	-101	850	84	52	15	244
166	244	177
ONRC21	ONRC	-77.39611944	45.66363056	47	112	25	10859	259
-172	431	180	-88	180	-101	850	84	52	15	244
166	244	177
ONRC22	ONRC	-77.39611944	45.66363056	47	112	25	10859	259
-172	431	180	-88	180	-101	850	84	52	15	244
166	244	177
ONRC23	ONRC	-77.39611944	45.66363056	47	112	25	10859	259
-172	431	180	-88	180	-101	850	84	52	15	244
166	244	177
ONRC24	ONRC	-77.39611944	45.66363056	47	112	25	10859	259
-172	431	180	-88	180	-101	850	84	52	15	244
166	244	177
ONRC25	ONRC	-77.39611944	45.66363056	47	112	25	10859	259
-172	431	180	-88	180	-101	850	84	52	15	244
166	244	177
ONRC26	ONRC	-77.39611944	45.66363056	47	112	25	10859	259
-172	431	180	-88	180	-101	850	84	52	15	244
166	244	177
ONRC27	ONRC	-77.39611944	45.66363056	47	112	25	10859	259
-172	431	180	-88	180	-101	850	84	52	15	244
166	244	177
ONRC28	ONRC	-77.39611944	45.66363056	47	112	25	10859	259
-172	431	180	-88	180	-101	850	84	52	15	244
166	244	177
ONRC29	ONRC	-77.39611944	45.66363056	47	112	25	10859	259
-172	431	180	-88	180	-101	850	84	52	15	244
```

166	244	177								
ONRC30	ONRC	-77.39611944		45.66363056		47	112	25	10859	259
-172	431	180	-88	180	-101	850	84	52	15	244
166	244	177								
ONRC31	ONRC	-77.39611944		45.66363056		47	112	25	10859	259
-172	431	180	-88	180	-101	850	84	52	15	244
166	244	177								
ONRC32	ONRC	-77.39611944		45.66363056		47	112	25	10859	259
-172	431	180	-88	180	-101	850	84	52	15	244
166	244	177								
ONRC33	ONRC	-77.39611944		45.66363056		47	112	25	10859	259
-172	431	180	-88	180	-101	850	84	52	15	244
166	244	177								
ONRC34	ONRC	-77.39611944		45.66363056		47	112	25	10859	259
-172	431	180	-88	180	-101	850	84	52	15	244
166	244	177								
ONRC35	ONRC	-77.39611944		45.66363056		47	112	25	10859	259
-172	431	180	-88	180	-101	850	84	52	15	244
166	244	177								
ONRC36	ONRC	-77.39611944		45.66363056		47	112	25	10859	259
-172	431	180	-88	180	-101	850	84	52	15	244
166	244	177								
ONRC37	ONRC	-77.39611944		45.66363056		47	112	25	10859	259
-172	431	180	-88	180	-101	850	84	52	15	244
166	244	177								
ONRC38	ONRC	-77.39611944		45.66363056		47	112	25	10859	259
-172	431	180	-88	180	-101	850	84	52	15	244
166	244	177								
ONRC39	ONRC	-77.39611944		45.66363056		47	112	25	10859	259
-172	431	180	-88	180	-101	850	84	52	15	244
166	244	177								
ONRC40	ONRC	-77.39611944		45.66363056		47	112	25	10859	259
-172	431	180	-88	180	-101	850	84	52	15	244
166	244	177								
ONRC41	ONRC	-77.39611944		45.66363056		47	112	25	10859	259
-172	431	180	-88	180	-101	850	84	52	15	244
166	244	177								
ONRC42	ONRC	-77.39611944		45.66363056		47	112	25	10859	259
-172	431	180	-88	180	-101	850	84	52	15	244
166	244	177								
ONRC43	ONRC	-77.39611944		45.66363056		47	112	25	10859	259
-172	431	180	-88	180	-101	850	84	52	15	244
166	244	177								
ONRC44	ONRC	-77.39611944		45.66363056		47	112	25	10859	259
-172	431	180	-88	180	-101	850	84	52	15	244
166	244	177								
ONRC45	ONRC	-77.39611944		45.66363056		47	112	25	10859	259
-172	431	180	-88	180	-101	850	84	52	15	244
166	244	177								
ONRC46	ONRC	-77.39611944		45.66363056		47	112	25	10859	259
-172	431	180	-88	180	-101	850	84	52	15	244
166	244	177								
ONRC47	ONRC	-77.39611944		45.66363056		47	112	25	10859	259
-172	431	180	-88	180	-101	850	84	52	15	244
166	244	177								
ONRC48	ONRC	-77.39611944		45.66363056		47	112	25	10859	259
-172	431	180	-88	180	-101	850	84	52	15	244
166	244	177								
ONRC49	ONRC	-77.39611944		45.66363056		47	112	25	10859	259
-172	431	180	-88	180	-101	850	84	52	15	244
166	244	177								
ONRC50	ONRC	-77.39611944		45.66363056		47	112	25	10859	259
-172	431	180	-88	180	-101	850	84	52	15	244

166 244 177
ONWL01 ONWL -80.65286389 46.84320833 33 112 25 11259 247
-199 446 158 -107 170 -120 845 99 49 19 259
164 243 170
ONWL02 ONWL -80.65286389 46.84320833 33 112 25 11259 247
-199 446 158 -107 170 -120 845 99 49 19 259
164 243 170
ONWL03 ONWL -80.65286389 46.84320833 33 112 25 11259 247
-199 446 158 -107 170 -120 845 99 49 19 259
164 243 170
ONWL04 ONWL -80.65286389 46.84320833 33 112 25 11259 247
-199 446 158 -107 170 -120 845 99 49 19 259
164 243 170
ONWL05 ONWL -80.65286389 46.84320833 33 112 25 11259 247
-199 446 158 -107 170 -120 845 99 49 19 259
164 243 170
ONWL06 ONWL -80.65286389 46.84320833 33 112 25 11259 247
-199 446 158 -107 170 -120 845 99 49 19 259
164 243 170
ONWL07 ONWL -80.65286389 46.84320833 33 112 25 11259 247
-199 446 158 -107 170 -120 845 99 49 19 259
164 243 170
ONWL08 ONWL -80.65286389 46.84320833 33 112 25 11259 247
-199 446 158 -107 170 -120 845 99 49 19 259
164 243 170
ONWL09 ONWL -80.65286389 46.84320833 33 112 25 11259 247
-199 446 158 -107 170 -120 845 99 49 19 259
164 243 170
ONWL10 ONWL -80.65286389 46.84320833 33 112 25 11259 247
-199 446 158 -107 170 -120 845 99 49 19 259
164 243 170
ONWL11 ONWL -80.65286389 46.84320833 33 112 25 11259 247
-199 446 158 -107 170 -120 845 99 49 19 259
164 243 170
ONWL12 ONWL -80.65286389 46.84320833 33 112 25 11259 247
-199 446 158 -107 170 -120 845 99 49 19 259
164 243 170
ONWL13 ONWL -80.65286389 46.84320833 33 112 25 11259 247
-199 446 158 -107 170 -120 845 99 49 19 259
164 243 170
ONWL14 ONWL -80.65286389 46.84320833 33 112 25 11259 247
-199 446 158 -107 170 -120 845 99 49 19 259
164 243 170
ONWL15 ONWL -80.65286389 46.84320833 33 112 25 11259 247
-199 446 158 -107 170 -120 845 99 49 19 259
164 243 170
ONWL16 ONWL -80.65286389 46.84320833 33 112 25 11259 247
-199 446 158 -107 170 -120 845 99 49 19 259
164 243 170
ONWL17 ONWL -80.65286389 46.84320833 33 112 25 11259 247
-199 446 158 -107 170 -120 845 99 49 19 259
164 243 170
ONWL18 ONWL -80.65286389 46.84320833 33 112 25 11259 247
-199 446 158 -107 170 -120 845 99 49 19 259
164 243 170
ONWL19 ONWL -80.65286389 46.84320833 33 112 25 11259 247
-199 446 158 -107 170 -120 845 99 49 19 259
164 243 170
ONWL20 ONWL -80.65286389 46.84320833 33 112 25 11259 247
-199 446 158 -107 170 -120 845 99 49 19 259
164 243 170
ONWL21 ONWL -80.65286389 46.84320833 33 112 25 11259 247
-199 446 158 -107 170 -120 845 99 49 19 259

164	243	170								
ONWL22	ONWL	-80.65286389		46.84320833		33	112	25	11259	247
-199	446	158	-107	170	-120	845	99	49	19	259
164	243	170								
ONWL23	ONWL	-80.65286389		46.84320833		33	112	25	11259	247
-199	446	158	-107	170	-120	845	99	49	19	259
164	243	170								
ONWL24	ONWL	-80.65286389		46.84320833		33	112	25	11259	247
-199	446	158	-107	170	-120	845	99	49	19	259
164	243	170								
ONWL25	ONWL	-80.65286389		46.84320833		33	112	25	11259	247
-199	446	158	-107	170	-120	845	99	49	19	259
164	243	170								
ONWL26	ONWL	-80.65286389		46.84320833		33	112	25	11259	247
-199	446	158	-107	170	-120	845	99	49	19	259
164	243	170								
ONWL27	ONWL	-80.65286389		46.84320833		33	112	25	11259	247
-199	446	158	-107	170	-120	845	99	49	19	259
164	243	170								
ONWL28	ONWL	-80.65286389		46.84320833		33	112	25	11259	247
-199	446	158	-107	170	-120	845	99	49	19	259
164	243	170								
ONWL29	ONWL	-80.65286389		46.84320833		33	112	25	11259	247
-199	446	158	-107	170	-120	845	99	49	19	259
164	243	170								
ONWL30	ONWL	-80.65286389		46.84320833		33	112	25	11259	247
-199	446	158	-107	170	-120	845	99	49	19	259
164	243	170								
ONWL31	ONWL	-80.65286389		46.84320833		33	112	25	11259	247
-199	446	158	-107	170	-120	845	99	49	19	259
164	243	170								
ONWL32	ONWL	-80.65286389		46.84320833		33	112	25	11259	247
-199	446	158	-107	170	-120	845	99	49	19	259
164	243	170								
ONWL33	ONWL	-80.65286389		46.84320833		33	112	25	11259	247
-199	446	158	-107	170	-120	845	99	49	19	259
164	243	170								
ONWL34	ONWL	-80.65286389		46.84320833		33	112	25	11259	247
-199	446	158	-107	170	-120	845	99	49	19	259
164	243	170								
ONWL35	ONWL	-80.65286389		46.84320833		33	112	25	11259	247
-199	446	158	-107	170	-120	845	99	49	19	259
164	243	170								
ONWL36	ONWL	-80.65286389		46.84320833		33	112	25	11259	247
-199	446	158	-107	170	-120	845	99	49	19	259
164	243	170								
ONWL37	ONWL	-80.65286389		46.84320833		33	112	25	11259	247
-199	446	158	-107	170	-120	845	99	49	19	259
164	243	170								
ONWL38	ONWL	-80.65286389		46.84320833		33	112	25	11259	247
-199	446	158	-107	170	-120	845	99	49	19	259
164	243	170								
ONWL39	ONWL	-80.65286389		46.84320833		33	112	25	11259	247
-199	446	158	-107	170	-120	845	99	49	19	259
164	243	170								
ONWL40	ONWL	-80.65286389		46.84320833		33	112	25	11259	247
-199	446	158	-107	170	-120	845	99	49	19	259
164	243	170								
ONWL41	ONWL	-80.65286389		46.84320833		33	112	25	11259	247
-199	446	158	-107	170	-120	845	99	49	19	259
164	243	170								
ONWL42	ONWL	-80.65286389		46.84320833		33	112	25	11259	247
-199	446	158	-107	170	-120	845	99	49	19	259

164	243	170								
ONWL43	ONWL	-80.65286389		46.84320833		33	112	25	11259	247
-199	446	158	-107	170	-120	845	99	49	19	259
164	243	170								
ONWL44	ONWL	-80.65286389		46.84320833		33	112	25	11259	247
-199	446	158	-107	170	-120	845	99	49	19	259
164	243	170								
ONWL45	ONWL	-80.65286389		46.84320833		33	112	25	11259	247
-199	446	158	-107	170	-120	845	99	49	19	259
164	243	170								
ONWL46	ONWL	-80.65286389		46.84320833		33	112	25	11259	247
-199	446	158	-107	170	-120	845	99	49	19	259
164	243	170								
ONWL47	ONWL	-80.65286389		46.84320833		33	112	25	11259	247
-199	446	158	-107	170	-120	845	99	49	19	259
164	243	170								
ONWL48	ONWL	-80.65286389		46.84320833		33	112	25	11259	247
-199	446	158	-107	170	-120	845	99	49	19	259
164	243	170								
ONWL49	ONWL	-80.65286389		46.84320833		33	112	25	11259	247
-199	446	158	-107	170	-120	845	99	49	19	259
164	243	170								
ONWL50	ONWL	-80.65286389		46.84320833		33	112	25	11259	247
-199	446	158	-107	170	-120	845	99	49	19	259
164	243	170								
ONCL01	ONCL	-94.27326944		49.08432222		24	109	22	12669	250
-230	480	177	-151	177	-151	641	101	18	51	277
68	277	68								
ONCL02	ONCL	-94.27326944		49.08432222		24	109	22	12669	250
-230	480	177	-151	177	-151	641	101	18	51	277
68	277	68								
ONCL03	ONCL	-94.27326944		49.08432222		24	109	22	12669	250
-230	480	177	-151	177	-151	641	101	18	51	277
68	277	68								
ONCL04	ONCL	-94.27326944		49.08432222		24	109	22	12669	250
-230	480	177	-151	177	-151	641	101	18	51	277
68	277	68								
ONCL05	ONCL	-94.27326944		49.08432222		24	109	22	12669	250
-230	480	177	-151	177	-151	641	101	18	51	277
68	277	68								
ONCL06	ONCL	-94.27326944		49.08432222		24	109	22	12669	250
-230	480	177	-151	177	-151	641	101	18	51	277
68	277	68								
ONCL07	ONCL	-94.27326944		49.08432222		24	109	22	12669	250
-230	480	177	-151	177	-151	641	101	18	51	277
68	277	68								
ONCL08	ONCL	-94.27326944		49.08432222		24	109	22	12669	250
-230	480	177	-151	177	-151	641	101	18	51	277
68	277	68								
ONCL09	ONCL	-94.27326944		49.08432222		24	109	22	12669	250
-230	480	177	-151	177	-151	641	101	18	51	277
68	277	68								
ONCL10	ONCL	-94.27326944		49.08432222		24	109	22	12669	250
-230	480	177	-151	177	-151	641	101	18	51	277
68	277	68								
ONCL11	ONCL	-94.27326944		49.08432222		24	109	22	12669	250
-230	480	177	-151	177	-151	641	101	18	51	277
68	277	68								
ONCL12	ONCL	-94.27326944		49.08432222		24	109	22	12669	250
-230	480	177	-151	177	-151	641	101	18	51	277
68	277	68								
ONCL13	ONCL	-94.27326944		49.08432222		24	109	22	12669	250
-230	480	177	-151	177	-151	641	101	18	51	277

68	277	68								
ONCL14	ONCL	-94.27326944		49.08432222		24	109	22	12669	250
-230	480	177	-151	177	-151	641	101	18	51	277
68	277	68								
ONCL15	ONCL	-94.27326944		49.08432222		24	109	22	12669	250
-230	480	177	-151	177	-151	641	101	18	51	277
68	277	68								
ONCL16	ONCL	-94.27326944		49.08432222		24	109	22	12669	250
-230	480	177	-151	177	-151	641	101	18	51	277
68	277	68								
ONCL17	ONCL	-94.27326944		49.08432222		24	109	22	12669	250
-230	480	177	-151	177	-151	641	101	18	51	277
68	277	68								
ONCL18	ONCL	-94.27326944		49.08432222		24	109	22	12669	250
-230	480	177	-151	177	-151	641	101	18	51	277
68	277	68								
ONCL19	ONCL	-94.27326944		49.08432222		24	109	22	12669	250
-230	480	177	-151	177	-151	641	101	18	51	277
68	277	68								
ONCL20	ONCL	-94.27326944		49.08432222		24	109	22	12669	250
-230	480	177	-151	177	-151	641	101	18	51	277
68	277	68								
ONCL21	ONCL	-94.27326944		49.08432222		24	109	22	12669	250
-230	480	177	-151	177	-151	641	101	18	51	277
68	277	68								
ONCL22	ONCL	-94.27326944		49.08432222		24	109	22	12669	250
-230	480	177	-151	177	-151	641	101	18	51	277
68	277	68								
ONCL23	ONCL	-94.27326944		49.08432222		24	109	22	12669	250
-230	480	177	-151	177	-151	641	101	18	51	277
68	277	68								
ONCL24	ONCL	-94.27326944		49.08432222		24	109	22	12669	250
-230	480	177	-151	177	-151	641	101	18	51	277
68	277	68								
ONCL25	ONCL	-94.27326944		49.08432222		24	109	22	12669	250
-230	480	177	-151	177	-151	641	101	18	51	277
68	277	68								
ONCL26	ONCL	-94.27326944		49.08432222		24	109	22	12669	250
-230	480	177	-151	177	-151	641	101	18	51	277
68	277	68								
ONCL27	ONCL	-94.27326944		49.08432222		24	109	22	12669	250
-230	480	177	-151	177	-151	641	101	18	51	277
68	277	68								
ONCL28	ONCL	-94.27326944		49.08432222		24	109	22	12669	250
-230	480	177	-151	177	-151	641	101	18	51	277
68	277	68								
ONCL29	ONCL	-94.27326944		49.08432222		24	109	22	12669	250
-230	480	177	-151	177	-151	641	101	18	51	277
68	277	68								
ONCL30	ONCL	-94.27326944		49.08432222		24	109	22	12669	250
-230	480	177	-151	177	-151	641	101	18	51	277
68	277	68								
ONCL31	ONCL	-94.27326944		49.08432222		24	109	22	12669	250
-230	480	177	-151	177	-151	641	101	18	51	277
68	277	68								
ONCL32	ONCL	-94.27326944		49.08432222		24	109	22	12669	250
-230	480	177	-151	177	-151	641	101	18	51	277
68	277	68								
ONCL33	ONCL	-94.27326944		49.08432222		24	109	22	12669	250
-230	480	177	-151	177	-151	641	101	18	51	277
68	277	68								
ONCL34	ONCL	-94.27326944		49.08432222		24	109	22	12669	250
-230	480	177	-151	177	-151	641	101	18	51	277

68	277	68								
ONCL35	ONCL	-94.27326944		49.08432222		24	109	22	12669	250
-230	480	177	-151	177	-151	641	101	18	51	277
68	277	68								
ONCL36	ONCL	-94.27326944		49.08432222		24	109	22	12669	250
-230	480	177	-151	177	-151	641	101	18	51	277
68	277	68								
ONCL37	ONCL	-94.27326944		49.08432222		24	109	22	12669	250
-230	480	177	-151	177	-151	641	101	18	51	277
68	277	68								
ONCL38	ONCL	-94.27326944		49.08432222		24	109	22	12669	250
-230	480	177	-151	177	-151	641	101	18	51	277
68	277	68								
ONCL39	ONCL	-94.27326944		49.08432222		24	109	22	12669	250
-230	480	177	-151	177	-151	641	101	18	51	277
68	277	68								
ONCL40	ONCL	-94.27326944		49.08432222		24	109	22	12669	250
-230	480	177	-151	177	-151	641	101	18	51	277
68	277	68								
ONCL41	ONCL	-94.27326944		49.08432222		24	109	22	12669	250
-230	480	177	-151	177	-151	641	101	18	51	277
68	277	68								
ONCL42	ONCL	-94.27326944		49.08432222		24	109	22	12669	250
-230	480	177	-151	177	-151	641	101	18	51	277
68	277	68								
ONCL43	ONCL	-94.27326944		49.08432222		24	109	22	12669	250
-230	480	177	-151	177	-151	641	101	18	51	277
68	277	68								
ONCL44	ONCL	-94.27326944		49.08432222		24	109	22	12669	250
-230	480	177	-151	177	-151	641	101	18	51	277
68	277	68								
ONCL45	ONCL	-94.27326944		49.08432222		24	109	22	12669	250
-230	480	177	-151	177	-151	641	101	18	51	277
68	277	68								
ONCL46	ONCL	-94.27326944		49.08432222		24	109	22	12669	250
-230	480	177	-151	177	-151	641	101	18	51	277
68	277	68								
ONCL47	ONCL	-94.27326944		49.08432222		24	109	22	12669	250
-230	480	177	-151	177	-151	641	101	18	51	277
68	277	68								
ONCL48	ONCL	-94.27326944		49.08432222		24	109	22	12669	250
-230	480	177	-151	177	-151	641	101	18	51	277
68	277	68								
ONCL49	ONCL	-94.27326944		49.08432222		24	109	22	12669	250
-230	480	177	-151	177	-151	641	101	18	51	277
68	277	68								
ONCL50	ONCL	-94.27326944		49.08432222		24	109	22	12669	250
-230	480	177	-151	177	-151	641	101	18	51	277
68	277	68								
ONTO01	ONTO	-79.47838333		47.13071667		33	109	24	11425	245
-200	445	160	-53	172	-123	866	97	48	22	280
158	271	171								
ONTO02	ONTO	-79.47838333		47.13071667		33	109	24	11425	245
-200	445	160	-53	172	-123	866	97	48	22	280
158	271	171								
ONTO03	ONTO	-79.47838333		47.13071667		33	109	24	11425	245
-200	445	160	-53	172	-123	866	97	48	22	280
158	271	171								
ONTO04	ONTO	-79.47838333		47.13071667		33	109	24	11425	245
-200	445	160	-53	172	-123	866	97	48	22	280
158	271	171								
ONTO05	ONTO	-79.47838333		47.13071667		33	109	24	11425	245
-200	445	160	-53	172	-123	866	97	48	22	280

```
158     271     171
ONTO06  ONTO    -79.47838333    47.13071667     33      109     24      11425   245
-200    445     160     -53     172     -123    866     97      48      22      280
158     271     171
ONTO07  ONTO    -79.47838333    47.13071667     33      109     24      11425   245
-200    445     160     -53     172     -123    866     97      48      22      280
158     271     171
ONTO08  ONTO    -79.47838333    47.13071667     33      109     24      11425   245
-200    445     160     -53     172     -123    866     97      48      22      280
158     271     171
ONTO09  ONTO    -79.47838333    47.13071667     33      109     24      11425   245
-200    445     160     -53     172     -123    866     97      48      22      280
158     271     171
ONTO10  ONTO    -79.47838333    47.13071667     33      109     24      11425   245
-200    445     160     -53     172     -123    866     97      48      22      280
158     271     171
ONTO11  ONTO    -79.47838333    47.13071667     33      109     24      11425   245
-200    445     160     -53     172     -123    866     97      48      22      280
158     271     171
ONTO12  ONTO    -79.47838333    47.13071667     33      109     24      11425   245
-200    445     160     -53     172     -123    866     97      48      22      280
158     271     171
ONTO13  ONTO    -79.47838333    47.13071667     33      109     24      11425   245
-200    445     160     -53     172     -123    866     97      48      22      280
158     271     171
ONTO14  ONTO    -79.47838333    47.13071667     33      109     24      11425   245
-200    445     160     -53     172     -123    866     97      48      22      280
158     271     171
ONTO15  ONTO    -79.47838333    47.13071667     33      109     24      11425   245
-200    445     160     -53     172     -123    866     97      48      22      280
158     271     171
ONTO16  ONTO    -79.47838333    47.13071667     33      109     24      11425   245
-200    445     160     -53     172     -123    866     97      48      22      280
158     271     171
ONTO17  ONTO    -79.47838333    47.13071667     33      109     24      11425   245
-200    445     160     -53     172     -123    866     97      48      22      280
158     271     171
ONTO18  ONTO    -79.47838333    47.13071667     33      109     24      11425   245
-200    445     160     -53     172     -123    866     97      48      22      280
158     271     171
ONTO19  ONTO    -79.47838333    47.13071667     33      109     24      11425   245
-200    445     160     -53     172     -123    866     97      48      22      280
158     271     171
ONTO20  ONTO    -79.47838333    47.13071667     33      109     24      11425   245
-200    445     160     -53     172     -123    866     97      48      22      280
158     271     171
ONTO21  ONTO    -79.47838333    47.13071667     33      109     24      11425   245
-200    445     160     -53     172     -123    866     97      48      22      280
158     271     171
ONTO22  ONTO    -79.47838333    47.13071667     33      109     24      11425   245
-200    445     160     -53     172     -123    866     97      48      22      280
158     271     171
ONTO23  ONTO    -79.47838333    47.13071667     33      109     24      11425   245
-200    445     160     -53     172     -123    866     97      48      22      280
158     271     171
ONTO24  ONTO    -79.47838333    47.13071667     33      109     24      11425   245
-200    445     160     -53     172     -123    866     97      48      22      280
158     271     171
ONTO25  ONTO    -79.47838333    47.13071667     33      109     24      11425   245
-200    445     160     -53     172     -123    866     97      48      22      280
158     271     171
ONTO26  ONTO    -79.47838333    47.13071667     33      109     24      11425   245
-200    445     160     -53     172     -123    866     97      48      22      280
```

```
158	271	171
ONTO27	ONTO	-79.47838333	47.13071667	33	109	24	11425	245
-200	445	160	-53	172	-123	866	97	48	22	280
158	271	171
ONTO28	ONTO	-79.47838333	47.13071667	33	109	24	11425	245
-200	445	160	-53	172	-123	866	97	48	22	280
158	271	171
ONTO29	ONTO	-79.47838333	47.13071667	33	109	24	11425	245
-200	445	160	-53	172	-123	866	97	48	22	280
158	271	171
ONTO30	ONTO	-79.47838333	47.13071667	33	109	24	11425	245
-200	445	160	-53	172	-123	866	97	48	22	280
158	271	171
ONTO31	ONTO	-79.47838333	47.13071667	33	109	24	11425	245
-200	445	160	-53	172	-123	866	97	48	22	280
158	271	171
ONTO32	ONTO	-79.47838333	47.13071667	33	109	24	11425	245
-200	445	160	-53	172	-123	866	97	48	22	280
158	271	171
ONTO33	ONTO	-79.47838333	47.13071667	33	109	24	11425	245
-200	445	160	-53	172	-123	866	97	48	22	280
158	271	171
ONTO34	ONTO	-79.47838333	47.13071667	33	109	24	11425	245
-200	445	160	-53	172	-123	866	97	48	22	280
158	271	171
ONTO35	ONTO	-79.47838333	47.13071667	33	109	24	11425	245
-200	445	160	-53	172	-123	866	97	48	22	280
158	271	171
ONTO36	ONTO	-79.47838333	47.13071667	33	109	24	11425	245
-200	445	160	-53	172	-123	866	97	48	22	280
158	271	171
ONTO37	ONTO	-79.47838333	47.13071667	33	109	24	11425	245
-200	445	160	-53	172	-123	866	97	48	22	280
158	271	171
ONTO38	ONTO	-79.47838333	47.13071667	33	109	24	11425	245
-200	445	160	-53	172	-123	866	97	48	22	280
158	271	171
ONTO39	ONTO	-79.47838333	47.13071667	33	109	24	11425	245
-200	445	160	-53	172	-123	866	97	48	22	280
158	271	171
ONTO40	ONTO	-79.47838333	47.13071667	33	109	24	11425	245
-200	445	160	-53	172	-123	866	97	48	22	280
158	271	171
ONTO41	ONTO	-79.47838333	47.13071667	33	109	24	11425	245
-200	445	160	-53	172	-123	866	97	48	22	280
158	271	171
ONTO42	ONTO	-79.47838333	47.13071667	33	109	24	11425	245
-200	445	160	-53	172	-123	866	97	48	22	280
158	271	171
ONTO43	ONTO	-79.47838333	47.13071667	33	109	24	11425	245
-200	445	160	-53	172	-123	866	97	48	22	280
158	271	171
ONTO44	ONTO	-79.47838333	47.13071667	33	109	24	11425	245
-200	445	160	-53	172	-123	866	97	48	22	280
158	271	171
ONTO45	ONTO	-79.47838333	47.13071667	33	109	24	11425	245
-200	445	160	-53	172	-123	866	97	48	22	280
158	271	171
ONTO46	ONTO	-79.47838333	47.13071667	33	109	24	11425	245
-200	445	160	-53	172	-123	866	97	48	22	280
158	271	171
ONTO47	ONTO	-79.47838333	47.13071667	33	109	24	11425	245
-200	445	160	-53	172	-123	866	97	48	22	280
```

158	271	171								
ONTO48	ONTO	-79.47838333		47.13071667		33	109	24	11425	245
-200	445	160	-53	172	-123	866	97	48	22	280
158	271	171								
ONTO49	ONTO	-79.47838333		47.13071667		33	109	24	11425	245
-200	445	160	-53	172	-123	866	97	48	22	280
158	271	171								
ONTO50	ONTO	-79.47838333		47.13071667		33	109	24	11425	245
-200	445	160	-53	172	-123	866	97	48	22	280
158	271	171								
MEEB01	MEEB	-69.16459444		44.79020278		61	120	29	9904	262
-148	410	14	-59	184	-73	1099	116	75	11	316
239	267	258								
MEEB02	MEEB	-69.16459444		44.79020278		61	120	29	9904	262
-148	410	14	-59	184	-73	1099	116	75	11	316
239	267	258								
MEEB03	MEEB	-69.16459444		44.79020278		61	120	29	9904	262
-148	410	14	-59	184	-73	1099	116	75	11	316
239	267	258								
MEEB04	MEEB	-69.16459444		44.79020278		61	120	29	9904	262
-148	410	14	-59	184	-73	1099	116	75	11	316
239	267	258								
MEEB05	MEEB	-69.16459444		44.79020278		61	120	29	9904	262
-148	410	14	-59	184	-73	1099	116	75	11	316
239	267	258								
MEEB06	MEEB	-69.16459444		44.79020278		61	120	29	9904	262
-148	410	14	-59	184	-73	1099	116	75	11	316
239	267	258								
MEEB07	MEEB	-69.16459444		44.79020278		61	120	29	9904	262
-148	410	14	-59	184	-73	1099	116	75	11	316
239	267	258								
MEEB08	MEEB	-69.16459444		44.79020278		61	120	29	9904	262
-148	410	14	-59	184	-73	1099	116	75	11	316
239	267	258								
MEEB09	MEEB	-69.16459444		44.79020278		61	120	29	9904	262
-148	410	14	-59	184	-73	1099	116	75	11	316
239	267	258								
MEEB10	MEEB	-69.16459444		44.79020278		61	120	29	9904	262
-148	410	14	-59	184	-73	1099	116	75	11	316
239	267	258								
MEEB11	MEEB	-69.16459444		44.79020278		61	120	29	9904	262
-148	410	14	-59	184	-73	1099	116	75	11	316
239	267	258								
MEEB12	MEEB	-69.16459444		44.79020278		61	120	29	9904	262
-148	410	14	-59	184	-73	1099	116	75	11	316
239	267	258								
MEEB13	MEEB	-69.16459444		44.79020278		61	120	29	9904	262
-148	410	14	-59	184	-73	1099	116	75	11	316
239	267	258								
MEEB14	MEEB	-69.16459444		44.79020278		61	120	29	9904	262
-148	410	14	-59	184	-73	1099	116	75	11	316
239	267	258								
MEEB15	MEEB	-69.16459444		44.79020278		61	120	29	9904	262
-148	410	14	-59	184	-73	1099	116	75	11	316
239	267	258								
MEEB16	MEEB	-69.16459444		44.79020278		61	120	29	9904	262
-148	410	14	-59	184	-73	1099	116	75	11	316
239	267	258								
MEEB17	MEEB	-69.16459444		44.79020278		61	120	29	9904	262
-148	410	14	-59	184	-73	1099	116	75	11	316
239	267	258								
MEEB18	MEEB	-69.16459444		44.79020278		61	120	29	9904	262
-148	410	14	-59	184	-73	1099	116	75	11	316

239	267	258								
MEEB19	MEEB	-69.16459444	44.79020278	61	120	29	9904	262		
-148	410	14	-59	184	-73	1099	116	75	11	316
239	267	258								
MEEB20	MEEB	-69.16459444	44.79020278	61	120	29	9904	262		
-148	410	14	-59	184	-73	1099	116	75	11	316
239	267	258								
MEEB21	MEEB	-69.16459444	44.79020278	61	120	29	9904	262		
-148	410	14	-59	184	-73	1099	116	75	11	316
239	267	258								
MEEB22	MEEB	-69.16459444	44.79020278	61	120	29	9904	262		
-148	410	14	-59	184	-73	1099	116	75	11	316
239	267	258								
MEEB23	MEEB	-69.16459444	44.79020278	61	120	29	9904	262		
-148	410	14	-59	184	-73	1099	116	75	11	316
239	267	258								
MEEB24	MEEB	-69.16459444	44.79020278	61	120	29	9904	262		
-148	410	14	-59	184	-73	1099	116	75	11	316
239	267	258								
MEEB25	MEEB	-69.16459444	44.79020278	61	120	29	9904	262		
-148	410	14	-59	184	-73	1099	116	75	11	316
239	267	258								
MEEB26	MEEB	-69.16459444	44.79020278	61	120	29	9904	262		
-148	410	14	-59	184	-73	1099	116	75	11	316
239	267	258								
MEEB27	MEEB	-69.16459444	44.79020278	61	120	29	9904	262		
-148	410	14	-59	184	-73	1099	116	75	11	316
239	267	258								
MEEB28	MEEB	-69.16459444	44.79020278	61	120	29	9904	262		
-148	410	14	-59	184	-73	1099	116	75	11	316
239	267	258								
MEEB29	MEEB	-69.16459444	44.79020278	61	120	29	9904	262		
-148	410	14	-59	184	-73	1099	116	75	11	316
239	267	258								
MEEB30	MEEB	-69.16459444	44.79020278	61	120	29	9904	262		
-148	410	14	-59	184	-73	1099	116	75	11	316
239	267	258								
MEEB31	MEEB	-69.16459444	44.79020278	61	120	29	9904	262		
-148	410	14	-59	184	-73	1099	116	75	11	316
239	267	258								
MEEB32	MEEB	-69.16459444	44.79020278	61	120	29	9904	262		
-148	410	14	-59	184	-73	1099	116	75	11	316
239	267	258								
MEEB33	MEEB	-69.16459444	44.79020278	61	120	29	9904	262		
-148	410	14	-59	184	-73	1099	116	75	11	316
239	267	258								
MEEB34	MEEB	-69.16459444	44.79020278	61	120	29	9904	262		
-148	410	14	-59	184	-73	1099	116	75	11	316
239	267	258								
MEEB35	MEEB	-69.16459444	44.79020278	61	120	29	9904	262		
-148	410	14	-59	184	-73	1099	116	75	11	316
239	267	258								
MEEB36	MEEB	-69.16459444	44.79020278	61	120	29	9904	262		
-148	410	14	-59	184	-73	1099	116	75	11	316
239	267	258								
MEEB37	MEEB	-69.16459444	44.79020278	61	120	29	9904	262		
-148	410	14	-59	184	-73	1099	116	75	11	316
239	267	258								
MEEB38	MEEB	-69.16459444	44.79020278	61	120	29	9904	262		
-148	410	14	-59	184	-73	1099	116	75	11	316
239	267	258								
MEEB39	MEEB	-69.16459444	44.79020278	61	120	29	9904	262		
-148	410	14	-59	184	-73	1099	116	75	11	316

239	267	258								
MEEB40	MEEB	-69.16459444		44.79020278		61	120	29	9904	262
-148	410	14	-59	184	-73	1099	116	75	11	316
239	267	258								
MEEB41	MEEB	-69.16459444		44.79020278		61	120	29	9904	262
-148	410	14	-59	184	-73	1099	116	75	11	316
239	267	258								
MEEB42	MEEB	-69.16459444		44.79020278		61	120	29	9904	262
-148	410	14	-59	184	-73	1099	116	75	11	316
239	267	258								
MEEB43	MEEB	-69.16459444		44.79020278		61	120	29	9904	262
-148	410	14	-59	184	-73	1099	116	75	11	316
239	267	258								
MEEB44	MEEB	-69.16459444		44.79020278		61	120	29	9904	262
-148	410	14	-59	184	-73	1099	116	75	11	316
239	267	258								
MEEB45	MEEB	-69.16459444		44.79020278		61	120	29	9904	262
-148	410	14	-59	184	-73	1099	116	75	11	316
239	267	258								
MEEB46	MEEB	-69.16459444		44.79020278		61	120	29	9904	262
-148	410	14	-59	184	-73	1099	116	75	11	316
239	267	258								
MEEB47	MEEB	-69.16459444		44.79020278		61	120	29	9904	262
-148	410	14	-59	184	-73	1099	116	75	11	316
239	267	258								
MEEB48	MEEB	-69.16459444		44.79020278		61	120	29	9904	262
-148	410	14	-59	184	-73	1099	116	75	11	316
239	267	258								
MEEB49	MEEB	-69.16459444		44.79020278		61	120	29	9904	262
-148	410	14	-59	184	-73	1099	116	75	11	316
239	267	258								
MEEB50	MEEB	-69.16459444		44.79020278		61	120	29	9904	262
-148	410	14	-59	184	-73	1099	116	75	11	316
239	267	258								
MEBSP01	MEBSP	-68.63551389		45.67254167		50	123	28	10256	260
-165	425	3	-75	179	-88	1022	113	70	14	292
211	278	232								
MEBSP02	MEBSP	-68.63551389		45.67254167		50	123	28	10256	260
-165	425	3	-75	179	-88	1022	113	70	14	292
211	278	232								
MEBSP03	MEBSP	-68.63551389		45.67254167		50	123	28	10256	260
-165	425	3	-75	179	-88	1022	113	70	14	292
211	278	232								
MEBSP04	MEBSP	-68.63551389		45.67254167		50	123	28	10256	260
-165	425	3	-75	179	-88	1022	113	70	14	292
211	278	232								
MEBSP05	MEBSP	-68.63551389		45.67254167		50	123	28	10256	260
-165	425	3	-75	179	-88	1022	113	70	14	292
211	278	232								
MEBSP06	MEBSP	-68.63551389		45.67254167		50	123	28	10256	260
-165	425	3	-75	179	-88	1022	113	70	14	292
211	278	232								
MEBSP07	MEBSP	-68.63551389		45.67254167		50	123	28	10256	260
-165	425	3	-75	179	-88	1022	113	70	14	292
211	278	232								
MEBSP08	MEBSP	-68.63551389		45.67254167		50	123	28	10256	260
-165	425	3	-75	179	-88	1022	113	70	14	292
211	278	232								
MEBSP09	MEBSP	-68.63551389		45.67254167		50	123	28	10256	260
-165	425	3	-75	179	-88	1022	113	70	14	292
211	278	232								
MEBSP10	MEBSP	-68.63551389		45.67254167		50	123	28	10256	260
-165	425	3	-75	179	-88	1022	113	70	14	292

211	278	232								
MEBSP11	MEBSP	-68.63551389		45.67254167		50	123	28	10256	260
-165	425	3	-75	179	-88	1022	113	70	14	292
211	278	232								
MEBSP12	MEBSP	-68.63551389		45.67254167		50	123	28	10256	260
-165	425	3	-75	179	-88	1022	113	70	14	292
211	278	232								
MEBSP13	MEBSP	-68.63551389		45.67254167		50	123	28	10256	260
-165	425	3	-75	179	-88	1022	113	70	14	292
211	278	232								
MEBSP14	MEBSP	-68.63551389		45.67254167		50	123	28	10256	260
-165	425	3	-75	179	-88	1022	113	70	14	292
211	278	232								
MEBSP15	MEBSP	-68.63551389		45.67254167		50	123	28	10256	260
-165	425	3	-75	179	-88	1022	113	70	14	292
211	278	232								
MEBSP16	MEBSP	-68.63551389		45.67254167		50	123	28	10256	260
-165	425	3	-75	179	-88	1022	113	70	14	292
211	278	232								
MEBSP17	MEBSP	-68.63551389		45.67254167		50	123	28	10256	260
-165	425	3	-75	179	-88	1022	113	70	14	292
211	278	232								
MEBSP18	MEBSP	-68.63551389		45.67254167		50	123	28	10256	260
-165	425	3	-75	179	-88	1022	113	70	14	292
211	278	232								
MEBSP19	MEBSP	-68.63551389		45.67254167		50	123	28	10256	260
-165	425	3	-75	179	-88	1022	113	70	14	292
211	278	232								
MEBSP20	MEBSP	-68.63551389		45.67254167		50	123	28	10256	260
-165	425	3	-75	179	-88	1022	113	70	14	292
211	278	232								
MEBSP21	MEBSP	-68.63551389		45.67254167		50	123	28	10256	260
-165	425	3	-75	179	-88	1022	113	70	14	292
211	278	232								
MEBSP22	MEBSP	-68.63551389		45.67254167		50	123	28	10256	260
-165	425	3	-75	179	-88	1022	113	70	14	292
211	278	232								
MEBSP23	MEBSP	-68.63551389		45.67254167		50	123	28	10256	260
-165	425	3	-75	179	-88	1022	113	70	14	292
211	278	232								
MEBSP24	MEBSP	-68.63551389		45.67254167		50	123	28	10256	260
-165	425	3	-75	179	-88	1022	113	70	14	292
211	278	232								
MEBSP25	MEBSP	-68.63551389		45.67254167		50	123	28	10256	260
-165	425	3	-75	179	-88	1022	113	70	14	292
211	278	232								
MEBSP26	MEBSP	-68.63551389		45.67254167		50	123	28	10256	260
-165	425	3	-75	179	-88	1022	113	70	14	292
211	278	232								
MEBSP27	MEBSP	-68.63551389		45.67254167		50	123	28	10256	260
-165	425	3	-75	179	-88	1022	113	70	14	292
211	278	232								
MEBSP28	MEBSP	-68.63551389		45.67254167		50	123	28	10256	260
-165	425	3	-75	179	-88	1022	113	70	14	292
211	278	232								
MEBSP29	MEBSP	-68.63551389		45.67254167		50	123	28	10256	260
-165	425	3	-75	179	-88	1022	113	70	14	292
211	278	232								
MEBSP30	MEBSP	-68.63551389		45.67254167		50	123	28	10256	260
-165	425	3	-75	179	-88	1022	113	70	14	292
211	278	232								
MEBSP31	MEBSP	-68.63551389		45.67254167		50	123	28	10256	260
-165	425	3	-75	179	-88	1022	113	70	14	292

211	278	232								
MEBSP32	MEBSP	-68.63551389		45.67254167		50	123	28	10256	260
-165	425	3	-75	179	-88	1022	113	70	14	292
211	278	232								
MEBSP33	MEBSP	-68.63551389		45.67254167		50	123	28	10256	260
-165	425	3	-75	179	-88	1022	113	70	14	292
211	278	232								
MEBSP34	MEBSP	-68.63551389		45.67254167		50	123	28	10256	260
-165	425	3	-75	179	-88	1022	113	70	14	292
211	278	232								
MEBSP35	MEBSP	-68.63551389		45.67254167		50	123	28	10256	260
-165	425	3	-75	179	-88	1022	113	70	14	292
211	278	232								
MEBSP36	MEBSP	-68.63551389		45.67254167		50	123	28	10256	260
-165	425	3	-75	179	-88	1022	113	70	14	292
211	278	232								
MEBSP37	MEBSP	-68.63551389		45.67254167		50	123	28	10256	260
-165	425	3	-75	179	-88	1022	113	70	14	292
211	278	232								
MEBSP38	MEBSP	-68.63551389		45.67254167		50	123	28	10256	260
-165	425	3	-75	179	-88	1022	113	70	14	292
211	278	232								
MEBSP39	MEBSP	-68.63551389		45.67254167		50	123	28	10256	260
-165	425	3	-75	179	-88	1022	113	70	14	292
211	278	232								
MEBSP40	MEBSP	-68.63551389		45.67254167		50	123	28	10256	260
-165	425	3	-75	179	-88	1022	113	70	14	292
211	278	232								
MEBSP41	MEBSP	-68.63551389		45.67254167		50	123	28	10256	260
-165	425	3	-75	179	-88	1022	113	70	14	292
211	278	232								
MEBSP42	MEBSP	-68.63551389		45.67254167		50	123	28	10256	260
-165	425	3	-75	179	-88	1022	113	70	14	292
211	278	232								
MEBSP43	MEBSP	-68.63551389		45.67254167		50	123	28	10256	260
-165	425	3	-75	179	-88	1022	113	70	14	292
211	278	232								
MEBSP44	MEBSP	-68.63551389		45.67254167		50	123	28	10256	260
-165	425	3	-75	179	-88	1022	113	70	14	292
211	278	232								
MEBSP45	MEBSP	-68.63551389		45.67254167		50	123	28	10256	260
-165	425	3	-75	179	-88	1022	113	70	14	292
211	278	232								
MEBSP46	MEBSP	-68.63551389		45.67254167		50	123	28	10256	260
-165	425	3	-75	179	-88	1022	113	70	14	292
211	278	232								
MEBSP47	MEBSP	-68.63551389		45.67254167		50	123	28	10256	260
-165	425	3	-75	179	-88	1022	113	70	14	292
211	278	232								
MEBSP48	MEBSP	-68.63551389		45.67254167		50	123	28	10256	260
-165	425	3	-75	179	-88	1022	113	70	14	292
211	278	232								
MEBSP49	MEBSP	-68.63551389		45.67254167		50	123	28	10256	260
-165	425	3	-75	179	-88	1022	113	70	14	292
211	278	232								
MEBSP50	MEBSP	-68.63551389		45.67254167		50	123	28	10256	260
-165	425	3	-75	179	-88	1022	113	70	14	292
211	278	232								
MASB01	MASB	-73.28606667		42.26363333		66	114	30	9132	255
-119	374	160	-44	180	-56	1166	108	79	10	319
254	319	258								
MASB02	MASB	-73.28606667		42.26363333		66	114	30	9132	255
-119	374	160	-44	180	-56	1166	108	79	10	319

254 319 258
MASB03 MASB -73.28606667 42.26363333 66 114 30 9132 255
-119 374 160 -44 180 -56 1166 108 79 10 319
254 319 258
MASB04 MASB -73.28606667 42.26363333 66 114 30 9132 255
-119 374 160 -44 180 -56 1166 108 79 10 319
254 319 258
MASB05 MASB -73.28606667 42.26363333 66 114 30 9132 255
-119 374 160 -44 180 -56 1166 108 79 10 319
254 319 258
MASB06 MASB -73.28606667 42.26363333 66 114 30 9132 255
-119 374 160 -44 180 -56 1166 108 79 10 319
254 319 258
MASB07 MASB -73.28606667 42.26363333 66 114 30 9132 255
-119 374 160 -44 180 -56 1166 108 79 10 319
254 319 258
MASB08 MASB -73.28606667 42.26363333 66 114 30 9132 255
-119 374 160 -44 180 -56 1166 108 79 10 319
254 319 258
MASB09 MASB -73.28606667 42.26363333 66 114 30 9132 255
-119 374 160 -44 180 -56 1166 108 79 10 319
254 319 258
MASB10 MASB -73.28606667 42.26363333 66 114 30 9132 255
-119 374 160 -44 180 -56 1166 108 79 10 319
254 319 258
MASB11 MASB -73.28606667 42.26363333 66 114 30 9132 255
-119 374 160 -44 180 -56 1166 108 79 10 319
254 319 258
MASB12 MASB -73.28606667 42.26363333 66 114 30 9132 255
-119 374 160 -44 180 -56 1166 108 79 10 319
254 319 258
MASB13 MASB -73.28606667 42.26363333 66 114 30 9132 255
-119 374 160 -44 180 -56 1166 108 79 10 319
254 319 258
MASB14 MASB -73.28606667 42.26363333 66 114 30 9132 255
-119 374 160 -44 180 -56 1166 108 79 10 319
254 319 258
MASB15 MASB -73.28606667 42.26363333 66 114 30 9132 255
-119 374 160 -44 180 -56 1166 108 79 10 319
254 319 258
MASB16 MASB -73.28606667 42.26363333 66 114 30 9132 255
-119 374 160 -44 180 -56 1166 108 79 10 319
254 319 258
MASB17 MASB -73.28606667 42.26363333 66 114 30 9132 255
-119 374 160 -44 180 -56 1166 108 79 10 319
254 319 258
MASB18 MASB -73.28606667 42.26363333 66 114 30 9132 255
-119 374 160 -44 180 -56 1166 108 79 10 319
254 319 258
MASB19 MASB -73.28606667 42.26363333 66 114 30 9132 255
-119 374 160 -44 180 -56 1166 108 79 10 319
254 319 258
MASB20 MASB -73.28606667 42.26363333 66 114 30 9132 255
-119 374 160 -44 180 -56 1166 108 79 10 319
254 319 258
MASB21 MASB -73.28606667 42.26363333 66 114 30 9132 255
-119 374 160 -44 180 -56 1166 108 79 10 319
254 319 258
MASB22 MASB -73.28606667 42.26363333 66 114 30 9132 255
-119 374 160 -44 180 -56 1166 108 79 10 319
254 319 258
MASB23 MASB -73.28606667 42.26363333 66 114 30 9132 255
-119 374 160 -44 180 -56 1166 108 79 10 319

254	319	258
MASB24	MASB	-73.28606667	42.26363333	66	114	30	9132	255
-119	374	160	-44	180	-56	1166	108	79	10	319
254	319	258
MASB25	MASB	-73.28606667	42.26363333	66	114	30	9132	255
-119	374	160	-44	180	-56	1166	108	79	10	319
254	319	258
MASB26	MASB	-73.28606667	42.26363333	66	114	30	9132	255
-119	374	160	-44	180	-56	1166	108	79	10	319
254	319	258
MASB27	MASB	-73.28606667	42.26363333	66	114	30	9132	255
-119	374	160	-44	180	-56	1166	108	79	10	319
254	319	258
MASB28	MASB	-73.28606667	42.26363333	66	114	30	9132	255
-119	374	160	-44	180	-56	1166	108	79	10	319
254	319	258
MASB29	MASB	-73.28606667	42.26363333	66	114	30	9132	255
-119	374	160	-44	180	-56	1166	108	79	10	319
254	319	258
MASB30	MASB	-73.28606667	42.26363333	66	114	30	9132	255
-119	374	160	-44	180	-56	1166	108	79	10	319
254	319	258
MASB31	MASB	-73.28606667	42.26363333	66	114	30	9132	255
-119	374	160	-44	180	-56	1166	108	79	10	319
254	319	258
MASB32	MASB	-73.28606667	42.26363333	66	114	30	9132	255
-119	374	160	-44	180	-56	1166	108	79	10	319
254	319	258
MASB33	MASB	-73.28606667	42.26363333	66	114	30	9132	255
-119	374	160	-44	180	-56	1166	108	79	10	319
254	319	258
MASB34	MASB	-73.28606667	42.26363333	66	114	30	9132	255
-119	374	160	-44	180	-56	1166	108	79	10	319
254	319	258
MASB35	MASB	-73.28606667	42.26363333	66	114	30	9132	255
-119	374	160	-44	180	-56	1166	108	79	10	319
254	319	258
MASB36	MASB	-73.28606667	42.26363333	66	114	30	9132	255
-119	374	160	-44	180	-56	1166	108	79	10	319
254	319	258
MASB37	MASB	-73.28606667	42.26363333	66	114	30	9132	255
-119	374	160	-44	180	-56	1166	108	79	10	319
254	319	258
MASB38	MASB	-73.28606667	42.26363333	66	114	30	9132	255
-119	374	160	-44	180	-56	1166	108	79	10	319
254	319	258
MASB39	MASB	-73.28606667	42.26363333	66	114	30	9132	255
-119	374	160	-44	180	-56	1166	108	79	10	319
254	319	258
MASB40	MASB	-73.28606667	42.26363333	66	114	30	9132	255
-119	374	160	-44	180	-56	1166	108	79	10	319
254	319	258
MASB41	MASB	-73.28606667	42.26363333	66	114	30	9132	255
-119	374	160	-44	180	-56	1166	108	79	10	319
254	319	258
MASB42	MASB	-73.28606667	42.26363333	66	114	30	9132	255
-119	374	160	-44	180	-56	1166	108	79	10	319
254	319	258
MASB43	MASB	-73.28606667	42.26363333	66	114	30	9132	255
-119	374	160	-44	180	-56	1166	108	79	10	319
254	319	258
MASB44	MASB	-73.28606667	42.26363333	66	114	30	9132	255
-119	374	160	-44	180	-56	1166	108	79	10	319

254 319 258
MASB45 MASB -73.28606667 42.26363333 66 114 30 9132 255
-119 374 160 -44 180 -56 1166 108 79 10 319
254 319 258
MASB46 MASB -73.28606667 42.26363333 66 114 30 9132 255
-119 374 160 -44 180 -56 1166 108 79 10 319
254 319 258
MASB47 MASB -73.28606667 42.26363333 66 114 30 9132 255
-119 374 160 -44 180 -56 1166 108 79 10 319
254 319 258
MASB48 MASB -73.28606667 42.26363333 66 114 30 9132 255
-119 374 160 -44 180 -56 1166 108 79 10 319
254 319 258
MASB49 MASB -73.28606667 42.26363333 66 114 30 9132 255
-119 374 160 -44 180 -56 1166 108 79 10 319
254 319 258
MASB50 MASB -73.28606667 42.26363333 66 114 30 9132 255
-119 374 160 -44 180 -56 1166 108 79 10 319
254 319 258
NYCM01 NYCM -74.16803889 41.9465 87 117 30 9228 279
-100 379 182 -24 203 -36 1159 115 78 10 322
252 303 258
NYCM02 NYCM -74.16803889 41.9465 87 117 30 9228 279
-100 379 182 -24 203 -36 1159 115 78 10 322
252 303 258
NYCM03 NYCM -74.16803889 41.9465 87 117 30 9228 279
-100 379 182 -24 203 -36 1159 115 78 10 322
252 303 258
NYCM04 NYCM -74.16803889 41.9465 87 117 30 9228 279
-100 379 182 -24 203 -36 1159 115 78 10 322
252 303 258
NYCM05 NYCM -74.16803889 41.9465 87 117 30 9228 279
-100 379 182 -24 203 -36 1159 115 78 10 322
252 303 258
NYCM06 NYCM -74.16803889 41.9465 87 117 30 9228 279
-100 379 182 -24 203 -36 1159 115 78 10 322
252 303 258
NYCM07 NYCM -74.16803889 41.9465 87 117 30 9228 279
-100 379 182 -24 203 -36 1159 115 78 10 322
252 303 258
NYCM08 NYCM -74.16803889 41.9465 87 117 30 9228 279
-100 379 182 -24 203 -36 1159 115 78 10 322
252 303 258
NYCM09 NYCM -74.16803889 41.9465 87 117 30 9228 279
-100 379 182 -24 203 -36 1159 115 78 10 322
252 303 258
NYCM10 NYCM -74.16803889 41.9465 87 117 30 9228 279
-100 379 182 -24 203 -36 1159 115 78 10 322
252 303 258
NYCM11 NYCM -74.16803889 41.9465 87 117 30 9228 279
-100 379 182 -24 203 -36 1159 115 78 10 322
252 303 258
NYCM12 NYCM -74.16803889 41.9465 87 117 30 9228 279
-100 379 182 -24 203 -36 1159 115 78 10 322
252 303 258
NYCM13 NYCM -74.16803889 41.9465 87 117 30 9228 279
-100 379 182 -24 203 -36 1159 115 78 10 322
252 303 258
NYCM14 NYCM -74.16803889 41.9465 87 117 30 9228 279
-100 379 182 -24 203 -36 1159 115 78 10 322
252 303 258
NYCM15 NYCM -74.16803889 41.9465 87 117 30 9228 279
-100 379 182 -24 203 -36 1159 115 78 10 322

```
252	303	258
NYCM16	NYCM	-74.16803889	41.9465	87	117	30	9228	279
-100	379	182	-24	203	-36	1159	115	78	10	322
252	303	258
NYCM17	NYCM	-74.16803889	41.9465	87	117	30	9228	279
-100	379	182	-24	203	-36	1159	115	78	10	322
252	303	258
NYCM18	NYCM	-74.16803889	41.9465	87	117	30	9228	279
-100	379	182	-24	203	-36	1159	115	78	10	322
252	303	258
NYCM19	NYCM	-74.16803889	41.9465	87	117	30	9228	279
-100	379	182	-24	203	-36	1159	115	78	10	322
252	303	258
NYCM20	NYCM	-74.16803889	41.9465	87	117	30	9228	279
-100	379	182	-24	203	-36	1159	115	78	10	322
252	303	258
NYCM21	NYCM	-74.16803889	41.9465	87	117	30	9228	279
-100	379	182	-24	203	-36	1159	115	78	10	322
252	303	258
NYCM22	NYCM	-74.16803889	41.9465	87	117	30	9228	279
-100	379	182	-24	203	-36	1159	115	78	10	322
252	303	258
NYCM23	NYCM	-74.16803889	41.9465	87	117	30	9228	279
-100	379	182	-24	203	-36	1159	115	78	10	322
252	303	258
NYCM24	NYCM	-74.16803889	41.9465	87	117	30	9228	279
-100	379	182	-24	203	-36	1159	115	78	10	322
252	303	258
NYCM25	NYCM	-74.16803889	41.9465	87	117	30	9228	279
-100	379	182	-24	203	-36	1159	115	78	10	322
252	303	258
NYCM26	NYCM	-74.16803889	41.9465	87	117	30	9228	279
-100	379	182	-24	203	-36	1159	115	78	10	322
252	303	258
NYCM27	NYCM	-74.16803889	41.9465	87	117	30	9228	279
-100	379	182	-24	203	-36	1159	115	78	10	322
252	303	258
NYCM28	NYCM	-74.16803889	41.9465	87	117	30	9228	279
-100	379	182	-24	203	-36	1159	115	78	10	322
252	303	258
NYCM29	NYCM	-74.16803889	41.9465	87	117	30	9228	279
-100	379	182	-24	203	-36	1159	115	78	10	322
252	303	258
NYCM30	NYCM	-74.16803889	41.9465	87	117	30	9228	279
-100	379	182	-24	203	-36	1159	115	78	10	322
252	303	258
NYCM31	NYCM	-74.16803889	41.9465	87	117	30	9228	279
-100	379	182	-24	203	-36	1159	115	78	10	322
252	303	258
NYCM32	NYCM	-74.16803889	41.9465	87	117	30	9228	279
-100	379	182	-24	203	-36	1159	115	78	10	322
252	303	258
NYCM33	NYCM	-74.16803889	41.9465	87	117	30	9228	279
-100	379	182	-24	203	-36	1159	115	78	10	322
252	303	258
NYCM34	NYCM	-74.16803889	41.9465	87	117	30	9228	279
-100	379	182	-24	203	-36	1159	115	78	10	322
252	303	258
NYCM35	NYCM	-74.16803889	41.9465	87	117	30	9228	279
-100	379	182	-24	203	-36	1159	115	78	10	322
252	303	258
NYCM36	NYCM	-74.16803889	41.9465	87	117	30	9228	279
-100	379	182	-24	203	-36	1159	115	78	10	322
```

252 303 258
NYCM37 NYCM -74.16803889 41.9465 87 117 30 9228 279
-100 379 182 -24 203 -36 1159 115 78 10 322
252 303 258
NYCM38 NYCM -74.16803889 41.9465 87 117 30 9228 279
-100 379 182 -24 203 -36 1159 115 78 10 322
252 303 258
NYCM39 NYCM -74.16803889 41.9465 87 117 30 9228 279
-100 379 182 -24 203 -36 1159 115 78 10 322
252 303 258
NYCM40 NYCM -74.16803889 41.9465 87 117 30 9228 279
-100 379 182 -24 203 -36 1159 115 78 10 322
252 303 258
NYCM41 NYCM -74.16803889 41.9465 87 117 30 9228 279
-100 379 182 -24 203 -36 1159 115 78 10 322
252 303 258
NYCM42 NYCM -74.16803889 41.9465 87 117 30 9228 279
-100 379 182 -24 203 -36 1159 115 78 10 322
252 303 258
NYCM43 NYCM -74.16803889 41.9465 87 117 30 9228 279
-100 379 182 -24 203 -36 1159 115 78 10 322
252 303 258
NYCM44 NYCM -74.16803889 41.9465 87 117 30 9228 279
-100 379 182 -24 203 -36 1159 115 78 10 322
252 303 258
NYCM45 NYCM -74.16803889 41.9465 87 117 30 9228 279
-100 379 182 -24 203 -36 1159 115 78 10 322
252 303 258
NYCM46 NYCM -74.16803889 41.9465 87 117 30 9228 279
-100 379 182 -24 203 -36 1159 115 78 10 322
252 303 258
NYCM47 NYCM -74.16803889 41.9465 87 117 30 9228 279
-100 379 182 -24 203 -36 1159 115 78 10 322
252 303 258
NYCM48 NYCM -74.16803889 41.9465 87 117 30 9228 279
-100 379 182 -24 203 -36 1159 115 78 10 322
252 303 258
NYCM49 NYCM -74.16803889 41.9465 87 117 30 9228 279
-100 379 182 -24 203 -36 1159 115 78 10 322
252 303 258
NYCM50 NYCM -74.16803889 41.9465 87 117 30 9228 279
-100 379 182 -24 203 -36 1159 115 78 10 322
252 303 258
NHDF01 NHDF -71.26134722 43.10903889 78 128 32 9321 278
-120 398 32 -34 196 -47 1066 113 76 10 300
239 261 250
NHDF02 NHDF -71.26134722 43.10903889 78 128 32 9321 278
-120 398 32 -34 196 -47 1066 113 76 10 300
239 261 250
NHDF03 NHDF -71.26134722 43.10903889 78 128 32 9321 278
-120 398 32 -34 196 -47 1066 113 76 10 300
239 261 250
NHDF04 NHDF -71.26134722 43.10903889 78 128 32 9321 278
-120 398 32 -34 196 -47 1066 113 76 10 300
239 261 250
NHDF05 NHDF -71.26134722 43.10903889 78 128 32 9321 278
-120 398 32 -34 196 -47 1066 113 76 10 300
239 261 250
NHDF06 NHDF -71.26134722 43.10903889 78 128 32 9321 278
-120 398 32 -34 196 -47 1066 113 76 10 300
239 261 250
NHDF07 NHDF -71.26134722 43.10903889 78 128 32 9321 278
-120 398 32 -34 196 -47 1066 113 76 10 300

239	261	250								
NHDF08	NHDF	-71.26134722		43.10903889		78	128	32	9321	278
-120	398	32	-34	196	-47	1066	113	76	10	300
239	261	250								
NHDF09	NHDF	-71.26134722		43.10903889		78	128	32	9321	278
-120	398	32	-34	196	-47	1066	113	76	10	300
239	261	250								
NHDF10	NHDF	-71.26134722		43.10903889		78	128	32	9321	278
-120	398	32	-34	196	-47	1066	113	76	10	300
239	261	250								
NHDF11	NHDF	-71.26134722		43.10903889		78	128	32	9321	278
-120	398	32	-34	196	-47	1066	113	76	10	300
239	261	250								
NHDF12	NHDF	-71.26134722		43.10903889		78	128	32	9321	278
-120	398	32	-34	196	-47	1066	113	76	10	300
239	261	250								
NHDF13	NHDF	-71.26134722		43.10903889		78	128	32	9321	278
-120	398	32	-34	196	-47	1066	113	76	10	300
239	261	250								
NHDF14	NHDF	-71.26134722		43.10903889		78	128	32	9321	278
-120	398	32	-34	196	-47	1066	113	76	10	300
239	261	250								
NHDF15	NHDF	-71.26134722		43.10903889		78	128	32	9321	278
-120	398	32	-34	196	-47	1066	113	76	10	300
239	261	250								
NHDF16	NHDF	-71.26134722		43.10903889		78	128	32	9321	278
-120	398	32	-34	196	-47	1066	113	76	10	300
239	261	250								
NHDF17	NHDF	-71.26134722		43.10903889		78	128	32	9321	278
-120	398	32	-34	196	-47	1066	113	76	10	300
239	261	250								
NHDF18	NHDF	-71.26134722		43.10903889		78	128	32	9321	278
-120	398	32	-34	196	-47	1066	113	76	10	300
239	261	250								
NHDF19	NHDF	-71.26134722		43.10903889		78	128	32	9321	278
-120	398	32	-34	196	-47	1066	113	76	10	300
239	261	250								
NHDF20	NHDF	-71.26134722		43.10903889		78	128	32	9321	278
-120	398	32	-34	196	-47	1066	113	76	10	300
239	261	250								
NHDF21	NHDF	-71.26134722		43.10903889		78	128	32	9321	278
-120	398	32	-34	196	-47	1066	113	76	10	300
239	261	250								
NHDF22	NHDF	-71.26134722		43.10903889		78	128	32	9321	278
-120	398	32	-34	196	-47	1066	113	76	10	300
239	261	250								
NHDF23	NHDF	-71.26134722		43.10903889		78	128	32	9321	278
-120	398	32	-34	196	-47	1066	113	76	10	300
239	261	250								
NHDF24	NHDF	-71.26134722		43.10903889		78	128	32	9321	278
-120	398	32	-34	196	-47	1066	113	76	10	300
239	261	250								
NHDF25	NHDF	-71.26134722		43.10903889		78	128	32	9321	278
-120	398	32	-34	196	-47	1066	113	76	10	300
239	261	250								
NHDF26	NHDF	-71.26134722		43.10903889		78	128	32	9321	278
-120	398	32	-34	196	-47	1066	113	76	10	300
239	261	250								
NHDF27	NHDF	-71.26134722		43.10903889		78	128	32	9321	278
-120	398	32	-34	196	-47	1066	113	76	10	300
239	261	250								
NHDF28	NHDF	-71.26134722		43.10903889		78	128	32	9321	278
-120	398	32	-34	196	-47	1066	113	76	10	300

239	261	250								
NHDF29	NHDF	-71.26134722	43.10903889	78	128	32	9321	278		
-120	398	32	-34	196	-47	1066	113	76	10	300
239	261	250								
NHDF30	NHDF	-71.26134722	43.10903889	78	128	32	9321	278		
-120	398	32	-34	196	-47	1066	113	76	10	300
239	261	250								
NHDF31	NHDF	-71.26134722	43.10903889	78	128	32	9321	278		
-120	398	32	-34	196	-47	1066	113	76	10	300
239	261	250								
NHDF32	NHDF	-71.26134722	43.10903889	78	128	32	9321	278		
-120	398	32	-34	196	-47	1066	113	76	10	300
239	261	250								
NHDF33	NHDF	-71.26134722	43.10903889	78	128	32	9321	278		
-120	398	32	-34	196	-47	1066	113	76	10	300
239	261	250								
NHDF34	NHDF	-71.26134722	43.10903889	78	128	32	9321	278		
-120	398	32	-34	196	-47	1066	113	76	10	300
239	261	250								
NHDF35	NHDF	-71.26134722	43.10903889	78	128	32	9321	278		
-120	398	32	-34	196	-47	1066	113	76	10	300
239	261	250								
NHDF36	NHDF	-71.26134722	43.10903889	78	128	32	9321	278		
-120	398	32	-34	196	-47	1066	113	76	10	300
239	261	250								
NHDF37	NHDF	-71.26134722	43.10903889	78	128	32	9321	278		
-120	398	32	-34	196	-47	1066	113	76	10	300
239	261	250								
NHDF38	NHDF	-71.26134722	43.10903889	78	128	32	9321	278		
-120	398	32	-34	196	-47	1066	113	76	10	300
239	261	250								
NHDF39	NHDF	-71.26134722	43.10903889	78	128	32	9321	278		
-120	398	32	-34	196	-47	1066	113	76	10	300
239	261	250								
NHDF40	NHDF	-71.26134722	43.10903889	78	128	32	9321	278		
-120	398	32	-34	196	-47	1066	113	76	10	300
239	261	250								
NHDF41	NHDF	-71.26134722	43.10903889	78	128	32	9321	278		
-120	398	32	-34	196	-47	1066	113	76	10	300
239	261	250								
NHDF42	NHDF	-71.26134722	43.10903889	78	128	32	9321	278		
-120	398	32	-34	196	-47	1066	113	76	10	300
239	261	250								
NHDF43	NHDF	-71.26134722	43.10903889	78	128	32	9321	278		
-120	398	32	-34	196	-47	1066	113	76	10	300
239	261	250								
NHDF44	NHDF	-71.26134722	43.10903889	78	128	32	9321	278		
-120	398	32	-34	196	-47	1066	113	76	10	300
239	261	250								
NHDF45	NHDF	-71.26134722	43.10903889	78	128	32	9321	278		
-120	398	32	-34	196	-47	1066	113	76	10	300
239	261	250								
NHDF46	NHDF	-71.26134722	43.10903889	78	128	32	9321	278		
-120	398	32	-34	196	-47	1066	113	76	10	300
239	261	250								
NHDF47	NHDF	-71.26134722	43.10903889	78	128	32	9321	278		
-120	398	32	-34	196	-47	1066	113	76	10	300
239	261	250								
NHDF48	NHDF	-71.26134722	43.10903889	78	128	32	9321	278		
-120	398	32	-34	196	-47	1066	113	76	10	300
239	261	250								
NHDF49	NHDF	-71.26134722	43.10903889	78	128	32	9321	278		
-120	398	32	-34	196	-47	1066	113	76	10	300

239	261	250								
NHDF50	NHDF	-71.26134722		43.10903889		78	128	32	9321	278
-120	398	32	-34	196	-47	1066	113	76	10	300
239	261	250								
MNBL01	MNBL	-93.12775		45.32922222		65	115	25	11574	281
-172	453	205	-95	205	-95	753	113	20	51	315
69	315	69								
MNBL02	MNBL	-93.12775		45.32922222		65	115	25	11574	281
-172	453	205	-95	205	-95	753	113	20	51	315
69	315	69								
MNBL03	MNBL	-93.12775		45.32922222		65	115	25	11574	281
-172	453	205	-95	205	-95	753	113	20	51	315
69	315	69								
MNBL04	MNBL	-93.12775		45.32922222		65	115	25	11574	281
-172	453	205	-95	205	-95	753	113	20	51	315
69	315	69								
MNBL05	MNBL	-93.12775		45.32922222		65	115	25	11574	281
-172	453	205	-95	205	-95	753	113	20	51	315
69	315	69								
MNBL06	MNBL	-93.12775		45.32922222		65	115	25	11574	281
-172	453	205	-95	205	-95	753	113	20	51	315
69	315	69								
MNBL07	MNBL	-93.12775		45.32922222		65	115	25	11574	281
-172	453	205	-95	205	-95	753	113	20	51	315
69	315	69								
MNBL08	MNBL	-93.12775		45.32922222		65	115	25	11574	281
-172	453	205	-95	205	-95	753	113	20	51	315
69	315	69								
MNBL09	MNBL	-93.12775		45.32922222		65	115	25	11574	281
-172	453	205	-95	205	-95	753	113	20	51	315
69	315	69								
MNBL10	MNBL	-93.12775		45.32922222		65	115	25	11574	281
-172	453	205	-95	205	-95	753	113	20	51	315
69	315	69								
MNBL11	MNBL	-93.12775		45.32922222		65	115	25	11574	281
-172	453	205	-95	205	-95	753	113	20	51	315
69	315	69								
MNBL12	MNBL	-93.12775		45.32922222		65	115	25	11574	281
-172	453	205	-95	205	-95	753	113	20	51	315
69	315	69								
MNBL13	MNBL	-93.12775		45.32922222		65	115	25	11574	281
-172	453	205	-95	205	-95	753	113	20	51	315
69	315	69								
MNBL14	MNBL	-93.12775		45.32922222		65	115	25	11574	281
-172	453	205	-95	205	-95	753	113	20	51	315
69	315	69								
MNBL15	MNBL	-93.12775		45.32922222		65	115	25	11574	281
-172	453	205	-95	205	-95	753	113	20	51	315
69	315	69								
MNBL16	MNBL	-93.12775		45.32922222		65	115	25	11574	281
-172	453	205	-95	205	-95	753	113	20	51	315
69	315	69								
MNBL17	MNBL	-93.12775		45.32922222		65	115	25	11574	281
-172	453	205	-95	205	-95	753	113	20	51	315
69	315	69								
MNBL18	MNBL	-93.12775		45.32922222		65	115	25	11574	281
-172	453	205	-95	205	-95	753	113	20	51	315
69	315	69								
MNBL19	MNBL	-93.12775		45.32922222		65	115	25	11574	281
-172	453	205	-95	205	-95	753	113	20	51	315
69	315	69								
MNBL20	MNBL	-93.12775		45.32922222		65	115	25	11574	281
-172	453	205	-95	205	-95	753	113	20	51	315

```
69	315	69
MNBL21	MNBL	-93.12775	45.32922222	65	115	25	11574	281
-172	453	205	-95	205	-95	753	113	20	51	315
69	315	69
MNBL22	MNBL	-93.12775	45.32922222	65	115	25	11574	281
-172	453	205	-95	205	-95	753	113	20	51	315
69	315	69
MNBL23	MNBL	-93.12775	45.32922222	65	115	25	11574	281
-172	453	205	-95	205	-95	753	113	20	51	315
69	315	69
MNBL24	MNBL	-93.12775	45.32922222	65	115	25	11574	281
-172	453	205	-95	205	-95	753	113	20	51	315
69	315	69
MNBL25	MNBL	-93.12775	45.32922222	65	115	25	11574	281
-172	453	205	-95	205	-95	753	113	20	51	315
69	315	69
MNBL26	MNBL	-93.12775	45.32922222	65	115	25	11574	281
-172	453	205	-95	205	-95	753	113	20	51	315
69	315	69
MNBL27	MNBL	-93.12775	45.32922222	65	115	25	11574	281
-172	453	205	-95	205	-95	753	113	20	51	315
69	315	69
MNBL28	MNBL	-93.12775	45.32922222	65	115	25	11574	281
-172	453	205	-95	205	-95	753	113	20	51	315
69	315	69
MNBL29	MNBL	-93.12775	45.32922222	65	115	25	11574	281
-172	453	205	-95	205	-95	753	113	20	51	315
69	315	69
MNBL30	MNBL	-93.12775	45.32922222	65	115	25	11574	281
-172	453	205	-95	205	-95	753	113	20	51	315
69	315	69
MNBL31	MNBL	-93.12775	45.32922222	65	115	25	11574	281
-172	453	205	-95	205	-95	753	113	20	51	315
69	315	69
MNBL32	MNBL	-93.12775	45.32922222	65	115	25	11574	281
-172	453	205	-95	205	-95	753	113	20	51	315
69	315	69
MNBL33	MNBL	-93.12775	45.32922222	65	115	25	11574	281
-172	453	205	-95	205	-95	753	113	20	51	315
69	315	69
MNBL34	MNBL	-93.12775	45.32922222	65	115	25	11574	281
-172	453	205	-95	205	-95	753	113	20	51	315
69	315	69
MNBL35	MNBL	-93.12775	45.32922222	65	115	25	11574	281
-172	453	205	-95	205	-95	753	113	20	51	315
69	315	69
MNBL36	MNBL	-93.12775	45.32922222	65	115	25	11574	281
-172	453	205	-95	205	-95	753	113	20	51	315
69	315	69
MNBL37	MNBL	-93.12775	45.32922222	65	115	25	11574	281
-172	453	205	-95	205	-95	753	113	20	51	315
69	315	69
MNBL38	MNBL	-93.12775	45.32922222	65	115	25	11574	281
-172	453	205	-95	205	-95	753	113	20	51	315
69	315	69
MNBL39	MNBL	-93.12775	45.32922222	65	115	25	11574	281
-172	453	205	-95	205	-95	753	113	20	51	315
69	315	69
MNBL40	MNBL	-93.12775	45.32922222	65	115	25	11574	281
-172	453	205	-95	205	-95	753	113	20	51	315
69	315	69
MNBL41	MNBL	-93.12775	45.32922222	65	115	25	11574	281
-172	453	205	-95	205	-95	753	113	20	51	315
```

```
69	315	69
MNBL42	MNBL	-93.12775	45.32922222	65	115	25	11574	281
-172	453	205	-95	205	-95	753	113	20	51	315
69	315	69
MNBL43	MNBL	-93.12775	45.32922222	65	115	25	11574	281
-172	453	205	-95	205	-95	753	113	20	51	315
69	315	69
MNBL44	MNBL	-93.12775	45.32922222	65	115	25	11574	281
-172	453	205	-95	205	-95	753	113	20	51	315
69	315	69
MNBL45	MNBL	-93.12775	45.32922222	65	115	25	11574	281
-172	453	205	-95	205	-95	753	113	20	51	315
69	315	69
MNBL46	MNBL	-93.12775	45.32922222	65	115	25	11574	281
-172	453	205	-95	205	-95	753	113	20	51	315
69	315	69
MNBL47	MNBL	-93.12775	45.32922222	65	115	25	11574	281
-172	453	205	-95	205	-95	753	113	20	51	315
69	315	69
MNBL48	MNBL	-93.12775	45.32922222	65	115	25	11574	281
-172	453	205	-95	205	-95	753	113	20	51	315
69	315	69
MNBL49	MNBL	-93.12775	45.32922222	65	115	25	11574	281
-172	453	205	-95	205	-95	753	113	20	51	315
69	315	69
MNBL50	MNBL	-93.12775	45.32922222	65	115	25	11574	281
-172	453	205	-95	205	-95	753	113	20	51	315
69	315	69
VABS01	VASB	-80.01676111	37.38003611	123	129	37	7857	298
-46	344	204	19	222	19	1018	100	72	11	282
218	279	218
VABS02	VASB	-80.01676111	37.38003611	123	129	37	7857	298
-46	344	204	19	222	19	1018	100	72	11	282
218	279	218
VABS03	VASB	-80.01676111	37.38003611	123	129	37	7857	298
-46	344	204	19	222	19	1018	100	72	11	282
218	279	218
VABS04	VASB	-80.01676111	37.38003611	123	129	37	7857	298
-46	344	204	19	222	19	1018	100	72	11	282
218	279	218
VABS05	VASB	-80.01676111	37.38003611	123	129	37	7857	298
-46	344	204	19	222	19	1018	100	72	11	282
218	279	218
VABS06	VASB	-80.01676111	37.38003611	123	129	37	7857	298
-46	344	204	19	222	19	1018	100	72	11	282
218	279	218
VABS07	VASB	-80.01676111	37.38003611	123	129	37	7857	298
-46	344	204	19	222	19	1018	100	72	11	282
218	279	218
VABS08	VASB	-80.01676111	37.38003611	123	129	37	7857	298
-46	344	204	19	222	19	1018	100	72	11	282
218	279	218
VABS09	VASB	-80.01676111	37.38003611	123	129	37	7857	298
-46	344	204	19	222	19	1018	100	72	11	282
218	279	218
VABS10	VASB	-80.01676111	37.38003611	123	129	37	7857	298
-46	344	204	19	222	19	1018	100	72	11	282
218	279	218
VABS11	VASB	-80.01676111	37.38003611	123	129	37	7857	298
-46	344	204	19	222	19	1018	100	72	11	282
218	279	218
VABS12	VASB	-80.01676111	37.38003611	123	129	37	7857	298
-46	344	204	19	222	19	1018	100	72	11	282
```

218	279	218								
VABS13	VASB	-80.01676111		37.38003611		123	129	37	7857	298
-46	344	204	19	222	19	1018	100	72	11	282
218	279	218								
VABS14	VASB	-80.01676111		37.38003611		123	129	37	7857	298
-46	344	204	19	222	19	1018	100	72	11	282
218	279	218								
VABS15	VASB	-80.01676111		37.38003611		123	129	37	7857	298
-46	344	204	19	222	19	1018	100	72	11	282
218	279	218								
VABS16	VASB	-80.01676111		37.38003611		123	129	37	7857	298
-46	344	204	19	222	19	1018	100	72	11	282
218	279	218								
VABS17	VASB	-80.01676111		37.38003611		123	129	37	7857	298
-46	344	204	19	222	19	1018	100	72	11	282
218	279	218								
VABS18	VASB	-80.01676111		37.38003611		123	129	37	7857	298
-46	344	204	19	222	19	1018	100	72	11	282
218	279	218								
VABS19	VASB	-80.01676111		37.38003611		123	129	37	7857	298
-46	344	204	19	222	19	1018	100	72	11	282
218	279	218								
VABS20	VASB	-80.01676111		37.38003611		123	129	37	7857	298
-46	344	204	19	222	19	1018	100	72	11	282
218	279	218								
VABS21	VASB	-80.01676111		37.38003611		123	129	37	7857	298
-46	344	204	19	222	19	1018	100	72	11	282
218	279	218								
VABS22	VASB	-80.01676111		37.38003611		123	129	37	7857	298
-46	344	204	19	222	19	1018	100	72	11	282
218	279	218								
VABS23	VASB	-80.01676111		37.38003611		123	129	37	7857	298
-46	344	204	19	222	19	1018	100	72	11	282
218	279	218								
VABS24	VASB	-80.01676111		37.38003611		123	129	37	7857	298
-46	344	204	19	222	19	1018	100	72	11	282
218	279	218								
VABS25	VASB	-80.01676111		37.38003611		123	129	37	7857	298
-46	344	204	19	222	19	1018	100	72	11	282
218	279	218								
VABS26	VASB	-80.01676111		37.38003611		123	129	37	7857	298
-46	344	204	19	222	19	1018	100	72	11	282
218	279	218								
VABS27	VASB	-80.01676111		37.38003611		123	129	37	7857	298
-46	344	204	19	222	19	1018	100	72	11	282
218	279	218								
VABS28	VASB	-80.01676111		37.38003611		123	129	37	7857	298
-46	344	204	19	222	19	1018	100	72	11	282
218	279	218								
VABS29	VASB	-80.01676111		37.38003611		123	129	37	7857	298
-46	344	204	19	222	19	1018	100	72	11	282
218	279	218								
VABS30	VASB	-80.01676111		37.38003611		123	129	37	7857	298
-46	344	204	19	222	19	1018	100	72	11	282
218	279	218								
VABS31	VASB	-80.01676111		37.38003611		123	129	37	7857	298
-46	344	204	19	222	19	1018	100	72	11	282
218	279	218								
VABS32	VASB	-80.01676111		37.38003611		123	129	37	7857	298
-46	344	204	19	222	19	1018	100	72	11	282
218	279	218								
VABS33	VASB	-80.01676111		37.38003611		123	129	37	7857	298
-46	344	204	19	222	19	1018	100	72	11	282

218	279	218								
VABS34	VASB	-80.01676111		37.38003611		123	129	37	7857	298
-46	344	204	19	222	19	1018	100	72	11	282
218	279	218								
VABS35	VASB	-80.01676111		37.38003611		123	129	37	7857	298
-46	344	204	19	222	19	1018	100	72	11	282
218	279	218								
VABS36	VASB	-80.01676111		37.38003611		123	129	37	7857	298
-46	344	204	19	222	19	1018	100	72	11	282
218	279	218								
VABS37	VASB	-80.01676111		37.38003611		123	129	37	7857	298
-46	344	204	19	222	19	1018	100	72	11	282
218	279	218								
VABS38	VASB	-80.01676111		37.38003611		123	129	37	7857	298
-46	344	204	19	222	19	1018	100	72	11	282
218	279	218								
VABS39	VASB	-80.01676111		37.38003611		123	129	37	7857	298
-46	344	204	19	222	19	1018	100	72	11	282
218	279	218								
VABS40	VASB	-80.01676111		37.38003611		123	129	37	7857	298
-46	344	204	19	222	19	1018	100	72	11	282
218	279	218								
VABS41	VASB	-80.01676111		37.38003611		123	129	37	7857	298
-46	344	204	19	222	19	1018	100	72	11	282
218	279	218								
VABS42	VASB	-80.01676111		37.38003611		123	129	37	7857	298
-46	344	204	19	222	19	1018	100	72	11	282
218	279	218								
VABS43	VASB	-80.01676111		37.38003611		123	129	37	7857	298
-46	344	204	19	222	19	1018	100	72	11	282
218	279	218								
VABS44	VASB	-80.01676111		37.38003611		123	129	37	7857	298
-46	344	204	19	222	19	1018	100	72	11	282
218	279	218								
VABS45	VASB	-80.01676111		37.38003611		123	129	37	7857	298
-46	344	204	19	222	19	1018	100	72	11	282
218	279	218								
VABS46	VASB	-80.01676111		37.38003611		123	129	37	7857	298
-46	344	204	19	222	19	1018	100	72	11	282
218	279	218								
VABS47	VASB	-80.01676111		37.38003611		123	129	37	7857	298
-46	344	204	19	222	19	1018	100	72	11	282
218	279	218								
VABS48	VASB	-80.01676111		37.38003611		123	129	37	7857	298
-46	344	204	19	222	19	1018	100	72	11	282
218	279	218								
VABS49	VASB	-80.01676111		37.38003611		123	129	37	7857	298
-46	344	204	19	222	19	1018	100	72	11	282
218	279	218								
VABS50	VASB	-80.01676111		37.38003611		123	129	37	7857	298
-46	344	204	19	222	19	1018	100	72	11	282
218	279	218								
NCAV01	NCAV	-82.53184167		35.61639722		124	134	40	7228	288
-45	333	216	80	216	29	1159	116	81	12	321
253	321	259								
NCAV02	NCAV	-82.53184167		35.61639722		124	134	40	7228	288
-45	333	216	80	216	29	1159	116	81	12	321
253	321	259								
NCAV03	NCAV	-82.53184167		35.61639722		124	134	40	7228	288
-45	333	216	80	216	29	1159	116	81	12	321
253	321	259								
NCAV04	NCAV	-82.53184167		35.61639722		124	134	40	7228	288
-45	333	216	80	216	29	1159	116	81	12	321

253	321	259								
NCAV05	NCAV	-82.53184167		35.61639722		124	134	40	7228	288
-45	333	216	80	216	29	1159	116	81	12	321
253	321	259								
NCAV06	NCAV	-82.53184167		35.61639722		124	134	40	7228	288
-45	333	216	80	216	29	1159	116	81	12	321
253	321	259								
NCAV07	NCAV	-82.53184167		35.61639722		124	134	40	7228	288
-45	333	216	80	216	29	1159	116	81	12	321
253	321	259								
NCAV08	NCAV	-82.53184167		35.61639722		124	134	40	7228	288
-45	333	216	80	216	29	1159	116	81	12	321
253	321	259								
NCAV09	NCAV	-82.53184167		35.61639722		124	134	40	7228	288
-45	333	216	80	216	29	1159	116	81	12	321
253	321	259								
NCAV10	NCAV	-82.53184167		35.61639722		124	134	40	7228	288
-45	333	216	80	216	29	1159	116	81	12	321
253	321	259								
NCAV11	NCAV	-82.53184167		35.61639722		124	134	40	7228	288
-45	333	216	80	216	29	1159	116	81	12	321
253	321	259								
NCAV12	NCAV	-82.53184167		35.61639722		124	134	40	7228	288
-45	333	216	80	216	29	1159	116	81	12	321
253	321	259								
NCAV13	NCAV	-82.53184167		35.61639722		124	134	40	7228	288
-45	333	216	80	216	29	1159	116	81	12	321
253	321	259								
NCAV14	NCAV	-82.53184167		35.61639722		124	134	40	7228	288
-45	333	216	80	216	29	1159	116	81	12	321
253	321	259								
NCAV15	NCAV	-82.53184167		35.61639722		124	134	40	7228	288
-45	333	216	80	216	29	1159	116	81	12	321
253	321	259								
NCAV16	NCAV	-82.53184167		35.61639722		124	134	40	7228	288
-45	333	216	80	216	29	1159	116	81	12	321
253	321	259								
NCAV17	NCAV	-82.53184167		35.61639722		124	134	40	7228	288
-45	333	216	80	216	29	1159	116	81	12	321
253	321	259								
NCAV18	NCAV	-82.53184167		35.61639722		124	134	40	7228	288
-45	333	216	80	216	29	1159	116	81	12	321
253	321	259								
NCAV19	NCAV	-82.53184167		35.61639722		124	134	40	7228	288
-45	333	216	80	216	29	1159	116	81	12	321
253	321	259								
NCAV20	NCAV	-82.53184167		35.61639722		124	134	40	7228	288
-45	333	216	80	216	29	1159	116	81	12	321
253	321	259								
NCAV21	NCAV	-82.53184167		35.61639722		124	134	40	7228	288
-45	333	216	80	216	29	1159	116	81	12	321
253	321	259								
NCAV22	NCAV	-82.53184167		35.61639722		124	134	40	7228	288
-45	333	216	80	216	29	1159	116	81	12	321
253	321	259								
NCAV23	NCAV	-82.53184167		35.61639722		124	134	40	7228	288
-45	333	216	80	216	29	1159	116	81	12	321
253	321	259								
NCAV24	NCAV	-82.53184167		35.61639722		124	134	40	7228	288
-45	333	216	80	216	29	1159	116	81	12	321
253	321	259								
NCAV25	NCAV	-82.53184167		35.61639722		124	134	40	7228	288
-45	333	216	80	216	29	1159	116	81	12	321

253	321	259								
NCAV26	NCAV	-82.53184167		35.61639722		124	134	40	7228	288
-45	333	216	80	216	29	1159	116	81	12	321
253	321	259								
NCAV27	NCAV	-82.53184167		35.61639722		124	134	40	7228	288
-45	333	216	80	216	29	1159	116	81	12	321
253	321	259								
NCAV28	NCAV	-82.53184167		35.61639722		124	134	40	7228	288
-45	333	216	80	216	29	1159	116	81	12	321
253	321	259								
NCAV29	NCAV	-82.53184167		35.61639722		124	134	40	7228	288
-45	333	216	80	216	29	1159	116	81	12	321
253	321	259								
NCAV30	NCAV	-82.53184167		35.61639722		124	134	40	7228	288
-45	333	216	80	216	29	1159	116	81	12	321
253	321	259								
NCAV31	NCAV	-82.53184167		35.61639722		124	134	40	7228	288
-45	333	216	80	216	29	1159	116	81	12	321
253	321	259								
NCAV32	NCAV	-82.53184167		35.61639722		124	134	40	7228	288
-45	333	216	80	216	29	1159	116	81	12	321
253	321	259								
NCAV33	NCAV	-82.53184167		35.61639722		124	134	40	7228	288
-45	333	216	80	216	29	1159	116	81	12	321
253	321	259								
NCAV34	NCAV	-82.53184167		35.61639722		124	134	40	7228	288
-45	333	216	80	216	29	1159	116	81	12	321
253	321	259								
NCAV35	NCAV	-82.53184167		35.61639722		124	134	40	7228	288
-45	333	216	80	216	29	1159	116	81	12	321
253	321	259								
NCAV36	NCAV	-82.53184167		35.61639722		124	134	40	7228	288
-45	333	216	80	216	29	1159	116	81	12	321
253	321	259								
NCAV37	NCAV	-82.53184167		35.61639722		124	134	40	7228	288
-45	333	216	80	216	29	1159	116	81	12	321
253	321	259								
NCAV38	NCAV	-82.53184167		35.61639722		124	134	40	7228	288
-45	333	216	80	216	29	1159	116	81	12	321
253	321	259								
NCAV39	NCAV	-82.53184167		35.61639722		124	134	40	7228	288
-45	333	216	80	216	29	1159	116	81	12	321
253	321	259								
NCAV40	NCAV	-82.53184167		35.61639722		124	134	40	7228	288
-45	333	216	80	216	29	1159	116	81	12	321
253	321	259								
NCAV41	NCAV	-82.53184167		35.61639722		124	134	40	7228	288
-45	333	216	80	216	29	1159	116	81	12	321
253	321	259								
NCAV42	NCAV	-82.53184167		35.61639722		124	134	40	7228	288
-45	333	216	80	216	29	1159	116	81	12	321
253	321	259								
NCAV43	NCAV	-82.53184167		35.61639722		124	134	40	7228	288
-45	333	216	80	216	29	1159	116	81	12	321
253	321	259								
NCAV44	NCAV	-82.53184167		35.61639722		124	134	40	7228	288
-45	333	216	80	216	29	1159	116	81	12	321
253	321	259								
NCAV45	NCAV	-82.53184167		35.61639722		124	134	40	7228	288
-45	333	216	80	216	29	1159	116	81	12	321
253	321	259								
NCAV46	NCAV	-82.53184167		35.61639722		124	134	40	7228	288
-45	333	216	80	216	29	1159	116	81	12	321

253	321	259								
NCAV47	NCAV	-82.53184167		35.61639722		124	134	40	7228	288
-45	333	216	80	216	29	1159	116	81	12	321
253	321	259								
NCAV48	NCAV	-82.53184167		35.61639722		124	134	40	7228	288
-45	333	216	80	216	29	1159	116	81	12	321
253	321	259								
NCAV49	NCAV	-82.53184167		35.61639722		124	134	40	7228	288
-45	333	216	80	216	29	1159	116	81	12	321
253	321	259								
NCAV50	NCAV	-82.53184167		35.61639722		124	134	40	7228	288
-45	333	216	80	216	29	1159	116	81	12	321
253	321	259								
