# Supplementary material for: Single-Locus versus Multilocus Patterns of Local Adaptation to Climate in Eastern White Pine (Pinus strobus, Pinaceae)
Source: PLoS One. 2016 Jul 7;11(7):e0158691. doi: 10.1371/journal.pone.0158691 (PMC4936701; doi:10.1371/journal.pone.0158691)
Supplement: S1 Text — (DOCX) [file pone.0158691.s017.docx]

**SUPPORTING INFORMATION**

**Supplemental Text S1**

**Single-locus versus Multilocus Patterns of Local Adaptation to Climate in Eastern White Pine (*Pinus strobus*, Pinaceae)**

**Om P. Rajora^1,4^**
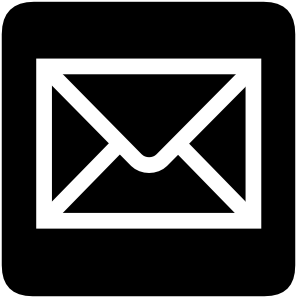
**, Andrew J. Eckert^2,4^, and John W. R. Zinck^1,3^**

^1^Faculty of Forestry and Environmental Management, University of New Brunswick, Fredericton, New Brunswick, Canada

^2^Department of Biology, Virginia Commonwealth University, Richmond, Virginia, United States of America

^3^Current address: Athletigen Technologies Inc., Kanata, Ontario, Canada

^4^ Joint first authors


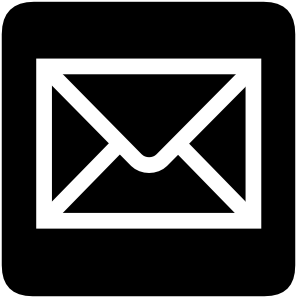
**Author for Correspondence:**

Om P. Rajora

Faculty of Forestry and Environmental Management

University of New Brunswick

Fredericton, New Brunswick, Canada

Telephone: +1-506- 458 7477; Fax: +1-506-453-3538

Email: Om.Rajora@unb.ca

**Short title:** Genetic architecture of local adaptation to climate

May 27, 2016

**Supplemental Text File S1**

**Supplemental Materials and Methods**

**Microsatellite genotyping**

Twelve nuclear microsatellites (SSRs) were used for genotyping (Table S1). For each SSR marker, one of the two primers was modified to accept a fluorescently-labelled M13 tail (700 nm or 800 nm wavelength). Polymerase chain reaction (PCR) reactions for the microsatellite amplification were prepared in a volume of 10 μL. The reaction mixtures contained 10 to 15 ng of DNA, 2 μL of 5x PCR Buffer (Promega, Madison, WI, USA), 0.5 μL of 2 mM MgCl_2_, 1 μL of 4 mM dNTPs, 0.1 μL of 10 mM untailed primer, 0.1 μL of 10 mM fluorescently-labelled M13 primers, 0.06 μL of 10 mM M13 tailed primer, 0.1 μL of 5 U/μL Taq DNA polymerase, and 5.09 μL of nuclease free water. We performed PCR amplifications with a “touchdown” step from 65 °C with reduction of 1^o^C per cycle to the annealing temperatures (Echt *et al.,* 1996*;* Rajora *et al*., 2000) and consisted of initial denaturation at 94°C for 30 s, followed by 27 cycles each of 94°C for 30 s, the annealing temperature for 30 s, and an extension of 72°C for 30 s, which was followed by a final extension step at 72°C for 3 min. PCR reactions were performed using 96 well EP Gradient S MasterCyclers (Eppendorf, Germany).

Microsatellite polymorphisms were identified by visualizing PCR products on a 6% agarose gel matrix (National Diagnostic Ureagel-6), suspended in a TBE buffer using Licor Biosciences IR4300 DNA analyzers (Licor, Lincoln, Nebraska, USA). To determine microsatellite fragment lengths, five LiCor 50-350 bp molecular weight size standards were included on each gel. We used Licor Biosciences Saga Generation 2 (v. 3.3, Lincoln Nebraska, USA) to score microsatellite genotype data, which was double checked and verified manually.

***F*_ST_ outliers and BayeScan**

Outliers with respect to population structure were identified using BayeScan (Foll and Gaggiotti, 2008). This method decomposes the overall value of *F*_ST_ into components due to populations (β) and to loci (α) using logistic regression. The model is formulated in a Bayesian context with parameters estimated via Markov Chain Monte Carlo (MCMC). Values for α that exclude 0 can be considered as outliers, due to the need for them to explain the observed pattern as opposed to a null model with just population-specific components. These two models are explored via a form of MCMC inference known as reversible-jump MCMC, where the jumps are between a model with only population-specific components and a model with both locus-specific and population-specific components. The estimated value of α is indicative of the type of selection needed to explain the result. A locus with an estimated α that is significantly greater than 0 is indicative of diversifying selection, whereas an estimated α that is significantly less than 0 is indicative of either balancing or purifying selection for a global optimum.

Parameters were estimated using three independent runs of the MCMC sampler for four different values (10:1 (default), 100:1, 1000:1, and 10000:1) for the prior odds of the null model (*i.e.* neutral evolution). The default settings were used for the MCMC sampler in each case: 20 pilot runs, each of length 5,000 steps, a burnin of 50,000 steps followed by an additional 50,000 steps thinned every 10 steps. Convergence of the three independent runs was assessed through estimation of effective sample size (ESS) for the log-likelihood value using the *EffectiveSize* function in the CODA library of R, time series plots of the log-likelihood value against sampling time in the MCMC chain using the *ts.plot* function, and pairwise Kolmogorov-Smirnov tests (α = 0.05) of the posterior distribution of the log-likelihood values for each independent run of the MCMC sampler for each of the four values of the prior odds of the null model. This general scheme was implemented for both marker sets, so that a total of 24 runs of the MCMC sampler as described above were made in total. All runs were carried out using the WineBottler v. 1.7.9 (<http://winebottler.kronenberg.org/>) PC emulator and the pre-compiled, graphic user interface version of BayeScan v. 2.1 ([http://cmpg. unibe.ch/software/BayeScan/download.html](http://cmpg.unibe.ch/software/BayeScan/download.html)).

**Estimation of intragenic and intergenic linkage disequilibrium**

Pairwise linkage disequilibrium (LD) was estimated for all comparisons of the 44 SNPs (*n* = 903) using the *LD* function from the genetics library in R. We focused on both the squared allelic correlation (*r*^2^) and the absolute value of *D*’. The former is standardized by allele frequencies, whereas the latter is standardized by the maximum value of *D*. Pairwise values of each statistic were classified as intragenic and intergenic based on whether or not the pair of SNPs was from the same or different gene (contigs). Here contigs are defined as the expressed sequence tag (EST) contig, in other words the unique EST cluster derived from assembly of raw EST reads, from which primers were designed for SNP discovery and genotyping. These varied in length from < 200 bp to > 600 bp in length.

**Supplemental Results and Conclusions**

***F*_ST_ outliers and BayeScan**

Results relevant to the study system obtained from application of BayeScan are summarized in the main text. Here, we summarize the technicalities of the MCMC process used to estimate parameters.

Visual inspection of the log-likelihood values for the three replicated MCMC runs per value of the prior odds assigned to the null model generally were consistent with the idea that convergence was attained in each run. First, similar mean values of the log-likelihood were reached across the three runs. Examples for each data set (*i.e.* SNPs and SSRs) are given in Figure S2. Second, the degree of mixing along the Markov chains was acceptable, although much lower for the SSR data. In general, there was less autocorrelation (i.e. more mixing) for the MCMC runs based on SNP data as compared to the SSR data. This was reflected in the effective sample size (ESS) estimates for the log-likelihood for results based on SNP data as compared with SSR data. In general, the values of ESS were larger for SNP data as compared with SSR data (Figure S3). All ESS estimates, however, were > 100. With ESS values of this size, model parameters were estimated well (*i.e.* they had Gaussian-like posterior distributions; Figure S4). Third, pairwise Kolmogorov-Smirnov tests were only significantly different among runs with different prior odds for the null model (Figure S5). Taken together this implies that for a given prior odds of the null model, convergence was reached for the three independent MCMC runs.

**Estimation of intragenic and intergenic linkage disequilibrium**

Pairwise estimates of linkage disequilibrium were divided into categories of whether or not the SNPs forming the pair were located in the same gene or not. Linkage disequilibrium estimates for SNPs located in the same gene were labeled as intragenic, while estimates involving SNPs located in different genes (contigs) were considered intergenic. Distributions for these quantities are illustrated in Figure S6. For each statistic, there was no statistically significant difference between distributions of each quantity between categories (Wilcoxon rank sum tests: *P* > 0.05). Differences, however, were apparent in the tails, with *r*^2^ having significantly more values greater than 0.40 for the intergenic comparisons (*Χ*^2^-test: *P* = 0.025).

**Geographical patterns of genetic diversity and disequilibrium**

*F*_IS_ approached 0 in the most recently colonized populations located in the northeastern portion of the range. This was due to the southern populations exhibiting strongly positive (SSRs) or strongly negative (SNPs) values of *F*_IS_. For SSRs, the strongly positive values of *F*_IS_ could be due to the presence of null alleles. If present, null alleles would have extremely low frequencies in our sample, since we observed only 0.23% (range: 0.00% - 1.10%) missing data on average across SSR loci (*n* = 1,450 trees). Alternatively, these positive values could represent a sampling bias for hypervariable loci, so that even the 50 trees sampled from each local population were not enough to capture heterozygotes for many of the alleles. More likely, though, it was the influence of post-Pleistocene expansion and the serial bottlenecks accompanying this expansion that influenced this pattern.

All of the previous studies that employed all or most of the 12 SSR markers that we have used for genetic diversity assessment in eastern white pine have consistently reported a deficiency of heterozygotes in comparison to Hardy-Weinberg equilibrium from different part of the species’ range (Rajora *et al*., 2000; Marquardt and Epperson 2004; Marquardt *et al*., 2007; Mehes *et al*., 2009; Chhatre and Rajora, 2014). A null allele was reported in low frequency at one (RPS60) of the 12 SSR loci (Rajora *et al*., 2000). We observed heterozygote deficiency all across the 12 SSR markers and not just at RPS60. It must be noted that Marquardt and Epperson (2004) and Marquardt *et al.* (2007) used only 7 of these SSR markers that showed no evidence of the occurrence of null alleles. Even then they observed significant deficiency of heterozygotes in eastern white pine populations from Michigan and Wisconsin. The observation of deficiency of heterozygotes for SSR markers appears to be a common phenomenon in other conifer trees, whether they have predominantly outbreeding system (e.g., *Picea glauca* – Rajora *et al.*, 2005; Fageria and Rajora 2013; *Picea mariana* – Rajora lab) or mixed mating system (e.g., *Thuja occidentalis* – Pandey and Rajora 2012), including eastern white pine’s sister species, western white pine (*Pinus monticola*) (Mehes *et al.*, 2009). Forest tree populations often do not conform to Hardy-Weinberg equilibrium because they are not at equilibrium; many Hardy-Weinberg equilibrium assumptions are violated in them, such as non-overlapping generations, no selection, no migration, no mutation, and equal viability of gametes and genotypes. A trend of decreasing heterozygote deficiency for SSRs from south to north and west to east is somewhat puzzling. It is unlikely due to genotype scoring errors because the same high stringency was applied to all populations with genotype scoring double- or triple-checked manually and using genotyping software. It may be due to differing strength of geographical effects on *H*_o_ versus *H_e_* (Figure S5). Additional work is needed to explain this trend. Nevertheless, the south to north and west to east trends in genetic diversity fit well with demography and postglacial migration/expansion of eastern white pine populations from a southern refugium along Atlantic seaboard and then to west through a route west of Appalachians (Zinck and Rajora 2016). The glacial refugial populations are expected to have higher genetic diversity, which will be reduced as we go farther from the refugial populations because of bottleneck and founder’s effects. This was exactly the case in our study. For example, the mean number of alleles per locus, and *H*_e_ were 15.25, and 0.82, respectively, in the southern North Carolina eastern white pine population versus 8.77, and 0.70, respectively, in a northern Nova Scotia population.

For SNPs, the negative values of *F*_IS_ may reflect the often-observed trend of increasing heterozygosity with stand age, which has been attributed to the action of natural selection (reviewed by Bush and Smouse, 1992). We sampled mature to old-age trees of eastern white pine in our study. More intensive natural selection for heterozygote advantage in candidate genes would be expected than that in random or neutral markers. The southern eastern white pine populations are evolutionarily older and have higher genetic diversity than the northern populations; thus, are expected to exhibit higher excess of heterozygotes at functional loci than the northern populations – the trend that we observed. Finally, as pointed out above, forest tree populations cannot be expected to conform to Hardy-Weinberg equilibrium. Heterozygous excess has been previously reported for allozyme markers in eastern white pine (Rajora *et al*., 1998) and for allozymes (e.g., *Picea mariana* – Yeh *et al.*, 1986) and SNPs (e.g., *Picea glauca* – Namroud *et al*., 2008) in other conifers. It is notable that the extent of heterozygote excess for SNPs differed among populations in *Picea glauca* (Namroud *et al*., 2008) as we observed. The genes coding for allozymes are functional and should have similar mutation rates as the candidate genes from which the SNPs were derived in our study.

**Supplemental References**

Bush RM, Smouse PE. 1992. Evidence for adaptive significance of allozymes in forest trees. *New Forests* 6: 179–196.

Chhatre VE, Rajora OP. 2014. Genetic divergence and signatures of natural selection in marginal populations of a keystone, long-lived conifer, eastern white pine (*Pinus strobus*) from northern Ontario. *PLoS ONE* 9: e97291.

Echt CS, May-Marquardt P, Hseih M, Zahorchak R. 1996. Characterization of microsatellite markers in eastern white pine. *Genome* 39: 1102–1108.

Fageria MS, Rajora OP. 2013. Effects of harvesting of increasing intensities on genetic diversity and population structure of white spruce. *Evolutionary Applications* 6:778-794.

Foll M, Gaggiotti O. 2008. A genome-scan method to identify selected loci appropriate for both dominant and codominant marker: A Bayesian perspective. *Genetics* 180: 977–993.

Marquardt PE, Epperson BK. 2004. Spatial and population genetic structure of microsatellites in white pine. *Molecular Ecology* 13: 3305–3315.

Marquardt PE, Echt CS, Epperson BK, Pubanz DM. 2007. Genetic structure, diversity, and inbreeding of eastern white pine under different management conditions. *Canadian Journal of Forest Research* 37: 2652–2662.

Mehes M, Nkongolo KK, Michael P. 2009. Assessing genetic diversity and structure of fragmented populations of eastern white pine (*Pinus strobus*) and western white pine (*P. monticola*) for conservation management. *Journal of Plant Ecology* 2: 143–151.

Namroud MC, Beaulieu J, Juge N, Laroche J, Bousquet J. 2008. Scanning the genome for gene single nucleotide polymorphisms involved in adaptive population differentiation in white spruce. *Molecular Ecology* 17: 3599–3613.

Pandey M, Rajora OP. 2012. Genetic diversity and differentiation of core versus peripheral populations of eastern white cedar, *Thuja occidentalis* L. (Cupressaceae). *American Journal of Botany* 99: 690-699.

Rajora OP, DeVerno L, Mosseler A, Innes D. 1998. Genetic diversity and population structure of disjunct Newfoundland and central Ontario populations of eastern white pine (*Pinus strobus* L.). *Canadian Journal of Botany* 76: 500-508.

Rajora OP, Rahman MH, Buchert GP, Dancik BP. 2000. Microsatellite DNA analysis of genetic effects of harvesting in old-growth eastern white pine (Pinus strobus) in Ontario. *Molecular Ecology* 9: 339–348.

Rajora OP, Mann IK, Shi Y.-Z. 2005. Genetic diversity and population structure of boreal white spruce (*Picea glauca*) in pristine conifer-dominated and mixed-wood forest stands. *Canadian Journal of Botany* 83:1096-1105*.*

Yeh FC, Khalil MAK, El-Kassaby YA, Trust DC. 1986. Allozyme variation in *Picea mariana* from Newfoundland: genetic diversity, population structure, and analysis of differentiation. *Canadian Journal of Forest Research* 16: 713-720.

Zinck JWR, Rajora OP. 2016. Post-glacial phylogeography and evolution of a wide-ranging highly-exploited keystone forest tree, eastern white pine (*Pinus strobus*) in North America: Single refugium, multiple routes. *BMC Evolutionary Biology* 16:56; DOI 10.1186/s12862-016-0624-1.
